# Discovery of Dual MER/AXL Kinase Inhibitors as Bifunctional Small Molecules for Inhibiting Tumor Growth and Enhancing Tumor Immune Microenvironment

**DOI:** 10.1021/acs.jmedchem.4c00400

**Published:** 2024-06-24

**Authors:** Mu-Chun Li, You-Liang Lai, Po-Hsien Kuo, Julakanti Satyanarayana Reddy, Chih-Ming Chen, Julakanti Manimala, Pei-Chen Wang, Ming-Shiem Wu, Chun-Yu Chang, Chen-Ming Yang, Chin-Yu Lin, Yu-Chen Huang, Chun-Hsien Chiu, Ling Chang, Wen-Hsing Lin, Teng-Kuang Yeh, Wan-Ching Yen, Hsing-Pang Hsieh

**Affiliations:** † Institute of Biotechnology and Pharmaceutical Research, National Health Research Institutes, Miaoli County 350401, Taiwan, ROC; ‡ Biomedical Translation Research Center (BioTReC), Academia Sinica, Taipei City 115202, Taiwan, ROC; § Department of Chemistry, National Tsing Hua University, Hsinchu City 300044, Taiwan, ROC

## Abstract

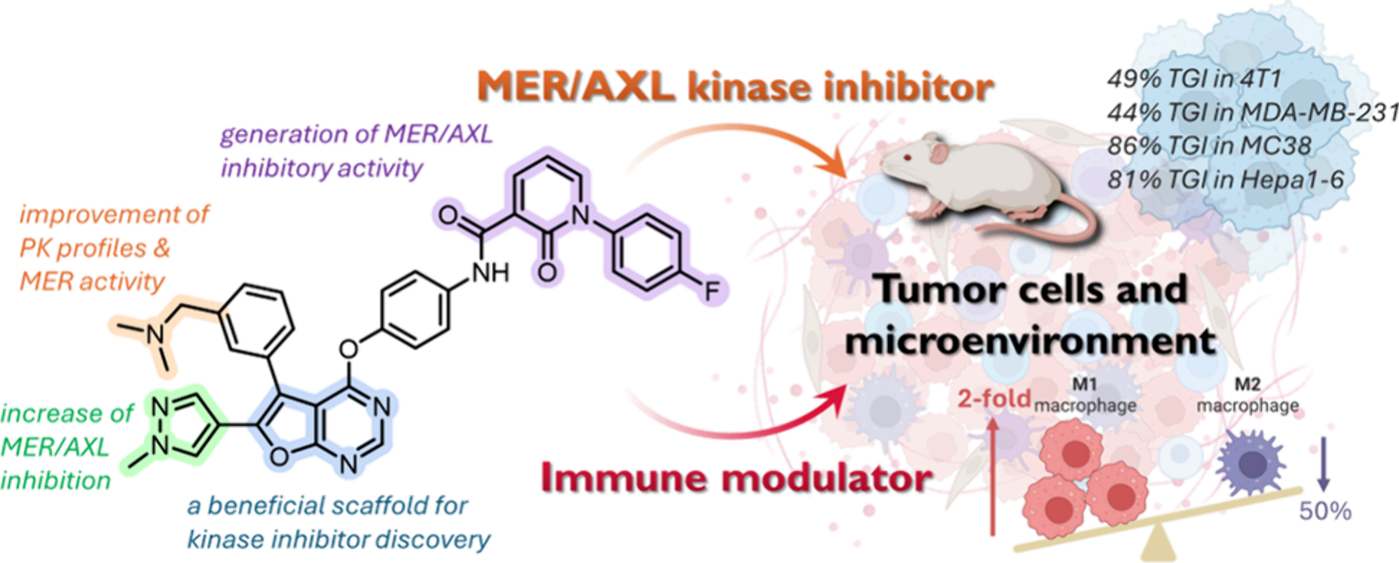

A series of bifunctional compounds have been discovered for their dual functionality as MER/AXL inhibitors and immune modulators. The furanopyrimidine scaffold, renowned for its suitability in kinase inhibitor discovery, offers at least three distinct pharmacophore access points. Insights from molecular modeling studies guided hit-to-lead optimization, which revealed that the 1,3-diketone side chain hybridized with furanopyrimidine scaffold that respectively combined amino-type substituent and 1*H*-pyrazol-4-yl substituent on the top and bottom of the aryl regions to produce **22** and **33**, exhibiting potent antitumor activities in various syngeneic and xenograft models. More importantly, **33** demonstrated remarkable immune-modulating activity by upregulating the expression of total T-cells, cytotoxic CD8^+^ T-cells, and helper CD4^+^ T-cells in the spleen. These findings underscored the bifunctional capabilities of **33** (**BPR5K230**) with excellent oral bioavailability (*F* = 54.6%), inhibiting both MER and AXL while modulating the tumor microenvironment and highlighting its diverse applicability for further studies to advance its therapeutic potential.

## Introduction

The TAM (TYRO3, AXL, and MER) family of receptor tyrosine kinases (RTKs) are characterized by a combination of two immunoglobulin-like (IgL) domains, dual fibronectin type III (FNIII) repeats in the extracellular region and a cytoplasmic kinase domain.^1^ TAM RTKs play important roles in innate immunity and homeostasis,^2,3^ and dysregulation of TAM RTKs has been implicated in the pathogenesis and progression of cancer.^4,5^ Of note, MER and AXL are known to be overexpressed in various types of hematological and solid tumor cancers and have been reported as poor prognostic factors. Their signaling pathways in primary tumors are associated with cancer progression, mesenchymal phenotype, metastasis, and drug resistance in both solid tumors and hematologic cancers.^6^ AXL and MER have also been recognized as potential negative immune regulators that suppress host tumor immune responses. AXL signaling suppresses proinflammatory Toll-like receptor (TLR) responses in antigen-presenting cells (APCs).^7^ During apoptotic cell ingestion, MER suppresses the M1 macrophage proinflammatory cytokine response and enhances M2 macrophage anti-inflammatory cytokine production.^8^ Recent preclinical studies demonstrated that MER mediates intrinsic and adaptive resistance to AXL-targeting agents in human head and neck squamous cell carcinoma (HNSCC), triple-negative breast cancer (TNBC), and non-small cell lung cancer (NSCLC). The combined MER and AXL therapies led to a more potent blockade of downstream signaling and resulted in synergistic growth inhibition in human TNBC and HNSCC xenograft models.^9^ Using mouse models of murine breast tumors, it is demonstrated that both MER and AXL receptors cooperate to promote breast cancer progression and immune escape. AXL drives the aggressiveness of tumors by affecting stemness, migration, invasion, and epithelial-to-mesenchymal transition (EMT), while MER affects immunogenic signals in the tumor microenvironment through efferocytosis and production of cytokines that impinge on the tumor milieu.^10^ Thus, therapeutic MER and AXL inhibition would reverse treatment resistance, reduce tumor cell survival and metastatic capacity, and, at the same time, create a more robust antitumor immune response. Successfully developing MER/AXL dual kinase inhibitors would have significant impacts on cancer patients’ treatment outcomes and overall survival. Furthermore, modulating the immune system through small molecule approaches could potentially synergize with biological modalities when used in combination.

**Figure 1:**
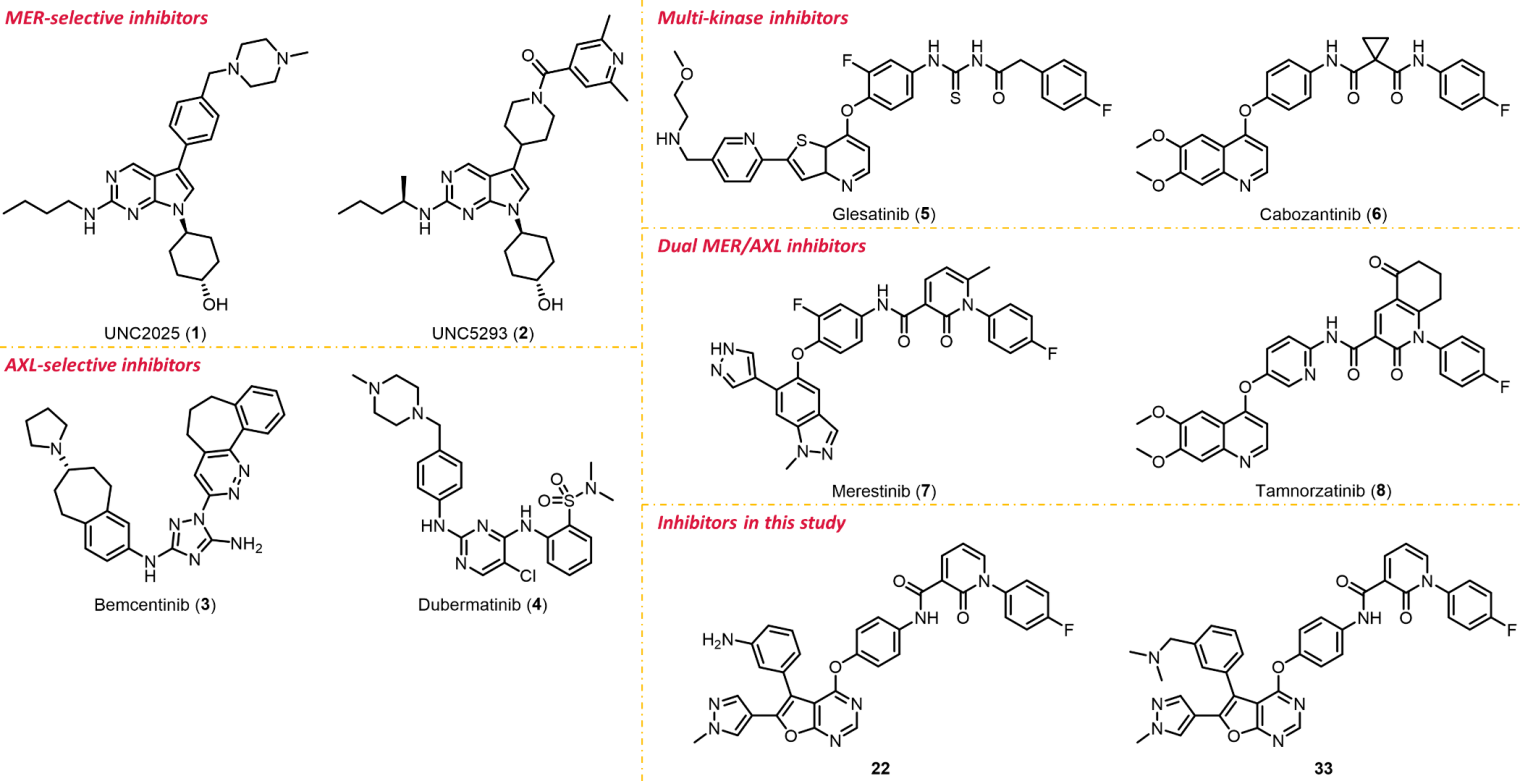
Representative MER-selective, AXL-selective, or dual MER/AXL inhibitors.

As shown in Figure 1, several type I (**1**–**4**) and type II (**5**–**8**) ATP-competitive MER or AXL inhibitors are currently under various investigation stages (Figure 1). UNC2025 (**1**) is a well-known MER-selective inhibitor with an extraordinary IC_50_ value of 0.74 nM and also potently suppressed FLT3 activity with an IC_50_ value of 0.80 nM.^11,12^ UNC5293 (**2**), another potent and orally bioavailable MER selective inhibitor (IC_50_ = 0.9 nM), exhibits a highly selective profile.^13^ Bemcentinib (BGB324 or R428, **3**) is a potent AXL selective inhibitor (IC_50_ = 14 nM) with more than 10-fold selectivity against MER and TYRO3 and also showed good inhibitory activities against TIE-2/TEK (4-fold), RET (9-fold), and FLT1/4 (8-fold and 5.5-fold).^14^ Currently, bemcentinib (**3**) is in a phase II clinical trial with pembrolizumab for treating advanced NSCLC patients.^15^ Dubermatinib (TP-0903, **4**) is another promising AXL selective inhibitor (IC_50_ = 27 nM), which is under investigation in several clinical trials in patients with advanced solid tumors or leukemia (e.g., AML, CLL, SLL).^16^ Regarding the similarities of ATP binding sites between TAMs and other RTKs, drugs targeting TAMs may also exhibit inhibitory effects against various oncogenic RTKs, such as MET, RON, FLT3, VEGFR2, and so forth.^17^ Several multitargeting MET inhibitors have shown strong inhibitory abilities against MER and AXL, including glesatinib (**5**), cabozantinib (**6**), and others with nanomolar-level IC_50_ values (Figure 1).^18−21^ Notably, merestinib (LY2801653, **7**) and tamnorzatinib (ONO-7475, **8**), identified as highly potent dual MER/AXL inhibitors, also behaved strong inhibitory abilities against MET protein.^22,23^ In addition to the aforementioned clinical candidates, numerous MER-selective, AXL-selective, or dual MER/AXL inhibitors are currently under study at various stages of development.^24−31^

Previously, we have reported a series of furanopyrimidine compounds being second-generation or third-generation epidermal growth factor receptor (EGFR) orally active inhibitors for the treatment of NSCLC.^32,33^ We continued to take advantage of the drug-like properties and the structural advantage of furanopyrimidine scaffold to develop orally bioavailable drugs. To achieve the discovery of dual MER/AXL inhibitors, we executed the hybridization of our furanopyrimidine scaffold and 1,3-diketone fragments widely utilized by MET inhibitors (applied in **5**–**8**) to identify a series of furanopyrimidine compounds as promising dual MER/AXL inhibitors in this study. Inhibitors **22** and **33** demonstrated strong *in vitro* with both MER and AXL at nanomolar levels and *in vivo* potent efficacy in both syngeneic and mouse models as well as human xenograft tumor models.

## Results and Discussion

### Initial SAR Study Started with the Hybridization with an EGFR Clinical Candidate Followed by Attenuation of the EGFR Inhibition

Our dual MER/AXL inhibitors discovery campaign began with the analogue (**10**)^34^ of our clinical candidate DBPR112 (**9**),^32^ which was potent to suppress wild-type (WT) EGFR and double mutant (DM; T790M/L858R) EGFR enzymes, but no effect against MER and AXL enzymes (Table 1). In the first-round screening, MER-selective inhibitor **1** and AXL-selective inhibitor **3** were used as reference compounds. To replace the hydrophobic moiety of **10** to achieve MER and AXL activity with attenuating EGFR activity, we obtained **11** by taking the place of the (*S*)-2-phenylglycinol moiety of **10** with a 1,3-diketone fragment, which was inspired by the pan-TAM inhibitor **6** and our previous studies.^34^ However, despite that **11** showed potent inhibition against AXL enzyme, **11** exhibited weak activity against MER enzyme. In addition, the Michael acceptor moiety still made **11** a potent anti-EGFR^DM^ compound with an IC_50_ value of 42 nM. In order to further abolish EGFR activities by removing the Michael acceptor of **11** to obtain **12**, we unexpectedly observed that the MER activity of **12** was increased significantly accompanied by a massive decrease in wild-type and double mutant EGFR activities. Furthermore, bearing 5-phenyl (**13**) or 5-hydrogen (**14**) furanopyrimidine analogues attenuated the MER and AXL activities. In summary, the 5-aniline and 1,3-diketone fragments of hit **12** played crucial roles that thoroughly overturned its enzymatically inhibitory ability from EGFR^WT^ and EGFR^DM^ to MER and AXL.

**Table 1:**
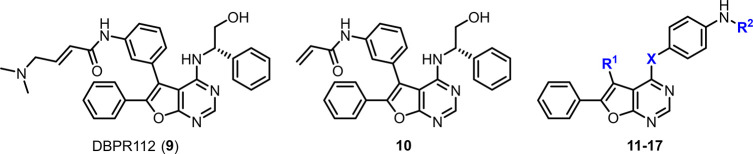

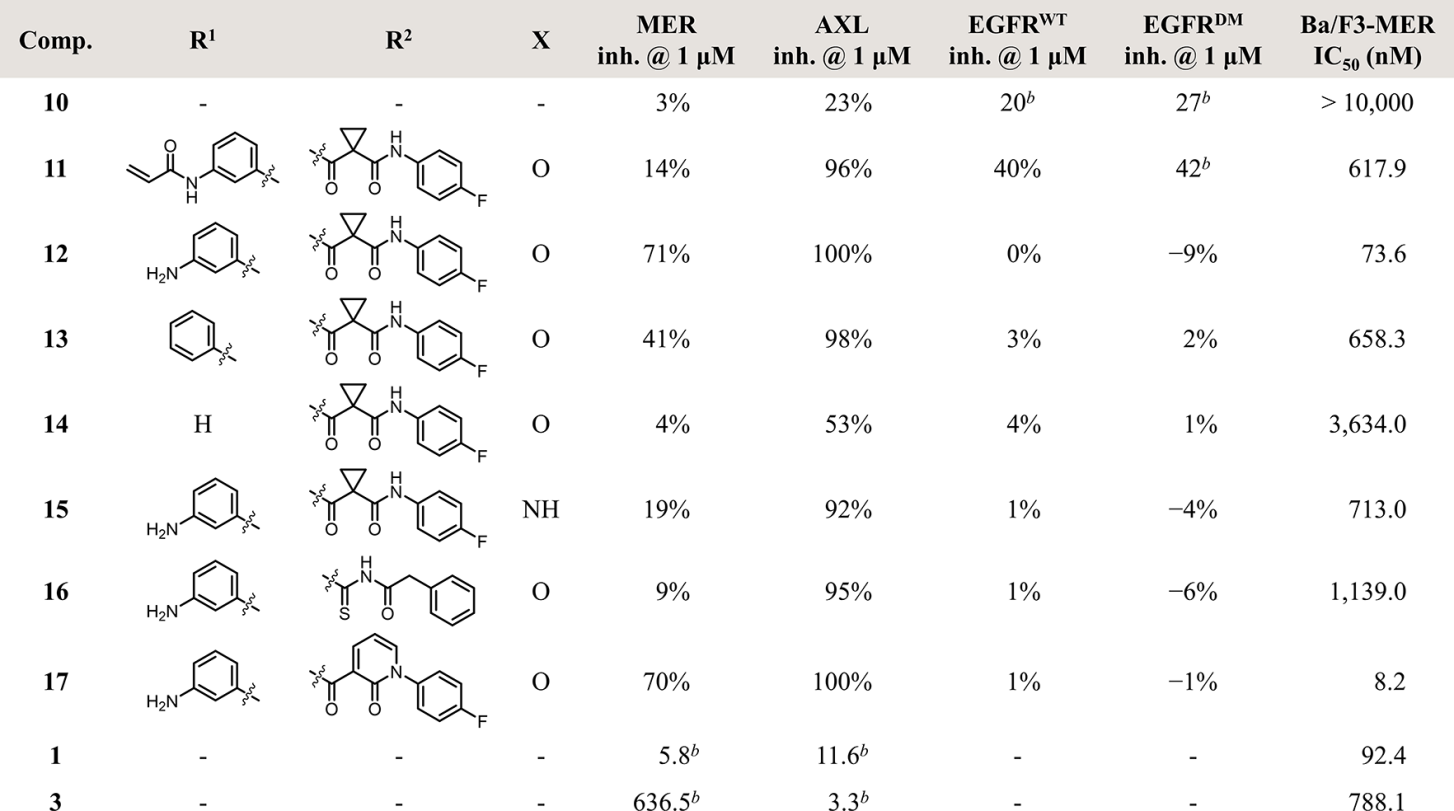
Initial SAR Exploration from Hybridization^a^

a The inhibition values of MER, AXL, EGFR^WT^, and EGFR^DM^ were performed using in-house Kinase-Glo assay; UNC2025 (**1**) and bemcentinib (**3**) were used as controls; all data are expressed as the mean of at least two independent experiments and are mostly within 15% error margins.

b IC50 value.

To examine the role of oxygen at the 4-position of the furanopyrimidine scaffold, **15** was synthesized and displayed a significant loss on MER inhibition, but AXL inhibition remained, as compared to **13**. By introducing different side chains, which are favorable to providing MET activity, **16** and **17** were implanted in the side chains of **5** and **7**, respectively, which maintained the anti-AXL potency. However, **16** lost the inhibitory activity against MER enzyme while **17** remained identical ability as **12**, showing that the 1,3-diketone moiety is pivotal for MER inhibition. To our success of first-round screening, both **12** and **17** displayed potent enzymatic MER and AXL inhibition. More importantly, **17** even showed excellent single-digit antiproliferative potency against Ba/F3-MER cells with an IC_50_ value of 8.4 nM, which was much better than **12** (9-fold) and the reference compound **1** (11-fold). These findings offered an opportunity for further investigation of the structure–activity relationship study of compound **17**.

### Binding Mode Analysis of **17**

**Figure 2:**
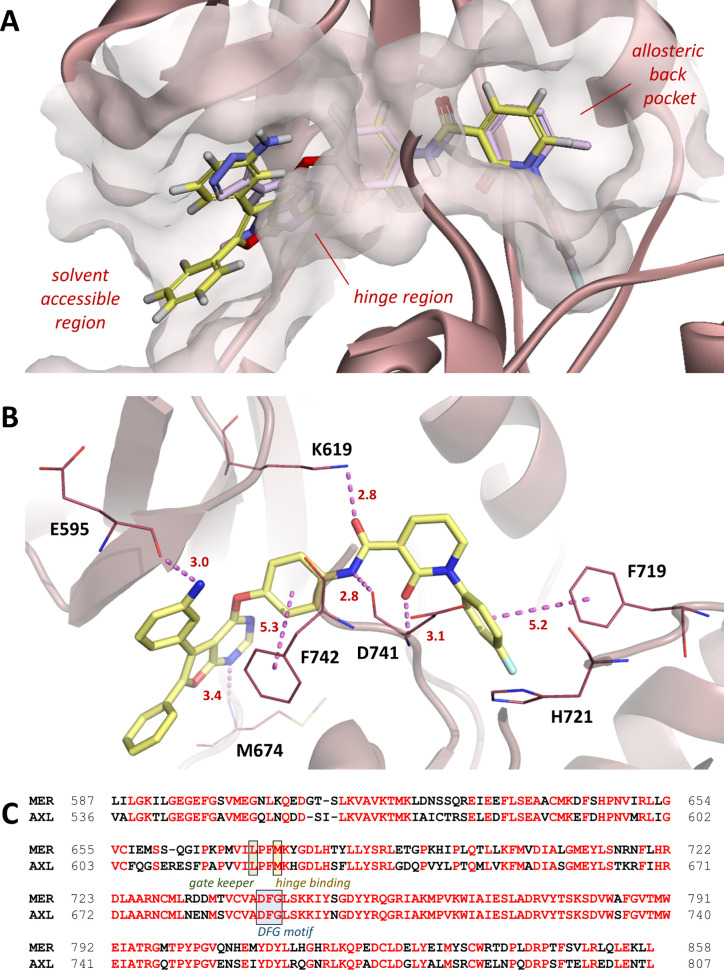
Binding mode analysis of **17**. (A) Superimposition of merestinib (**7**, pink) and **17** (yellow) (PDB: 7AAY). (B) Molecular docking of **17** (yellow) into MER kinase domain (PDB: 7AAY). Hydrogen bonding is shown as violet. Bond length unit, Å. (C) Sequence alignment of MER and AXL kinase domains, where the identical amino acid residues are colored in red.

To understand the binding modes of **17** with MER protein, the molecular modeling study was carried out with the X-ray cocrystal structure of MER and merestinib (**7**) (PDB: 7AAY).^35^ Merestinib (**7**) is a known type II MET inhibitor with potent IC_50_ values of 0.8 and 11 nM against MER and AXL enzymes, respectively.^22^ The superposition of type II cocrystal MER-merestinib complex and **17** is shown in Figure 2A, and the binding mode analysis is shown in Figure 2B. As depicted in Figure 2A, **17** adopts a type II binding mode with MER, while **17** and **7** overlap extensively in the ATP binding site. In addition, the 1,3-diketone moiety of **17** located in the allosteric back pocket and the two phenyl rings on the furanopyrimidine scaffold faced the solvent-accessible region. Binding mode analysis (Figure 2B) indicated that the 1-nitrogen of the scaffold formed the crucial hinge interaction with the backbone of Met674. The phenyl ring, acting as a linker between the scaffold and the 1,3-diketone group, faced the Phe742 from the DFG motif. This π–π interaction, a typical type II binding mode, achieved the DFG-out conformation. The oxygen and amide NH from the backbone of Asp741 individually formed bidentate hydrogen bonds with **17**. The terminal phenyl ring was perpendicular to the Phe719 on the α-helix A forging another π–π interaction. Besides, the amino group on the 5-phenyl ring reacted with the Glu595, which is close to the solvent accessible region. Intriguingly, the phenyl ring on the 6-position of the scaffold made none of the contribution to the interaction between **17** and MER protein, and this finding spurred us to explore potential functional groups to raise inhibitory activity. We would also like to demonstrate the binding modes between **17** and AXL; however, none of the X-ray cocrystal structures of AXL kinase in complex with a type II inhibitor has been published so far. Notably, the kinase domains of MER and AXL are highly similar sharing nearly 70% identity overall (Figure 2C),^36^ suggesting that a type II inhibitor might bind to MER and AXL enzymes with similar binding modes.

### SAR Exploration of the Functional Groups Close to the Solvent Accessible Region

To enhance the inhibitory ability against MER enzyme and maintain the inhibitory ability against AXL enzyme, several substituents were introduced to replace the 6-phenyl ring in a series of analogue **17** (Table 2). Removal of the 6-phenyl ring in **18** or replacement with bromide in **19** resulted in a significant loss of potency against MER kinase and Ba/F3-MER cells. The cellular potency was recovered by the introduction of a methyl group in **20** but still 2-fold weaker than **17** against Ba/F3-MER cells. The above results showed that the modification at the 6-position affected the activity in MER rather than AXL, suggesting that the solvent accessible region near the 6-position may not play an important role in determining the activity of AXL. The strong potency was retrieved by the incorporation of a 1*H*-pyrazol-4-yl group in **21**. The inhibitory abilities of **21** against both enzymatic and cellular assays were nearly identical to those of **17**, indicating that an aromatic substituent at 6-position is pivotal for the activity of MER protein. More interestingly, the introduction of an additional methyl group in **22**, building upon the modifications from **21**, has resulted in the best IC_50_ value observed so far with both single-digit enzyme and cellular activities. Moreover, **22** demonstrated superior antiproliferative activity against Ba/F3-MER cells, further highlighting its potential as a promising candidate for MER/AXL dual inhibitory activities. Intriguingly, the introduction of an additional methyl group of methyl-1*H*-pyrazol-4-yl **22** to obtain 1,3-dimethyl-1*H*-pyrazol-4-yl **23** led to a decrease in potency. It is speculated that the additional methyl group may have interfered with the rotation of the pyrazole ring. At the same token, this hypothesis was also observed that the 3,5-dimethylisoxazol-4-yl group in **24** abolished MER activity as well as dramatically decreased AXL activity. The two methyl groups blocked the rotation of the 5-membered aromatic ring and further caused a loss of important interactions or conformational changes necessary for optimal binding to the target proteins. By changing the orientation of the pyrazole ring from 1*H*-pyrazol-4-yl **22** to 1*H*-pyrazol-3-yl **25**, **25** exhibited 2- to 7-fold weaker activity in all enzymatic and cellular assays. In the presence of a thiophen-3-yl ring at the 6-position in **26**, an enhancement of inhibitory activity against MER and AXL enzymes was observed, suggesting that MER activity largely affected different heteroaromatic rings, but AXL activity was slightly influenced by heteroaromatic rings. The most promising dual MER/AXL inhibitor **22** was then examined for its pharmacokinetics (PK) profile and potential for further development (Table 4). The PK study was conducted in mice, and key parameters such as half-life, area under the curve (AUC), and others were monitored following intravenous (iv) and oral (po) administration of 3 mg/kg of **22**. To improve the moderate solubility of **22**, it was converted into a hydrochloride salt. **22** exhibited a reasonable PK profile, including an acceptable drug exposure represented by the area under the curve (AUC_(0–inf)_). However, the oral bioavailability (*F*) of **22** was determined to be 19.2%, which was considered relatively low.

**Table 2:**
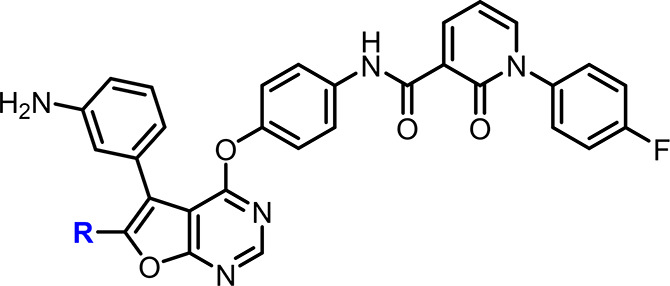

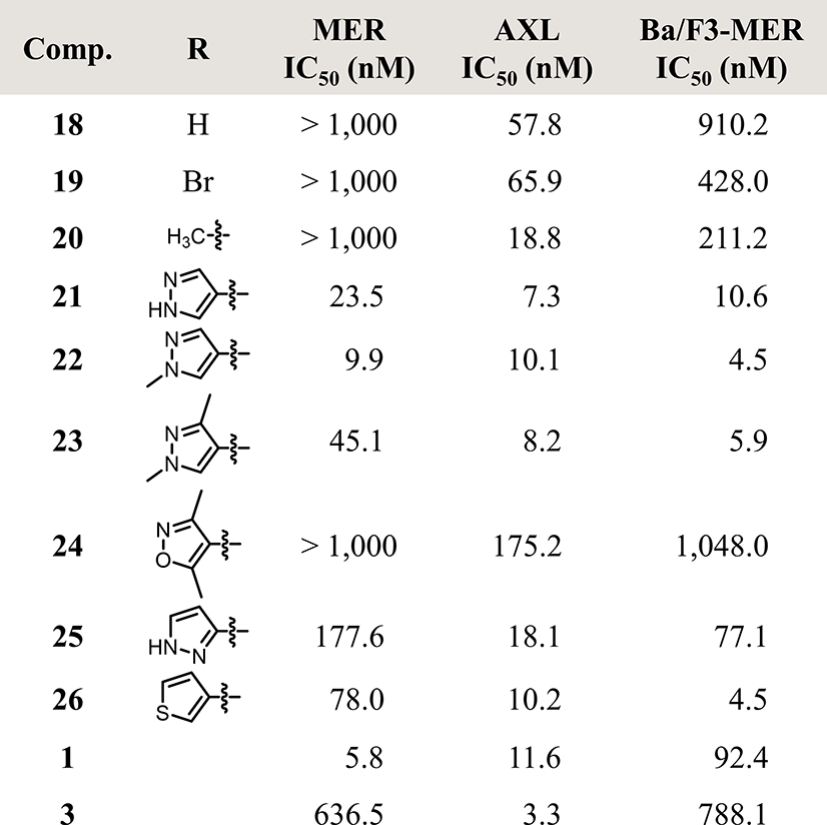
SAR Exploration of 6-Substituted Furanopyrimidines^a^

a The IC_50_ values of MER and AXL were performed using in-house Kinase-Glo assay; UNC2025 (**1**) and bemcentinib (**3**) were used as controls; all data are expressed as the mean of at least two independent experiments and are mostly within 15% error margins.

**Table 3:**
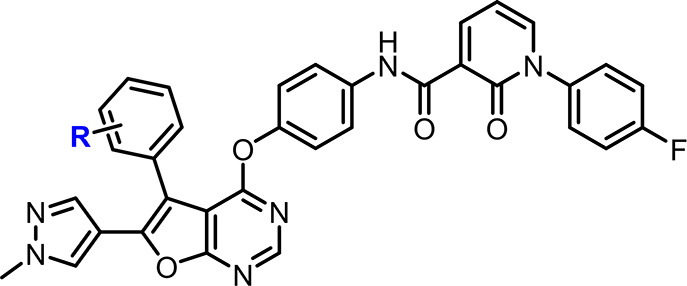

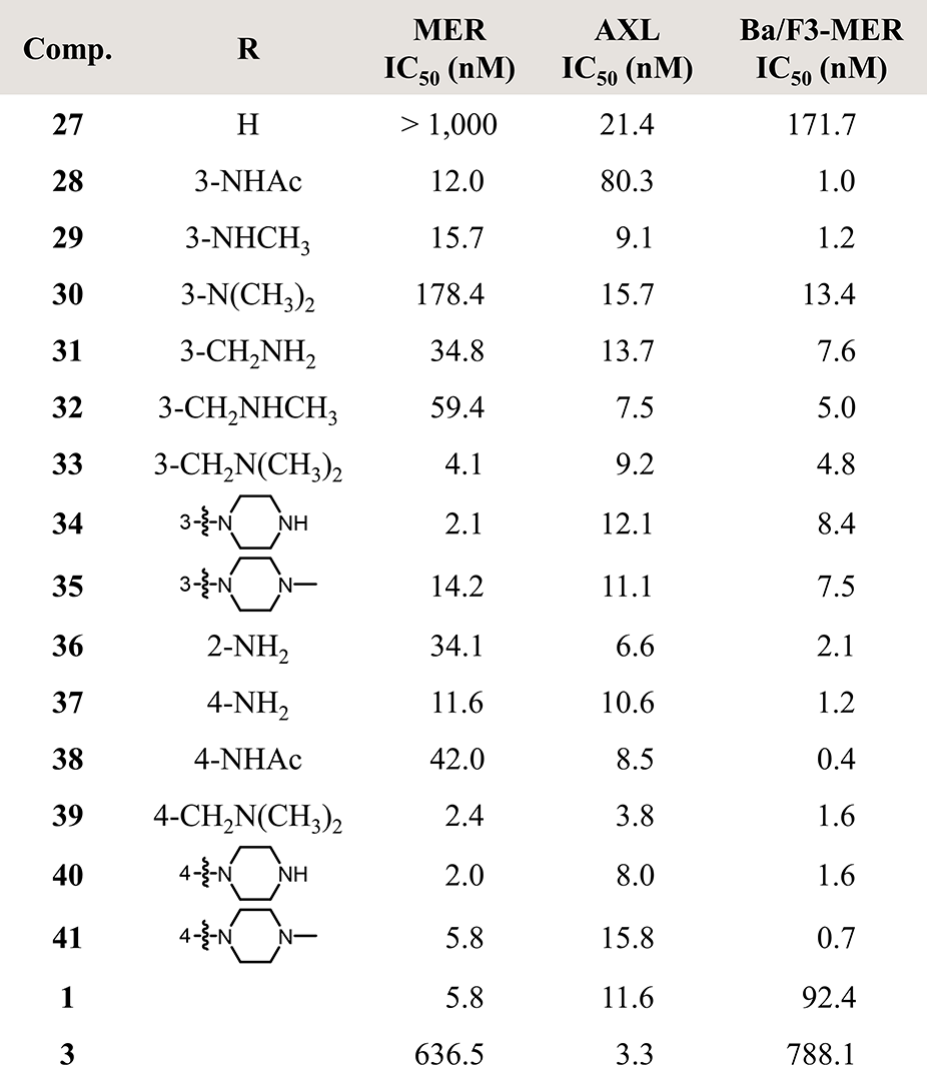
SAR Exploration of Substitution on 5-Phenyl Ring^a^

a The IC_50_ values of MER and AXL were performed using in-house Kinase-Glo assay; UNC2025 (**1**) and bemcentinib (**3**) were used as controls; all data are expressed as the mean of at least two independent experiments and are mostly within 15% error margins.

**Table 4:**
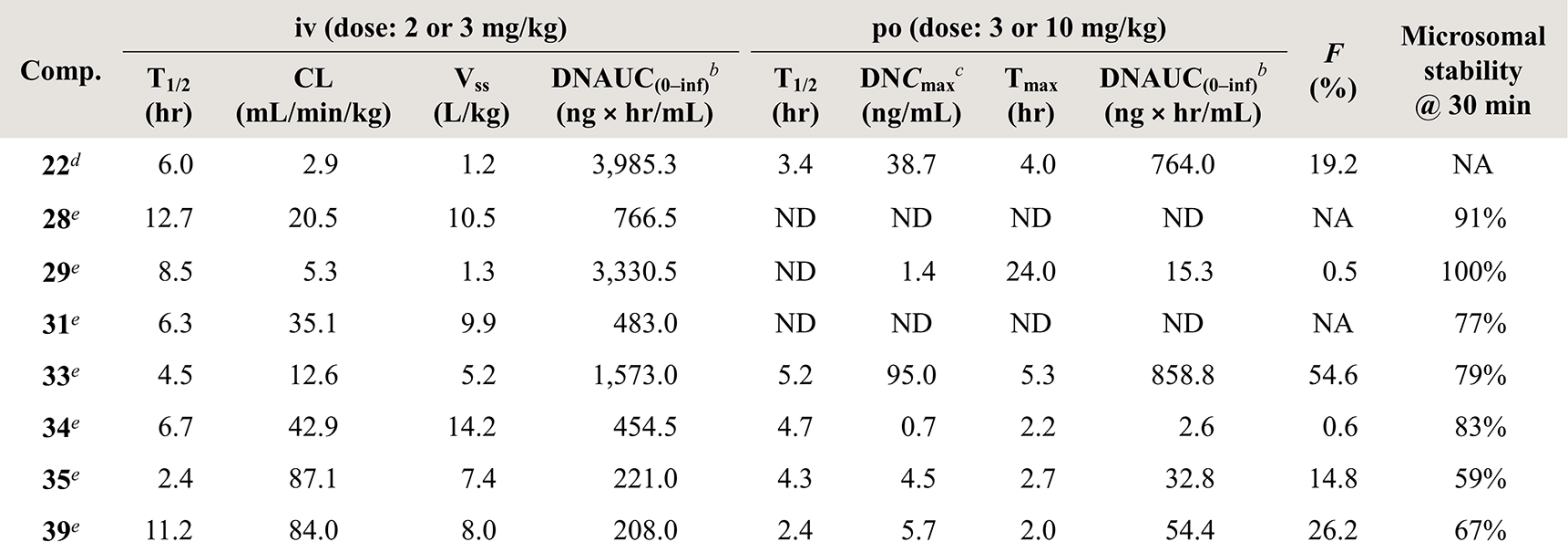
Pharmacokinetics Profile of Potential Compounds in Mice^a^

a *T*_1/2_, half-life; CL, clearance; *V*_ss_, steady state distribution volume; DNAUC_(0–inf)_, dose-normalized area under curve from zero to time infinity; DN*C*_max_, dose-normalized maximum plasma concentrations; *T*_max_, time to *C*_max_; *F*, oral bioavailability; ND, not determined; NA, not available.

b DNAUC_(0–inf)_ = AUC_(0–inf)_/dose.

c DN*C*_max_ = *C*_max_/dose.

d iv dosage and po dosage are 3 mg/kg.

e I.V. dosage is 2 mg/kg, and P.O. dosage is 10 mg/kg.

### Improvement of Pharmacokinetics Profile

The AUC_(0–inf)_ value per dose of **22** by i.v. administration is much higher than that of **22** by p.o. administration, which indicates that **22** may have an oral permeability problem. Having established the low water solubility of **22**, our subsequent efforts focused on implementing polar or solubilizing substituents at the 5-phenyl ring for the PK improvement campaign (Table 3). We initially removed the *m*-amino group from **22**, **27** attenuated its MER inhibitory ability, and AXL inhibitory ability was decreased by 2-fold. The findings suggested that the amino group of aryl substituents at the 5-position of the furanopyrimidine scaffold is critical for enzymatic binding affinity, particularly in relation to MER activity. These observations aligned with the results obtained for **12** and **13**, as highlighted in the initial stage of the SAR study. Adding an acyl group on the *m*-amino in **28** showed similar potency against MER enzyme compared to **22** but 8-fold weaker potency against AXL enzyme. Substituting the *m*-amino group of **22** with one or two methyl groups led to different outcomes; monomethyl **29** showed identical potency to **22**, but dimethyl **30** showed poor inhibition against MER and AXL kinases. Furthermore, when the *m*-amino of **22** was replaced by an *m*-aminomethyl in **31** or a *m*-(methylamino)methyl in **32**, the inhibitory ability against MER protein decreased by 3- to 6-fold but maintained its ability to AXL protein. Most interestingly, **33** with the *N*,*N*-(dimethylamino)methyl substituent at the meta-position on the 5-phenyl ring exhibited single-digit nanomolar potency against both MER and AXL enzymatically activities as well as an IC_50_ value of 5 nM suppressing Ba/F3-MER cells.

We also introduced cyclic solubilizing groups at the *meta*-position, a piperazinyl in **34** and a 4-methylpiperazinyl in **35**, and both compounds showed strong inhibitory effects against MER and AXL enzymes. Next, we altered the position of substituents to explore how different structural modifications affect inhibitory abilities. **36** and **37**, the former with an *ortho*-amino group and the latter with a *para*-amino group, exhibited equal-level inhibitory potency against AXL kinase compared to **22**, but only **36** exhibited one-third of the inhibitory potency against MER kinase. By imitating the strategy of introducing solubilizing groups at the *meta*-position and observing their effect, we attempted to introduce similar solubilizing groups at the *para*-position. Among **38**–**41**, only **38** with an acyl substituent showed a 3.5-fold decrease in potency against MER protein compared to its counterpart **28**. The others (**39**–**41**) all displayed increased inhibitory abilities against MER enzyme and maintained identical potency in inhibiting AXL enzyme, compared to their counterparts (**33**–**35**).

Having several promising dual MER/AXL inhibitors in hand, we selected **28**, **29**, **31**, **33**, **34**, **35**, and **39** to evaluate their pharmacokinetics parameters due to their excellent antiproliferative ability against Ba/F3-MER cells and compared them to **22**. Apart from **28**, the analogues were also converted into hydrochloride salts as **22**. Among the tested compounds, **28**, **29**, and **31** demonstrated unfavorable PK profiles, possibly attributable to their poor solubility characteristics. Both **34** and **35**, which feature piperazine substituents, exhibited high clearance rates after administration with 42.9 mL/min/kg for **34** and 87.1 mL/min/kg for **35**. In our system, the presence of *N*,*N*-(dimethylamino)methyl group has a significant impact on the PK data. **33** and **39** displayed improved PK profiles compared to **22**, and the oral bioavailability of both compounds exceeded 20%. According to the parameters of DN*C*_max_ and DNAUC_(0–inf)_ of **33**, we speculated that **33** would demonstrate satisfactory efficacy in further development.

### Effect of **33** on Inhibition of MER and AXL Phosphorylation *In Vitro*

**Figure 3:**
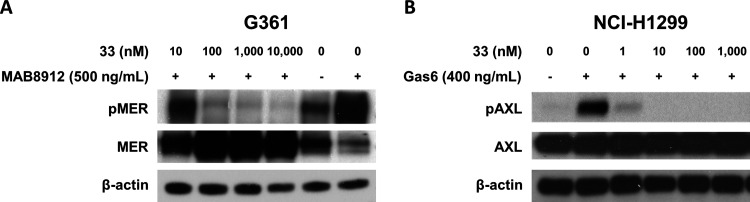
Inhibition of MER and AXL phosphorylation by **33** in tumor cell lines. (A) G361 cells were treated with **33** for 48 h followed by 30 min of incubation with a MER agonist antibody, MAB8912. (B) NCI-H1299 cells were treated with **33** for 1 h and then stimulated with recombinant human GAS6 for 30 min. At the end of drug treatment, cells were lysed, and the soluble protein was separated using electrophoresis on a sodium dodecyl sulfate and polyacrylamide gel and analyzed through Western blotting. Proteins were detected by exposure to the X-ray film.

The ability of **33** to inhibit MER and AXL activity was studied in a human melanoma cell line G361 with a high level of MER expression and a human NSCLC cell line H1299 with a high level of AXL expression by Western blotting. As shown in Figure 3, **33** treatment effectively blocked MER phosphorylation induced by anti-human MER monoclonal antibody MAB8912 in G361 cells and Gas6-mediated AXL phosphorylation in H1299 cells. These results validated the capability of **33** to effectively inhibit MER and AXL within the cellular environment.

### Antitumor Efficacy and Immunomodulatory Activities of **22** and **33***In Vivo*

**Figure 4:**
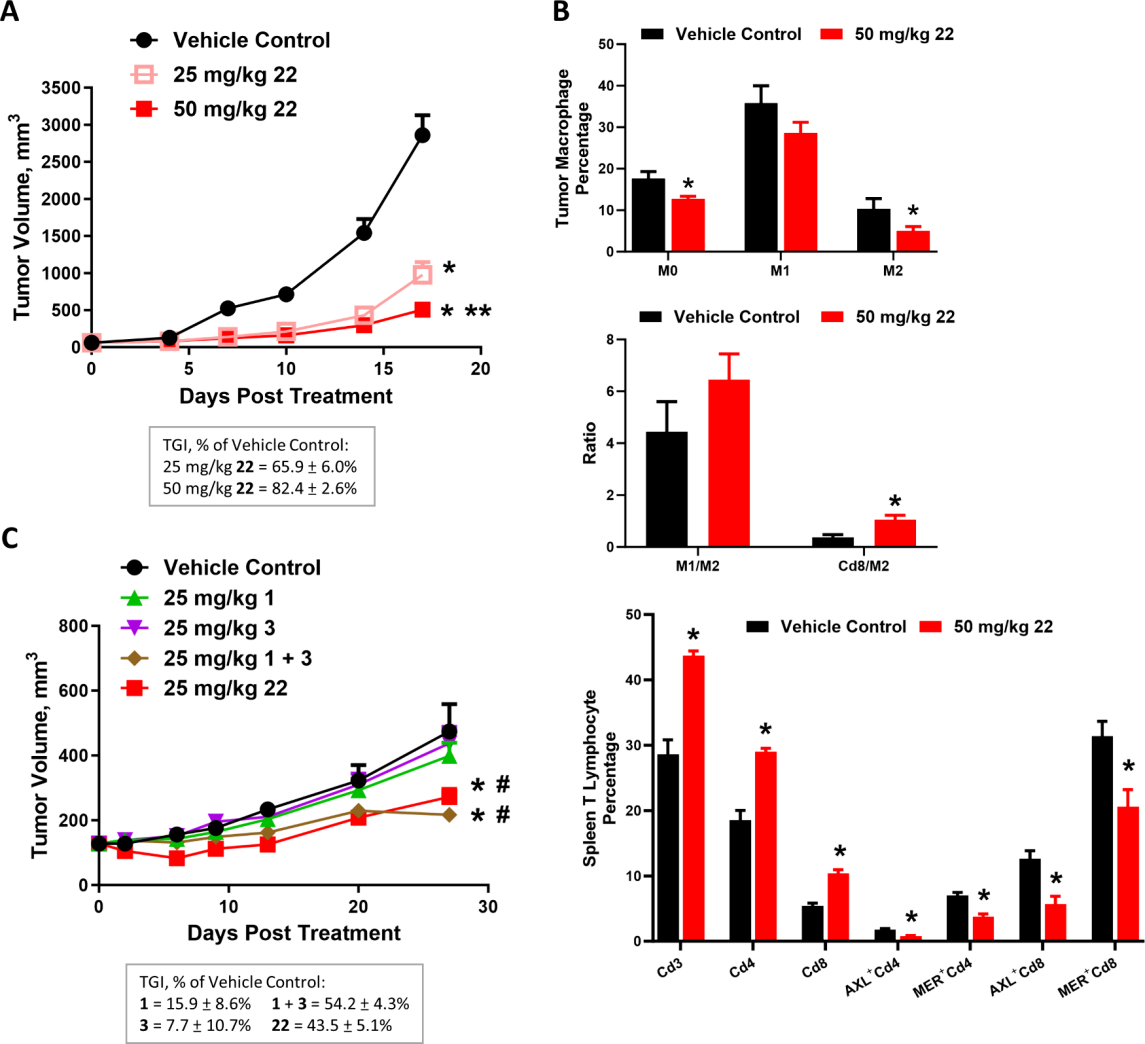
Antitumor efficacy and immunomodulatory activities of **22***in vivo*. (A) Dose-dependent antitumor effect of **22**. MC38 murine colon tumor-bearing mice were dosed orally BID at 25 or 50 mg/kg and FOTO regimen for 3 weeks. (B) Immunomodulatory activities of **22**. MC38 tumor-bearing mice were treated with 50 mg/kg **22** orally BID and FOTO regimen for 2 weeks. Thereafter, tumor and spleen tissues were harvested and isolated into single cells. The immune cells were analyzed by flow cytometry analyses. (C) MER and AXL dual inhibition within the tumor by **22***in vivo*. MDA-MB-231 human triple-negative breast xenograft tumors were treated with **22**, MER-selective agent **1**, and AXL-selective agent **3**, either alone or in combination. All compounds were dosed at 25 mg/kg orally BID and FOTO regimen for 4 weeks. Data were expressed as mean ± SEM, *n* = 8 mice per group for panels (A) and (C) and *n* = 5–7 per group for panel (B). *p < 0.05 vs vehicle control, **p < 0.05 vs 25 mg/kg. **22** measured using one-way ANOVA and Bonferroni post-test comparison. #: *p* < 0.05 vs **1** and **3** single agents. Error bars in some data points are smaller than the symbols.

The antitumor efficacy and the immunomodulatory activity of **22** were examined in a murine colon tumor MC38 syngeneic model. MC38 tumor does not express MER and AXL kinases on the tumor and is utilized to evaluate the effect of **22** on the tumor immune microenvironment and its impact on tumor growth.^21^ Six to seven-week-old immunocompetent female C57BL/6 tumor-bearing mice at an average tumor volume of 50–60 mm^3^ were treated with **22** orally at 25 or 50 mg/kg twice a day (BID) with a five-days-on and two-days-off (FOTO) treatment schedule for 3 weeks. As shown in Figure 4A, the treatment with **22** delayed the tumor growth with tumor growth inhibition (TGI) values of 65.9 ± 6.0 and 82.4 ± 2.6% at 25 and 50 mg/kg, respectively. No significant body weight loss was observed during the treatment period (Figures 4A and S1).

To evaluate the immunomodulatory activity of **22**, tumor-bearing animals (average tumor volume around 150 mm^3^) were treated with 50 mg/kg of **22** orally BID and FOTO for 2 weeks. Thereafter, tumor and spleen tissues from the control and treated animals were harvested and the immune cells were analyzed by flow-cytometry analysis. As seen in Figure 4B (top panel), **22** produced a 50% decrease in the percentage of pro-tumor M2-like macrophages (Cd206^+^ Cd11b^+^ F4/80^+^) without altering the percentage of antitumor M1-like macrophages (Cd86^+^ Cd11b^+^ F4/80^+^), resulting in an overall reduction in intratumoral Mo macrophage populations (CD45^+^ Cd11b^+^ F4/80^+^).^37^ The modulation of the tumor microenvironment by **22** was further confirmed by a 3-fold increase in the ratio of CD8^+^ cytotoxic T cells to M2-like macrophages within the tumor (Figure 4B, middle panel). An increase in the intratumoral M1-to-M2 ratio was also observed although it was not statistically significant (Figure 4B, middle panel). In the spleen, **22** produced an average of 1.5-fold increase in the percentage of Cd3^+^ pan-T cell and Cd4^+^ helper T cell populations and a 2-fold increase in Cd8^+^ cytotoxic T cells (Figure 4B, bottom panel). Notably, the increase in Cd4^+^ and Cd8^+^ cell populations by **22** treatment was associated with a reduction in the expression of MER and AXL enzymes on these cells in the spleen (Figure 4B, bottom panel). Collectively, these data indicate that **22** is an effective immunomodulatory agent and its antitumor efficacy is mediated by remodeling the tumor immune microenvironment.

The effect **22** on MER and AXL dual inhibition within the tumor *in vivo* was evaluated in a human MER and AXL overexpression triple-negative breast tumor MDA-MB-231 xenograft model. Female immunodeficient NOD/SCID mice-bearing subcutaneous tumors at an average tumor volume of 150 mm^3^ were treated with vehicle control, **22** at 25 mg/kg orally BID, FOTO treatment schedule for 4 weeks. MER-selective agent **1** and AXL-selective agent **3**, either alone or in combination, were included for comparison. As seen in Figure 4C (body weight change in Figure S2), 4-week treatment of either **1** and **3** alone had a limited antitumor effect, with TGI values of 15.9 ± 8.6 and 7.7 ± 10.7%, respectively. The combination of **1** and **3** delayed tumor growth with a TGI value of 52.4 ± 4.3%. Notably, **22** decreased MDA-MB-231 tumor volume by 43% vs vehicle control (TGI value is 43.0 ± 5.1% of control) and was equally efficacious as the combination of **1** and **3**, verifying **22**’s dual MER and AXL inhibitory activities.

**Figure 5:**
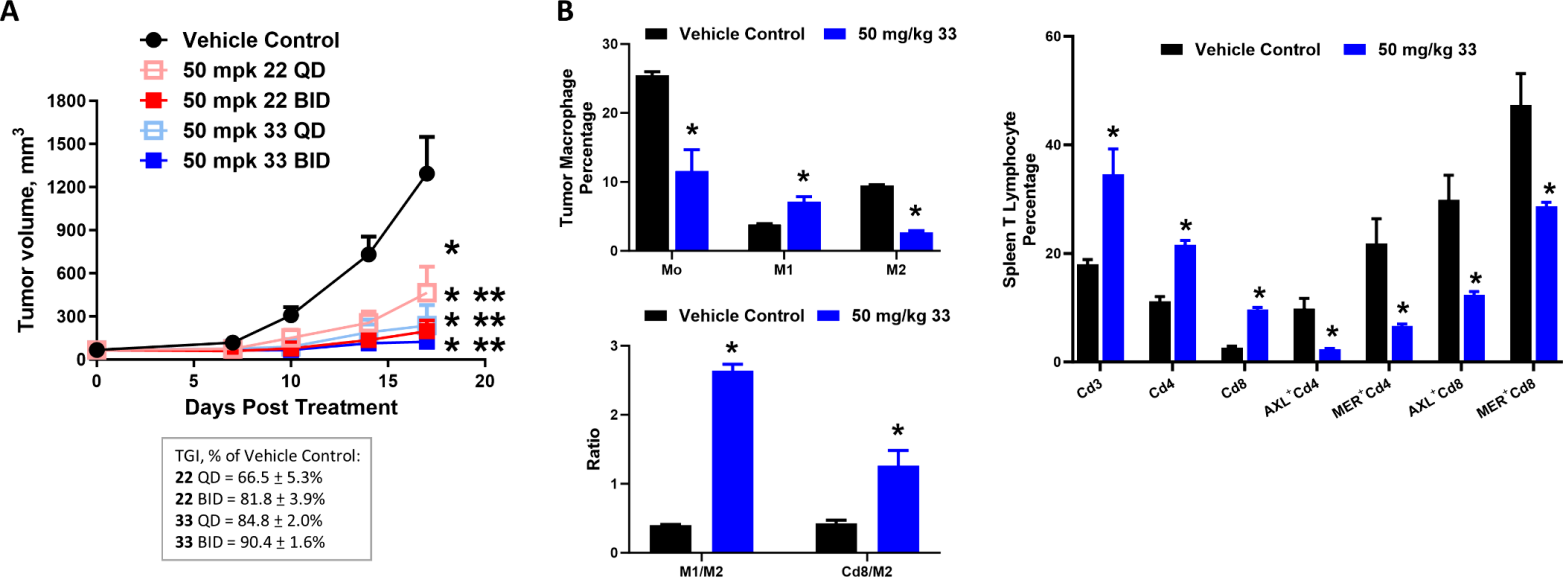
Antitumor efficacy and immunomodulatory activities of **33***in vivo*. (A) Comparison of antitumor effect of **22** and **33** in the MC38 murine colon tumor model. Tumor-bearing mice were dosed orally with either QD or BID at 50 mg/kg and FOTO regimen for 3 weeks. (B) Immunophenotyping of MC38 tumor-bearing mice treated with 50 mg/kg **33** QD and FOTO regimen for 2 weeks. Thereafter, tumor and spleen tissues were harvested and isolated into single cells. The immune cells were analyzed by flow cytometry analyses. Data were expressed as mean ± SEM, *n* = 8 mice per group for antitumor efficacy studies and *n* = 4 mice per group for immunophenotyping experiment. *p < 0.05 vs vehicle control, **p < 0.05 vs **33** measured using one-way ANOVA and Bonferroni post-test comparison. Error bars in some data points are smaller than the symbols.

To determine whether the improved PK profiles of **33** could translate into enhanced antitumor effect and immunomodulatory activities *in vivo*, we compared the antitumor efficacy of **22** BID and **33** at 50 mg/kg, either orally once a day (QD) or BID and FOTO regimen, for 3 weeks in the MC38 tumor model. As seen in Figure 5A, **22** QD and BID inhibited tumor growth by 66.5 ± 5.3 and 81.8 ± 3.9%, respectively. Importantly, treatment with **33** QD was equally efficacious as treatment with **22** BID, producing a TGI value of 84.8 ± 2.0%. No further increase in tumor growth inhibition by **33** with the BID treatment schedule was observed (TGI = 90.4 ± 1.6%). Both agents were well-tolerated without significant body weight loss during the course of the study (Figure S3).

As an immunomodulatory agent, **33** produced more pronounced effects than **22** on the immune cells in the tumor microenvironment and in the spleen. As seen in Figure 5B upper left panel, treatment with **33** reduced 50% intratumoral Mo macrophages and 70% M2-like macrophages and increased 2-fold in M1-like macrophages. The immunomodulatory activities of **33** were further demonstrated by a 4-fold increase in the ratio of M1/M2 within the tumor and a 3-fold increase in the ratio of Cd8^+^ T cells to M2 within the tumor (Figure 5B, lower left panel). In the spleen, **33** produced a 2-fold increase in the percentage of Cd3^+^ pan-T cell and Cd4^+^ helper T cell populations and a 6-fold increase in the percentage of Cd8^+^ cytotoxic T cells (Figure 5B, right panel). Compared to **22**, the expression of MER and AXL on the Cd4^+^ and Cd8^+^ cell surface was reduced to a greater extent by the treatment of **33** in the spleen (Figure 5B, right panel). The above findings demonstrated that the modification of the solubilizing group of **22** improved the PK profiles and enhanced the antitumor efficacy and immunomodulatory effects of **33**.

**Figure 6:**
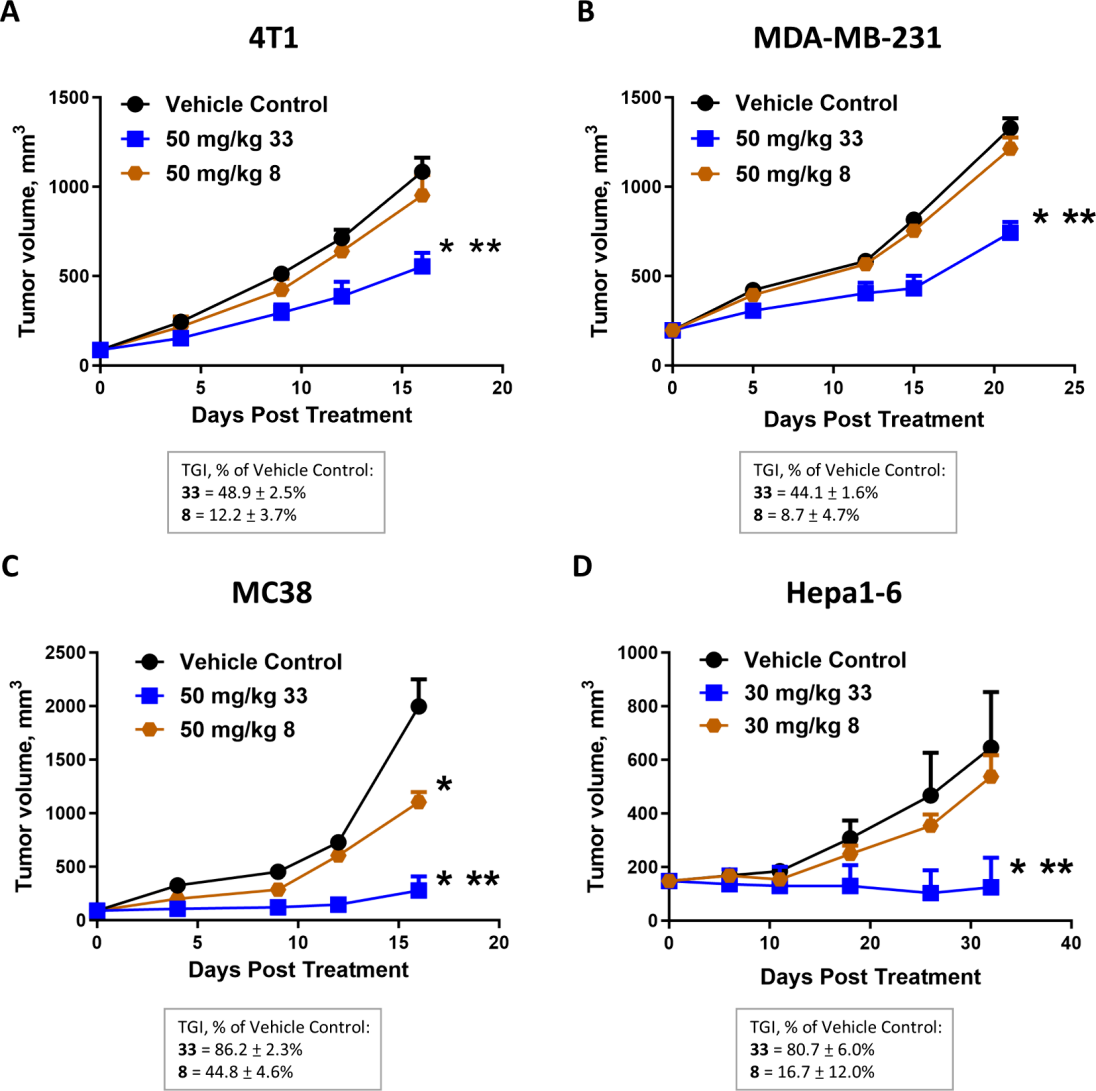
Antitumor effect of **8** and **33***in vivo*. (A) 4T1 murine triple-negative breast tumors, (B) MDA-MB-231 human triple-negative breast xenograft tumors, (C) MC38 murine colon tumors, (D) Hepa1–6 murine liver tumors. 4T1, MDA-MB-231, and MC38 tumor-bearing mice were dosed with QD at 50 mg/kg and FOTO regimen; Hepa1–6 tumor-bearing mice were dosed QD orally at 30 mg/kg and FOTO regimen. Treatment length depended on the individual tumor growth rate of each model. Data were expressed as mean ± SEM, *n* = 8–9 mice per group. *p < 0.05 vs vehicle control, **p < 0.05 vs **8** measured using one-way ANOVA and Bonferroni post-test comparison. Error bars in some data points are smaller than the symbols.

To extend the utility of **33** as a dual MER and AXL targeted agent and immunotherapeutic agent, we further evaluated the antitumor efficacy of **33** in four different tumor models and compared the treatment effect of **33** with that of the clinical stage dual MER and AXL inhibitor tamnorzatinib (**8**). **8** showed potent dual inhibitory ability against MER and AXL with IC_50_ values of 9.8 and 2.2 nM, respectively. Both MC38 and Hepa1–6 do not express MER and AXL kinases on the tumors and were used to evaluate the antitumor immunomodulatory activities of **33**. 4T1 expresses AXL enzyme on the tumor and both MER and AXL enzymes on the immune cells and was used to examine the effect of **33** on tumor growth and host antitumor immunity. MDA-MB-231 coexpresses MER and AXL proteins on the tumor and was used to evaluate the effect of **33** on dual MER and AXL inhibition within the tumor. Both compounds were used at 30 mg/kg QD and FOTO regimen in Hepa1–6 tumor model and 50 mg/kg QD and FOTO regimen in MC38, 4T1, and MDA-MB-231 tumor models. Animals were treated at various lengths based on the tumor growth rate of the given tumor model. Figure 6 summarizes the results of these experiments. Treatment with **33** produced TGI values of 49, 44, 86, and 81% in 4T1, MDA-MB-231, MC38, and Hepa1–6, respectively. On the other hand, treatment with **8** produced TGI values of 12, 9, 45, and 17% in these tumor models. During the treatment, none of the mice showed severe adverse effects or massive body weight loss (Figures S4–S7).

To understand the specificity and potential off-target effects of **33**, it was profiled across a panel of 658 human protein kinases (including 288 mutant kinases) at a screening concentration of 1 μM by the radioactive HotSpot kinase assay.^38^ The results revealed that **33** possessed a superior target selectivity with a kinome selectivity *S*(10) score of 0.032 (12/370 nonmutant kinases) (Table S1). In addition to desired target kinases, **33** also strongly inhibits TYRO3, the homology kinase of MER and AXL, and other kinases that are known to be targeted by type II kinase inhibitors, including FLT3, KIT, TRKA/B/C, and so forth. To further understand to safety profile of **33**, we also have carried out a 14-day toxicity examination with repeated dose in ICR mice, and the clinical candidate tamnorzatinib (**8**) was utilized as the positive control (Figures S8–S11). **33** showed no significant toxicity at any dose level, only a mild decline in kidney serum biomarker CRE at 30 and 100 mpk. These results were similar to those observed for **8** at a 100 mpk dosage. To sum up, the *in vivo* data suggested that **33** could function as an excellent immune modulator and an effective anticancer agent simultaneously, which could be a promising candidate for further investigation as a potential therapeutic agent for cancer treatment.

## Chemistry

**Scheme 1:**
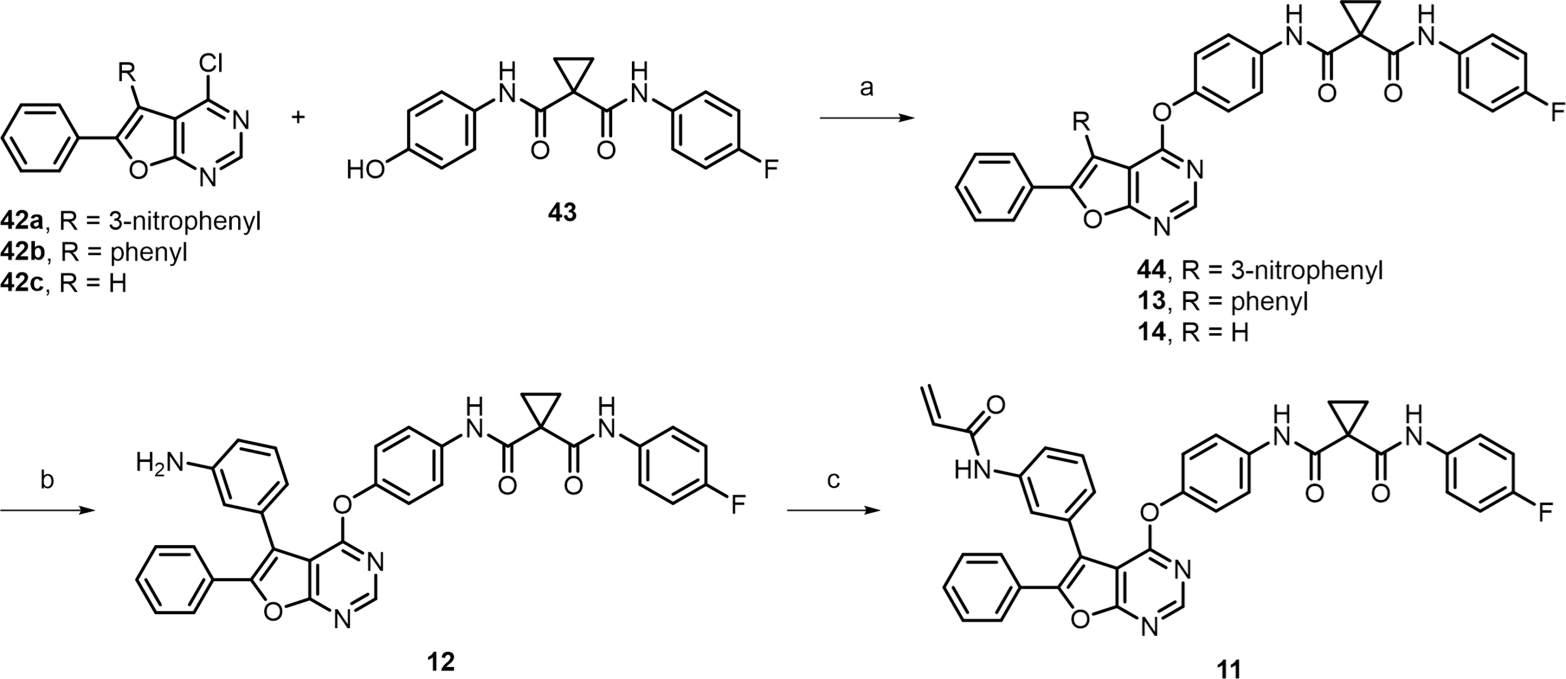
Synthetic Route for MER/AXL Inhibitors **11**–**14** Reagents and conditions: (a) DMF, rt, 16–24 h, 58–99%; (b) iron powder, sat. NH_4_Cl_(aq)_, ethanol, CH_2_Cl_2_, H_2_O, 80 °C, 1.5 h, 65%; (c) acrylic acid, EDCI, CH_2_Cl_2_, rt, 3.5 h, 62%.

Syntheses of compounds **11**–**14** are outlined in Scheme 1. Similar to our prior studies,^32,33^ the 4-chloride in building blocks **42a**–**c** was initially substituted by dicarboxamide **43** through nucleophilic aromatic substitution reaction (S_N_Ar), resulting in the formation of intermediate **44**, as well as inhibitors **13** and **14**. The nitro group of **44** was subsequently reduced to an amino group with iron powder under acidic conditions to generate inhibitor **12**. Finally, amide bond formation was accomplished by treating **12** with acrylic acid, resulting in the synthesis of inhibitor **11** in a moderate yield.

**Scheme 2:**
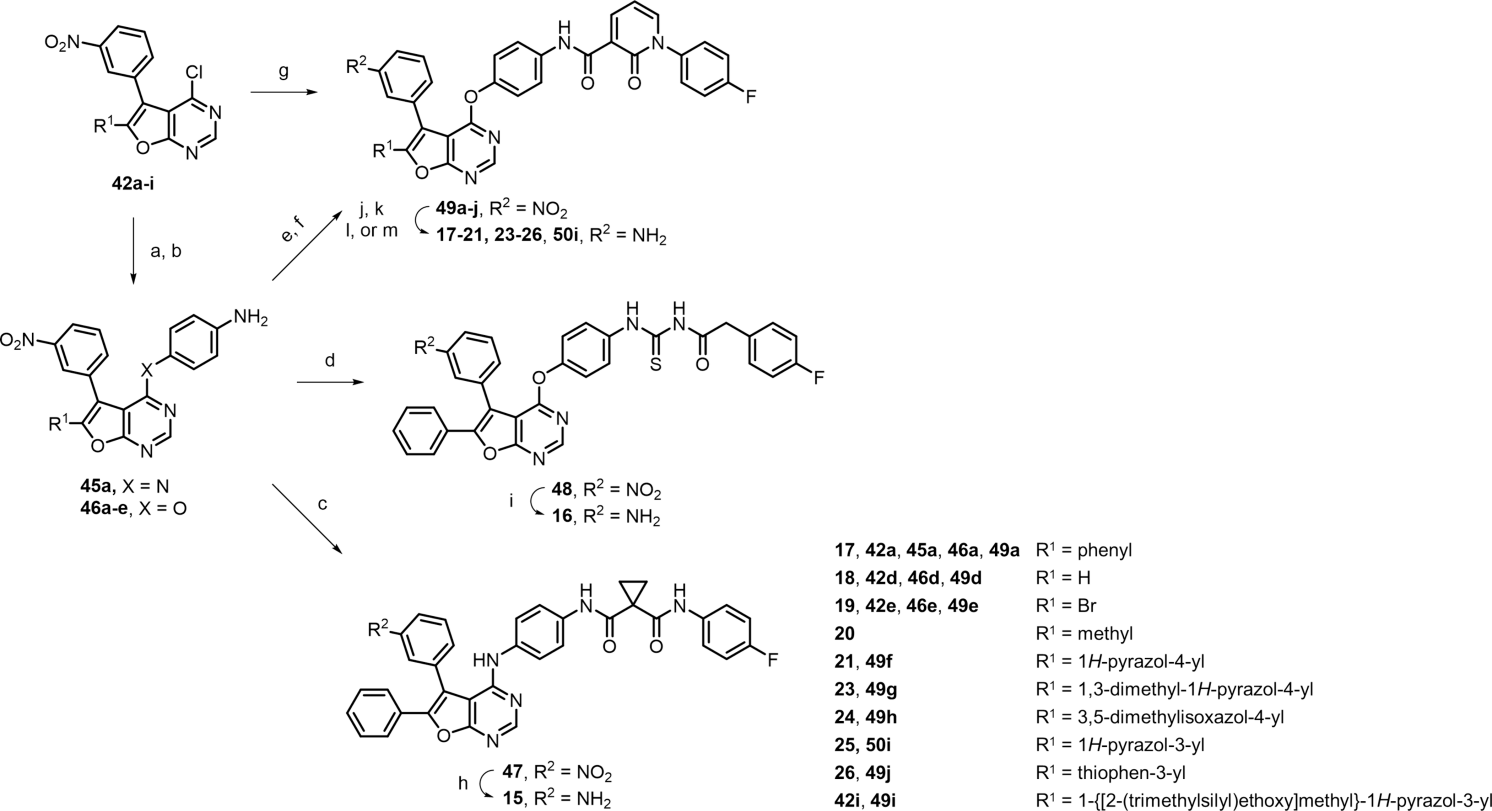
Synthetic Route for MER/AXL Inhibitors **15**–**21** and **23**–**26** Reagents and conditions: (a) benzene-1,4-diamine, ethanol, reflux, 16 h, 89%; (b) 4-aminophenol, NaH or K_2_CO_3_, DMF, rt, 8–16 h, 75–99%; (c) 1-[(4-fluorophenyl)carbamoyl]cyclopropane-1-carboxylic acid, EDCI, DMA, rt, 3 h, 20%; (d) (4-fluorophenyl)acetyl isothiocyanate, toluene, ethanol, rt, 45 min, 68%; (e) 1-(4-fluorophenyl)-2-oxo-1,2-dihydropyridine-3-carboxylic acid, DIPEA, CH_2_Cl_2_, 80 °C, 1 h, sealed tube, 28% for **49a**; TBTU, DIPEA, CH_2_Cl_2_, rt, 12 h, 60% for **49d**; EDCI, DMAP, DMF, rt, 16 h, 55% for **49e**; (f) appropriate boronic acids or boronic esters, Pd(dppf)Cl_2_, Na_2_CO_3_, 1,4-dioxane or DMF/THF, 75–110 °C, 5–16 h, 64–88%; (g) 1-(4-fluorophenyl)-*N*-(4-hydroxyphenyl)-2-oxo-1,2-dihydropyridine-3-carboxamide, NaH, DMF, rt, 2 h, 77%; (h) SnCl_2_·2H_2_O, ethanol, reflux, 4 h, 42%; (i) iron powder, sat. NH_4_Cl_(aq)_, ethanol, CH_2_Cl_2_, H_2_O, 70 °C, 16 h, 67%; (j) iron powder, sat. NH_4_Cl_(aq)_, ethanol, CH_2_Cl_2_, H_2_O, 70–80 °C, 4–16 h, 5–93% for **17**, **19**, **26**, **50i**; (k) SnCl_2_·2H_2_O, ethanol, CH_2_Cl_2_/H_2_O or DMF, rt–75 °C, 1.5–20 h, 20–85% for **18**, **21**, **23**, **24**; (l) from **19**, methylboronic acid, Pd_2_dba_3_, SPhos, K_3_PO_4_, 90 °C, 24 h, 54%; (m) from **50i**, trifluoroacetic acid, CH_2_Cl_2_, rt, 16 h, 39%.

In Scheme 2, we detailed the syntheses of inhibitors **15**–**21** and **23**–**26**. To synthesize **15**, building block **42a** was coupled with benzene-1,4-diamine initially to form intermediate **45a**. The amino group of **45a** was then substituted with a fluorophenyl-containing carboxylic acid to generate intermediate **47**. Subsequently, the nitro-reduction of **47** using stannic chloride generated **15** in a 42% yield. By a similar token, the 4-chloride of scaffolds **42a**, **42d**, and **42e** were first substituted by 4-aminophenol under basic conditions to form corresponding anilines **46a**, **46d**, and **46e**, respectively. Inhibitor **16** was obtained by coupling the amino group of **46a** with (4-fluorophenyl)acetyl isothiocyanate, followed by nitro group reduction on the 5-phenyl ring of **48**. Additionally, **46a** reacted with 1-(4-fluorophenyl)-2-oxo-1,2-dihydropyridine-3-carboxylic acid under basic conditions to yield **49a**, which was then transformed into inhibitor **17** through nitro-reduction with iron powder and saturated ammonium chloride. Following the same campaign, **46d** and **46e** were converted into **49d** and **49e**, and then, both compounds were subsequently reduced to yield inhibitors **18** and **19**, respectively. Notably, **19** and **49e**, both featuring a 6-bromo functional group, served as valuable intermediates for further synthesis. Inhibitor **20** was generated from **19***via* Suzuki coupling reaction using methylboronic acid in a moderate yield. In a similarity, **49e** was coupled with different boronic esters to produce **49f**–**h** or coupled with thiophen-3-ylboronic acid to receive **49j**. The same as the abovementioned reductions, **49f**–**j** were converted into inhibitors **21**, **23**, **24**, and **26** with stannic chloride or iron powder under acid conditions. For **25**, the 6-bromo group of **42e** was first coupled with SEM-protected 1*H*-pyrazol-3-yl boronic ester, forming building block **42i**. Through the S_N_Ar reaction, **42i** was treated with the presynthesized carboxamide to generate **49i**. Subsequently, an iron-catalyzed reduction was performed, and the protecting group of **50i** was removed using trifluoroacetic acid to receive the desired inhibitor **25**.

**Scheme 3:**
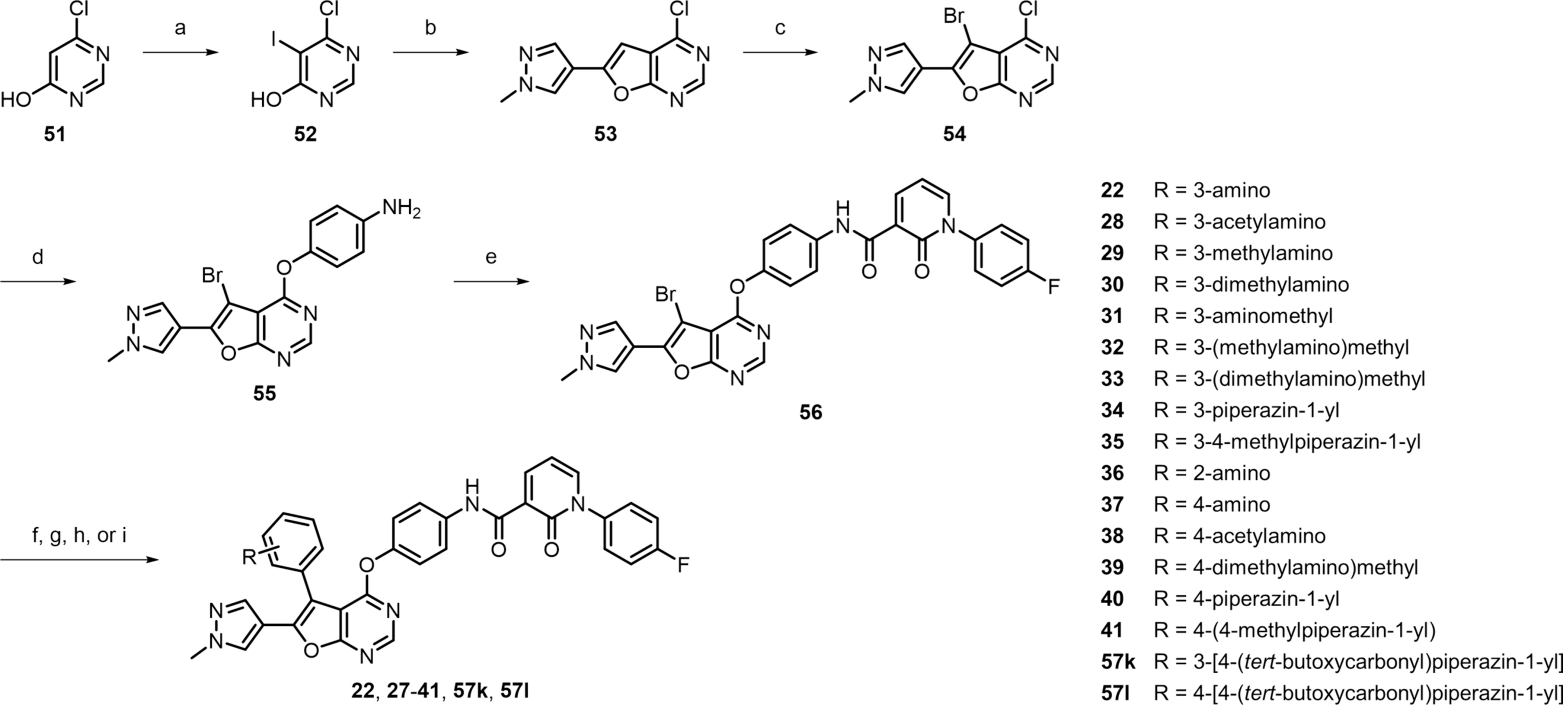
Synthetic Route for MER/AXL Inhibitors **22** and **27**–**41** Reagents and conditions: (a) *N*-iodosuccinimide, trifluoroacetic acid, CH_2_Cl_2_, rt, 12 h, 86%; (b) 4-ethynyl-1-methyl-1*H*-pyrazole, Pd(PPh_3_)_4_, CuI, Et_3_N, 70 °C, 12 h, 30%; (c) *N*-bromosuccinimide, DMF, rt, 2 h, 72%; (d) 4-aminophenol, NaH, DMF, THF, rt, 12 h, 92%; (e) 1-(4-fluorophenyl)-2-oxo-1,2-dihydropyridine-3-carboxylic acid, TBTU, DIPEA, DMF, rt, 10 h, quant.; (f) appropriate boronic acids or boronic esters, Pd(dppf)Cl_2_, Na_2_CO_3_, DMF or DMF/THF, 80–120 °C, 5–16 h, 17–82%; (g) from **22**, acetyl chloride, Et_3_N, CH_2_Cl_2_, rt, 16 h, 64%; (h) from **57k** or **57l**, trifluoroacetic acid, CH_2_Cl_2_, rt, 16 h, 71–94%; (i) from **34**, formaldehyde, NaBH(OAc)_3_, methanol, CH_2_Cl_2_, rt, 1.5 h, 38%.

Inhibitors **22**, and **27**–**41** were obtained following the procedure presented in Scheme 3. The process commenced with the iodination of commercially available 6-chloropyrimidin-4-ol **51** using *N*-iodosuccinimide under acidic conditions, generating **52** in an 86% yield. The 5-iodide of **52** was then subjected to Sonogashira coupling reaction with 4-ethynyl-1-methyl-1*H*-pyrazole, facilitated by a tetrakis(triphenylphosphine)palladium catalyst. This was subsequently followed by a ring closure reaction to obtain the pyrazolyl-containing furanopyrimidine scaffold **53**. To further functionalize the scaffold, the bromination of **53** was taken place using *N*-bromosuccinimide to receive scaffold **54** in a good yield. Building upon the approach outlined in Scheme 2, the 4-chloride of **54** was reacted with 4-aminophenol under basic conditions to offer aniline **55**. This aniline was subsequently coupled with 1-(4-fluorophenyl)-2-oxo-1,2-dihydropyridine-3-carboxylic acid to provide **56** in excellent yields. Suzuki coupling of **56** with various boronic acids or boronic esters employing Pd(dppf)Cl_2_ as the catalyst. This approach produced inhibitors **22**, **27**, **29**–**33**, **36**–**39**, and **41**, as well as intermediates **57k** and **57l**. Additionally, the amino group on the 5-phenyl ring of **22** was reacted with acetyl chloride under basic conditions to obtain inhibitor **28** in a moderate yield. The intermediates **57k** and **57l**, both containing Boc protecting groups, were transformed into inhibitors **34** and **40**, respectively, by treatment with trifluoroacetic acid. For inhibitor **35**, reductive amination of **34** was performed using formaldehyde with NaBH(OAc)_3_ as the reducing agent, resulting in desired inhibitor **35** with a moderate yield.

## Conclusions

Kinase-targeted cancer therapies have been extensively studied for decades, while cancer immunotherapy has witnessed remarkable advancements in recent years. Both approaches have shown potential to significantly enhance patient outcomes, and ongoing research continues to explore combining them for even more effective and personalized cancer treatments. In this study, we discovered a series of promising MER/AXL inhibitors possessed a furanopyrimidine scaffold that exhibited excellent immunomodulatory effects and effective antitumor efficacy in murine syngeneic and xenograft tumor models.

Initially, leveraging the advantages of our EGFR clinical candidate and known privileged pharmacophores, we designed and synthesized 34 MER/AXL kinase inhibitors in order to increase enzymatic activities against both MER and AXL as well as improve drug-like properties. Through SAR studies, we revealed that the pharmacophore side chain (**17** > **15** ≫ **16**) hybridized with a furanopyrimidine scaffold, leading to **17** as an initial lead. Optimal combinations, including an amino and *N*-(dimethylamino)methyl substituent at the meta-position on top aryl region and 1*H*-pyrazol-4-yl substituent on the bottom of the aryl region, produced dual inhibitors **22** and **33**, exhibiting excellent biological inhibitory activities with acceptable PK profiles.

More importantly, **22** and **33** not only demonstrated remarkable *in vitro* inhibitory potency at the single-digit nanomolar level but also exhibited excellent immune-modulating activity by enhancing the expression of total T-cells, cytotoxic CD8^+^ T-cells, and helper CD4^+^ T-cells in the spleen. Additionally, **33** also remarkably exhibited anticancer activity, achieving a TGI value of nearly 90% at 50 mg/kg BID orally in the MC38 syngeneic murine colorectal model. By the same token, **33** suppressed tumor growth significantly in both the 4T1 syngeneic and MDA-MB-231 xenograft triple-negative breast cancer models, with TGI values exceeding 50%. Moreover, **33** significantly delayed tumor growth in the Hepa1–6 liver cancer model, showing an extraordinary TGI value of 81% with a 30 mg/kg dose regimen. In summary, the immunomodulatory property and antitumor capability of **33** (**BPR5K230**) highlight its potential as a promising targeted therapy for cancer. Further preclinical studies are ongoing and will be revealed in due course.

## Experimental Section

### General Methods for Chemistry

All commercial chemicals and solvents are of reagent grade and were used without further purification unless otherwise stated. All reactions were carried out under a dry nitrogen or argon atmosphere and were monitored for completion by TLC using Merck 60 F254 silica gel glass-backed plates or aluminum plates; zones were detected visually under UV irradiation (254 nm) or by spraying with potassium permanganate reagent (Aldrich) followed by heating at 80 °C. Flash column chromatography was carried out using silica gel (Silicycle SiliaFlash P60, R12030B, 230–400 mesh or Merck grade 9385, 230–400 mesh). ^1^H and ^13^C NMR spectra were recorded with Bruker 400 or 600 MHz AVANCE III spectrometers. Data analysis was done using Mnova software (Mestrelab Research). Chemical shift (δ) was reported in ppm and referenced to solvent residual signals as follows: DMSO-*d*_6_ at 2.50 ppm, CDCl_3_ at 7.26 ppm for ^1^H NMR; DMSO-*d*_6_ at 39.5 ppm, CDCl_3_ at 77.0 ppm for ^13^C NMR. Splitting patterns are indicated as follows: s = singlet; d = doublet; q = quartet; dd = doublet of doublets; ddd = doublet of doublets of doublets; m = multiplet. Coupling constants (*J*) were given in Hertz (Hz). Low-resolution mass spectra (LRMS) data were measured with an Agilent MSD-1100 ESI-MS/MS system or Agilent Infinity II 1290 LC/MS (ESI) systems. High-resolution mass spectra (HRMS) data were measured with a Varian 901-MS FT-ICR HPLC/MS-MS system. The purity of the final compounds was determined using high-performance liquid chromatography (HPLC) system (Hitachi 2000 series) equipped with a C18 column (Agilent ZORBAX Eclipse XDB-C18 5 μm. 4.6 mm × 150 mm) and operating at 25 °C, or ultra-performance liquid chromatography (UPLC) system (Waters Acquity UPLC/BSM) equipped with a C18 column (Waters Acquity BEH-C18 1.7 μm. 2.1 mm × 50 mm) and operating at 25 °C. For the HPLC system, elution was carried out using acetonitrile as mobile phase A, and water containing 0.1% formic acid and 2 mmol NH_4_OAc as mobile phase B. Elution conditions: at 0.0 min, phase A 10% + phase B 90% with 0.5 mL/min flow rate of the mobile phase; at 25.0 min, phase A 90% + phase B 10% with 0.5 mL/min flow rate of the mobile phase; at 30.5 min, phase A 10% + phase B 90% with 1.0 mL/min flow rate of the mobile phase; at 34.5 min, phase A 10% + phase B 90% with 0.5 mL/min flow rate of the mobile phase; at 37.0 min, phase A 10% + phase B 90% with 0.5 mL/min flow rate of the mobile phase. The injection volume of the sample was 20 μL. For the UPLC system, elution was carried out using acetonitrile as mobile phase A and water containing 0.1% formic acid and 2 mmol NH_4_OAc as mobile phase B. Elution conditions: at 0.00 min, phase A 10% + phase B 90%; at 4.15 min, phase A 90% + phase B 10%; at 5.00 min, phase A 10% + phase B 90%; at 6.50 min, phase A 10% + phase B 90%. The flow rate of the mobile phase was 0.6 mL/min, and the injection volume of the sample was 5 μL. Peaks were detected at 254 nm. The purity of all tested compounds was determined and confirmed to be greater than 95% by HPLC or UPLC analysis except for compounds **13** (94.1%), **16** (80.2%), **17** (86.9%), **21** (91.4%), **22** (94.1%), **25** (87.7%), **26** (91.8%), **29** (93.5%), **30** (94.2%), **31** (92.5%), **32** (93.3%), **40** (94.6%), and **42** (91.9%). IUPAC nomenclature of compounds was obtained with Mnova software (Mestrelab Research).

#### *N*^1^-[4-({5-[3-(Acryloylamino)phenyl]-6-phenylfuro[2,3-*d*]pyrimidin-4-yl}oxy)phenyl]-*N*^1^-(4-fluorophenyl)cyclopropane-1,1-dicarboxamide (**11**)

To a solution of **12** (89 mg, 0.15 mmol, 1.0 equiv) in dichloromethane (1 mL) were added acrylic acid (15 μL, 0.22 mmol, 1.5 equiv) and EDCI (43 mg, 0.22 mmol, 1.5 equiv), and then, the reaction mixture was stirred at room temperature. After stirring for 3.5 h, the reaction mixture was concentrated *in vacuo* and purified by flash column chromatography (50% ethyl acetate in hexane) to yield the title compound **11** (60 mg, 0.09 mmol, 62%) as a white solid. ^1^H NMR (600 MHz, DMSO-*d*_6_) δ 10.25 (s, 1H), 10.11 (s, 1H), 10.06 (s, 1H), 8.54 (s, 1H), 7.96 (dd, *J* = 2.1, 1.8 Hz, 1H), 7.74 (ddd, *J* = 8.4, 2.1, 1.2 Hz, 1H), 7.65–7.61 (m, 4H), 7.60–7.56 (m, 2H), 7.46–7.40 (m, 4H), 7.33 (ddd, *J* = 7.8, 1.8, 1.2 Hz, 1H), 7.17–7.11 (m, 4H), 6.41 (dd, *J* = 16.8, 10.2 Hz, 1H), 6.24 (dd, *J* = 16.8, 2.1 Hz, 1H), 5.74 (dd, *J* = 10.2, 2.1 Hz, 1H), 1.45 (s, 4H). ^13^C NMR (151 MHz, DMSO-*d*_6_) δ 168.13, 168.09, 166.82, 163.29, 158.25 (d, *J*_C–F_ = 240.2 Hz), 152.93, 148.67, 147.69, 139.31, 136.38, 135.20, 131.73, 131.36, 129.61, 129.17, 128.97, 128.50, 127.13, 126.91, 125.30, 122.36 (d, *J*_C–F_ = 8.0 Hz), 121.77, 121.49, 120.62, 119.15, 115.03 (d, *J*_C–F_ = 22.2 Hz), 114.92, 106.41, 31.45, 15.43. LRMS (ESI) *m*/*z* 654.2 [M + H]^+^. HRMS (ESI) *m*/*z* for C_38_H_28_FN_5_NaO_5_ [M + Na]^+^, calcd 676.1972, found 676.1972. HPLC purity 98.02% (*t*_R_ = 27.83 min).

#### *N*^1^-(4-{[5-(3-Aminophenyl)-6-phenylfuro[2,3-*d*]pyrimidin-4-yl]oxy}phenyl)-*N*^1^-(4-fluorophenyl)cyclopropane-1,1-dicarboxamide (**12**)

To a solution of **44** (209 mg, 0.33 mmol, 1.0 equiv) in ethanol (6.6 mL), dichloromethane (6.6 mL) and water (1.3 mL) were added iron powder (56 mg, 1.00 mmol, 3.0 equiv) and sat. NH_4_Cl_(aq)_ (0.7 mL), and then, the reaction mixture was stirred at 80 °C. After stirring for 4 h, the reaction mixture was cooled down to room temperature, filtered through Celite, washed with methanol (10 mL) and dichloromethane (10 mL), concentrated *in vacuo*, and purified by flash chromatography (50% ethyl acetate in hexane) to yield the title compound **12** (130 mg, 0.22 mmol, 65%) as a white solid. ^1^H NMR (600 MHz, DMSO-*d*_6_) δ 10.12 (s, 1H), 10.08 (s, 1H), 8.51 (s, 1H), 7.67–7.57 (m, 6H), 7.45–7.38 (m, 3H), 7.17–7.07 (m, 5H), 6.76 (dd, *J* = 1.2, 1.2 Hz, 1H), 6.71 (dd, *J* = 9.0, 2.7 Hz, 1H), 6.60 (ddd, *J* = 8.4, 2.7, 1.2 Hz, 1H), 5.19 (s, 2H), 1.46 (s, 4H). ^13^C NMR (151 MHz, DMSO-*d*_6_) δ 168.14, 168.10, 166.74, 163.32, 158.25 (d, *J*_C–F_ = 240.1 Hz), 152.75, 148.84, 148.25, 147.82, 136.34, 135.20, 131.28, 129.34, 129.14, 128.82, 126.77, 122.37 (d, *J*_C–F_ = 7.9 Hz), 121.83, 121.51, 117.27, 115.87, 115.16, 115.03 (d, *J*_C–F_ = 22.2 Hz), 113.93, 106.54, 31.46, 15.44. LRMS (ESI) *m*/*z* 600.2 [M + H]^+^. HRMS (ESI) *m*/*z* for C_35_H_27_FN_5_O_4_ [M + H]^+^, calcd 600.2047, found 600.2051. HPLC purity 94.06% (*t*_R_ = 28.21 min).

#### *N*^1^-{4-[(5,6-Diphenylfuro[2,3-*d*]pyrimidin-4-yl)oxy]phenyl}-*N*^1^-(4-fluorophenyl)cyclopropane-1,1-dicarboxamide (**13**)

To a solution of sodium hydride (15 mg, 0.38 mmol, 1.5 equiv) in DMF (1 mL) at 0 °C was added a solution of *N*^1^-(4-fluorophenyl)-*N*^1^-(4-hydroxyphenyl)cyclopropane-1,1-dicarboxamide (**43**) (79 mg, 0.25 mmol, 1.0 equiv) in DMF (1 mL), and then, the reaction mixture was stirred at room temperature. After stirring for 20 min, the reaction mixture was cooled down to 0 °C, and 4-chloro-5,6-diphenylfuro[2,3-*d*]pyrimidine (**42b**) (77 mg, 0.25 mmol, 1.0 equiv) was added and then stirred at room temperature. After stirring for 16 h, the reaction mixture was filtered through Celite, concentrated *in vacuo*, and purified by thin-plate chromatography (2% methanol in dichloromethane) to yield the title compound **13** (85 mg, 0.15 mmol, 58%) as a white solid. ^1^H NMR (600 MHz, DMSO-*d*_6_) δ 10.12 (s, 1H), 10.08 (s, 1H), 8.51 (s, 1H), 7.67–7.57 (m, 6H), 7.45–7.38 (m, 3H), 7.17–7.07 (m, 5H), 6.76 (dd, *J* = 1.2, 1.2 Hz, 1H), 6.71 (dd, *J* = 9.0, 2.7 Hz, 1H), 6.60 (ddd, *J* = 8.4, 2.7, 1.2 Hz, 1H), 5.19 (s, 2H), 1.46 (s, 4H). ^13^C NMR (151 MHz, DMSO-*d*_6_) δ 168.13, 168.08, 166.83, 163.4, 158.24 (d, *J*_C–F_ = 240.2 Hz), 152.88, 148.71, 147.75 136.38, 135.20, 130.77, 130.16, 129.55, 128.90, 128.59, 128.42, 126.97, 122.35 (d, *J*_C–F_ = 7.7 Hz), 121.74, 121.55, 115.02 (d, *J*_C–F_ = 22.8 Hz), 106.43, 31.44, 15.45. LRMS (ESI) *m*/*z* 585.2 [M + H]^+^. HRMS (ESI) *m*/*z* for C_35_H_25_FN_4_NaO_4_ [M + Na]^+^, calcd 607.1758, found 607.1758. HPLC purity 98.24% (*t*_R_ = 31.08 min).

#### *N*^1^-(4-Fluorophenyl)-*N*^1^-{4-[(6-phenylfuro[2,3-*d*]pyrimidin-4-yl)oxy]phenyl}cyclopropane-1,1-dicarboxamide (**14**)

To a solution of sodium hydride (15 mg, 0.38 mmol, 1.5 equiv) in DMF (1 mL) at 0 °C was added a solution of *N*^1^-(4-fluorophenyl)-*N*^1^-(4-hydroxyphenyl)cyclopropane-1,1-dicarboxamide (**43**) (79 mg, 0.25 mmol, 1.0 equiv) in DMF (1 mL), and then, the reaction mixture was stirred at room temperature. After stirring for 20 min, the reaction mixture was cooled down to 0 °C, 4-chloro-6-phenylfuro[2,3-*d*]pyrimidine (**42c**) (58 mg, 0.25 mmol, 1.0 equiv) and then stirred at room temperature. After stirring for 16 h, the reaction mixture was filtered through Celite, concentrated *in vacuo*, and purified by thin-plate chromatography (2% methanol in dichloromethane) to yield the title compound **14** (76 mg, 0.15 mmol, 59%) as a white solid. ^1^H NMR (600 MHz, DMSO-*d*_6_) δ 10.19 (s, 1H), 10.09 (s, 1H), 8.50 (s, 1H), 7.98 (d, *J* = 7.2 Hz, 2H), 7.73 (d, *J* = 9.0 Hz, 2H), 7.67–7.63 (m, 3H), 7.54 (dd, *J* = 7.8, 7.2 Hz, 2H), 7.49–7.45 (m, 1H), 7.27 (d, *J* = 9.0 Hz, 2H), 7.15 (dd, *J* = 9.0, 9.0 Hz, 2H), 1.48 (s, 4H). ^13^C NMR (151 MHz, DMSO-*d*_6_) δ 168.13, 167.98, 162.88, 158.27 (d, *J*_C–F_ = 240.2 Hz), 153.92, 152.58, 147.78, 136.60, 135.21 (d, *J*_C–F_ = 2.6 Hz), 129.77, 129.21, 128.41, 124.97, 122.39 (d, *J*_C–F_ = 8.0 Hz), 121.83, 121.71, 115.03 (d, *J*_C–F_ = 22.2 Hz), 106.44, 98.06, 31.50, 15.46. LRMS (ESI) *m*/*z* 509.1 [M + H]^+^. HRMS (ESI) *m*/*z* for C_29_H_21_FN_4_NaO_4_ [M + Na]^+^, calcd 531.1445, found 531.1443. HPLC purity 98.25% (*t*_R_ = 29.04 min).

#### *N*^1^-(4-{[5-(3-Aminophenyl)-6-phenylfuro[2,3-*d*]pyrimidin-4-yl]amino}phenyl)-*N*^1^-(4-fluorophenyl)cyclopropane-1,1-dicarboxamide (**15**)

To a solution of **47** (83 mg, 0.13 mmol, 1.0 equiv) in ethanol (5.0 mL) was added SnCl_2_·2H_2_O (118 mg, 0.52 mmol, 4.0 equiv), and then, the reaction mixture was stirred at reflux. After stirring for 4 h, the reaction mixture was cooled down to room temperature and concentrated *in vacuo*. Then, the mixture was dissolved in ethyl acetate (10 mL) and washed with sat. NaHCO_3(aq)_ (10 mL × 3) and brine (10 mL). The combined organic layers were dried over MgSO_4_, concentrated *in vacuo* and purified by thin-plate chromatography (50% ethyl acetate in hexane) to yield the title compound **15** (33 mg, 0.06 mmol, 42%) as a yellow solid. ^1^H NMR (600 MHz, DMSO-*d*_6_) δ 10.06 (s, 1H), 10.04 (s, 1H), 8.50 (s, 1H), 7.64–7.60 (m, 4H), 7.56 (d, *J* = 9.0 Hz, 2H), 7.41 (dd, *J* = 8.4, 7.2 Hz, 2H), 7.37–7.31 (m, 4H), 7.14 (dd, *J* = 9.0, 8.4 Hz, 2H), 7.04 (s, 1H), 6.81 (ddd, *J* = 8.4, 2.7, 0.9 Hz, 1H), 6.79 (ddd, *J* = 2.7, 2.4 Hz, 1H), 6.76 (ddd, *J* = 7.2, 2.4, 0.9 Hz, 1H), 5.54 (s, 2H), 1.45 (s, 4H). ^13^C NMR (151 MHz, DMSO-*d*_6_) δ 168.30, 167.97, 164.49, 158.26 (d, *J*_C–F_ = 238.5 Hz), 154.38, 153.67, 150.21, 146.01, 135.15, 134.38, 134.01, 131.93, 130.80, 128.96, 128.91, 125.91, 122.38 (d, *J*_C–F_ = 7.8 Hz), 121.12, 119.63, 115.98, 115.54, 115.04 (d, *J*_C–F_ = 21.9 Hz), 114.68, 113.88, 103.68, 31.28, 15.48. LRMS (ESI) *m*/*z* 599.2 [M + H]^+^. HRMS (ESI) *m*/*z* calcd for C_35_H_26_FN_6_O_3_ [M – H]^+^, calcd 597.2050, found 597.2050. HPLC purity 80.18% (*t*_R_ = 28.72 min).

#### (4-{[5-(3-Aminophenyl)-6-phenylfuro[5,4-*d*]pyrimidin-4-yl]oxy}phenyl)-*N*-(phenylacetyl)carbamothioic amide (**16**)

To a solution of **48** (166 mg, 0.27 mmol, 1.0 equiv) in ethanol (6.5 mL), dichloromethane (6.5 mL), and water (1.3 mL) were added iron powder (94 mg, 1.68 mmol, 6.3 equiv) and sat. NH_4_Cl_(aq)_ (0.7 mL), and then, the reaction mixture was stirred at 80 °C. After stirring for 4 h, the reaction mixture was cooled down to room temperature, filtered through Celite, washed with methanol (10 mL) and dichloromethane (10 mL), and concentrated *in vacuo* to yield the title compound **16** (142 mg, 0.25 mmol, 93%) as a white solid. ^1^H NMR (600 MHz, DMSO-*d*_6_) δ 12.35 (s, 1H), 11.73 (s, 1H), 8.54 (s, 1H), 7.65–7.59 (m, 4H), 7.45–7.39 (m, 3H), 7.37–7.33 (m, 4H), 7.31–7.26 (m, 1H), 7.24 (d, *J* = 9.0 Hz, 2H), 7.10 (dd, *J* = 8.1, 7.5 Hz, 1H), 6.76 (dd, *J* = 2.1, 1.5 Hz, 1H), 6.71 (ddd, *J* = 7.5, 1.5, 1.2 Hz, 1H), 6.59 (ddd, *J* = 8.1, 2.1, 1.2 Hz, 1H), 5.18 (s, 2H), 3.82 (s, 2H). ^13^C NMR (151 MHz, DMSO-*d*_6_) δ 179.17, 173.14, 166.79, 162.98, 152.74, 149.94, 148.82, 148.33, 135.09, 134.26, 131.19, 129.53, 129.36, 129.14, 128.82, 128.77, 128.43, 126.99, 126.78, 125.76, 121.94, 117.26, 115.85, 115.16, 113.96, 106.67, 42.36. LRMS (ESI) *m*/*z* 572.3 [M + H]^+^. HRMS (ESI) *m*/*z* for C_33_H_25_N_5_NaO_3_S [M + Na]^+^, calcd 594.1576, found 594.1576. HPLC purity 86.91% (*t*_R_ = 28.92 min).

#### *N*-(4-{[5-(3-Aminophenyl)-6-phenylfuro[5,4-*d*]pyrimidin-4-yl]oxy}phenyl)-1-(4-fluorophenyl)-2-oxo-1,2-dihydropyridine-3-carboxamide (**17**)

To a solution of **49a** (310 mg, 0.48 mmol, 1.0 equiv) in ethanol (17.8 mL), dichloromethane (17.8 mL), and water (3.3 mL) were added iron powder (165 mg, 2.95 mmol, 6.1 equiv) and sat. NH_4_Cl_(aq)_ (1.2 mL), and then, the reaction mixture was stirred at 80 °C. After stirring for 4 h, the reaction mixture was cooled down to room temperature, filtered through Celite, washed with methanol (10 mL) and dichloromethane (10 mL), concentrated *in vacuo*, and purified by flash chromatography (3% methanol in dichloromethane) to yield the title compound **17** (17 mg, 0.03 mmol, 5%) as a white solid. ^1^H NMR (600 MHz, DMSO-*d*_6_) δ 11.97 (s, 1H), 8.58 (dd, *J* = 7.2, 2.4 Hz, 1H), 8.52 (s, 1H), 8.11 (dd, *J* = 6.6, 1.8 Hz, 1H), 7.74 (d, *J* = 9.0 Hz, 2H), 7.64–7.58 (m, 4H), 7.45–7.39 (m, 5H), 7.20 (d, *J* = 9.0 Hz, 2H), 7.11 (dd, *J* = 7.8, 7.8 Hz, 1H), 6.76 (dd, *J* = 2.4, 1.8 Hz, 1H), 6.74–6.69 (m, 2H), 6.60 (ddd, *J* = 8.4, 2.4, 1.2 Hz, 1H), 5.19 (s, 2H). ^13^C NMR (151 MHz, DMSO-*d*_6_) δ 166.75, 163.22, 161.85, 161.20, 152.78, 148.84, 148.26,147.96, 144.78, 143.94, 136.33, 135.76, 131.263, 129.35 (d, *J*_C–F_ = 8.6 Hz), 129.33, 129.15, 128.83, 126.77, 122.35, 120.74, 120.43, 117.26, 116.16, 116.00, 115.89, 115.16, 113.93, 106.97, 106.58. LRMS (ESI) *m*/*z* 610.5 [M + H]^+^. HRMS (ESI) *m*/*z* for C_36_H_24_FN_5_NaO_4_ [M + Na]^+^, calcd 632.1710, found 632.1717. HPLC purity 97.46% (*t*_R_ = 28.27 min).

#### *N*-(4-{[5-(3-Aminophenyl)furo[2,3-*d*]pyrimidin-4-yl]oxy}phenyl)-1-(4-fluorophenyl)-2-oxo-1,2-dihydropyridine-3-carboxamide (**18**)

To a solution of **49d** (150 mg, 0.27 mmol, 1.0 equiv) in ethanol (5.3 mL) was added SnCl_2_·2H_2_O (480 mg, 2.13 mmol, 8.0 equiv), and then, the reaction mixture was stirred at 70 °C. After stirring for 1.5 h, the reaction mixture was cooled down to room temperature and concentrated *in vacuo*. Then, the mixture was dissolved in ethyl acetate (10 mL) and washed with NaHCO_3(aq)_ (10 mL) and brine (10 mL). The combined organic layers were dried over MgSO_4_, concentrated *in vacuo*, and purified by flash chromatography (1–2% methanol in dichloromethane) to yield the title compound **18** (121 mg, 0.23 mmol, 85%) as a white solid. ^1^H NMR (600 MHz, DMSO-*d*_6_) δ 11.99 (s, 1H), 8.59 (dd, *J* = 7.8, 2.4 Hz, 1H), 8.52 (s, 1H), 8.31 (s, 1H), 8.10 (dd, *J* = 6.9, 2.4 Hz, 1H), 7.77 (d, *J* = 9.0 Hz, 2H), 7.61 (dd, *J* = 9.0, 8.4 Hz, 2H), 7.31 (d, *J* = 9.0 Hz, 2H), 7.09 (dd, *J* = 7.8, 7.8 Hz, 1H), 6.97 (dd, *J* = 2.1, 1.8 Hz, 1H), 7.93 (ddd, *J* = 7.8, 2.1, 1.8 Hz, 1H), 6.72 (dd, *J* = 7.8, 6.9 Hz, 1H), 6.58 (ddd, *J* = 7.8, 2.1, 2.1 Hz, 1H), 5.19 (s, 2H). ^13^C NMR (151 MHz, DMSO-*d*_6_) δ 168.53, 163.48, 161.88 (d, *J*_C–F_ = 244.4 Hz), 161.85, 161.23, 152.78, 148.78, 147.90, 144.79, 143.93, 140.90, 136.32, 135.85, 130.33, 129.35 (d, *J*_C–F_ = 9.0 Hz), 129.02, 122.50, 121.24, 120.82, 120.44, 116.19, 116.09 (d, *J*_C–F_ = 22.8 Hz), 113.94, 113.65, 106.97, 103.28. LRMS (ESI) *m*/*z* 534.2 [M + H]^+^. HRMS (ESI) *m*/*z* for C_30_H_20_FN_5_NaO_4_ [M + Na]^+^, calcd 556.1397, found 556.1393. HPLC purity 95.03% (*t*_R_ = 23.03 min).

#### *N*-(4-{[5-(3-Aminophenyl)-6-bromofuro[2,3-*d*]pyrimidin-4-yl]oxy}phenyl)-1-(4-fluorophenyl)-2-oxo-1,2-dihydropyridine-3-carboxamide (**19**)

To a solution of **49e** (200 mg, 0.31 mmol, 1.0 equiv) in ethanol (6.2 mL), dichloromethane (6.2 mL) and water (1.2 mL) were added iron powder (104 mg, 1.86 mmol, 6.0 equiv) and sat. NH_4_Cl_(aq)_ (0.6 mL), and then, the reaction mixture was stirred at 80 °C. After stirring for 6 h, the reaction mixture was cooled down to room temperature and concentrated *in vacuo*. Then, the mixture was dissolved in dichloromethane (10 mL) and washed with water (10 mL × 3). The combined organic layers were dried over MgSO_4_, concentrated *in vacuo*, and purified by flash chromatography (1% methanol in dichloromethane) to yield the title compound **19** (178 mg, 0.29 mmol, 93%) as a yellow solid. ^1^H NMR (600 MHz, DMSO-*d*_6_) δ 11.97 (s, 1H), 8.58 (dd, *J* = 7.8, 2,4 Hz, 1H), 8.52 (s, 1H), 8.10 (dd, *J* = 7.2, 2.4 Hz, 1H), 7.75 (d, *J* = 9.0 Hz, 2H), 7.61 (dd, *J* = 9.0, 4.8 Hz, 2H), 7.41 (dd, *J* = 9.0, 8.4 Hz, 2H), 7.25 (d, *J* = 9.0 Hz, 2H), 7.11 (dd, *J* = 7.8, 7.2 Hz, 1H), 6.88 (d, *J* = 2.4, 1.5 Hz, 1H), 6.82 (d, *J* = 7.5 Hz, 1H), 6.71 (dd, *J* = 7.5, 7.5 Hz, 1H), 6.60 (ddd, *J* = 7.5, 1.5, 0.6 Hz, 1H), 5.26 (s, 2H). ^13^C NMR (151 MHz, DMSO-*d*_6_) δ 167.67, 162.15, 161.87 (d, *J*_C–F_ = 246.1 Hz), 161.84, 161.21, 152.80, 148.56, 147.74, 144.77, 143.92, 136.30, 135.90, 129.34 (d, *J*_C–F_ = 9.1 Hz), 129.28, 128.72, 125.80, 122.38, 120.77, 120.41, 119.74, 117.16, 116.07 (d, *J*_C–F_ = 23.0 Hz), 115.07, 113.94, 106.96, 105.10. LRMS (ESI) *m*/*z* 612.1 [M + H]^+^. HRMS (ESI) *m*/*z* for C_30_H_20_BrFN_5_O_4_ [M + H]^+^, calcd 612.0683, found 612.0683. HPLC purity 95.64% (*t*_R_ = 25.70 min).

#### *N*-(4-{[5-(3-Aminophenyl)-6-methylfuro[2,3-*d*]pyrimidin-4-yl]oxy}phenyl)-1-(4-fluorophenyl)-2-oxo-1,2-dihydropyridine-3-carboxamide (**20**)

To a solution of **19** (89 mg, 0.15 mmol, 1.0 equiv) in toluene (7.0 mL) were added methylboronic acid (35 mg, 0.58 mmol, 4.0 equiv), Pd_2_dba_3_ (5.32 mg, 0.01 mmol, 4 mol %), SPhos (9.54 mg, 0.02 mmol, 16 mol %), and K_3_PO_4_ (93 mg, 0.44 mmol, 3.0 equiv), and then, the reaction mixture was stirred at 90 °C. After stirring for 24 h, the reaction mixture was cooled down to room temperature, added with water, and extracted into dichloromethane (10 mL × 3). The combined organic layers were washed with brine, dried over Na_2_SO_4_, concentrated *in vacuo*, and purified by flash chromatography (0.5–1% methanol in dichloromethane) to yield the title compound **20** (43 mg, 0.08 mmol, 54%) as a white solid. ^1^H NMR (600 MHz, DMSO-*d*_6_) δ 11.97 (s, 1H), 8.58 (dd, *J* = 7.2, 2.1 Hz, 1H), 8.44 (s, 1H), 8.10 (dd, *J* = 6.9, 2.1 Hz, 1H), 7.74 (d, *J* = 9.0 Hz, 2H), 7.61 (dd, *J* = 9.0, 4.8 Hz, 2H), 7.42 (dd, *J* = 9.0, 8.4 Hz, 2H), 7.24 (d, *J* = 9.0 Hz, 2H), 7.09 (dd, *J* = 7.8, 7.8 Hz, 1H), 6.81 (dd, *J* = 2.4, 1.8 Hz, 1H), 6.74 (ddd, *J* = 7.8, 1.8, 1.2 Hz, 1H), 6.71 (dd, *J* = 7.2, 6.9 Hz, 1H), 6.56 (ddd, *J* = 7.8, 2.4, 1.2 Hz, 1H), 5.18 (s, 2H), 2.49 (s, 3H). ^13^C NMR (151 MHz, DMSO-*d*_6_) δ 166.99 162.35, 161.87 (d, *J*_C–F_ = 246.1 Hz), 161.84, 161.18, 151.77, 150.19, 148.51, 148.00, 144.75, 143.89, 136.31 (d, *J*_C–F_ = 3.0 Hz), 135.70, 130.85, 129.33 (d, *J*_C–F_ = 9.1 Hz), 128.66, 122.42, 120.75, 120.43, 117.39, 116.07 (d, *J*_C–F_ = 22.8 Hz), 115.36, 115.20, 113.19, 106.95, 104.83, 12.48. LRMS (ESI) *m*/*z* 548.2 [M + H]^+^. HRMS (ESI) *m*/*z* for C_31_H_22_FN_5_NaO_4_ [M + Na]^+^, calcd 570.1554, found 570.1547. HPLC purity 91.44% (*t*_R_ = 24.11 min).

#### *N*-(4-{[5-(3-Aminophenyl)-6-(1*H*-pyrazol-4-yl)furo[2,3-*d*]pyrimidin-4-yl]oxy}phenyl)-1-(4-fluorophenyl)-2-oxo-1,2-dihydropyridine-3-carboxamide (**21**)

To a solution of **49f** (86 mg, 0.14 mmol, 1.0 equiv) in ethanol (8.0 mL) and *N*,*N*-dimethylformamide (0.75 mL) was added SnCl_2_·2H_2_O (93 mg, 0.41 mmol, 3.0 equiv), and then, the reaction mixture was stirred at room temperature. After stirring for 20 h, the reaction mixture was concentrated *in vacuo*. Then, the mixture was dissolved in dichloromethane (10 mL) and quenched with 6N NaOH until the pH value was over 9.0. The combined organic layers were washed with brine, dried over MgSO_4_, concentrated *in vacuo*, and purified by flash chromatography (3% methanol in dichloromethane with 1% NH_4_OH) to yield the title compound **21** (16 mg, 0.03 mmol, 20%) as a beige solid. ^1^H NMR (400 MHz, CDCl_3_) δ 11.85 (s, 1H), 8.75 (dd, *J* = 7.0, 2.0 Hz, 1H), 8.48 (s, 1H), 7.84 (s, 2H), 7.76 (d, *J* = 9.0 Hz, 2H), 7. 7.61 (dd, *J* = 6.8, 2.0 Hz, 1H), 7.41 (dd, *J* = 9.2, 4.8 Hz, 2H), 7.30–7.23 (m, 3H), 7.11 (d, *J* = 9.0 Hz, 2H), 6.97 (d, *J* = 7.6 Hz, 1H), 6.89 (dd, *J* = 2.4, 1.6 Hz, 1H), 6.74 (d, *J* = 8.8 Hz, 1H), 6.61 (dd, *J* = 7.0, 6.8 Hz, 1H), 3.72 (s, 2H). ^13^C NMR (151 MHz, 5% CD_3_OD in CDCl_3_) δ 166.89, 162.90, 162.59 (d, *J*_C–F_ = 250.4 Hz), 162.31, 161.52, 161.42, 151.58, 148.50, 146.31, 145.09, 144.99, 141.77, 135.62, 135.40, 135.29, 131.51, 129.31, 128.26 (d, *J*_C–F_ = 8.9 Hz), 121.72, 121.42, 121.32, 120.40, 116.61 (d, *J*_C–F_ = 20.5 Hz), 115.25, 112.80, 111.17, 107.27, 107.12. LRMS (ESI) *m*/*z* 600.5 [M + H]^+^. HRMS (ESI) *m*/*z* for C_33_H_22_FN_7_NaO_4_ [M + Na]^+^, calcd 622.1615, found 622.1614. UPLC purity 94.12% (*t*_R_ = 2.259 min).

#### *N*-(4-{[5-(3-Aminophenyl)-6-(1-methyl-1*H*-pyrazol-4-yl)furo[2,3-*d*]pyrimidin-4-yl]oxy}phenyl)-1-(4-fluorophenyl)-2-oxo-1,2-dihydropyridine-3-carboxamide (**22**)

To a solution of **56** (3.11 g, 5.17 mmol, 1.0 equiv) in *N*,*N*-dimethylformamide (51.7 mL) and tetrahydrofuran (51.7 mL) were added (3-aminophenyl)boronic acid (1.06 g, 7.75 mmol, 1.5 equiv), Pd(dppf)Cl_2_ (1.14 g, 1.56 mmol, 30 mol %), and 2 M Na_2_CO_3(aq)_ (10.2 mL, 4.0 equiv). The reaction mixture was degassed for 30 min, refilled with Argon_(g)_, and stirred at 110 °C. After stirring for 16 h, the reaction mixture was cooled down to room temperature, filtered through Celite, added with water (10 mL), and extracted into CH_2_Cl_2_ (10 mL × 3). The combined organic layers were washed with brine, dried over MgSO_4_, concentrated *in vacuo*, and purified by flash chromatography (2–3% methanol in dichloromethane) to yield the title compound **22** (2.74 g, 4.47 mmol, 86%) as a white solid. ^1^H NMR (400 MHz, DMSO-*d*_6_) δ 11.97 (s, 1H), 8.58 (dd, *J* = 7.6, 2.4 Hz, 1H), 8.45 (s, 1H), 8.11 (dd, *J* = 7.0, 2.4 Hz, 1H), 8.05 (d, *J* = 0.8 Hz, 1H), 7.73 (d, *J* = 9.0 Hz, 2H), 7.61 (dd, *J* = 8.8, 4.8 Hz, 2H), 7.52 (d, *J* = 0.8 Hz, 1H), 7.42 (dd, *J* = 8.8, 8.4 Hz, 2H), 7.20 (d, *J* = 9.0 Hz, 2H), 7.13 (dd, *J* = 7.8, 7.6 Hz, 1H), 6.81 (dd, *J* = 2.2, 1.6 Hz, 1H), 6.74 (ddd, *J* = 7.6, 1.6, 1.0 Hz, 1H), 6.72 (dd, *J* = 7.6, 7.0 Hz, 1H), 6.61 (ddd, *J* = 7.8, 2.2, 1.0 Hz, 1H), 5.21 (s, 2H), 3.87 (s, 3H). ^13^C NMR (101 MHz, DMSO-*d*_6_) δ 166.71, 162.53, 161.85 (d, *J*_C–F_ = 242.6 Hz), 161.84, 161.19, 151.87, 148.69, 148.03, 144.77, 144.34, 143.93, 136.65, 136.32, 135.70, 130.89, 129.44, 129.35 (d, *J*_C–F_ = 9.0 Hz), 128.96, 122.37, 120.72, 120.43, 117.41, 116.08 (d, *J*_C–F_ = 23.1 Hz), 115.38, 113.80, 112.69, 110.95, 106.96, 106.24, 38.78. LRMS (ESI) *m*/*z* 614.3 [M + H]^+^. HRMS (ESI) *m*/*z* for C_34_H_24_FN_7_NaO_4_ [M + Na]^+^, calcd 636.1772, found 636.1771. UPLC purity 99.67% (*t*_R_ = 2.479 min).

#### *N*-(4-{[5-(3-Aminophenyl)-6-(1,3-dimethyl-1*H*-pyrazol-4-yl)furo[2,3-*d*]pyrimidin-4-yl]oxy}phenyl)-1-(4-fluorophenyl)-2-oxo-1,2-dihydropyridine-3-carboxamide (**23**)

To a solution of **49g** (100 mg, 0.15 mmol, 1.0 equiv) in ethanol (9.0 mL) and *N*,*N*-dimethylformamide (2.0 mL) was added SnCl_2_·2H_2_O (103 mg, 2.13 mmol, 8.0 equiv), and then, the reaction mixture was stirred at 75 °C. After stirring for 16 h, the reaction mixture was cooled down to room temperature and concentrated *in vacuo*. Then, the mixture was dissolved in dichloromethane (10 mL) and quenched with 6 N NaOH until the pH value was over 9.0. The combined organic layers were washed with brine, dried over MgSO_4_, concentrated *in vacuo*, and purified by Combiflash automated flash chromatography (3% methanol in dichloromethane) to yield the title compound **23** (50 mg, 0.08 mmol, 52%) as a white solid. ^1^H NMR (600 MHz, CDCl_3_) δ 11.85 (s, 1H), 8.75 (dd, *J* = 7.2, 2.1 Hz, 1H), 8.45 (s, 1H), 7.76 (d, *J* = 9.0 Hz, 2H), 7.60 (dd, *J* = 6.9, 2.1 Hz, 1H), 7.41 (dd, *J* = 9.0, 4.8 Hz, 2H), 7.33 (s, 1H), 7.27 (dd, *J* = 9.0, 7.8 Hz, 2H), 7.19 (dd, *J* = 7.8, 7.8 Hz, 1H), 7.12 (d, *J* = 9.0 Hz, 2H), 6.91 (ddd, *J* = 7.8, 1.8, 1.2 Hz, 1H), 6.85 (dd, *J* = 2.4, 1.8 Hz, 1H), 6.69 (ddd, *J* = 7.8, 2.4, 1.2 Hz, 1H), 6.60 (dd, *J* = 7.2, 6.9 Hz, 1H), 3.80 (s, 3H), 3.68 (s, 2H), 2.31 (s, 3H). ^13^C NMR (151 MHz, DMSO-*d*_6_) δ 166.86, 162.59, 161.87 (d, *J*_C–F_ = 246.0 Hz), 161.84, 161.19, 151.86, 148.55, 148.05, 146.08, 145.05, 144.76, 143.92, 136.32, 135.69, 131.63, 131.30, 129.34 (d, *J*_C–F_ = 8.9 Hz), 128.78, 122.38, 120.74, 120.44, 117.51, 116.07 (d, *J*_C–F_ = 23.0 Hz), 115.39, 114.06, 113.46, 108.45, 106.96, 105.79, 38.49, 12.94. LRMS (ESI) *m*/*z* 628.2 [M + H]^+^. HRMS (ESI) *m*/*z* for C_35_H_26_FN_7_NaO_4_ [M+ Na]^+^, calcd 650.1928, found 650.1925. HPLC purity 99.82% (*t*_R_ = 22.61 min).

#### *N*-(4-{[5-(3-Aminophenyl)-6-(3,5-dimethyl-1,2-oxazol-4-yl)furo[2,3-*d*]pyrimidin-4-yl]oxy}phenyl)-1-(4-fluorophenyl)-2-oxo-1,2-dihydropyridine-3-carboxamide (**24**)

To a solution of **49h** (87 mg, 0.13 mmol, 1.0 equiv) in ethanol (18.0 mL) and *N*,*N*-dimethylformamide (0.5 mL) was added SnCl_2_·2H_2_O (75 mg, 0.33 mmol, 2.5 equiv), and then, the reaction mixture was stirred at 75 °C. After stirring for 16 h, the reaction mixture was cooled down to room temperature and concentrated *in vacuo*. Then, the mixture was dissolved in dichloromethane (10 mL) and quenched with 6 N NaOH until the pH value was over 9.0. The combined organic layers were washed with brine, dried over MgSO_4_, concentrated *in vacuo*, and purified by Combiflash automated flash chromatography (25–50% ethyl acetate in dichloromethane) to yield the title compound **24** (38 mg, 0.06 mmol, 46%) as a white solid. ^1^H NMR (600 MHz, CDCl_3_) δ 11.88 (s, 1H), 8.75 (dd, *J* = 7.5, 2.4 Hz, 1H), 8.53 (s, 1H), 7.80 (d, *J* = 9.0 Hz, 2H), 7.61 (dd, *J* = 7.2, 2.4 Hz, 1H), 7.41 (dd, *J* = 9.0, 4.8 Hz, 2H), 7.30–7.25 (m, 2H), 7.18–7.14 (m, 3H), 6.86 (ddd, *J* = 7.8, 1.8, 0.9 Hz, 1H), 6.80 (dd, *J* = 2.4, 1.8 Hz, 1H), 6.66 (ddd, *J* = 8.4, 2.4, 0.0 Hz, 1H), 6.61 (dd, *J* = 7.5, 7.2 Hz, 1H), 3.67 (s, 2H), 2.16 (s, 3H), 2.14 (s, 3H). ^13^C NMR (151 MHz, CDCl_3_) δ 169.42, 168.04, 163.60, 162.75 (d, *J*_C–F_ = 250.5 Hz), 162.48, 161.28, 159.45, 153.13, 148.29, 146.54, 145.08, 141.69, 141.47, 136.09, 135.90, 131.26, 129.56, 128.44 (d, *J*_C–F_ = 9.1 Hz), 122.39, 121.96, 121.53, 120.13, 118.54, 116.86 (d, *J*_C–F_ = 23.1 Hz), 116.15, 114.91, 107.24, 106.73, 105.69, 12.12, 10.96. LRMS (ESI) *m*/*z* 629.2 [M + H]^+^. HRMS (ESI) *m*/*z* for C_35_H_25_FN_6_NaO_5_ [M + Na]^+^, calcd 651.1768, found 651.1762. HPLC purity 87.74% (*t*_R_ = 25.17 min).

#### *N*-(4-{[5-(3-Aminophenyl)-6-(1*H*-pyrazol-3-yl)furo[2,3-*d*]pyrimidin-4-yl]oxy}phenyl)-1-(4-fluorophenyl)-2-oxo-1,2-dihydropyridine-3-carboxamide (**25**)

To a solution of **50i** (113 mg, 0.15 mmol, 1.0 equiv) in dichloromethane (6.0 mL) at 0 °C was added trifluoroacetic acid (534 μL, 6.97 mmol, 45.0 equiv), and then, the reaction mixture was stirred at room temperature. After stirring for 16 h, the reaction mixture was concentrated *in vacuo* and dissolved in dichloromethane (4.0 mL), methanol (1.0 mL), and NH_4_OH (1.0 mL) at 0 °C and then stirred at room temperature. After stirring for a further 2 h, the reaction mixture was concentrated *in vacuo* and purified by Combiflash automated flash chromatography (2% methanol in dichloromethane) to yield the title compound **25** (36 mg, 0.06 mmol, 39%) as a beige solid. ^1^H NMR (600 MHz, DMSO-*d*_6_) δ 11.96 (s, 1H), 8.57 (dd, *J* = 7.5, 2.4 Hz, 1H), 8.50 (s, 1H), 8.09 (dd, *J* = 6.6, 2.4 Hz, 1H), 7.79 (s, 1H), 7.74 (d, *J* = 9.0 Hz, 2H), 7.60 (dd, *J* = 9.0, 4.8 Hz, 2H), 7.41 (dd, *J* = 9.0, 8.4 Hz, 2H), 7.21 (d, *J* = 9.0 Hz, 2H), 7.09 (dd, *J* = 8.1, 7.5 Hz, 1H), 6.83 (dd, *J* = 2.4, 1.8 Hz, 1H), 6.77 (d, *J* = 7.5 Hz, 1H), 6.71 (dd, *J* = 7.5, 6.6 Hz, 1H), 6.60 (d, *J* = 8.1 Hz, 1H), 6.27 (d, *J* = 2.4 Hz, 1H), 5.14 (s, 2H). ^13^C NMR (151 MHz, DMSO-*d*_6_) δ 166.81, 163.09, 161.88 (d, *J*_C–F_ = 245.8 Hz), 161.84, 161.20, 152.52, 148.35, 148.00, 144.77, 143.88, 140.70, 136.31, 135.74, 130.87, 129.64, 129.33 (d, *J*_C–F_ = 8.9 Hz), 128.55, 122.38, 120.76, 120.45, 117.84, 116.08 (d, *J*_C–F_ = 23.3 Hz), 115.86, 115.44, 113.75, 106.98, 106.09, 104.56, 69.79. LRMS (ESI) *m*/*z* 600.2 [M + H]^+^. HRMS (ESI) *m*/*z* for C_33_H_22_FN_7_NaO_4_ [M + Na]^+^, calcd 622.1615, found 622.1768. UPLC purity 91.84% (*t*_R_ = 2.298 min).

#### *N*-(4-{[5-(3-Aminophenyl)-6-(thiophen-3-yl)furo[2,3-*d*]pyrimidin-4-yl]oxy}phenyl)-1-(4-fluorophenyl)-2-oxo-1,2-dihydropyridine-3-carboxamide (**26**)

To a solution of **49j** (375 mg, 0.58 mmol, 1.0 equiv) in ethanol (11.6 mL), dichloromethane (11.6 mL), and water (2.3 mL) were added iron powder (195 mg, 3.49 mmol, 6.0 equiv) and sat. NH_4_Cl_(aq)_ (1.2 mL), and then, the reaction mixture was stirred at 80 °C. After stirring for 6 h, the reaction mixture was cooled down to room temperature and concentrated *in vacuo*. Then, the mixture was dissolved in dichloromethane (10 mL) and washed with water (10 mL × 3). The combined organic layers were dried over MgSO_4_, concentrated *in vacuo*, and purified by flash chromatography (0.5–1% methanol in dichloromethane) to yield the title compound **26** (83 mg, 0.13 mmol, 23%) as a white solid. ^1^H NMR (600 MHz, DMSO-*d*_6_) δ 11.97 (s, 1H), 8.58 (dd, *J* = 7.2, 2.4 Hz, 1H), 8.50 (s, 1H), 8.10 (dd, *J* = 6.6, 2.4 Hz, 1H), 7.91 (dd, *J* = 3.0, 1.2 Hz, 1H), 7.73 (d, *J* = 9.0 Hz, 2H), 7.62 (dd, *J* = 5.4, 3.0 Hz, 1H), 7.61 (dd, *J* = 9.0, 4.8 Hz, 2H), 7.42 (dd, *J* = 9.0, 8.4 Hz, 2H), 7.19 (d, *J* = 9.0 Hz, 2H), 7.13 (dd, *J* = 8.1, 7.5 Hz, 1H),7.08 (dd, *J* = 5.4, 1.2 Hz, 1H), 6.79 (dd, *J* = 2.1, 1.8 Hz, 1H), 6.73 (ddd, *J* = 7.5, 1.8, 1.2 Hz, 1H), 6.71 (dd, *J* = 7.2, 6.6 Hz, 1H), 6.63 (ddd, *J* = 8.1, 2.1, 1.2 Hz, 1H), 5.22 (s, 2H). ^13^C NMR (101 MHz, DMSO-*d*_6_) δ 166.63, 163.09, 161.87 (d, *J*_C–F_ = 246.1 Hz), 161.84, 161.19, 152.56, 148.74, 147.97, 145.76, 144.76, 143.92, 136.32, 135.74, 131.60, 131.06, 129.69, 129.34 (d, *J*_C–F_ = 8.9 Hz), 129.03, 127.61, 125.35, 125.04, 122.35, 120.73, 120.43, 117.43, 116.07 (d, *J*_C–F_ = 23.1 Hz), 115.39, 114.48, 113.98, 106.96, 106.43. LRMS (ESI) *m*/*z* 616.2 [M + H]^+^. HRMS (ESI) *m*/*z* for C_34_H_22_FN_5_NaO_4_S [M + Na]^+^, calcd 638.1274, found 638.1272. HPLC purity 99.59% (*t*_R_ = 27.95 min).

#### 1-(4-Fluorophenyl)-*N*-(4-{[6-(1-methyl-1*H*-pyrazol-4-yl)furo[2,3-*d*]pyrimidin-4-yl]oxy}phenyl)-2-oxo-1,2-dihydropyridine-3-carboxamide (**27**) and 1-(4-Fluorophenyl)-*N*-[4-({5-[3-(methylamino)phenyl]-6-(1-methyl-1*H*-pyrazol-4-yl)furo[2,3-*d*]pyrimidin-4-yl}oxy)phenyl]-2-oxo-1,2-dihydropyridine-3-carboxamide (**29**)

To a solution of **56** (76 mg, 0.13 mmol, 1.0 equiv) in *N*,*N*-dimethylformamide (2.5 mL) and tetrahydrofuran (2.5 mL) were added *N*-methyl-3-(4,4,5,5-tetramethyl-1,3,2-dioxaborolan-2-yl)aniline (44 mg, 0.19 mmol, 1.5 equiv), Pd(dppf)Cl_2_ (19 mg, 0.03 mmol, 21 mol %), and 2 M Na_2_CO_3(aq)_ (0.3 mL, 4.0 equiv). The reaction mixture was degassed for 30 min, refilled with Argon_(g)_, and stirred at 110 °C. After stirring for 12 h, the reaction mixture was cooled down to room temperature, filtered through Celite, added with water (10 mL), and extracted into CH_2_Cl_2_ (10 mL × 3), The combined organic layers were washed with brine, dried over MgSO_4_, concentrated *in vacuo*, and purified by flash chromatography (2% methanol in dichloromethane) to yield the title compound **27** (19 mg, 0.04 mmol, 29%) as a white solid and **29** (41 mg, 0.07 mmol, 52%) as a light-yellow solid. For **27**: ^1^H NMR (600 MHz, DMSO-*d*_6_) δ 12.00 (s, 1H), 8.60 (dd, *J* = 7.5, 1.8 Hz, 1H), 8.45 (s, 1H), 8.35 (s, 2H), 8.12 (dd, *J* = 6.6, 1.8 Hz, 1H), 8.02 (s, 1H), 7.79 (d, *J* = 9.0 Hz, 2H), 7.62 (dd, *J* = 9.0, 4.8 Hz, 2H), 7.43 (dd, *J* = 9.0, 8.4 Hz, 2H), 7.30 (d, *J* = 9.0 Hz, 2H), 7.13 (s, 1H), 6.73 (dd, *J* = 7.5, 6.6 Hz, 1H), 3.92 (s, 3H). ^13^C NMR (151 MHz, DMSO-*d*_6_) δ 167.58, 162.13, 161.88 (d, *J*_C–F_ = 245.8 Hz), 161.85, 161.26, 151.63, 149.38, 148.08, 144.81, 143.97, 136.72, 136.33, 135.91, 129.35 (d, *J*_C–F_ = 8.9 Hz), 129.21, 122.29, 120.95, 120.44, 116.09 (d, *J*_C–F_ = 23.08 Hz), 111.50, 106.97, 106.39, 95.19, 38.84. LRMS (ESI) *m*/*z* 523.2 [M + H]^+^. HRMS (ESI) *m*/*z* for C_28_H_19_FN_6_NaO_4_ [M + Na]^+^, calcd 545.1350, found 545.1361. HPLC purity 99.68% (*t*_R_ = 22.82 min). For **29**: ^1^H NMR (600 MHz, DMSO-*d*_6_) δ 11.96 (s, 1H), 8.58 (dd, *J* = 7.2, 2.4 Hz, 1H), 8.45 (s, 1H), 8.11 (dd, *J* = 6.6, 2.4 Hz, 1H), 8.06 (s, 1H), 7.73 (d, *J* = 9.0 Hz, 2H), 7.61 (dd, *J* = 9.0, 4.8 Hz, 2H), 7.54 (s, 1H), 7.42 (dd, *J* = 9.0, 8.4 Hz, 2H), 7.21–7.16 (m, 3H), 6.80 (dd, *J* = 2.4, 1.5 Hz, 1H), 6.78 (ddd, *J* = 7.8, 1.5, 1.2 Hz, 1H), 6.71 (dd, *J* = 7.2, 6.6 Hz, 1H), 6.59 (ddd, *J* = 8.4, 2.4, 1.2 Hz, 1H), 5.78 (q, *J* = 4.8 Hz, 1H), 3.86 (s, 3H), 2.65 (d, *J* = 4.8 Hz, 3H). ^13^C NMR (151 MHz, DMSO-*d*_6_) δ 166.74, 162.65, 161.88 (d, *J*_C–F_ = 246.1 Hz), 161.84, 161.20, 151.90, 149.83, 148.10, 144.77, 144.36, 143.93, 136.67, 136.32, 135.71, 130.93, 129.49, 129.35 (d, *J*_C–F_ = 8.9 Hz), 128.86, 122.35, 120.79, 120.44, 117.13, 116.08 (d, *J*_C–F_ = 23.1 Hz), 113.03, 112.83, 111.87, 110.96, 106.97, 106.31, 29.66. LRMS (ESI) *m*/*z* 628.3 [M + H]^+^. HRMS (ESI) *m*/*z* for C_35_H_26_FN_7_NaO_4_ [M + Na]^+^, calcd 650.1928, found 650.1934. HPLC purity 94.22% (*t*_R_ = 24.66 min).

#### *N*-[4-({5-[3-(Acetylamino)phenyl]-6-(1-methyl-1*H*-pyrazol-4-yl)furo[2,3-*d*]pyrimidin-4-yl}oxy)phenyl]-1-(4-fluorophenyl)-2-oxo-1,2-dihydropyridine-3-carboxamide (**28**)

To a solution of **22** (32 mg, 0.05 mmol, 1.0 equiv) in dichloromethane (1.0 mL) were added triethylamine (30 μL, 0.22 mmol, 4.1 equiv) and acetyl chloride (12 μL, 0.17 mmol, 3.2 equiv), and then, the reaction mixture was stirred at room temperature. After stirring for 16 h, the reaction mixture was quenched with iced water (10 mL) and washed with sat. NaHCO_3(aq)_ (10 mL × 3) and brine. The combined organic layers were dried over MgSO_4_, concentrated *in vacuo*, and purified by flash chromatography (5% methanol in dichloromethane) to yield the title compound **28** (22 mg, 0.03 mmol, 64%) as a white solid. ^1^H NMR (400 MHz, DMSO-*d*_6_) δ 11.96 (s, 1H), 10.08 (s, 1H), 8.57 (dd, *J* = 7.4, 2.2 Hz, 1H), 8.47 (s, 1H), 8.12 (s, 1H), 8.10 (dd, *J* = 6.4, 2.2 Hz, 1H), 7.93 (dd, *J* = 2.8, 1.8 Hz, 1H), 7.72 (d, *J* = 10.2 Hz, 2H), 7.65–7.53 (m, 4H), 7.45–7.36 (m, 3H), 7.33 (dd, *J* = 7.6, 1.8 Hz, 1H), 7.19 (d, *J* = 10.2 Hz, 2H), 6.71 (dd, *J* = 7.4, 6.4 Hz, 1H), 3.86 (s, 3H), 2.05 (s, 3H). ^13^C NMR (101 MHz, DMSO-*d*_6_) δ 168.59, 166.75, 162.53, 161.88 (d, *J*_C–F_ = 246.8 Hz), 161.85, 161.19, 152.04, 147.96, 144.77, 144.64, 143.92, 139.31, 136.71, 136.32 (d, *J*_C–F_ = 3.0 Hz), 135.74, 130.84, 129.72, 129.35 (d, *J*_C–F_ = 9.1 Hz), 128.88, 125.02, 122.32, 120.76, 120.68, 120.43, 118.89, 116.08 (d, *J*_C–F_ = 23.1 Hz), 111.85, 110.65, 106.97, 106.15, 38.80, 24.03. LRMS (ESI) *m*/*z* 656.2 [M + H]^+^. HRMS (ESI) *m*/*z* for C_36_H_26_FN_7_NaO_5_ [M + Na]^+^, calcd 678.1877, found 678.1872. HPLC purity 93.47% (*t*_R_ = 22.07 min).

#### *N*-[4-({5-[3-(Dimethylamino)phenyl]-6-(1-methyl-1*H*-pyrazol-4-yl)furo[2,3-*d*]pyrimidin-4-yl}oxy)phenyl]-1-(4-fluorophenyl)-2-oxo-1,2-dihydropyridine-3-carboxamide (**30**)

To a solution of **56** (80 mg, 0.13 mmol, 1.0 equiv) in *N*,*N*-dimethylformamide (4.0 mL) were added (3-(dimethylamino)phenyl)boronic acid (44 mg, 0.27 mmol, 2.0 equiv), Pd(dppf)Cl_2_ (29 mg, 0.04 mmol, 30 mol %), and 2 M Na_2_CO_3(aq)_ (0.3 mL, 4.1 equiv). The reaction mixture was degassed for 30 min, refilled with Argon_(g)_, and stirred at 100 °C. After stirring for 16 h, the reaction mixture was cooled down to room temperature, filtered through Celite, added with water (10 mL), and extracted into CH_2_Cl_2_ (10 mL × 3), The combined organic layers were washed with brine, dried over Na_2_SO_4_, concentrated *in vacuo*, and purified by flash chromatography (1% methanol in dichloromethane) to yield the title compound **30** (51 mg, 0.08 mmol, 60%) as a brown solid. ^1^H NMR (600 MHz, DMSO-*d*_6_) δ 11.95 (s, 1H), 8.58 (dd, *J* = 7.5, 2.4 Hz, 1H), 8.46 (s, 1H), 8.11 (dd, *J* = 6.6, 2.4 Hz, 1H), 8.07 (s, 1H), 7.73 (d, *J* = 8.7 Hz, 2H), 7.61 (dd, *J* = 9.0, 4.8 Hz, 2H), 7.54 (s, 1H), 7.42 (dd, *J* = 9.0, 8.4 Hz, 2H), 7.28 (dd, *J* = 8.1, 7.5 Hz, 1H), 7.19 (d, *J* = 8.7 Hz, 2H), 7.04 (dd, *J* = 2.1, 1.8 Hz, 1H), 6.90 (d, *J* = 7.5 Hz, 1H), 6.77 (dd, *J* = 8.1, 2.1 Hz, 1H), 6.72 (dd, *J* = 7.5, 6.6 Hz, 1H), 3.87 (s, 3H), 2.88 (s, 6H). ^13^C NMR (151 MHz, DMSO-*d*_6_) δ 166.63, 162.35, 161.90 (d, *J*_C–F_ = 248.4 Hz), 161.78, 160.72, 151.14, 149.91, 147.85, 144.41, 144.32, 136.80, 135.35, 130.79, 128.49, 128.37, 128.17 (d, *J*_C–F_ = 9.1 Hz), 121.43, 121.11, 120.60, 117.40, 115.99 (d, *J*_C–F_ = 23.1 Hz), 113.66, 112.64, 111.80, 111.35, 106.74, 106.48, 28.97, 28.94. LRMS (ESI) *m*/*z* 642.2 [M + H]^+^. HRMS (ESI) *m*/*z* for C_36_H_28_FN_7_NaO_4_ [M + Na]^+^, calcd 664.2085, found 664.2092. UPLC purity 92.53% (*t*_R_ = 2.944 min).

#### *N*-[4-({5-[3-(Aminomethyl)phenyl]-6-(1-methyl-1*H*-pyrazol-4-yl)furo[2,3-*d*]pyrimidin-4-yl}oxy)phenyl]-1-(4-fluorophenyl)-2-oxo-1,2-dihydropyridine-3-carboxamide (**31**)

To a solution of **56** (80 mg, 0.13 mmol, 1.0 equiv) in *N*,*N*-dimethylformamide (2.2 mL) and tetrahydrofuran (2.2 mL) were added (3-(aminomethyl)phenyl)boronic acid (30 mg, 0.20 mmol, 1.5 equiv), Pd(dppf)Cl_2_ (20 mg, 0.03 mmol, 21 mol %), and 2 M Na_2_CO_3(aq)_ (0.3 mL, 4.1 equiv). The reaction mixture was degassed for 30 min, refilled with Argon_(g)_, and stirred at 100 °C. After stirring for 16 h, the reaction mixture was cooled down to room temperature, filtered through Celite, added with water (10 mL), and extracted into CH_2_Cl_2_ (10 mL × 3). The combined organic layers were washed with brine, dried over Na_2_SO_4_, concentrated *in vacuo*, and purified by flash chromatography (7–8% methanol in dichloromethane) to yield the title compound **31** (63 mg, 0.10 mmol, 75%) as a beige solid. ^1^H NMR (600 MHz, DMSO-*d*_6_) δ 11.96 (s, 1H), 8.57 (dd, *J* = 6.9, 1.8 Hz, 1H), 8.48 (s, 1H), 8.11 (dd, *J* = 6.9, 1.8 Hz, 1H), 8.07 (s, 1H), 7.72 (d, *J* = 9.0 Hz, 2H), 7.60 (dd, *J* = 9.0, 5.4 Hz, 2H), 7.55 (s, 1H), 7.42 (dd, *J* = 9.0, 8.4 Hz, 2H), 7.20 (d, *J* = 9.0 Hz, 2H), 7.09 (dd, *J* = 10.8, 3.0 Hz, 1H), 6.71 (dd, *J* = 7.2, 6.6 Hz, 1H), 3.88–3.83 (m, 5H). ^13^C NMR (151 MHz, DMSO-*d*_6_) δ 166.77, 162.60, 161.88 (d, *J*_C–F_ = 246.0 Hz), 161.84, 161.20, 152.05, 147.99, 144.77, 144.61, 143.94, 136.63, 136.31, 135.73, 130.35, 129.56, 129.34 (d, *J*_C–F_ = 8.6 Hz), 129.23, 128.66, 128.36, 127.45, 122.28, 120.79, 120.42, 116.08 (d, *J*_C–F_ = 23.0 Hz), 111.93, 110.70, 106.97, 106.21, 44.56, 39.09. LRMS (ESI) *m*/*z* 628.2 [M + H]^+^. HRMS (ESI) *m*/*z* for C_35_H_27_FN_7_O_4_ [M + H]^+^, calcd 628.2109, found 628.2110. HPLC purity 93.29% (*t*_R_ = 15.57 min).

#### 1-(4-Fluorophenyl)-*N*-{4-[(5-{3-[(methylamino)methyl]phenyl}-6-(1-methyl-1*H*-pyrazol-4-yl)furo[2,3-*d*]pyrimidin-4-yl)oxy]phenyl}-2-oxo-1,2-dihydropyridine-3-carboxamide (**32**)

To a solution of **56** (80 mg, 0.13 mmol, 1.0 equiv) in *N*,*N*-dimethylformamide (2.6 mL) and tetrahydrofuran (2.6 mL) were added *N*-methyl[3-(4,4,5,5-tetramethyl-1,3,2-dioxaborolan-2-yl)phenyl]methanamine (66 mg, 0.27 mmol, 2.0 equiv), Pd(dppf)Cl_2_ (29 mg, 0.04 mmol, 30 mol %), and 2 M Na_2_CO_3(aq)_ (0.3 mL, 4.1 equiv). The reaction mixture was degassed for 30 min, refilled with Argon_(g)_, and stirred at 100 °C. After stirring for 16 h, the reaction mixture was cooled down to room temperature, filtered through Celite, added with water (10 mL), and extracted into CH_2_Cl_2_ (10 mL × 3). The combined organic layers were washed with brine, dried over Na_2_SO_4_, concentrated *in vacuo*, and purified by flash chromatography (2–3% methanol in dichloromethane) to yield the title compound **32** (58 mg, 0.09 mmol, 68%) as a beige solid. ^1^H NMR (600 MHz, DMSO-*d*_6_) δ 11.95 (s, 1H), 8.57 (dd, *J* = 7.2, 2.1 Hz, 1H), 8.47 (s, 1H), 8.11 (dd, *J* = 6.9, 2.1 Hz, 1H), 8.05 (s, 1H), 7.72 (d, *J* = 9.0 Hz, 2H), 7.63–7.57 (m, 3H), 7.54–7.50 (m, 2H), 7.45–7.39 (m, 3H), 7.36 (ddd, *J* = 7.2, 1.8, 1.2 Hz, 1H), 7.19 (d, *J* = 9.0 Hz, 2H), 6.71 (dd, *J* = 7.2, 6.9 Hz, 1H), 3.86 (s, 3H), 3.67 (s, 2H), 2.18 (s, 3H). ^13^C NMR (151 MHz, DMSO-*d*_6_) δ 166.79, 162.63, 161.88 (d, *J*_C–F_ = 246.1 Hz), 161.84, 161.20, 152.03, 148.03, 144.77, 144.56, 143.93, 141.05, 136.58, 136.32, 135.70, 130.15, 129.80, 129.54, 129.35 (d, *J*_C–F_ = 8.9 Hz), 128.29, 128.24, 128.01, 122.28, 120.74, 120.43, 116.08 (d, *J*_C–F_ = 23.3 Hz), 112.11, 110.76, 106.97, 106.25, 54.82, 38.77, 35.39. LRMS (ESI) *m*/*z* 642.2 [M + H]^+^. HRMS (ESI) *m*/*z* for C_36_H_29_FN_7_O_4_ [M + H]^+^, calcd 642.2265, found 642.2258. HPLC purity 97.39% (*t*_R_ = 16.35 min).

#### *N*-{4-[(5-{3-[(Dimethylamino)methyl]phenyl}-6-(1-methyl-1*H*-pyrazol-4-yl)furo[2,3-*d*]pyrimidin-4-yl)oxy]phenyl}-1-(4-fluorophenyl)-2-oxo-1,2-dihydropyridine-3-carboxamide (**33**)

To a solution of **56** (80 mg, 0.13 mmol, 1.0 equiv) in *N*,*N*-dimethylformamide (2.6 mL) and tetrahydrofuran (2.6 mL) was added *N*,*N*-dimethyl[3-(4,4,5,5-tetramethyl-1,3,2-dioxaborolan-2-yl)phenyl]methanamine (52 mg, 0.20 mmol, 1.5 equiv), Pd(dppf)Cl_2_ (29 mg, 0.04 mmol, 30 mol %), and 2 M Na_2_CO_3(aq)_ (0.3 mL, 4.1 equiv). The reaction mixture was degassed for 30 min, refilled with Argon_(g)_, and stirred at 100 °C. After stirring for 16 h, the reaction mixture was cooled down to room temperature, filtered through Celite, added water (10 mL), and extracted into CH_2_Cl_2_ (10 mL × 3). The combined organic layers were washed with brine, dried over Na_2_SO_4_, concentrated *in vacuo*, and purified by flash chromatography (4–5% methanol in dichloromethane) to yield the title compound **33** (51 mg, 0.08 mmol, 58%) as a beige solid. ^1^H NMR (600 MHz, DMSO-*d*_6_) δ 11.95 (s, 1H), 8.58 (dd, *J* = 7.2, 2.4 Hz, 1H), 8.44 (s, 1H), 8.10 (dd, *J* = 6.9, 2.4 Hz, 1H), 7.91 (s, 1H), 7.72 (d, *J* = 8.4 Hz, 2H), 7.60 (dd, *J* = 9.0, 4.8 Hz, 2H), 7.42 (dd, *J* = 9.0, 9.0 Hz, 2H), 7.39 (d, *J* = 1.2 Hz, 1H), 7.17 (dd, *J* = 7.5, 1.8 Hz, 1H), 7.15–7.10 (m, 3H), 6.77 (dd, *J* = 8.1, 1.2 Hz, 1H), 6.71 (dd, *J* = 7.2, 6.9 Hz, 1H), 6.61 (ddd, *J* = 8.1, 7.5, 1.2 Hz, 1H), 5.03 (s, 2H), 3.85 (s, 3H). ^13^C NMR (151 MHz, 10% DMSO-*d*_6_ in CDCl_3_) δ 166.76, 162.50, 162.13 (d, *J*_C–F_ = 249.9 Hz), 161.93, 160.77, 151.48, 147.85, 144.51, 144.42, 141.38, 138.87, 137.00, 135.44, 135.40, 130.52, 130.27, 128.56, 128.26, 128.22, 128.04 (d, *J*_C–F_ = 8.8 Hz), 127.91, 121.57, 121.43, 120.71, 116.22 (d, *J*_C–F_ = 23.1 Hz), 112.08, 111.53, 106.80, 106.50, 63.66, 44.86, 38.66. LRMS (ESI) *m*/*z* 656.3 [M + H]^+^. HRMS (ESI) *m*/*z* for C_37_H_31_FN_7_O_4_ [M + H]^+^, calcd 656.2422, found 656.2430. HPLC purity 98.64% (*t*_R_ = 16.15 min).

#### 1-(4-Fluorophenyl)-*N*-[4-({6-(1-methyl-1*H*-pyrazol-4-yl)-5-[3-(piperazin-1-yl)phenyl]furo[2,3-*d*]pyrimidin-4-yl}oxy)phenyl]-2-oxo-1,2-dihydropyridine-3-carboxamide (**34**)

To a solution of **57k** (107 mg, 0.14 mmol, 1.0 equiv) in dichloromethane (1.4 mL) at 0 °C was added trifluoroacetic acid (260 μL, 3.40 mmol, 24.8 equiv), and then, the reaction mixture was stirred at room temperature. After stirring for 16 h, the reaction mixture was concentrated *in vacuo*, and purified by flash chromatography (8% methanol in dichloromethane) to yield the title compound **34** (88 mg, 0.13 mmol, 94%) as a white solid. ^1^H NMR (600 MHz, DMSO-*d*_6_) δ 11.96 (s, 1H), 8.58 (dd, *J* = 7.2, 2.4 Hz, 1H), 8.47 (s, 1H), 8.11 (dd, *J* = 6.6, 2.4 Hz, 1H), 8.08 (s, 1H), 7.73 (d, *J* = 9.0 Hz, 2H), 7.60 (dd, *J* = 9.0, 4.8 Hz, 2H), 7.54 (s, 1H), 7.42 (dd, *J* = 9.0, 9.0 Hz, 2H), 7.32 (dd, *J* = 8.4, 8.1 Hz, 1H), 7.26 (dd, *J* = 2.7, 1.8 Hz, 1H), 7.19 (d, *J* = 9.0 Hz, 2H), 7.04 (d, *J* = 8.4 Hz, 1H), 6.98 (dd, *J* = 8.1, 2.7 Hz, 1H), 6.72 (dd, *J* = 7.2, 6.6 Hz, 1H), 3.87 (s, 3H), 3.11 (s, 4H), 2.85 (s, 4H). ^13^C NMR (151 MHz, DMSO-*d*_6_) δ 166.76, 161.89 (d, *J*_C–F_ = 247.0 Hz), 161.84, 161.20, 151.97, 151.16, 148.15, 144.77, 144.45, 143.94, 136.62, 136.31, 135.70, 130.95, 129.60, 129.35 (d, *J*_C–F_ = 9.1 Hz), 128.98, 122.30, 120.78, 120.44, 120.20, 117.43, 116.09 (d, *J*_C–F_ = 23.0 Hz), 115.10, 112.60, 110.85, 106.98, 106.35, 48.45, 45.00. LRMS (ESI) *m*/*z* 683.3 [M + H]^+^. HRMS (ESI) *m*/*z* for C_38_H_32_FN_8_O_4_ [M + H]^+^, calcd 683.2531, found 683.2531. HPLC purity 98.01% (*t*_R_ = 16.43 min).

#### 1-(4-Fluorophenyl)-*N*-[4-({5-[3-(4-methylpiperazin-1-yl)phenyl]-6-(1-methyl-1*H*-pyrazol-4-yl)furo[2,3-*d*]pyrimidin-4-yl}oxy)phenyl]-2-oxo-1,2-dihydropyridine-3-carboxamide (**35**)

To a solution of **34** (70 mg, 0.10 mmol, 1.0 equiv) in methanol (3.5 mL) and dichloromethane (3.5 mL) were added formaldehyde (61 μL, 0.21 mmol, 2.0 equiv) and NaBH(OAc)_3_ (108 mg, 0.51 mmol, 5.0 equiv), and then, the reaction mixture was stirred at room temperature. After stirring for 1.5 h, the reaction mixture was concentrated *in vacuo*, dissolved in dichloromethane (20 mL), and washed with water (10 mL × 3). The combined organic layers were washed with brine, dried over Na_2_SO_4_, concentrated *in vacuo*, and purified by flash chromatography (5–6% methanol in dichloromethane) to yield the title compound **35** (27 mg, 0.04 mmol, 38%) as a white solid. ^1^H NMR (600 MHz, DMSO-*d*_6_) δ 11.96 (s, 1H), 8.58 (dd, *J* = 7.5, 2.1 Hz, 1H), 8.47 (s, 1H), 8.11 (dd, *J* = 6.9, 2.1 Hz, 1H), 8.08 (s, 1H), 7.73 (d, *J* = 9.0 Hz, 2H), 7.61 (dd, *J* = 9.0, 8.4 Hz, 2H), 7.31 (dd, *J* = 8.4, 7.8 Hz, 1H), 7.27 (dd, *J* = 3.0, 2.1 Hz, 1H), 7.19 (d, *J* = 9.0 Hz, 2H), 7.03 (ddd, *J* = 7.8, 2.1, 1.2 Hz, 1H), 6.98 (ddd, *J* = 8.4, 3.0, 1.2 Hz, 1H), 6.72 (dd, *J* = 7.5, 6.9 Hz, 1H), 3.87 (s, 3H), 3.14 (s, 4H), 2.40 (s, 4H), 2.18 (s, 3H). ^13^C NMR (151 MHz, DMSO-*d*_6_) δ 166.75, 161.89 (d, *J*_C–F_ = 250.2 Hz), 161.84, 161.19, 151.96, 150.77, 148.13, 144.76, 144.43, 143.94, 136.63, 136.32, 135.71, 130.93, 129.58, 129.34 (d, *J*_C–F_ = 8.9 Hz), 128.95, 122.30, 120.75, 120.44, 120.09, 117.36, 116.08 (d, *J*_C–F_ = 23.1 Hz), 115.08, 112.60, 110.84, 106.96, 106.33, 69.78, 54.44, 47.78, 45.65. LRMS (ESI) *m*/*z* 697.3 [M + H]^+^. HRMS (ESI) *m*/*z* for C_39_H_34_FN_8_O_4_ [M + H]^+^, calcd 697.2687, found 697.2684. UPLC purity 100.00% (*t*_R_ = 2.091 min).

#### *N*-(4-{[5-(2-Aminophenyl)-6-(1-methyl-1*H*-pyrazol-4-yl)furo[2,3-*d*]pyrimidin-4-yl]oxy}phenyl)-1-(4-fluorophenyl)-2-oxo-1,2-dihydropyridine-3-carboxamide (**36**)

To a solution of **56** (100 mg, 0.17 mmol, 1.0 equiv) in *N*,*N*-dimethylformamide (1.6 mL) and tetrahydrofuran (1.6 mL) were added 2-(4,4,5,5-tetramethyl-1,3,2-dioxaborolan-2-yl)aniline (47 mg, 0.21 mmol, 1.3 equiv), Pd(dppf)Cl_2_ (38 mg, 0.05 mmol, 31 mol %), and 2 M Na_2_CO_3(aq)_ (0.3 mL, 4.0 equiv). The reaction mixture was degassed for 30 min, refilled with Argon_(g)_, and stirred at 80 °C. After stirring for 16 h, the reaction mixture was cooled down to room temperature, filtered through Celite, added with water (10 mL), washed with NaHCO_3(aq)_ (20 mL), and extracted into CH_2_Cl_2_ (10 mL × 3). The combined organic layers were washed with brine, dried over MgSO_4_, concentrated *in vacuo*, and purified by Combiflash automated flash chromatography (5% methanol in dichloromethane) to yield the title compound **36** (20 mg, 0.03 mmol, 20%) as a brown solid. ^1^H NMR (600 MHz, CDCl_3_) δ 11.82 (s, 1H), 8.73 (dd, *J* = 7.2, 2.4 Hz, 1H), 8.47 (s, 1H), 7.73 (d, *J* = 9.0 Hz, 2H), 7.66 (d, *J* = 0.6 Hz, 1H), 7.59 (dd, *J* = 6.9, 2.4 Hz, 1H), 7.51 (s, 1H), 7.40 (dd, *J* = 9.0, 4.8 Hz, 2H), 7.29–7.21 (m, 4H), 7.08 (d, *J* = 9.0 Hz, 2H), 6.87–6.80 (m, 2H), 6.59 (dd, *J* = 7.2, 6.9 Hz, 1H), 3.88 (s, 3H), 3.73 (s, 2H). ^13^C NMR (151 MHz, CDCl_3_) δ 167.64, 163.14, 162.73 (d, *J*_C–F_ = 250.1 Hz), 162.45, 161.17, 152.34, 148.60, 145.73, 144.99, 144.86, 141.38, 137.67, 135.92, 135.80, 131.44, 129.88, 128.61, 128.43 (d, *J*_C–F_ = 8.6 Hz), 122.45, 121.87, 121.35, 118.58, 116.81 (d, *J*_C–F_ = 23.1 Hz), 115.84, 115.65, 111.97, 108.22, 107.30, 107.17, 39.20. LRMS (ESI) *m*/*z* 614.2 [M + H]^+^. HRMS (ESI) *m*/*z* for C_34_H_24_FN_7_NaO_4_ [M + Na]^+^, calcd 636.1772, found 636.1772. UPLC purity 96.65% (*t*_R_ = 2.669 min).

#### *N*-(4-{[5-(4-Aminophenyl)-6-(1-methyl-1*H*-pyrazol-4-yl)furo[2,3-*d*]pyrimidin-4-yl]oxy}phenyl)-1-(4-fluorophenyl)-2-oxo-1,2-dihydropyridine-3-carboxamide (**37**)

To a solution of **56** (80 mg, 0.13 mmol, 1.0 equiv) in *N*,*N*-dimethylformamide (2.6 mL) and tetrahydrofuran (2.6 mL) were added 4-(4,4,5,5-tetramethyl-1,3,2-dioxaborolan-2-yl)aniline (44 mg, 0.20 mmol, 1.5 equiv), Pd(dppf)Cl_2_ (29 mg, 0.04 mmol, 30 mol %), and 2 M Na_2_CO_3(aq)_ (0.3 mL, 4.1 equiv). The reaction mixture was degassed for 30 min, refilled with Argon_(g)_, and stirred at 110 °C. After stirring for 16 h, the reaction mixture was cooled down to room temperature, filtered through Celite, added with water (10 mL), and extracted into CH_2_Cl_2_ (10 mL × 3). The combined organic layers were washed with brine, dried over Na_2_SO_4_, concentrated *in vacuo*, and purified by flash chromatography (2% methanol in dichloromethane) to yield the title compound **37** (45 mg, 0.07 mmol, 55%) as beige solid. ^1^H NMR (600 MHz, DMSO-*d*_6_) δ 11.97 (s, 1H), 8.58 (dd, *J* = 7.2, 2.1 Hz, 1H), 8.43 (s, 1H), 8.11 (dd, *J* = 6.6, 2.1 Hz, 1H), 8.02 (s, 1H), 7.73 (d, *J* = 9.0 Hz, 2H), 7.61 (dd, *J* = 9.0, 4.8 Hz, 2H), 7.54 (d, *J* = 0.6 Hz, 1H), 7.42 (dd, *J* = 9.0, 8.4 Hz, 2H), 7.28 (d, *J* = 8.4 Hz, 2H), 7.20 (d, *J* = 9.0 Hz, 2H), 6.72 (dd, *J* = 7.2, 6.6 Hz, 1H), 6.64 (d, *J* = 8.4 Hz, 1H), 5.30 (s, 2H), 3.87 (s, 3H). ^13^C NMR (151 MHz, DMSO-*d*_6_) δ 166.73, 161.87 (d, *J*_C–F_ = 244.0 Hz), 161.84, 161.17, 151.73, 148.74, 148.09, 144.76, 143.92, 143.81, 136.46, 136.32, 135.67, 130.78, 129.34 (d, *J*_C–F_ = 8.8 Hz), 129.15, 122.32, 120.75, 120.44, 116.71, 116.07 (d, *J*_C–F_ = 23.0 Hz), 113.59, 112.75, 111.21, 106.96, 106.49, 73.50, 24.95. LRMS (ESI) *m*/*z* 614.2 [M + H]^+^. HRMS (ESI) *m*/*z* for C_34_H_24_FN_7_NaO_4_ [M + Na]^+^, calcd 636.1772, found 636.1773. HPLC purity 96.96% (*t*_R_ = 22.14 min).

#### *N*-[4-({5-[4-(Acetylamino)phenyl]-6-(1-methyl-1*H*-pyrazol-4-yl)furo[2,3-*d*]pyrimidin-4-yl}oxy)phenyl]-1-(4-fluorophenyl)-2-oxo-1,2-dihydropyridine-3-carboxamide (**38**)

To a solution of **56** (90 mg, 0.15 mmol, 1.0 equiv) in *N*,*N*-dimethylformamide (2.5 mL) and tetrahydrofuran (2.5 mL) were added [4-(acetylamino)phenyl]boronic acid (40 mg, 0.22 mmol, 1.5 equiv), Pd(dppf)Cl_2_ (16 mg, 0.02 mmol, 15 mol %), and 2 M Na_2_CO_3(aq)_ (0.3 mL, 4.1 equiv). The reaction mixture was degassed for 30 min, refilled with Argon_(g)_, and stirred at 100 °C. After stirring for 16 h, the reaction mixture was cooled down to room temperature, filtered through Celite, added with water (10 mL), and extracted into CH_2_Cl_2_ (10 mL × 3). The combined organic layers were washed with brine, dried over Na_2_SO_4_, concentrated *in vacuo*, and purified by flash chromatography (3% methanol in dichloromethane) to yield the title compound **38** (81 mg, 0.12 mmol, 83%) as a white solid. ^1^H NMR (600 MHz, DMSO-*d*_6_) δ 11.95 (s, 1H), 10.97 (s, 1H), 8.57 (dd, *J* = 7.5, 2.4 Hz, 1H), 8.45 (s, 1H), 8.09 (dd, *J* = 6.9, 2.4 Hz, 1H), 8.03 (s, 1H), 7.72 (d, *J* = 9.0 Hz, 2H), 7.70 (d, *J* = 8.4 Hz, 2H), 7.60 (dd, *J* = 9.0, 4.8 Hz, 2H), 7.57 (d, *J* = 8.4 Hz, 2H), 7.52 (s, 1H), 7.41 (dd, *J* = 9.0, 8.4 Hz, 2H), 7.19 (d, *J* = 9.0 Hz, 2H), 6.71 (dd, *J* = 7.5, 6.9 Hz, 1H), 3.86 (s, 3H), 2.06 (s, 3H). ^13^C NMR (151 MHz, DMSO-*d*_6_) δ 168.45, 166.73, 162.61, 161.86 (d, *J*_C−F_ = 246.1 Hz), 161.83, 161.16, 151.95, 147.97, 144.74, 144.43, 143.87, 139.30, 136.55, 136.30, 135.72, 130.55, 129.44, 129.32 (d, *J*_C−F_ = 8.9 Hz), 124.70, 122.28, 120.75, 120.43, 118.66, 116.07 (d, *J*_C−F_ = 23.1 Hz), 111.78, 110.78, 106.95, 106.29, 38.76, 24.04. LRMS (ESI) *m*/*z* 656.2 [M + H]^+^. HRMS (ESI) *m*/*z* for C_36_H_26_FN_7_NaO_5_ [M + Na]^+^, calcd 678.1877, found 678.1877. HPLC purity 97.91% (*t*_R_ = 21.33 min).

#### *N*-{4-[(5-{4-[(Dimethylamino)methyl]phenyl}-6-(1-methyl-1*H*-pyrazol-4-yl)furo[2,3-*d*]pyrimidin-4-yl)oxy]phenyl}-1-(4-fluorophenyl)-2-oxo-1,2-dihydropyridine-3-carboxamide (**39**)

To a solution of **56** (100 mg, 0.17 mmol, 1.0 equiv) in *N*,*N*-dimethylformamide (1.7 mL) and tetrahydrofuran (1.7 mL) were added *N*,*N*-dimethyl-1-[4-(4,4,5,5-tetramethyl-1,3,2-dioxaborolan-2-yl)phenyl]methanamine hydrochloride (74 mg, 0.25 mmol, 1.5 equiv), Pd(dppf)Cl_2_ (33 mg, 0.05 mmol, 27 mol %), and 2 M Na_2_CO_3(aq)_ (0.3 mL, 4.1 equiv). The reaction mixture was degassed for 30 min, refilled with Argon_(g)_, and stirred at 100 °C. After stirring for 16 h, the reaction mixture was cooled down to room temperature, filtered through Celite, added with water (10 mL), and extracted into CH_2_Cl_2_ (10 mL × 3). The combined organic layers were washed with brine, dried over Na_2_SO_4_, concentrated *in vacuo*, and purified by flash chromatography (3% methanol in dichloromethane) to yield the title compound **39** (70 mg, 0.11 mmol, 64%) as a white solid. ^1^H NMR (600 MHz, DMSO-*d*_6_) δ 11.94 (s, 1H), 8.57 (dd, *J* = 7.2, 1.8 Hz, 1H), 8.47 (s, 1H), 8.10 (dd, *J* = 6.6, 1.8 Hz, 1H), 8.06 (s, 1H), 7.71 (d, *J* = 9.0 Hz, 2H), 7.63–7.57 (m, 4H), 7.45 (s, 1H), 7.44–7.38 (m, 4H), 7.18 (d, *J* = 9.0 Hz, 2H), 6.71 (dd, *J* = 7.2, 6.6 Hz, 1H), 3.86 (s, 3H), 3.47 (s, 2H), 2.18 (s, 6H). ^13^C NMR (151 MHz, DMSO-*d*_6_) δ 166.78, 162.60, 161.87 (d, *J*_C–F_ = 246.0 Hz), 161.83, 161.18, 151.99, 148.02, 144.76, 144.58, 143.89, 138.76, 136.50, 136.30, 135.70, 129.96, 129.58, 129.32 (d, *J*_C–F_ = 8.9 Hz), 129.04, 128.82, 122.23, 120.79, 120.43, 116.06 (d, *J*_C–F_ = 23.0 Hz), 111.91, 110.75, 106.96, 106.25, 62.97, 44.92, 38.78. LRMS (ESI) *m*/*z* 656.2 [M + H]^+^. HRMS (ESI) *m*/*z* for C_37_H_31_FN_7_O_4_ [M + H]^+^, calcd 656.2422, found 656.2427. UPLC purity 94.62% (*t*_R_ = 1.962 min).

#### 1-(4-Fluorophenyl)-*N*-[4-({6-(1-methyl-1*H*-pyrazol-4-yl)-5-[4-(piperazin-1-yl)phenyl]furo[2,3-*d*]pyrimidin-4-yl}oxy)phenyl]-2-oxo-1,2-dihydropyridine-3-carboxamide (**40**)

To a solution of **57l** (99 mg, 0.13 mmol, 1.0 equiv) in dichloromethane (1.3 mL) at 0 °C was added trifluoroacetic acid (240 μL, 3.13 mmol, 24.8 equiv), and then, the reaction mixture was stirred at room temperature. After stirring for 16 h, the reaction mixture was concentrated *in vacuo* and purified by flash chromatography (8% methanol in dichloromethane) to yield the title compound **40** (61 mg, 0.09 mmol, 71%) as a white solid. ^1^H NMR (600 MHz, DMSO-*d*_6_) δ 11.96 (s, 1H), 8.58 (dd, *J* = 7.2, 2.4 Hz, 1H), 8.44 (s, 1H), 8.10 (dd, *J* = 6.6, 2.4 Hz, 1H), 8.05 (s, 1H), 7.73 (d, *J* = 9.0 Hz, 2H), 7.60 (dd, *J* = 9.0, 4.8 Hz, 2H), 7.51 (s, 1H), 7.48 (d, *J* = 8.7 Hz, 2H), 7.42 (dd, *J* = 9.0, 8.4 Hz, 2H), 7.19 (d, *J* = 9.0 Hz, 2H), 7.01 (d, *J* = 8.7 Hz, 2H), 6.71 (dd, *J* = 7.2, 6.6 Hz, 1H), 3.86 (s, 3H), 3.16 (s, 4H), 2.88 (s, 4H). ^13^C NMR (101 MHz, DMSO-*d*_6_) δ 166.76, 162.72, 161.88 (d, *J*_C–F_ = 246.9 Hz), 161.85, 161.19, 151.83, 151.00, 148.09, 144.77, 144.11, 143.90, 136.54, 136.31 (d, *J*_C–F_ = 3.1 Hz), 135.73, 130.80, 129.346, 129.342 (d, *J*_C–F_ = 9.0 Hz), 122.41, 120.81, 120.43, 119.67, 116.09 (d, *J*_C–F_ = 23.1 Hz), 114.43, 112.15, 111.03, 106.98, 106.32, 48.30, 45.38, 38.78. LRMS (ESI) *m*/*z* 683.3 [M + H]^+^. HRMS (ESI) *m*/*z* for C_38_H_32_FN_8_O_4_ [M + H]^+^, calcd 683.2531, found 683.2543. HPLC purity 98.08% (*t*_R_ = 16.07 min).

#### 1-(4-Fluorophenyl)-*N*-[4-({5-[4-(4-methylpiperazin-1-yl)phenyl]-6-(1-methyl-1*H*-pyrazol-4-yl)furo[2,3-*d*]pyrimidin-4-yl}oxy)phenyl]-2-oxo-1,2-dihydropyridine-3-carboxamide (**41**)

To a solution of **56** (100 mg, 0.17 mmol, 1.0 equiv) in *N*,*N*-dimethylformamide (2.8 mL) and tetrahydrofuran (2.8 mL) were added 1-methyl-4-[4-(4,4,5,5-tetramethyl-1,3,2-dioxaborolan-2-yl)phenyl]piperazine (75 mg, 0.25 mmol, 1.5 equiv), Pd(dppf)Cl_2_ (36 mg, 0.05 mmol, 30 mol %), and 2 M Na_2_CO_3(aq)_ (0.3 mL, 4.1 equiv). The reaction mixture was degassed for 30 min, refilled with Argon_(g)_, and stirred at 100 °C. After stirring for 16 h, the reaction mixture was cooled down to room temperature, filtered through Celite, added with water (10 mL), and extracted into CH_2_Cl_2_ (10 mL × 3). The combined organic layers were washed with brine, dried over Na_2_SO_4_, concentrated *in vacuo*, and purified by flash chromatography (6–8% methanol in dichloromethane) to yield the title compound **41** (20 mg, 0.03 mmol, 17%) as a brown solid. ^1^H NMR (600 MHz, DMSO-*d*_6_) δ 11.95 (s, 1H), 8.58 (dd, *J* = 7.2, 2.4 Hz, 1H), 8.44 (s, 1H), 8.10 (dd, *J* = 6.6, 2.4 Hz, 1H), 8.05 (s, 1H), 7.73 (d, *J* = 9.0 Hz, 2H), 7.61 (dd, *J* = 8.7, 4.8 Hz, 2H), 7.52 (s, 1H), 7.48 (d, *J* = 8.4 Hz, 2H), 7.42 (dd, *J* = 9.0, 8.7 Hz, 2H), 7.20 (d, *J* = 9.0 Hz, 2H), 7.03 (d, *J* = 8.4 Hz, 2H), 6.72 (dd, *J* = 7.2, 6.6 Hz, 1H), 3.87 (s, 3H), 3.21 (s, 4H), 2.46 (s, 4H). 2.23 (s, 3H). ^13^C NMR (151 MHz, DMSO-*d*_6_) δ 166.73, 162.69, 161.84 (d, *J*_C–F_ = 246.0 Hz), 161.80, 161.15, 151.80, 150.44, 148.08, 144.72, 144.11, 143.86, 136.52, 136.27 (d, *J*_C–F_ = 2.4 Hz), 135.67, 130.75, 129.32, 129.29 (d, *J*_C–F_ = 8.8 Hz), 122.34, 120.78, 120.43, 119.73, 116.02 (d, *J*_C–F_ = 23.1 Hz), 114.46, 112.12, 110.97, 106.91, 106.31, 54.53, 47.25, 40.04, 38.73. LRMS (ESI) *m*/*z* 697.3 [M + H]^+^. HRMS (ESI) *m*/*z* for C_39_H_34_FN_8_O_4_ [M + H]^+^, calcd 697.2687, found 697.2688. HPLC purity 91.88% (*t*_R_ = 16.59 min).

### Computational Studies

The protein structure of MER (PDB: 7AAY) was utilized in this study which was downloaded from RCSB Protein Data Bank (PDB).^35^ All docking analyses were conducted using the Discovery Studio 2021//LigandFit program (BIOVIA Inc., San Diego, CA, USA) with the CHARMm force field through Align and Superimpose Proteins, CDOCKER, and Flexible Docking protocols with default parameters.^39^ The number of docking poses was set as 10 with default parameters. The decision of the best pose was according to the lowest binding energy of the compound as well as the nitrogen atom of the compound forming a hydrogen bond with the backbone amide nitrogen of Met274 in MER. This was imposed based on the observation of hydrogen bonds in the cocrystal structures of the MET kinase complexed to merestinib (PDB: 7AAY). The docking results were shown as the cartoon model processed by PyMoL and Discovery Studio 2021.^40,41^

### MER and AXL Enzyme Inhibition Assays

A purified kinase (MER or AXL) was incubated with a compound or DMSO (control) for 15 min in an assay buffer (25 mM Tris pH 7.4, 10 mM MgCl_2_, 4 mM MnCl_2_, 2 mM DTT, 0.01% BSA, 0.02% TritonX-100, 0.01% Brij35 and 0.5 mM Na_3_VO_4_ for MER; and 40 mM Tris pH 7.4, 20 mM MgCl_2_, 2 mM DTT, 0.01% BSA and 0.5 mM Na_3_VO_4_ for AXL). The above-prepared substrate and ATP (12 μM for MER; 50 μM for AXL) were added. The mixture was incubated for 3 h at 30 °C. The luminescence was calculated to determine the kinase activity using a Kinase Glo assay kit for MER and an ADP-Glo Kinase Assay kit for AXL following the manufacturer’s instructions (Promega Corp., Madison, Wisconsin).

### Cell Culture

The Ba/F3-TMEM-MerTK cells were maintained in RPMI 1640 (Gibco, New York, NY, USA). The culture media were supplemented with 10% heat-inactivated fetal bovine serum (FBS) and 1% penicillin and streptomycin (HyClone, Logan, UT, USA). The cells were maintained at 37 °C in an incubator (Thermo Fisher Scientific, New York, NY, USA) with an atmosphere of 5% CO_2_.

### Cell Viability Assay

Ba/F3-TMEM-MerTK cells, which overexpressed MER, were seeded in 96-well clear plates at a density of 8 × 10^3^ cells per well overnight. Then, cells were treated with indicated concentrations of test compounds for 72 h. At the end of incubation, for a 96-well microtiter plate, MTS Mix reagent containing culture medium, MTS (tetrazolium compound [3-(4,5-dimethylthiazol-2-yl)-5-(3-carboxymethoxyphenyl)-2-(4-sulfophenyl)-2*H*-tetrazolium, inner salt]; Promega, Madison, WI, USA), and PMS (phenazine methosulfate; Sigma, St. Louis, MO, USA) in a ratio of 8:2:0.1, respectively. The medium in the well was removed, and the MTS Mix reagent was then added to cells (100 μL/well). The plates were incubated for 1.5 h at 37 °C in a humidified 5% CO_2_ atmosphere, and the absorbance was then recorded at 490 nm by a Victor2 plate reader (PerkinElmer, Ramsey, MN, USA).

### Western Blotting Analysis

To measure the effect of **33** on AXL phosphorylation, NCI-H1299 nonsmall cell lung cancer cells were plated at 4 × 10^5^ cells/well in 6 well plates and incubated at 37 °C with 5% CO_2_ for 3 to 4 h and then switched to serum-free culture medium overnight. The next day, diluted **33** was added to the cells and incubated for 1 h. Cells were then stimulated with recombinant human Gas6 (400 ng/mL final concentration) for 30 min. For phosphorylated MER measurement, human melanoma G361 cells were seeded 2 × 10^6^ cell/well in 6 well plates for 2 days followed by serum-free culture medium at 37 °C with 5% CO_2_ overnight. Diluted **33** was then added and incubated for another 48 h followed by stimulation with MER-agonist antibody MAB8912 (500 ng/mL final concentration) for 30 min. The treated and untreated cells were incubated in PBS with 2 mM Na_3_VO_4_ for 5 min on ice and washed with PBS. Cells were lysed in RIPA Lysis and Extraction (Thermo Scientific, Waltham, MA, USA) with 2 mM sodium orthovanadate, 1xHalt Phosphatase Inhibitor Cocktail, and 1xHalt Protease Inhibitor Cocktail. Protein lysates were resolved in SDS-PAGE and then transferred onto apolyvinylidene difluoride membrane (Millipore, Bedford, MA, USA). Membranes were immunoblotted with appropriate antibodies and reacted with the SuperSignal West Pico PLUS Chemiluminescent Substrate (Thermo Scientific, Waltham, MA, USA), followed by exposure to X-ray film. The sources of primary antibodies were as follows: antiphospho-AXL (Tyr702) and anti-AXL (C89E7) were procured from Cell Signaling Technology; antiphospho-MER proto-oncogene tyrosine kinase (MER) (Y749 + Y753 + Y754) and anti-MERTK (Y323) were from Abcam. β-actin antibody was purchased from Thermo Fisher, MA5-15739. The secondary antibody horseradish peroxidase (HRP)-linked goat antirabbit IgG (111-035-003) was purchased from Cell Signaling Technology, human Gas6 from Abcam and MER agonist antibody MAB8912 from R&D System. Proteins were detected using the SuperSignal reagent (Pierce, Rockford, IL) followed by exposure to the X-ray film.

### *In Vivo* Pharmacokinetics Study

The animal studies were performed according to NHRI institutional animal care and committee-approved procedures. Male ICR mice (25–35 g) were obtained from BioLASCO (Taiwan Co., Ltd., Ilan, Taiwan). A single 2.0, 3.0, or 10 mg/kg dose of the compounds, as a PEG400/DMA (80/20, v/v) for both iv and oral dosing or DMSO/CrEL/D5W (5%/5%/90%,v/v/v)(iv) and 1%CMC+0.5%Tween80 (oral) solution, was separately administered to mice. At 0 (before dosing), 0.5, 1, 2, 4, 6, 8, 16, and 24 h after dosing, a blood sample was collected from groups of three mice at each time point by cardiac puncture and plasma was separated from the blood by centrifugation and stored in a freezer (−70 °C) before analysis. All samples were analyzed for the parent drug by LC-MS/MS. Plasma concentration data were analyzed with a noncompartmental method.

### *In Vitro* Microsomal Stability Assay

The potent compounds (1 μM) were incubated with mouse liver microsomes to perform the metabolic stability study. All incubations were initiated with the addition of NADPH-generating system at 37 °C for 30 min. At 0 and the end of incubation, 100 μL of aliquots were taken from the incubation mixture and placed into centrifuge tubes containing 100 μL of ice-cold acetonitrile to terminate the metabolic reaction. The samples were vortexed and centrifuged, and then, the supernatant injected onto LC/MS. Percent of remaining of each compound was calculated by comparing peak areas at 30 min to that at the initiation of incubation.

### Animal Studies

Female C7BL/6 mice were used for MC38 murine colon cancer cells and Hepa1–6 murine liver cancer cells, Balb/c mice for 4T1 murine triple-negative breast cancer cells, and NOD/SCID mice for MDA-MB-231 human triple-negative breast cancer cells. All mice were used between 6 and 7 weeks of age. MC38 cells and Hepa1–6 cells were prepared at 10^5^ and 10^6^ cells, respectively, per mouse in 100 μL of culture medium and implanted subcutaneously into the left flank region of mice with a 25–5/8 gauge needle. 4T1 was prepared at 1 million cells and MDA-MB-231 was prepared at 5 million cells in 1:1 matrigel and culture medium. For the 4T1 tumor model, cells were injected into the fourth left mammary fat pad in 50 μL per mouse. For the MDA-MB-231 xenograft tumor model, tumor cells were either implanted subcutaneously or into mammary fat pad as described in the text. Tumor cells were detected as free of Mycoplasma spp prior to injection into animals. Treatment was initiated after randomization when the average tumor size reached approximately 50–60 mm^3^ for MC38 and 4T1 and 150 to 200 mm^3^ for Hepa1–6 and MDA-MB-231. Animals received vehicle control, 50 mg/kg twice a day (BID) of **22** or **33**, or once a day (QD). The reference compound **8** (tamnorzatinib) was given at 50 mg/kg QD. MER-selective compound **1** (UNC2025) and AXL-selective compound **3** (bemcentinib) were dosed at 25 mg/kg BID, alone or in combination. A five-days-on and two-days-off (FOTO) treatment schedule was used; the treatment length for each model was indicated in figure legends. All compounds were freshly prepared daily in 10%DMA/40%PEG400/50% (1%CMC). Tumor growth was measured with an electronic caliper, and volumes were calculated as *L* × *W* × *W*/2. Tumor size and animal body weight were measured once a week after tumor cell inoculation. Tumor growth inhibition (% TGI) = [1 – Δ*T*/Δ*C*] × 100, where Δ*T* is the difference of average tumor volume on the measured day and day 0 of treated groups and Δ*C* is the difference of average tumor volume on the measured day and day 0 of control groups. The uses and experimental procedures in animals were approved by the Institutional Animal Care and Use Committees (IACUCs) of the National Health Research Institutes. All animals received humane care according to the criteria outlined in the “Guide for the Care and Use of Laboratory Animals“.

### Flow-Cytometry Analysis

MC38 tumor-bearing mice were treated with 50 mg/kg of **22** BID and FOTO for 2 weeks. Tumor and spleen tissues from the control and treated animals were harvested 2 h after the last dose (day 11). Non-necrotic tissues were carefully removed from the tumors followed by digestion with 0.1% collagenase III in DMEM medium for 30 min at 37 °C with agitation every 10 min. The digestion was terminated by adding 10% FBS containing PBS. The single-cell suspension was filtered through a 40 μm filter after red blood cell lysis. Aliquots of the cell suspension were preincubated with the mouse Fc receptor blocker for 10 min before staining with appropriate antimouse antibody conjugate for Cd45, Cd3, Cd4, Cd8, Cd11b, Cd86, F4/80, AXL, and MER (all antibody conjugates from BioLegend) at 4 °C for 30 min followed by two washes in 1% BSA containing buffer and analyzed by flow cytometry to quantify the accumulation of infiltrating immune cells into the tumor. For analysis of Cd206, the cells were fixed using the Cyto X/Cytoperm kit (BD Biosciences) after surface marker staining and then stained with antimouse Cd206 antibody.

### Statistical Analysis

Data are expressed as mean ± standard error of the mean (SEM). Differences in mean values between groups were analyzed through a nonparametric *t* test. One-way analysis of variance (ANOVA) test, followed by Bonferroni post-test comparison, was employed for multiple comparison analysis. A *p* value of <0.05 indicated significant differences. GraphPad Prism 9 was used for conducting the statistical analysis.

## Supporting Information Available

SMILES string for compounds **11**–**41** (CSV)
7AAY_**17** (docking pose of **17** in MER) (PDB)
^1^H NMR, ^13^C NMR, HPLC purity, and UPLC purity spectra of final compounds **11**–**41**, synthesis procedure and compound characterization data for intermediate compounds **60**–**64**, **66**, **67**, and **69**–**72**, mice body weight change during drug treatment, and kinase profiling data of **33** (PDF)

## Supplementary Material

jm4c00400_si_001.csv

jm4c00400_si_002.pdb

## Abbreviations Used

AML: acute myeloid leukemia
APCs: antigen-presenting cells
CLL: chronic lymphocytic leukemia
CMC: carboxymethyl cellulose
CrEL: cremophor EL
D5W: 5% dextrose in water
DIPEA: *N*,*N*-diisopropylethylamine
DM: double mutant
DMEM: Dulbecco’s modified eagle medium
EDCI: 1-ethyl-3-(3-(dimethylamino)propyl)carbodiimide
EMT: epithelial-to-mesenchymal transition
FBS: fetal bovine serum
FLT1: FMS-like tyrosine kinase 1
FLT3: FMS-like tyrosine kinase 3
FLT4: FMS-like tyrosine kinase 4
FNIII: fibronectin type III
FOTO: five-days-on and two-days-off
HNSCC: head and neck squamous cell carcinoma
IgL: immunoglobulin-like
LRMS: low-resolution mass spectra
NA: not available
ND: not detected/determined
ng: nanogram
NHRI: National Health Research Institutes
nM: nanomolar
PMS: phenazine methosulfate
RIPA: radio-immunoprecipitation assay
RTKs: receptor tyrosine kinases
SDS-PAGE: sodium dodecyl-sulfate polyacrylamide gel electrophoresis
SEM: 2-(trimethylsilyl)ethoxymethyl/standard error of the mean
SLL: small lymphocytic lymphoma
Sphos: 2-dicyclohexylphosphino-2′,6′-dimethoxybiphenyl
TAM: TYRO3, AXL, and MER
TBTU: 2-(1*H*-benzotriazole-1-yl)-1,1,3,3-tetramethylaminium tetrafluoroborate
TGI: tumor growth inhibition
TNBC: triple-negative breast cancer
UPLC: ultraperformance liquid chromatography
