# Supplementary material for: Discovery of Dual MER/AXL Kinase Inhibitors as Bifunctional Small Molecules for Inhibiting Tumor Growth and Enhancing Tumor Immune Microenvironment
Source: J Med Chem. 2024 Jun 24;67(13):10906–27. doi: 10.1021/acs.jmedchem.4c00400 (PMC11247487; doi:10.1021/acs.jmedchem.4c00400)
Supplement: Supplementary file 3 — jm4c00400_si_003.pdf [file jm4c00400_si_003.pdf]

<u>**Supporting Information**</u>

Discovery of Dual MER/AXL Kinase Inhibitors as Bifunctional Small Molecules for Inhibiting Tumor Growth and Enhancing Tumor Immune Microenvironment

Mu-Chun Li,[a,b] You-Liang Lai,[a] Po-Hsien Kuo,[a] Julakanti Satyanarayana Reddy,[a] Chih-Ming Chen,[a] Julakanti Manimala,[a] Pei-Chen Wang,[a] Ming-Shiem Wu,[a] Chun-Yu Chang,[a] Chen-Ming Yang,[a] Chin-Yu Lin,[a] Yu-Chen Huang,[a] Chun-Hsien Chiu,[a] Ling Chang,[a] Wen-Hsing Lin,[a] Teng-Kuang Yeh,[a]* Wan-Ching Yen,[a]* Hsing-Pang Hsieh[a,b,c]*

[a]Institute of Biotechnology and Pharmaceutical Research, National Health Research Institutes, Miaoli County 350401, Taiwan, ROC.
[b]Biomedical Translation Research Center (BioTReC), Academia Sinica, Taipei City 115202, Taiwan, ROC.
[c]Department of Chemistry, National Tsing Hua University, Hsinchu City 300044, Taiwan, ROC.

Corresponding authors:
Prof. Hsing-Pang Hsieh
E-mail: hphsieh@nhri.edu.tw
Phone: +886-37-206-166 ext. 35700

Dr. Wan-Ching Yen
E-mail: jeanwcyen@nhri.edu.tw
Phone: +886-37-206-166 ext. 35746

Dr. Teng-Kuang Yeh
E-mail: tkyeh@nhri.edu.tw
Phone: +886-37-206-166 ext. 35757

Table of Contents

1. Mouse body weight change during *in vivo* efficacy evaluation S2
2. 14-day toxicity examination of **33** in ICR mice S6
3. Experimental procedures and compound characterization data for **42i**–**57l** S8
4. ^{1}H and ^{13}C spectra of compounds **11**–**41** S19
5. Kinase profiling data for **33** S50
6. HPLC trace of **22** and **33** S60

1. Mouse body weight change during *in vivo* efficacy evaluation

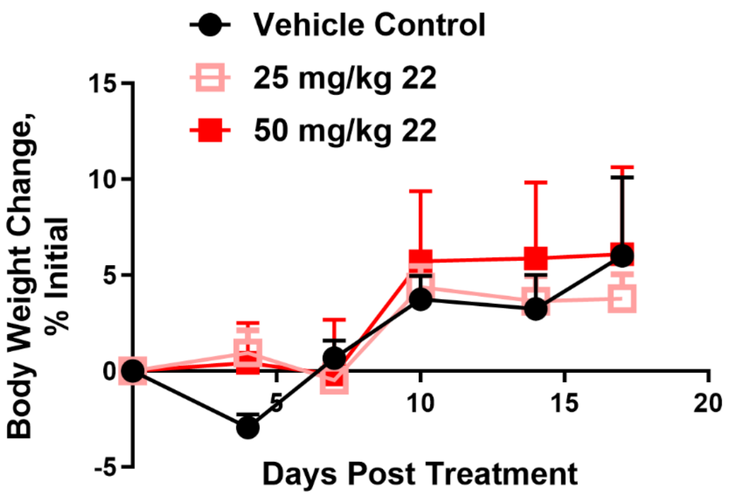

Figure S1. Mouse body weight change during the treatment of **22** in the MC38 syngeneic model.

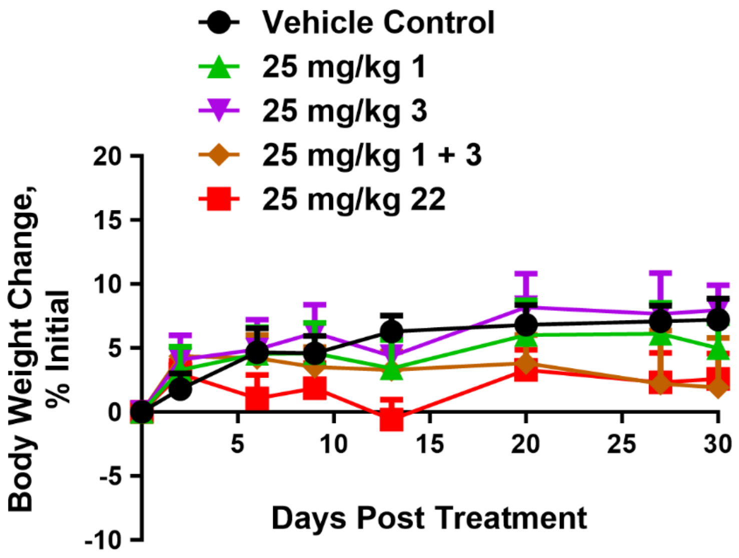

Figure S2. Mouse body weight change during the treatment of **1**, **3**, **1**+**3**, and **22** in the MDA-MB-231 xenograft model.

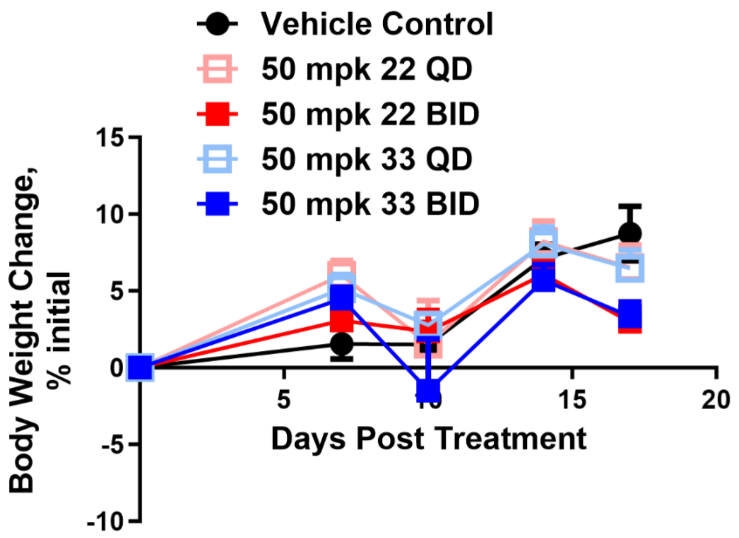

Figure S3. Mouse body weight change during the treatment of **22** and **33** in the MC38 syngeneic model.

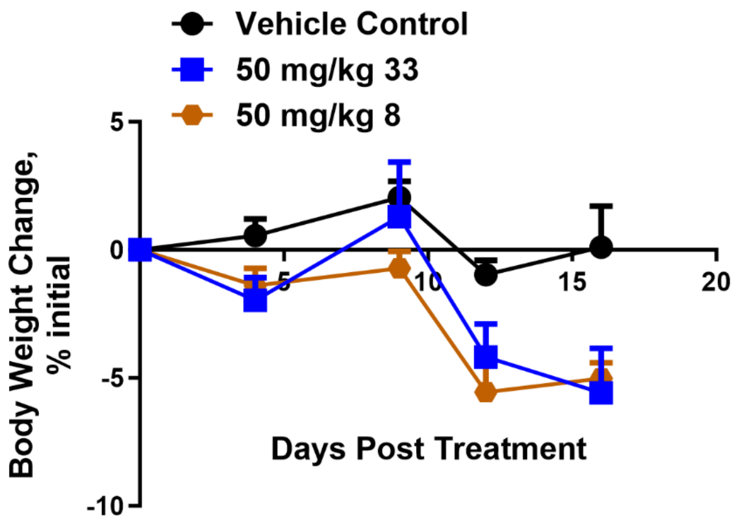

Figure S4. Mouse body weight change during the treatment of **33** and **8** in the 4T1 syngeneic model.

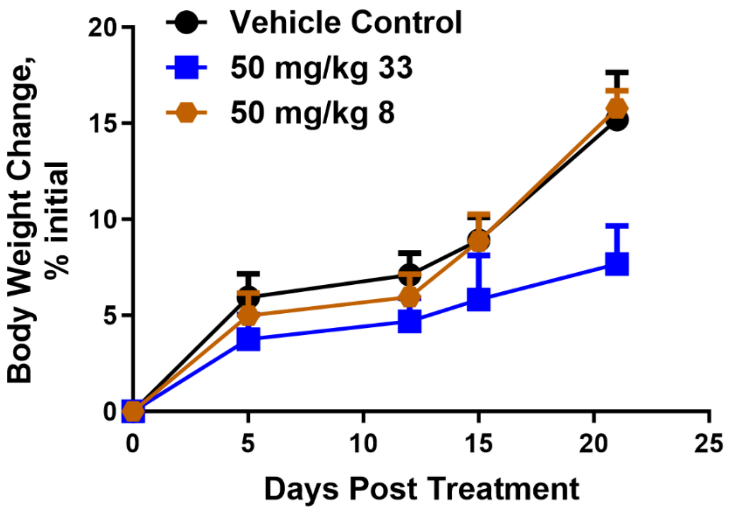

Figure S5. Mouse body weight change during the treatment of **33** and **8** in the MDA-MB-231 xenograft model.

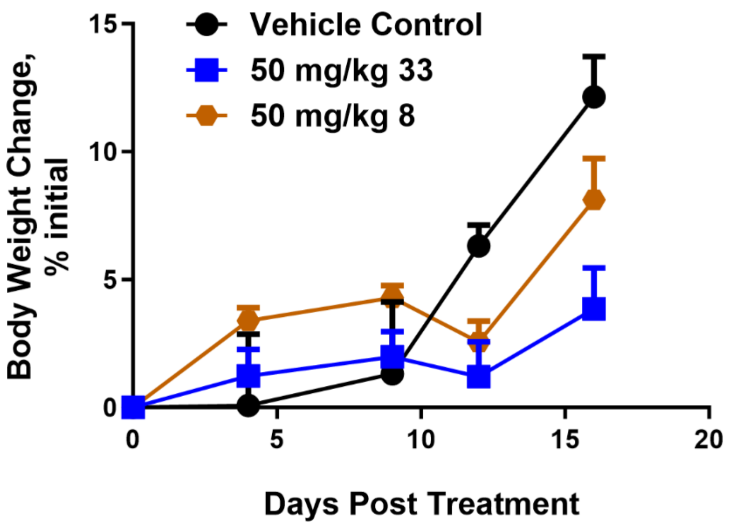

Figure S6. Mouse body weight change during the treatment of **33** and **8** in the MC38 syngeneic model.

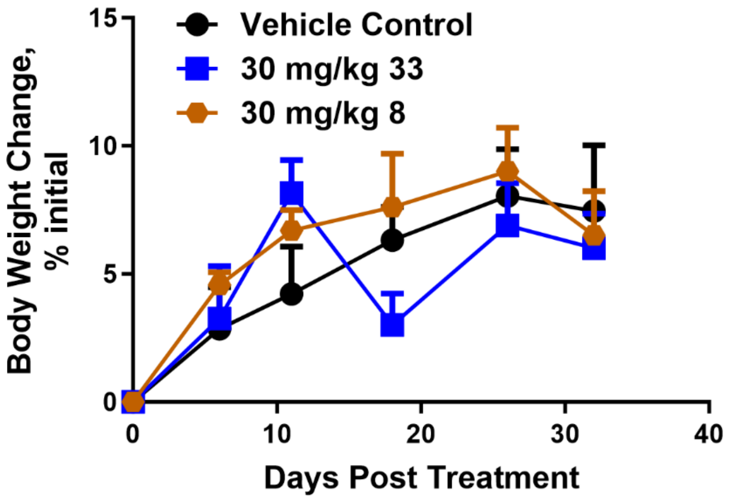

Figure S7. Mouse body weight change during the treatment of **33** and **8** in the Hepa1-6 syngeneic model.

2. 14-day toxicity examination of 33 in ICR mice

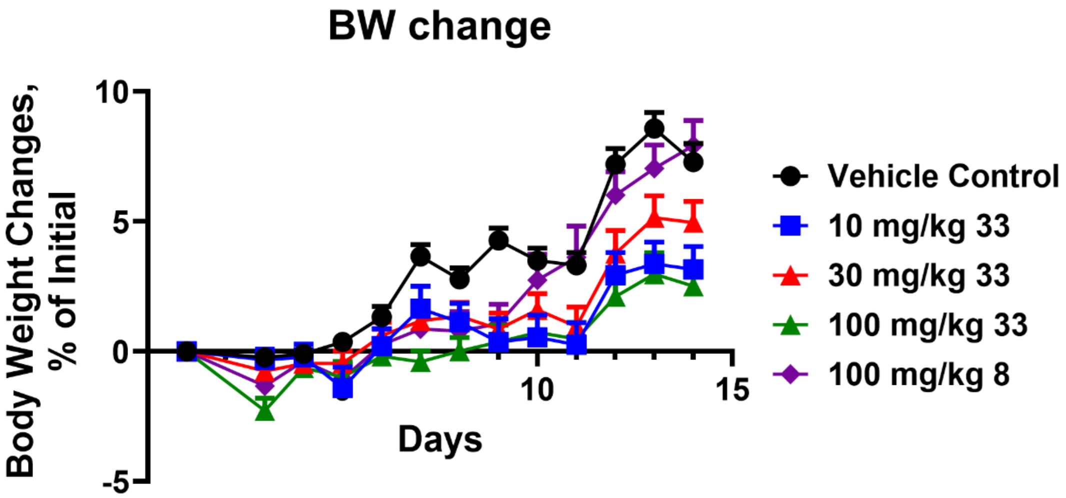

Figure S8. 14-day repeated dose toxicity study of **33** in ICR mice. No effect on body weight.

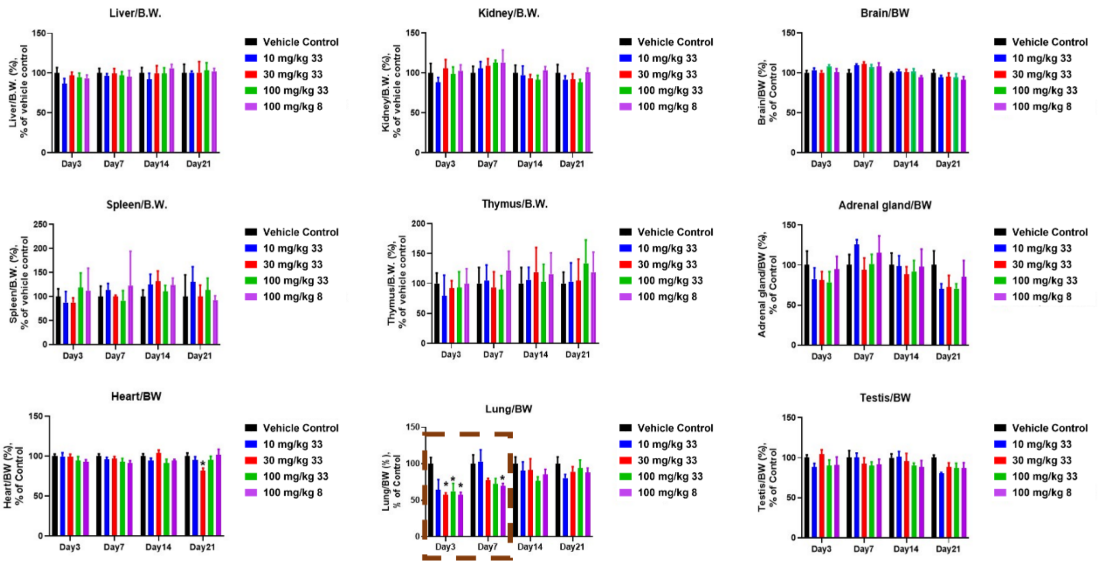

Figure S9. 14-day repeated dose toxicity study of **33** in ICR mice. No significant effects on organ weight.

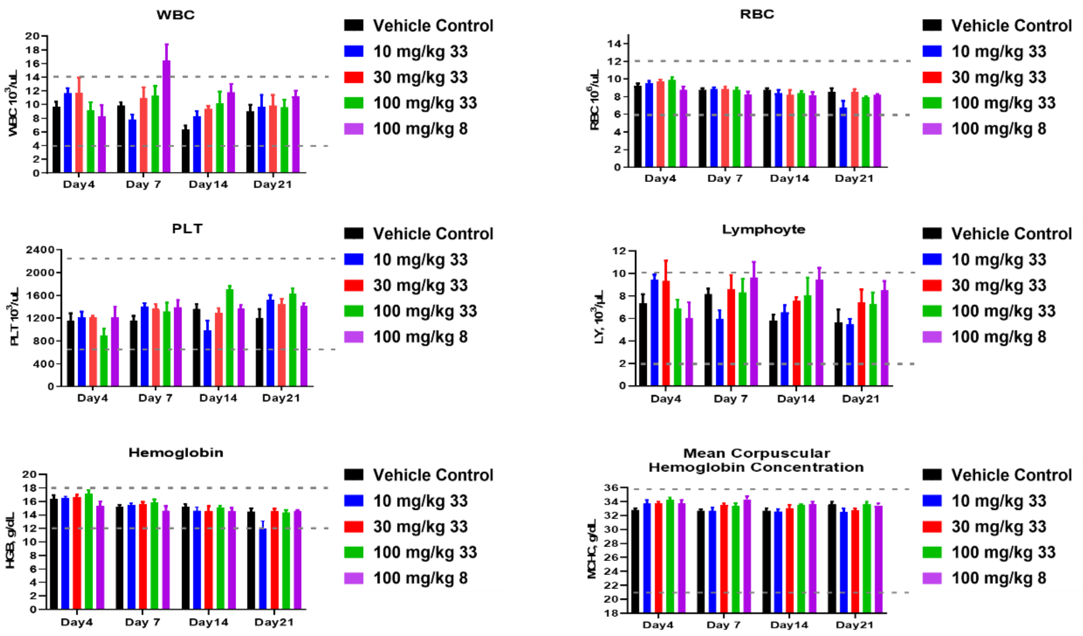

Figure S10. 14-day repeated dose toxicity study of **33** in ICR mice. No significant effects on organ CBC.

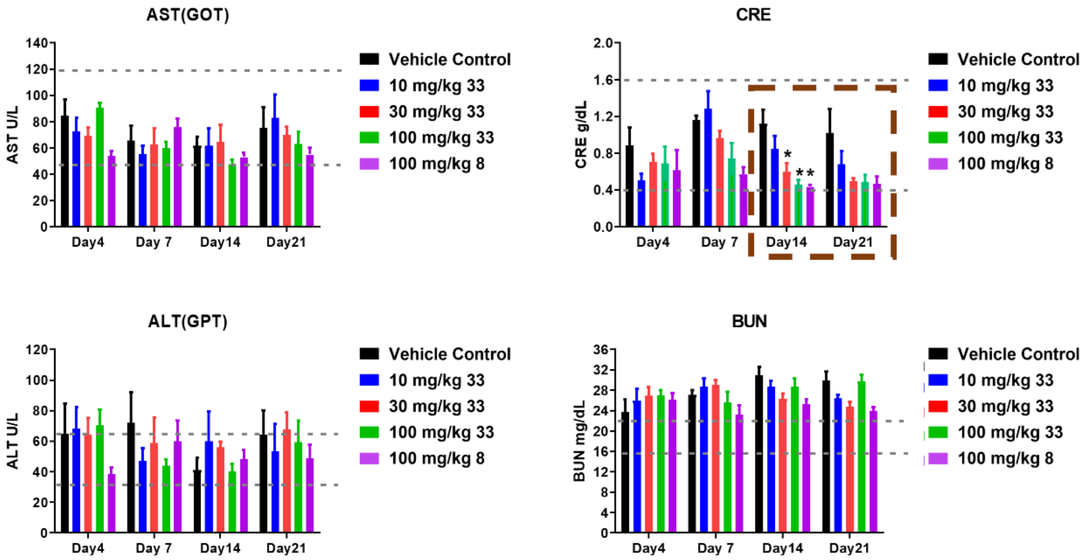

Figure S11. 14-day repeated dose toxicity study of **33** in ICR mice. Mild effect on CRE at 30 and 100 mpk on day 14 and day 21 post treatment.

3. Experimental procedures and compound characterization data for 42i–57l

4-Chloro-5-(3-nitrophenyl)-6-(1-{[2-(trimethylsilyl)ethoxy]methyl}-1*H*-pyrazol-3-yl)furo[2,3-*d*]pyrimidine (42i)

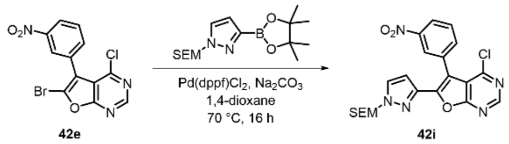

To a solution of **42e** (206 mg, 0.58 mmol, 1.0 equiv.) in 1,4-dioxane (2.0 mL) was added 3-(4,4,5,5-tetramethyl-1,3,2-dioxaborolan-2-yl)-1-{[2-(trimethylsilyl)ethoxy]methyl}-1*H*-pyrazole (282 mg, 0.87 mmol, 1.5 equiv.), Pd(dppf)Cl_2 (42 mg, 0.06 mmol, 10 mol%) and 2M sodium carbonate (1.16 mL, 4.0 equiv.). The reaction mixture was degassed for 30 minutes, refilled with $Argon_{(g)}$ and stirred at 70 °C. After stirring for 16 hours, the reaction mixture was cooled down to room temperature, filtered through Celite, added water (10 mL), and extracted into dichloromethane (10 mL × 3). The combined organic layers were washed with brine, dried over $MgSO_4$, concentrated *in vacuo*, and purified by flash chromatography (17% ethyl acetate in hexane) to yield the title compound **42i** (87 mg, 0.35 mmol, 60%) as mist yellow solid. LRMS (ESI) *m/z*: 472.1 $[M+H]^+$.

***N*[1]-(4-Fluorophenyl)-*N*[1]-(4-{[5-(3-nitrophenyl)-6-phenylfuro[2,3-*d*]pyrimidin-4-yl]oxy}phenyl)cyclopropane-1,1-dicarboxamide (44).**

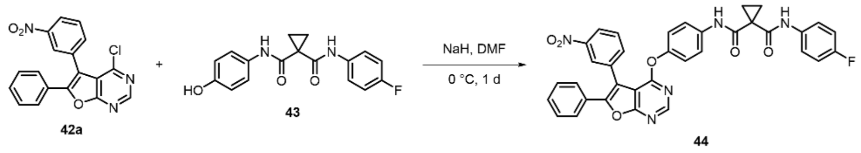

To a solution of sodium hydride (19 mg, 0.48 mmol, 1.4 equiv.) in DMF (5.6 mL) at 0 °C was added a solution of *N*[1]-(4-fluorophenyl)-*N*[1]-(4-hydroxyphenyl)cyclopropane-1,1-dicarboxamide (**43**) (130 mg, 0.41 mmol, 1.2 equiv) in DMF (1 mL) then the reaction mixture was stirred at room temperature. After stirring for 20 minutes, the reaction mixture was cooled down to 0 °C, added 4-chloro-5-(3-nitrophenyl)-6-phenylfuro[2,3-*d*]pyrimidine (**42a**) (117 mg, 0.33 mmol, 1.0 equiv) then stirred at room temperature. After stirring for 24 hours, the resulting precipitate was collected and washed with DCM to yield the title compound **44** (223 mg, 0.35 mmol, > 99%) as a white solid without further purification. LRMS (ESI) *m/z*: 630.2 $[M+H]^+$.

***N*[1]-{[5-(3-Nitrophenyl)-6-phenylfuro[2,3-*d*]pyrimidin-4-yl]benzene-1,4-diamine (45a)**

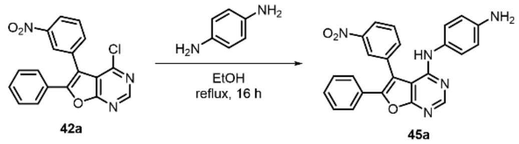

To a solution of 4-chloro-5-(3-nitrophenyl)-6-phenylfuro[2,3-*d*]pyrimidine (**42a**) (270 mg, 0.77 mmol, 1.0 equiv.) in ethanol (3.8 mL) was added benzene-1,4-diamine (166 mg, 1.54 mmol, 2.0 equiv) then the reaction mixture was stirred at reflux. After stirring for 16 hours, the reaction mixture was cooled down to room temperature and added H_2O (5 mL). The resulting precipitate was collected, washed with DCM (10 mL), and concentrated *in vacuo* to yield the title compound **45a** (290 mg, 0.68 mmol, 89%) as a white solid without further purification. LRMS (ESI) *m/z*: 424.1 $[M+H]^+$.

4-{[5-(3-Nitrophenyl)-6-phenylfuro[2,3-*d*]pyrimidin-4-yl]oxy}aniline (46a)

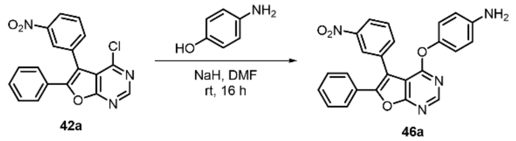

To a solution of sodium hydride (117 mg, 4.43 mmol, 1.5 equiv.) in *N*,*N*-dimethylformamide (59 mL) at 0 °C was added 4-aminophenol (482 mg, 4.42 mmol, 1.5 equiv) then the reaction mixture was stirred at 0 °C. After stirring for 30 minutes, the reaction mixture was added **42a** (1.04 g, 2.96 mmol, 1.0 equiv) and then stirred at room temperature. After stirred for 16 hours, the reaction mixture was filtered

through Celite, and concentrated *in vacuo* to yield the title compound **46a** (1.24 g, 2.92 mmol, 99%) as white solid. LRMS (ESI) *m/z*: 425.1 $[M+H]^+$.

4-{[5-(3-Nitrophenyl)furo[2,3-*d*]pyrimidin-4-yl]oxy}aniline (46d)

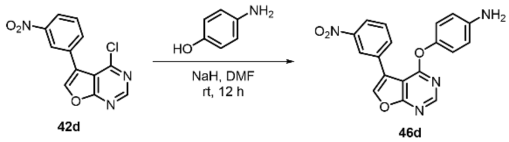

To a solution of sodium hydride (32 mg, 0.80 mmol, 1.1 equiv.) in *N*,*N*-dimethylformamide (1.6 mL) at 0 °C was added 4-aminophenol (79 mg, 4.42 mmol, 1.5 equiv) then the reaction mixture was stirred at 0 °C. After stirring for 30 minutes, the reaction mixture was added a solution of **42d** (200 mg, 0.73 mmol, 1.0 equiv) in THF (3.6 mL) then stirred at room temperature. After stirring for 12 hours, the reaction mixture was quenched with water (10 mL). The resulting precipitate was collected, and the organic layers were combined, dried over $MgSO_4$, concentrated *in vacuo*, and purified by flash chromatography (1% methanol in dichloromethane) to yield the title compound **46d** (200 mg, 0.57 mmol, 79%) as a brown solid. LRMS (ESI) *m/z*: 349.1 $[M+H]^+$.

4-{[6-Bromo-5-(3-nitrophenyl)furo[2,3-*d*]pyrimidin-4-yl]oxy}aniline (46e)

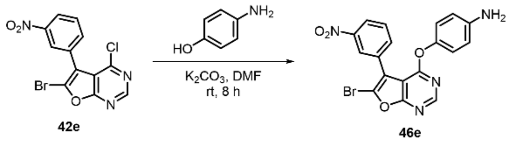

To a solution of potassium carbonate (3.20 g, 23.2 mmol, 2.1 equiv.) in *N*,*N*-dimethylformamide (56 mL) at 0 °C was added 4-aminophenol (1.5 g, 13.7 mmol, 1.2 equiv) then the reaction mixture was stirred at 0 °C. After stirring for 30 minutes, the reaction mixture was added **42e** (4.00 g, 11.3 mmol, 1.0 equiv.) and then stirred at room temperature. After stirring for 8 hours, the reaction mixture was extracted into dichloromethane (10 mL × 3). The combined organic layers were dried over $MgSO_4$, concentrated *in vacuo*, and purified by flash chromatography (2% methanol in dichloromethane) to yield the title compound **46e** (3.60 g, 8.43 mmol, 75%) as a brown solid. LRMS (ESI) *m/z*: 354.1 $[M+H]^+$.

***N*[1]-(4-Fluorophenyl)-*N*[1]-(4-{[5-(3-nitrophenyl)-6-phenylfuro[2,3-*d*]pyrimidin-4-yl]amino}phenyl)cyclopropane-1,1-dicarboxamide (47)**

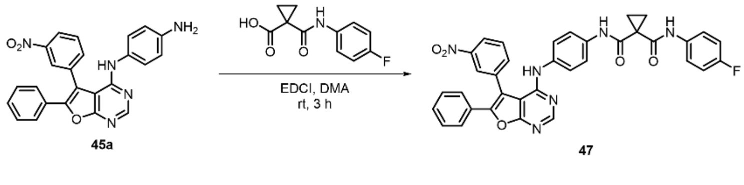

To a solution of **45a** (290 mg, 0.68 mmol, 1.0 equiv.) in dimethylacetamide (1.4 mL) was added 1-[(4-fluorophenyl)carbamoyl]cyclopropanecarboxylic acid (166 mg, 0.82

mmol, 1.2 equiv) and EDCI (160 mg, 0.82 mmol, 1.2 equiv.) then the reaction mixture was stirred at room temperature. After stirring for 3 hours, the reaction mixture was added water (5 mL), and the resulting precipitate was collected and purified by thin-plate chromatography to yield the title compound **47** (85 mg, 0.14 mmol, 20%) as a white solid. LRMS (ESI) *m/z*: 629.1 $[M+H]^+$.

_N_-[(4-Fluorophenyl)acetyl](4-{[5-(3-nitrophenyl)-6-phenylfuro[2,3-_d_]pyrimidin-4-yl]oxy}phenyl)carbamothioic amide (48)

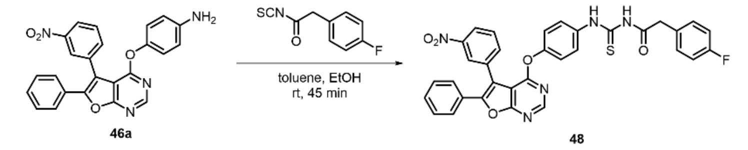

To a solution of **46a** (258 mg, 0.61 mmol, 1.0 equiv.) in toluene (4.6 mL) and ethanol (4.6 mL) was added (4-fluorophenyl)acetyl isothiocyanate (162 mg, 0.83 mmol, 1.4 equiv.) then the reaction was stirred at room temperature. After stirring for 45 minutes, the reaction mixture was concentrated *in vacuo* and purified by flash column chromatography (35% ethyl acetate in hexane) to yield the title compound **48** (256 mg, 0.41 mmol, 68%) as a white solid. LRMS (ESI) *m/z*: 620.1 $[M+H]^+$.

1-(4-Fluorophenyl)-_N_-(4-{[5-(3-nitrophenyl)-6-phenylfuro[2,3-_d_]pyrimidin-4-yl]oxy}phenyl)-2-oxo-1,2-dihydropyridine-3-carboxamide (49a)

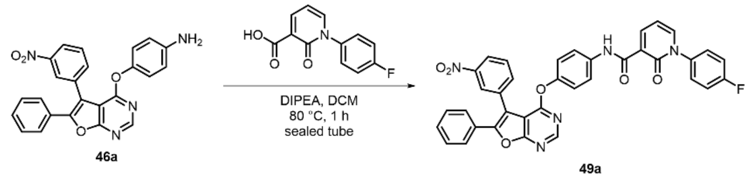

To a solution of 1-(4-fluorophenyl)-2-oxo-1,2-dihydropyridine-3-carboxylic acid (788 mg, 3.38 mmol, 1.5 equiv.) in oxalyl chloride (0.3 mL) then the reaction mixture was stirred at 80 °C. After stirred for 2 hours, the reaction mixture was cooled down to room temperature, added into a solution of **46a** (955 mg, 2.25 mmol, 1.0 equiv.) in dichloromethane (15 mL) at 80 °C then the reaction mixture was stirred at room temperature. After stirred for 1 hour, the reaction mixture was washed with $NaHCO_{3(aq)}$ (10 mL) and brine (10 mL), dried over $MgSO_4$, concentrated *in vacuo*, and purified by flash chromatography (50% ethyl acetate in hexane) to yield the title compound **49a** (400 mg, 0.63 mmol, 28%) as a white solid. LRMS (ESI) *m/z*: 640.2 $[M+H]^+$.

1-(4-Fluorophenyl)-*N*-(4-{[5-(3-nitrophenyl)furo[2,3-*d*]pyrimidin-4-yl]oxy}phenyl)-2-oxo-1,2-dihydropyridine-3-carboxamide (49d)

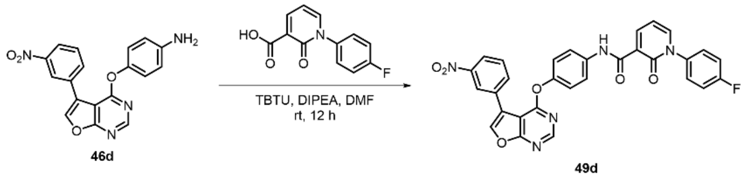

To a solution of 1-(4-fluorophenyl)-2-oxo-1,2-dihydropyridine-3-carboxylic acid (160 mg, 0.69 mmol, 1.2 equiv.) in *N*,*N*-dimethylformamide (9.4 mL) at 0 °C was added TBTU (276 mg, 0.86 mmol, 1.0 equiv.), DIPEA (360 µL, 2.07 mmol, 3.4 equiv.), and **46d** (200 mg, 0.57 mmol, 1.0 equiv.) then the reaction mixture was stirred at 0 °C for 2 hours. After stirring at room temperature for 10 hours, the reaction mixture was quenched with water (20 mL). The resulting precipitate was collected by filtration to yield the title compound **49d** (195 mg, 0.35 mmol, 60%) as a white solid. LRMS (ESI) *m/z*: 564.1 $[M+H]^+$.

***N*-(4-{[6-Bromo-5-(3-nitrophenyl)furo[2,3-*d*]pyrimidin-4-yl]oxy}phenyl)-1-(4-fluorophenyl)-2-oxo-1,2-dihydropyridine-3-carboxamide (49e)**

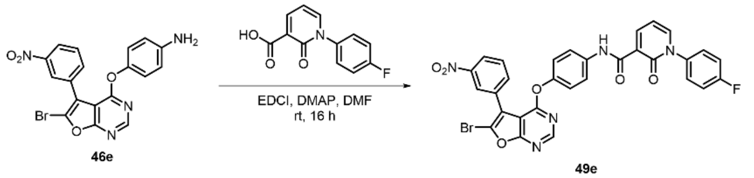

To a solution of **46e** (875 mg, 2.05 mmol, 1.0 equiv.) in *N*,*N*-dimethylformamide (20.5 mL) was added 1-(4-fluorophenyl)-2-oxo-1,2-dihydropyridine-3-carboxylic acid (525 mg, 2.25 mmol, 1.1 equiv.), EDCI (588 mg, 3.07 mmol, 1.5 equiv.), and DMAP (175 mg, 1.43 mmol, 0.7 equiv.) then the reaction mixture was stirred at room temperature. After stirred for 16 hours, the reaction mixture was concentrated *in vacuo*. Then the mixture was dissolved in dichloromethane (20 mL) and extracted with water (10 mL × 3). The combined organic layers were dried over $MgSO_4$, concentrated *in vacuo*, and purified by flash chromatography (1% methanol in dichloromethane) to yield the title compound **49e** (725 mg, 1.13 mmol, 55%) as a brown solid. LRMS (ESI) *m/z*: 642.1 $[M+H]^+$.

1-(4-Fluorophenyl)-*N*-(4-{[5-(3-nitrophenyl)-6-(1*H*-pyrazol-4-yl)furo[2,3-*d*]pyrimidin-4-yl]oxy}phenyl)-2-oxo-1,2-dihydropyridine-3-carboxamide (49f)

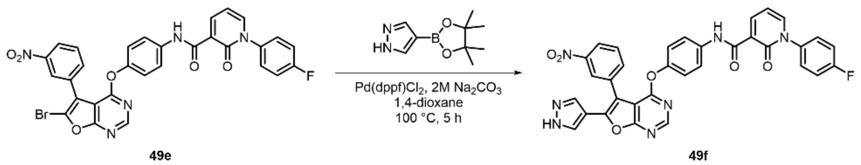

To a solution of **49e** (100 mg, 0.16 mmol, 1.0 equiv.) in 1,4-dioxane (1.5 mL) was added 4-(4,4,5,5-tetramethyl-1,3,2-dioxaborolan-2-yl)-1*H*-pyrazole (36 mg, 0.19 mmol, 1.2 equiv.), Pd(dppf)Cl_2 (23 mg, 0.03 mmol, 20 mol%) and 2M sodium carbonate (156 µL, 1.5 equiv.). The reaction mixture was degassed for 30 minutes, refilled with $Argon_{(g)}$ and stirred at 100 °C. After stirred for 5 hours, the reaction mixture was cooled down to room temperature, filtered through Celite, added water (10 mL), and extracted into dichloromethane (10 mL × 3), The combined organic layers were washed with brine, dried over $MgSO_4$, concentrated *in vacuo*, and purified by flash chromatography (2.5% methanol in dichloromethane) to yield the title compound **49f** (86 mg, 0.14 mmol, 88%) as white solid. LRMS (ESI) *m/z*: 630.2 $[M+H]^+$.

***N*-(4-{[6-(1,3-Dimethyl-1*H*-pyrazol-4-yl)-5-(3-nitrophenyl)furo[2,3-*d*]pyrimidin-4-yl]oxy}phenyl)-1-(4-fluorophenyl)-2-oxo-1,2-dihydropyridine-3-carboxamide (49g)**

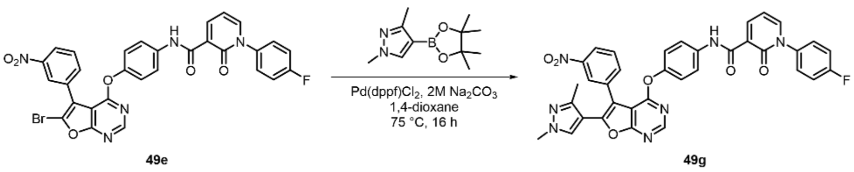

To a solution of **49e** (145 mg, 0.23 mmol, 1.0 equiv.) in 1,4-dioxane (3.0 mL) was added 1,3-dimethyl-4-(4,4,5,5-tetramethyl-1,3,2-dioxaborolan-2-yl)pyrazole (65 mg, 0.29 mmol, 1.3 equiv.), Pd(dppf)Cl_2 (17 mg, 0.02 mmol, 10 mol%) and 2M sodium carbonate (452 µL, 4.0 equiv.). The reaction mixture was degassed for 30 minutes, refilled with $Argon_{(g)}$ and stirred at 75 °C. After stirring for 16 hours, the reaction mixture was cooled down to room temperature, filtered through Celite, added H_2O (10 mL), and extracted into dichloromethane (10 mL × 3). The combined organic layers were washed with brine, dried over $MgSO_4$, concentrated *in vacuo*, and purified by flash chromatography (25% ethyl acetate in dichloromethane) to yield the title compound **49g** (100 mg, 0.15 mmol, 67%) as mist yellow solid. LRMS (ESI) *m/z*: 658.2 $[M+H]^+$.

***N*-(4-{[6-(3,5-Dimethyl-1,2-oxazol-4-yl)-5-(3-nitrophenyl)furo[2,3-*d*]pyrimidin-4-yl]oxy}phenyl)-1-(4-fluorophenyl)-2-oxo-1,2-dihydropyridine-3-carboxamide (49h)**

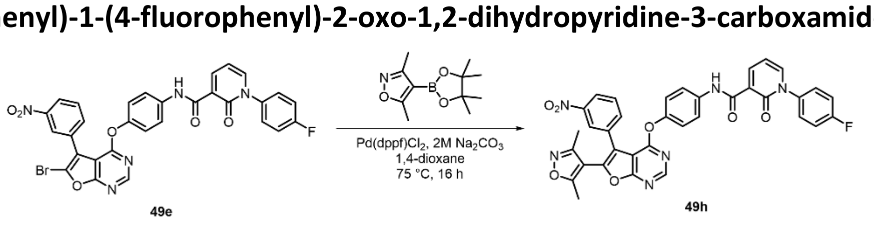

To a solution of **49e** (133 mg, 0.21 mmol, 1.0 equiv.) in 1,4-dioxane (3.5 mL) was added 3,5-dimethyl-4-(4,4,5,5-tetramethyl-1,3,2-dioxaborolan-2-yl)-1,2-oxazole (65 mg, 0.29 mmol, 1.4 equiv.), Pd(dppf)Cl_2 (15 mg, 0.02 mmol, 10 mol%) and 2M

sodium carbonate (414 µL, 4.0 equiv.). The reaction mixture was degassed for 30 minutes, refilled with Argon$_{(g)}$ and stirred at 75 °C. After stirring for 16 hours, the reaction mixture was cooled down to room temperature, filtered through Celite, added water (10 mL), and extracted into dichloromethane (10 mL × 3). The combined organic layers were washed with brine, dried over $MgSO_4$, concentrated *in vacuo*, and purified by flash chromatography (33% ethyl acetate in dichloromethane) to yield the title compound **49h** (87 mg, 0.13 mmol, 64%) as mist yellow solid. LRMS (ESI) *m/z*: 659.2 $[M+H]^+$.

1-(4-Fluorophenyl)-*N*-(4-{[5-(3-nitrophenyl)-6-(1-{[2-(trimethylsilyl)ethoxy]methyl}-1*H*-pyrazol-3-yl)furo[2,3-*d*]pyrimidin-4-yl]oxy}phenyl)-2-oxo-1,2-dihydropyridine-3-carboxamide (49i)

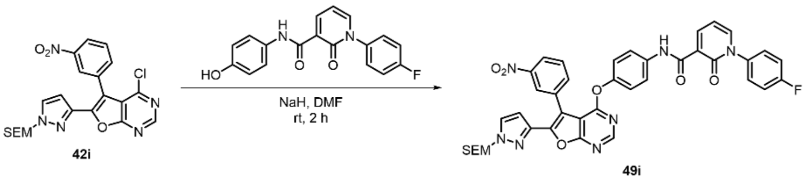

To a solution of sodium hydride (16 mg, 0.40 mmol, 1.1 equiv.) in *N*,*N*-dimethylformamide (1.0 mL) at 0 °C was added a solution of 1-(4-fluorophenyl)-*N*-(4-hydroxyphenyl)-2-oxo-1,2-dihydropyridine-3-carboxamide (126 mg, 0.39 mmol, 1.1 equiv.) then the reaction mixture was stirred at 0 °C. After stirring for 30 minutes, the reaction mixture was added a solution of **42i** (167 mg, 0.35 mmol, 1.0 equiv.) in *N*,*N*-dimethylformamide (2.0 mL) then stirred at room temperature. After stirring for 2 hours, the reaction mixture was quenched with H_2O (10 mL) and extracted into ethyl acetate (10 mL × 3). The combined organic layers were washed with brine (10 mL), dried over $MgSO_4$, concentrated *in vacuo*, and purified by flash chromatography (1% methanol in dichloromethane) to yield the title compound **49i** (208 mg, 0.27 mmol, 77%) as mist yellow solid. LRMS (ESI) *m/z*: 782.2 $[M+H]^+$.

1-(4-Fluorophenyl)-*N*-(4-{[5-(3-nitrophenyl)-6-(thiophen-3-yl)furo[2,3-*d*]pyrimidin-4-yl]oxy}phenyl)-2-oxo-1,2-dihydropyridine-3-carboxamide (49j)

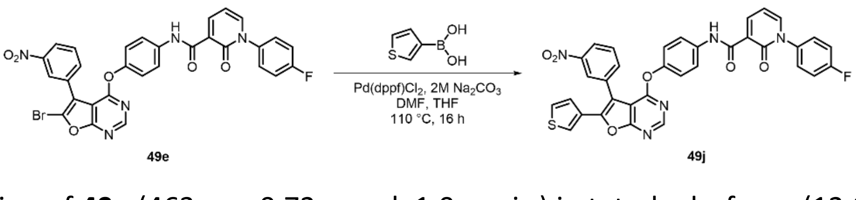

To a solution of **49e** (463 mg, 0.72 mmol, 1.0 equiv.) in tetrahydrofuran (12.0 mL) and *N*,*N*-dimethylformamide (12.0 mL) was added thiophen-3-ylboronic acid (138 mg, 1.08 mmol, 1.5 equiv.), Pd(dppf)Cl_2 (158 mg, 0.22 mmol, 30 mol%) and 2M sodium carbonate (1.5 mL, 4.0 equiv.). The reaction mixture was degassed for 30 minutes, refilled with Argon$_{(g)}$ and stirred at 110 °C. After stirring for 16 hours, the reaction

mixture was cooled down to room temperature, filtered through Celite, added water (10 mL), and extracted into dichloromethane (10 mL × 3). The combined organic layers were washed with brine, dried over Na_2SO_4, concentrated *in vacuo*, and purified by flash chromatography (1% methanol in dichloromethane) to yield the title compound **49j** (375 mg, 0.58 mmol, 81%) as a brown solid. LRMS (ESI) *m/z*: 646.0 $[M+H]^+$.

_N_-(4-{[5-(3-Aminophenyl)-6-(1-{[2-(trimethylsilyl)ethoxy]methyl}-1*H*-pyrazol-3-yl)furo[2,3-*d*]pyrimidin-4-yl]oxy}phenyl)-1-(4-fluorophenyl)-2-oxo-1,2-dihydropyridine-3-carboxamide (50i)

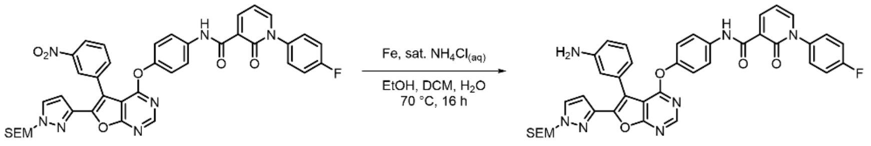

49i 50i

To a solution of **49i** (207 mg, 0.27 mmol, 1.0 equiv.) in ethanol (3.0 mL), dichloromethane (4.5 mL) and water (0.6 mL) was added iron powder (46 mg, 0.82 mmol, 3.0 equiv.) and sat. $NH_4Cl_{(aq)}$ (272 µL) then the reaction mixture was stirred at 70 °C. After stirred for 16 hours, the reaction mixture was cooled down to room temperature, quenched with a solution of 6% NH_4OH in methanol (1 mL), filtered through Celite and concentrated *in vacuo*, and purified by Combiflash automated flash chromatography (2% methanol in dichloromethane) to yield the title compound **50i** (134 mg, 0.18 mmol, 67%) as beige solid. LRMS (ESI) *m/z*: 730.3 $[M+H]^+$.

6-Chloro-5-iodopyrimidin-4-ol (52)

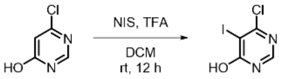

51 52

To a solution of 6-chloropyrimidin-4-ol (**51**) (5.00 g, 38.3 mmol, 1.0 equiv.) in dichloromethane (106.0 mL) at 0 °C was added *N*-iodosuccinimide (9.48 g, 42.1 mmol, 1.1 equiv.) and trifluoroacetic acid (21.3 mL, 278.2 mmol, 7.3 equiv.) dropwise then the reaction mixture was stirred at room temperature. After stirring for 12 hours, the reaction mixture was concentrated *in vacuo*, dissolved in ether, and washed with water (20 mL × 3). The resulting precipitate was collected by filtration to yield the title compound **52** (8.40 g, 32.8 mmol, 86%) as a pink solid. LRMS (ESI) *m/z*: 256.1 $[M+H]^+$.

4-Chloro-6-(1-methyl-1*H*-pyrazol-4-yl)furo[2,3-*d*]pyrimidine (53)

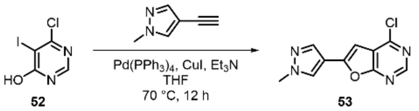

52 53

To a solution of **52** (6.90 g, 26.9 mmol, 1.0 equiv.) in tetrahydrofuran (90.0 mL) was added 4-ethynyl-1-methyl-1*H*-pyrazole (2.85 g, 26.9 mmol, 1.0 equiv.), $Pd(PPh_3)_4$ (1.55 g, 1.34 mmol, 5 mol%), CuI (256 mg, 1.34 mmol, 5 mol%), and triethylamine (11.0 mL, 78.8 mmol, 2.9 equiv.) then the reaction mixture was stirred at 70 °C. After stirring for 12 hours, the reaction mixture was cooled down to room temperature, concentrated *in vacuo*, dissolved in ethyl acetate (20 mL), and washed with water (20 mL × 3). The combined organic layers were washed with brine, dried over $MgSO_4$, concentrated *in vacuo*, and purified by flash chromatography (15–20% ethyl acetate in hexane) to yield the title compound **53** (1.90 g, 8.10 mmol, 30%) as a white solid. LRMS (ESI) *m/z*: 235.1 $[M+H]^+$.

5-Bromo-4-chloro-6-(1-methyl-1*H*-pyrazol-4-yl)furo[2,3-*d*]pyrimidine (54)

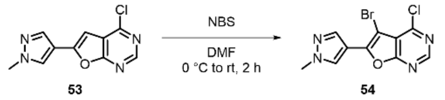

To a solution of **53** (1.90 g, 8.10 mmol, 1.0 equiv.) in *N*,*N*-dimethylformamide (20.0 mL) at 0 °C was added *N*-bromosuccinimide (1.72 g, 9.66 mmol, 1.2 equiv.) then the reaction mixture was stirred at 0 °C. After stirring for 30 minutes, the reaction mixture was warmed up to room temperature. After stirring for a further 2 hours, the reaction mixture was diluted with water (30 mL) slowly. Then the resulting precipitate was collected by filtration to yield **54** (1.85 g, 5.86 mmol, 72%) as a white solid without further purification. LRMS (ESI) *m/z*: 313.0 $[M+H]^+$.

4-{[5-Bromo-6-(1-methyl-1*H*-pyrazol-4-yl)furo[2,3-*d*]pyrimidin-4-yl]oxy}aniline (55)

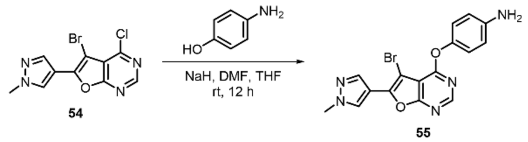

To a solution of sodium hydride (232 mg, 5.80 mmol, 1.1 equiv.) in *N*,*N*-dimethylformamide (10.0 mL) at 0 °C was added a solution of 4-aminophenol (577 mg, 5.29 mmol, 1.0 equiv.) in tetrahydrofuran (10.0 mL) and a solution of **54** (1.65 g, 5.23 mmol, 1.0 equiv.) in *N*,*N*-dimethylformamide (7.6 mL) and tetrahydrofuran (7.6 mL) then the reaction mixture was stirred at room temperature. After stirring for 12 hours, the reaction mixture was quenched with water (10 mL) and stirred for 30 minutes. Then the resulting precipitate was collected by filtration to yield the title compound **55** (1.85 g, 4.8 mmol, 92%) as a brown solid without further purification. LRMS (ESI) *m/z*: 386.1 $[M+H]^+$.

***N*-(4-{[5-Bromo-6-(1-methyl-1*H*-pyrazol-4-yl)furo[2,3-*d*]pyrimidin-4-yl]oxy}phenyl)-1-(4-fluorophenyl)-2-oxo-1,2-dihydropyridine-3-carboxamide (56)**

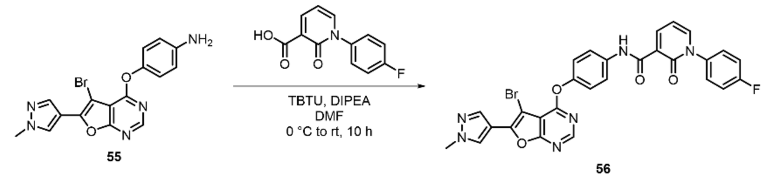

To a solution of 1-(4-fluorophenyl)-2-oxo-1,2-dihydropyridine-3-carboxylic acid (1.45 g, 6.21 mmol, 1.2 equiv.) in *N*,*N*-dimethylformamide (9.4 mL) at 0 °C was added TBTU (2.49 g, 7.75 mmol, 1.5 equiv.), DIPEA (3.24 mL, 18.6 mmol, 3.6 equiv.), and **55** (2.00 g, 5.18 mmol, 1.0 equiv.) then the reaction mixture was stirred at 0 °C for 2 hours. After stirring at room temperature for 10 hours, the reaction mixture was quenched with water (200 mL). The resulting precipitate was collected by filtration to yield the title compound **56** (3.10 g, 5.15 mmol, quant.) as a white solid without further purification. LRMS (ESI) *m/z*: 601.1 $[M+H]^+$.

***tert*-Butyl 4-(3-{4-[4-({[1-(4-fluorophenyl)-2-oxo-1,2-dihydropyridin-3-yl]carbonyl}amino)phenoxy]-6-(1-methyl-1*H*-pyrazol-4-yl)furo[2,3-*d*]pyrimidin-5-yl}phenyl)piperazine-1-carboxylate (57k)**

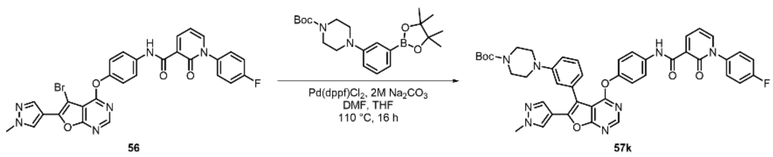

To a solution of **56** (100 mg, 0.17 mmol, 1.0 equiv.) in *N*,*N*-dimethylformamide (2.8 mL) and tetrahydrofuran (2.8 mL) was added *tert*-butyl 4-[3-(4,4,5,5-tetramethyl-1,3,2-dioxaborolan-2-yl)phenyl]piperazine-1-carboxylate (97 mg, 0.25 mmol, 1.5 equiv.), Pd(dppf)Cl_2 (36 mg, 0.05 mmol, 30 mol%) and 2M $Na_2CO_{3(aq)}$ (0.3 mL, 4.0 equiv.). The reaction mixture was degassed for 30 minutes, refilled with $Argon_{(g)}$ and stirred at 110 °C. After stirred for 16 hours, the reaction mixture was cooled down to room temperature, filtered through Celite, added water (10 mL), and extracted into CH_2Cl_2 (10 mL × 3), The combined organic layers were washed with brine, dried over Na_2SO_4, concentrated *in vacuo*, and purified by flash chromatography (2% methanol in dichloromethane) to yield the title compound **57k** (107 mg, 0.14 mmol, 82%) as bronze solid. LRMS (ESI) *m/z*: 783.3 $[M+H]^+$.

tert-Butyl 4-(4-{4-[4-({[1-(4-fluorophenyl)-2-oxo-1,2-dihydropyridin-3-yl]carbonyl}amino)phenoxy]-6-(1-methyl-1_H_-pyrazol-4-yl)furo[2,3-_d_]pyrimidin-5-yl}phenyl)piperazine-1-carboxylate (57l)

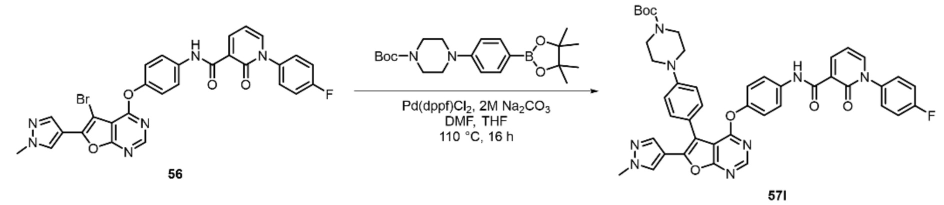

To a solution of **56** (100 mg, 0.17 mmol, 1.0 equiv.) in _N_,_N_-dimethylformamide (2.8 mL) and tetrahydrofuran (2.8 mL) was added _tert_-butyl 4-[4-(4,4,5,5-tetramethyl-1,3,2-dioxaborolan-2-yl)phenyl]piperazine-1-carboxylate (97 mg, 0.25 mmol, 1.5 equiv.), Pd(dppf)Cl_2 (36 mg, 0.05 mmol, 30 mol%) and 2M $Na_2CO_{3(aq)}$ (0.3 mL, 4.0 equiv.). The reaction mixture was degassed for 30 minutes, refilled with $Argon_{(g)}$ and stirred at 110 °C. After stirred for 16 hours, the reaction mixture was cooled down to room temperature, filtered through Celite, added water (10 mL), and extracted into CH_2Cl_2 (10 mL × 3), The combined organic layers were washed with brine, dried over Na_2SO_4, concentrated _in vacuo_, and purified by flash chromatography (3% methanol in dichloromethane) to yield the title compound **57l** (99 mg, 0.13 mmol, 76%) as brown solid. LRMS (ESI) _m/z_: 783.3 $[M+H]^+$.

4. ^{1}H and ^{13}C spectra of compounds 11–41

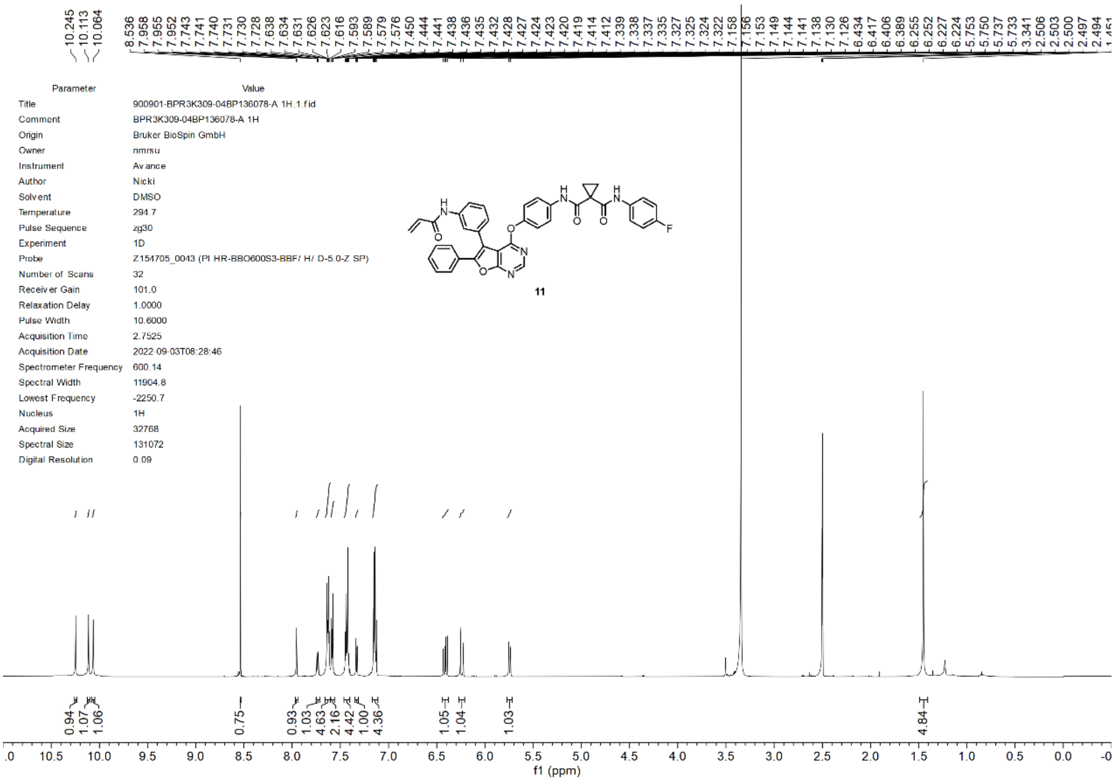

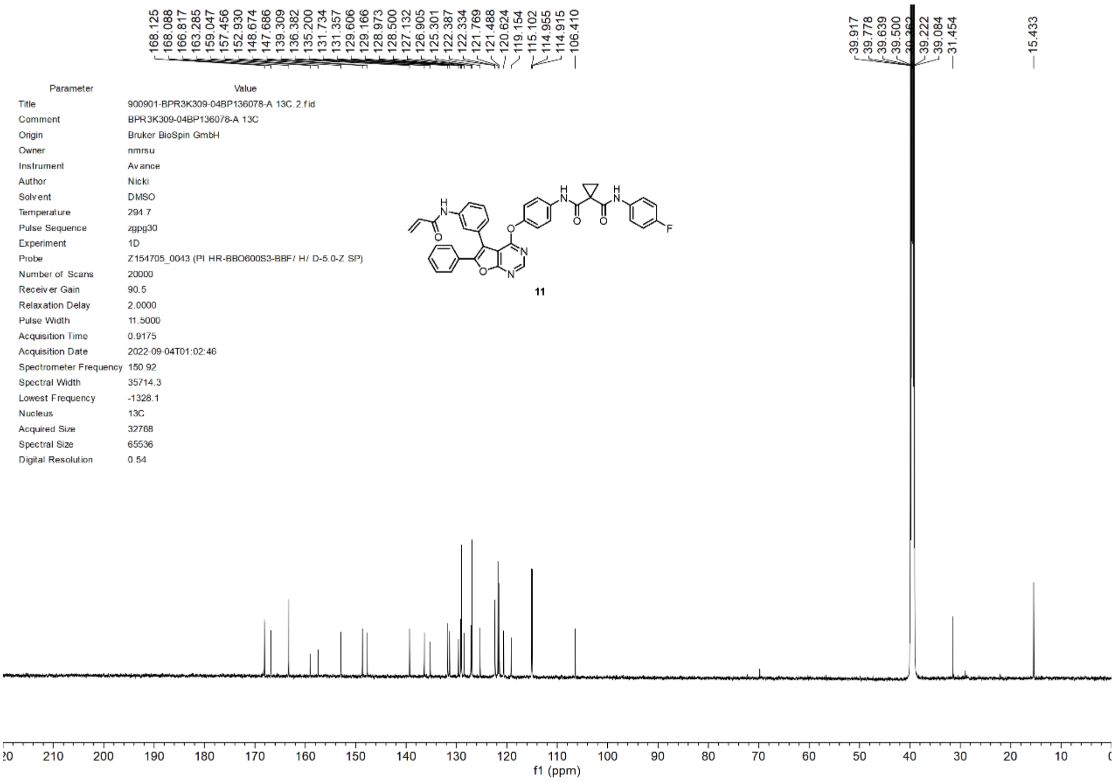

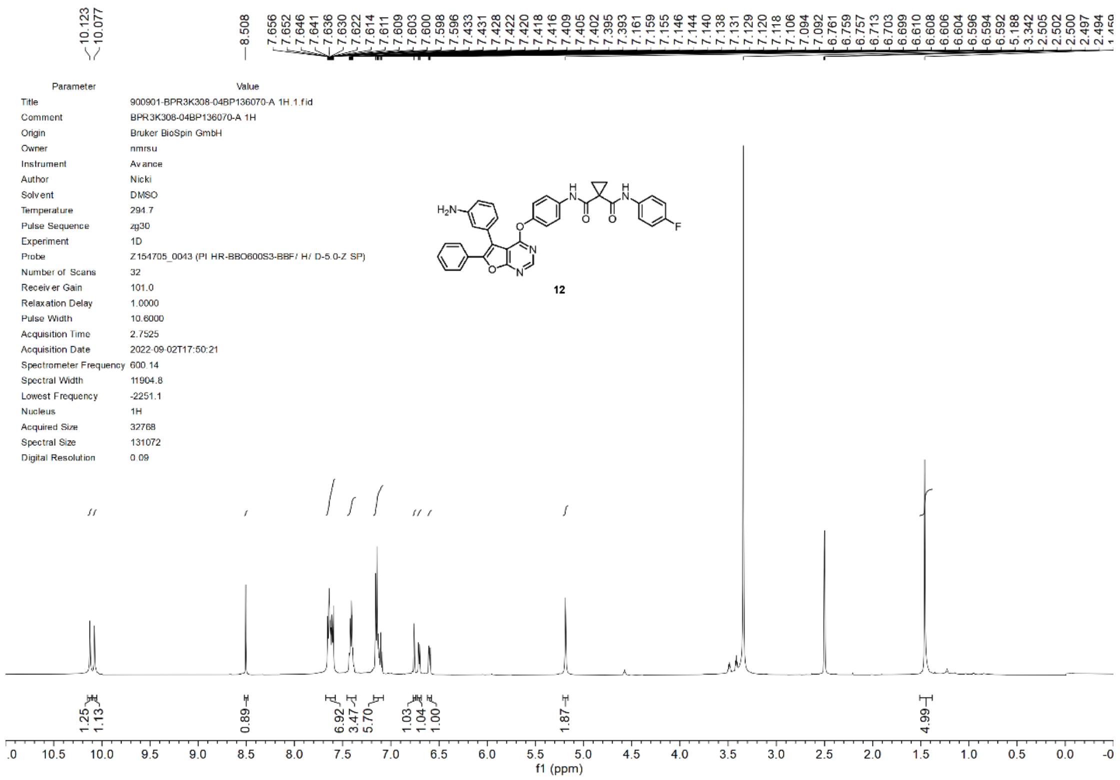
10.123
10.077
8.508
7.656
7.652
7.646
7.641
7.636
7.630
7.622
7.614
7.611
7.609
7.603
7.600
7.598
7.596
7.433
7.431
7.428
7.422
7.420
7.418
7.416
7.409
7.405
7.402
7.395
7.393
7.161
7.159
7.155
7.146
7.144
7.140
7.138
7.131
7.129
7.120
7.118
7.106
7.094
7.092
6.761
6.759
6.757
6.713
6.703
6.699
6.610
6.608
6.606
6.604
6.596
6.594
6.592
5.188
3.342
2.505
2.502
2.500
2.497
2.494
Parameter Value
Title 900901-BPR3K308-04BP136070-A 1H.1.fid
Comment BPR3K308-04BP136070-A 1H
Origin Bruker BioSpin GmbH
Owner nmrsu
Instrument Avance
Author Nicki
Solvent DMSO
Temperature 294.7
Pulse Sequence zg30
Experiment 1D
Probe Z154705_0043 (PI HR-BBO600S3-BBF/ H/ D-5.0-Z SP)
Number of Scans 32
Receiver Gain 101.0
Relaxation Delay 1.0000
Pulse Width 10.6000
Acquisition Time 2.7525
Acquisition Date 2022-09-02T17:50:21
Spectrometer Frequency 600.14
Spectral Width 11904.8
Lowest Frequency -2251.1
Nucleus 1H
Acquired Size 32768
Spectral Size 131072
Digital Resolution 0.09
H2N
O
N
N
H
N
H
N
O
O
F
12
1.25
1.13
0.89
6.92
3.47
5.70
1.03
1.04
1.00
1.87
4.99
f1 (ppm)

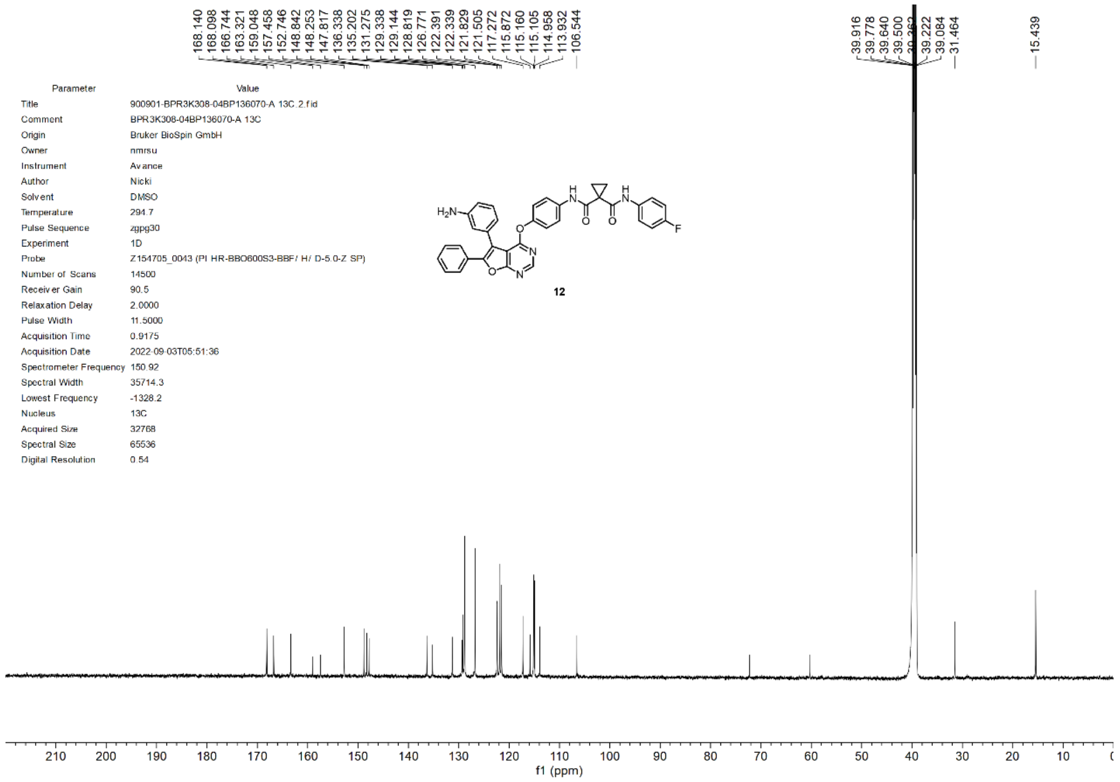
168.140
168.098
166.744
163.321
159.048
157.458
152.746
148.842
148.253
147.817
136.338
135.202
131.275
129.338
129.144
128.819
126.771
122.391
122.339
121.829
121.505
117.272
115.872
115.160
115.105
114.958
113.932
106.544
39.916
39.778
39.640
39.500
39.222
39.084
31.464
15.439
Parameter Value
Title 900901-BPR3K308-04BP136070-A 13C.2.fid
Comment BPR3K308-04BP136070-A 13C
Origin Bruker BioSpin GmbH
Owner nmrsu
Instrument Avance
Author Nicki
Solvent DMSO
Temperature 294.7
Pulse Sequence zgpg30
Experiment 1D
Probe Z154705_0043 (PI HR-BBO600S3-BBF/ H/ D-5.0-Z SP)
Number of Scans 14500
Receiver Gain 90.5
Relaxation Delay 2.0000
Pulse Width 11.5000
Acquisition Time 0.9175
Acquisition Date 2022-09-03T05:51:36
Spectrometer Frequency 150.92
Spectral Width 35714.3
Lowest Frequency -1328.2
Nucleus 13C
Acquired Size 32768
Spectral Size 65536
Digital Resolution 0.54
H2N
O
N
N
H
N
H
N
O
O
F
12
f1 (ppm)

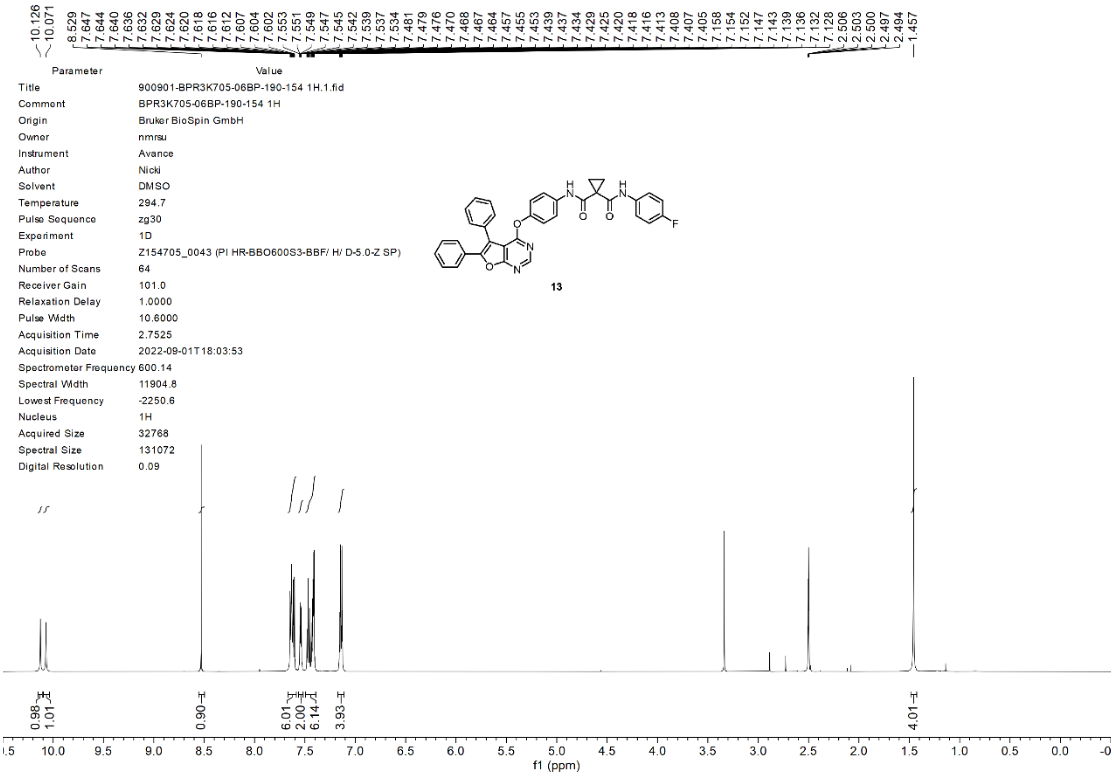

Parameter	Value
Title	900901-BPR3K705-06BP-190-154 1H.1.fid
Comment	BPR3K705-06BP-190-154 1H
Origin	Bruker BioSpin GmbH
Owner	nmrsu
Instrument	Avance
Author	Nicki
Solvent	DMSO
Temperature	294.7
Pulse Sequence	zg30
Experiment	1D
Probe	Z154705_0043 (PI HR-BBO600S3-BBF/ H/ D-5.0-Z SP)
Number of Scans	64
Receiver Gain	101.0
Relaxation Delay	1.0000
Pulse Width	10.6000
Acquisition Time	2.7525
Acquisition Date	2022-09-01T18:03:53
Spectrometer Frequency	600.14
Spectral Width	11904.8
Lowest Frequency	-2250.6
Nucleus	1H
Acquired Size	32768
Spectral Size	131072
Digital Resolution	0.09

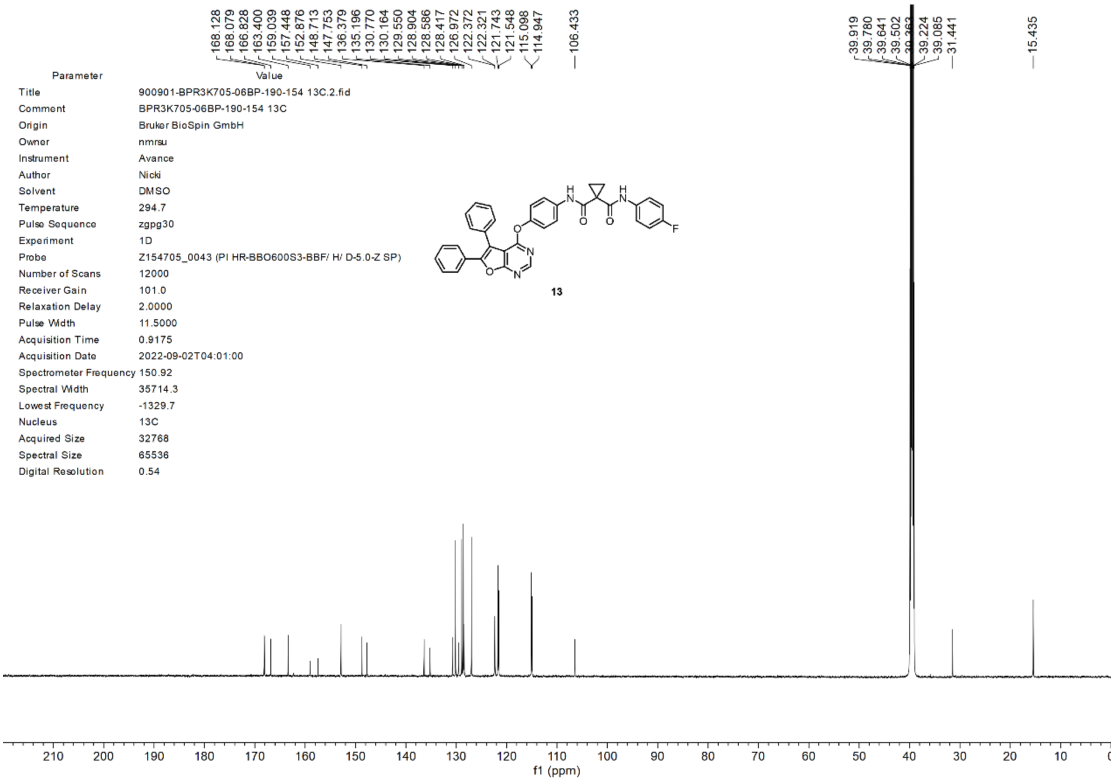

Parameter	Value
Title	900901-BPR3K705-06BP-190-154 13C.2.fid
Comment	BPR3K705-06BP-190-154 13C
Origin	Bruker BioSpin GmbH
Owner	nmrsu
Instrument	Avance
Author	Nicki
Solvent	DMSO
Temperature	294.7
Pulse Sequence	zgpg30
Experiment	1D
Probe	Z154705_0043 (PI HR-BBO600S3-BBF/ H/ D-5.0-Z SP)
Number of Scans	12000
Receiver Gain	101.0
Relaxation Delay	2.0000
Pulse Width	11.5000
Acquisition Time	0.9175
Acquisition Date	2022-09-02T04:01:00
Spectrometer Frequency	150.92
Spectral Width	35714.3
Lowest Frequency	-1329.7
Nucleus	13C
Acquired Size	32768
Spectral Size	65536
Digital Resolution	0.54

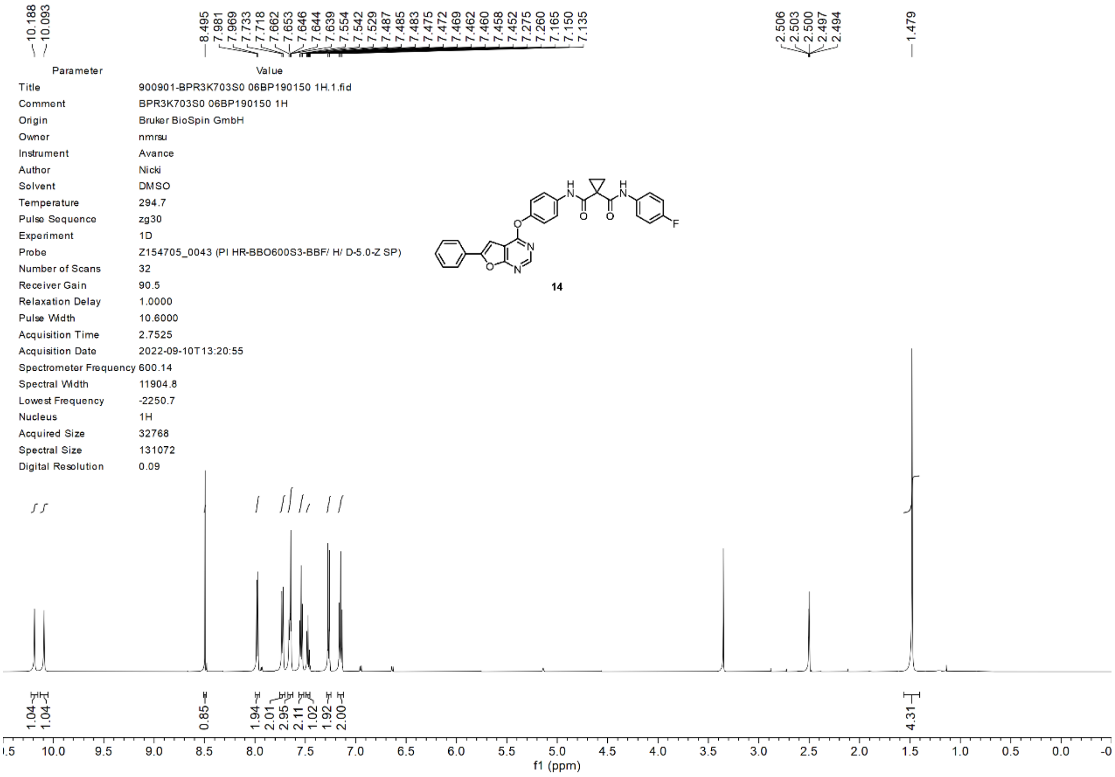
10.188
10.093
8.495
7.981
7.969
7.733
7.718
7.662
7.653
7.646
7.644
7.639
7.554
7.542
7.529
7.487
7.485
7.483
7.475
7.472
7.469
7.462
7.460
7.458
7.452
7.275
7.260
7.165
7.150
7.135
2.506
2.503
2.500
2.497
2.494
1.479
Parameter Value
Title 900901-BPR3K703S0 06BP190150 1H.1.fid
Comment BPR3K703S0 06BP190150 1H
Origin Bruker BioSpin GmbH
Owner nmrsu
Instrument Avance
Author Nicki
Solvent DMSO
Temperature 294.7
Pulse Sequence zg30
Experiment 1D
Probe Z154705_0043 (PI HR-BBO600S3-BBF/ H/ D-5.0-Z SP)
Number of Scans 32
Receiver Gain 90.5
Relaxation Delay 1.0000
Pulse Width 10.6000
Acquisition Time 2.7525
Acquisition Date 2022-09-10T13:20:55
Spectrometer Frequency 600.14
Spectral Width 11904.8
Lowest Frequency -2250.7
Nucleus 1H
Acquired Size 32768
Spectral Size 131072
Digital Resolution 0.09
14
1.04
1.04
0.85
1.94
2.01
2.95
2.11
1.02
1.92
2.00
4.31
f1 (ppm)

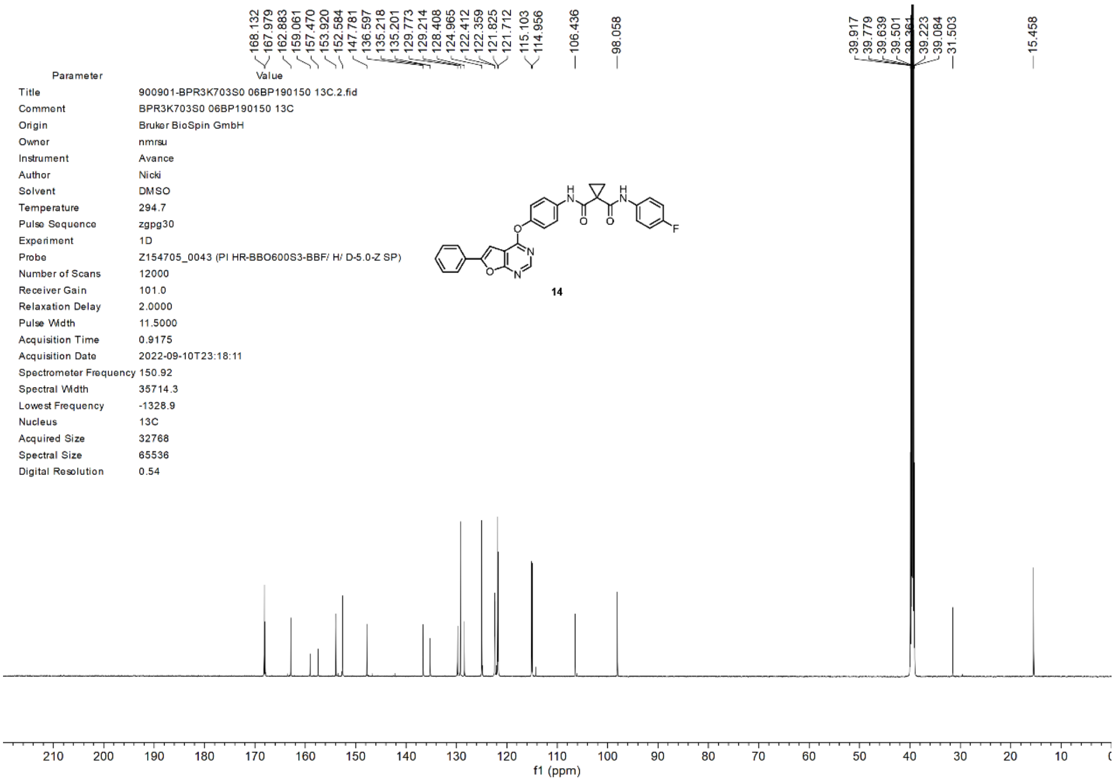
168.132
167.979
162.883
159.061
157.470
153.920
152.584
147.781
136.597
135.218
135.201
129.773
129.214
128.408
124.965
122.412
122.359
121.825
121.712
115.103
114.956
106.436
98.058
39.917
39.779
39.639
39.501
39.361
39.223
39.084
31.503
15.458
Parameter Value
Title 900901-BPR3K703S0 06BP190150 13C.2.fid
Comment BPR3K703S0 06BP190150 13C
Origin Bruker BioSpin GmbH
Owner nmrsu
Instrument Avance
Author Nicki
Solvent DMSO
Temperature 294.7
Pulse Sequence zgpg30
Experiment 1D
Probe Z154705_0043 (PI HR-BBO600S3-BBF/ H/ D-5.0-Z SP)
Number of Scans 12000
Receiver Gain 101.0
Relaxation Delay 2.0000
Pulse Width 11.5000
Acquisition Time 0.9175
Acquisition Date 2022-09-10T23:18:11
Spectrometer Frequency 150.92
Spectral Width 35714.3
Lowest Frequency -1328.9
Nucleus 13C
Acquired Size 32768
Spectral Size 65536
Digital Resolution 0.54
14
f1 (ppm)

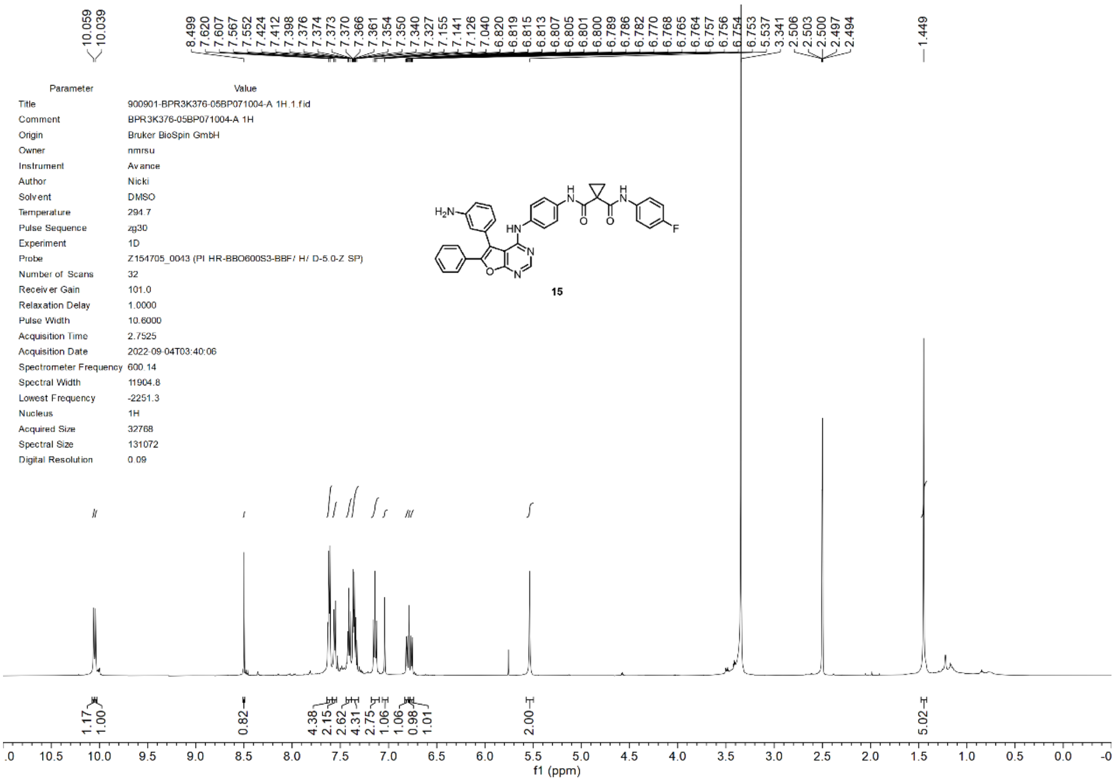
10.059
10.039
8.499
7.620
7.607
7.567
7.552
7.424
7.412
7.398
7.376
7.374
7.373
7.370
7.366
7.361
7.354
7.350
7.340
7.327
7.155
7.141
7.126
7.040
6.820
6.819
6.815
6.813
6.807
6.805
6.801
6.800
6.789
6.786
6.782
6.770
6.768
6.765
6.764
6.757
6.756
6.754
6.753
5.537
3.341
2.506
2.503
2.500
2.497
2.494
1.449
Parameter Value
Title 900901-BPR3K376-05BP071004-A 1H.1.fid
Comment BPR3K376-05BP071004-A 1H
Origin Bruker BioSpin GmbH
Owner nmrsu
Instrument Avance
Author Nicki
Solvent DMSO
Temperature 294.7
Pulse Sequence zg30
Experiment 1D
Probe Z154705_0043 (PI HR-BBO600S3-BBF/ H/ D-5.0-Z SP)
Number of Scans 32
Receiver Gain 101.0
Relaxation Delay 1.0000
Pulse Width 10.6000
Acquisition Time 2.7525
Acquisition Date 2022-09-04T03:40:06
Spectrometer Frequency 600.14
Spectral Width 11904.8
Lowest Frequency -2251.3
Nucleus 1H
Acquired Size 32768
Spectral Size 131072
Digital Resolution 0.09
H2N
HN
N
O
15
F
1.17
1.00
0.82
4.38
2.15
2.62
4.31
2.75
1.06
1.06
0.98
1.01
2.00
5.02
f1 (ppm)

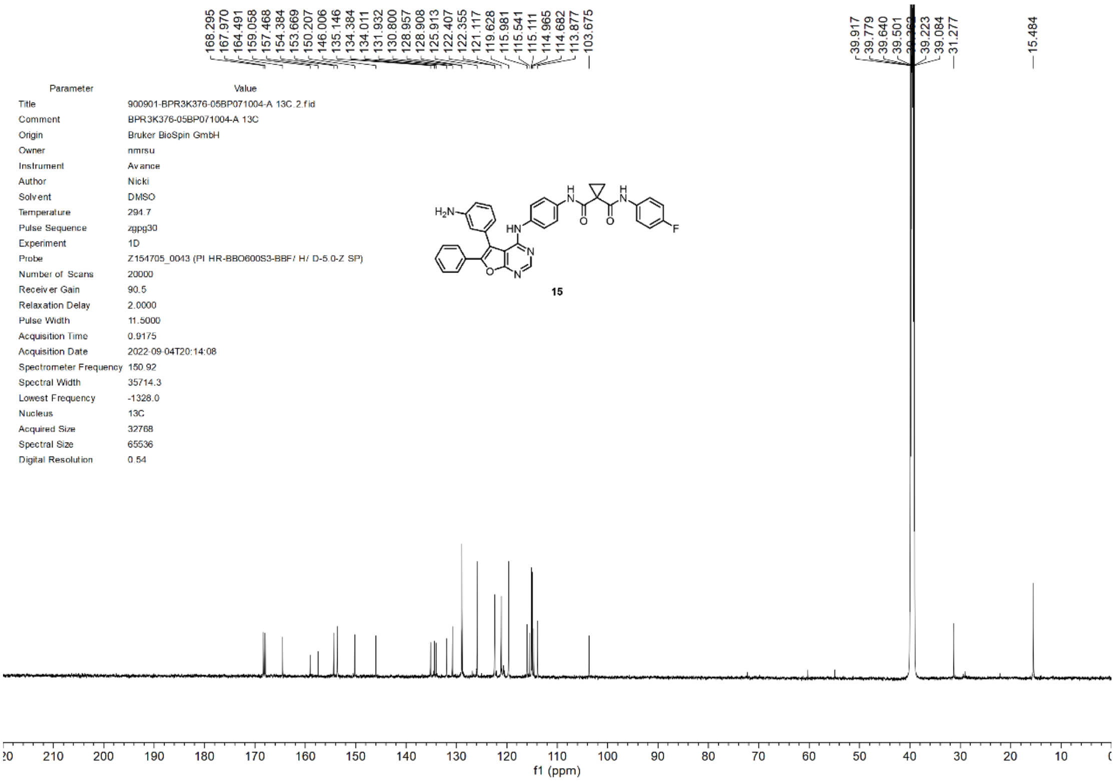
168.295
167.970
164.491
159.058
157.468
154.384
153.669
150.207
146.006
135.146
134.384
134.011
131.932
130.800
128.957
128.908
125.913
122.407
122.355
121.117
119.628
115.981
115.541
115.111
114.965
114.682
113.877
103.675
39.917
39.779
39.640
39.501
39.223
39.084
31.277
15.484
Parameter Value
Title 900901-BPR3K376-05BP071004-A 13C.2.fid
Comment BPR3K376-05BP071004-A 13C
Origin Bruker BioSpin GmbH
Owner nmrsu
Instrument Avance
Author Nicki
Solvent DMSO
Temperature 294.7
Pulse Sequence zgpg30
Experiment 1D
Probe Z154705_0043 (PI HR-BBO600S3-BBF/ H/ D-5.0-Z SP)
Number of Scans 20000
Receiver Gain 90.5
Relaxation Delay 2.0000
Pulse Width 11.5000
Acquisition Time 0.9175
Acquisition Date 2022-09-04T20:14:08
Spectrometer Frequency 150.92
Spectral Width 35714.3
Lowest Frequency -1328.0
Nucleus 13C
Acquired Size 32768
Spectral Size 65536
Digital Resolution 0.54
H2N
HN
N
O
15
F
f1 (ppm)

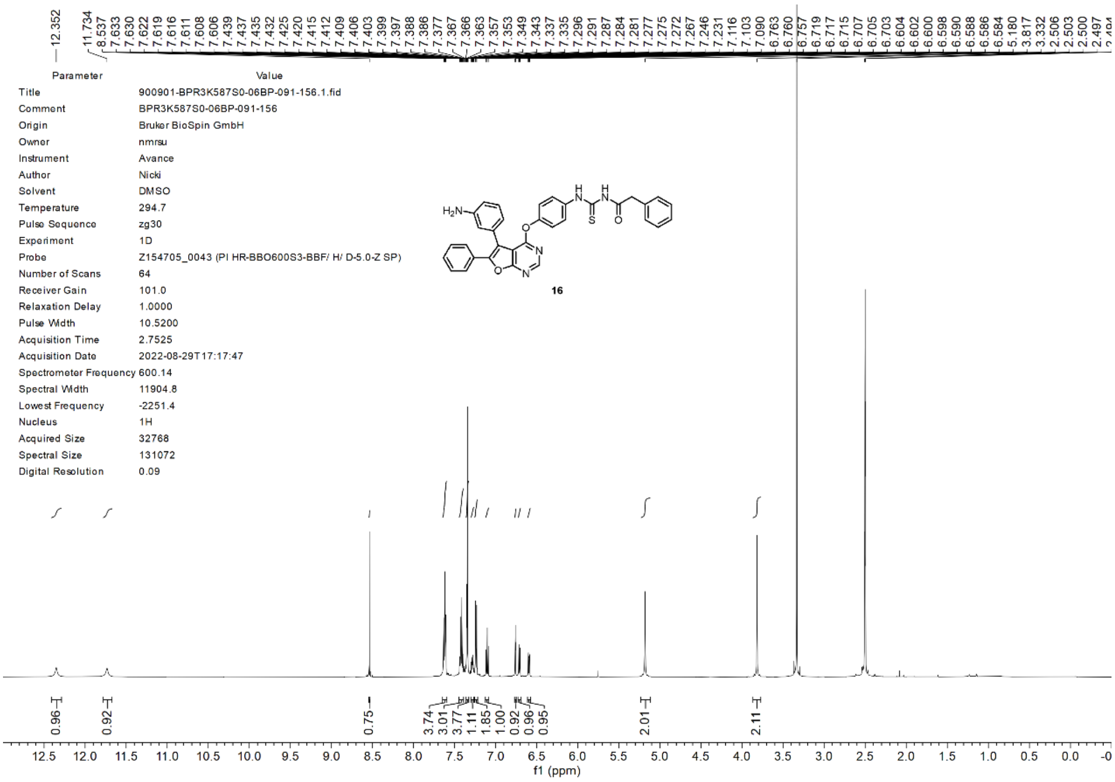
Parameter Value
Title 900901-BPR3K587S0-06BP-091-156.1.fid
Comment BPR3K587S0-06BP-091-156
Origin Bruker BioSpin GmbH
Owner nmrsu
Instrument Avance
Author Nicki
Solvent DMSO
Temperature 294.7
Pulse Sequence zg30
Experiment 1D
Probe Z154705_0043 (PI HR-BBO600S3-BBF/ H/ D-5.0-Z SP)
Number of Scans 64
Receiver Gain 101.0
Relaxation Delay 1.0000
Pulse Width 10.5200
Acquisition Time 2.7525
Acquisition Date 2022-08-29T17:17:47
Spectrometer Frequency 600.14
Spectral Width 11904.8
Lowest Frequency -2251.4
Nucleus 1H
Acquired Size 32768
Spectral Size 131072
Digital Resolution 0.09
16
f1 (ppm)

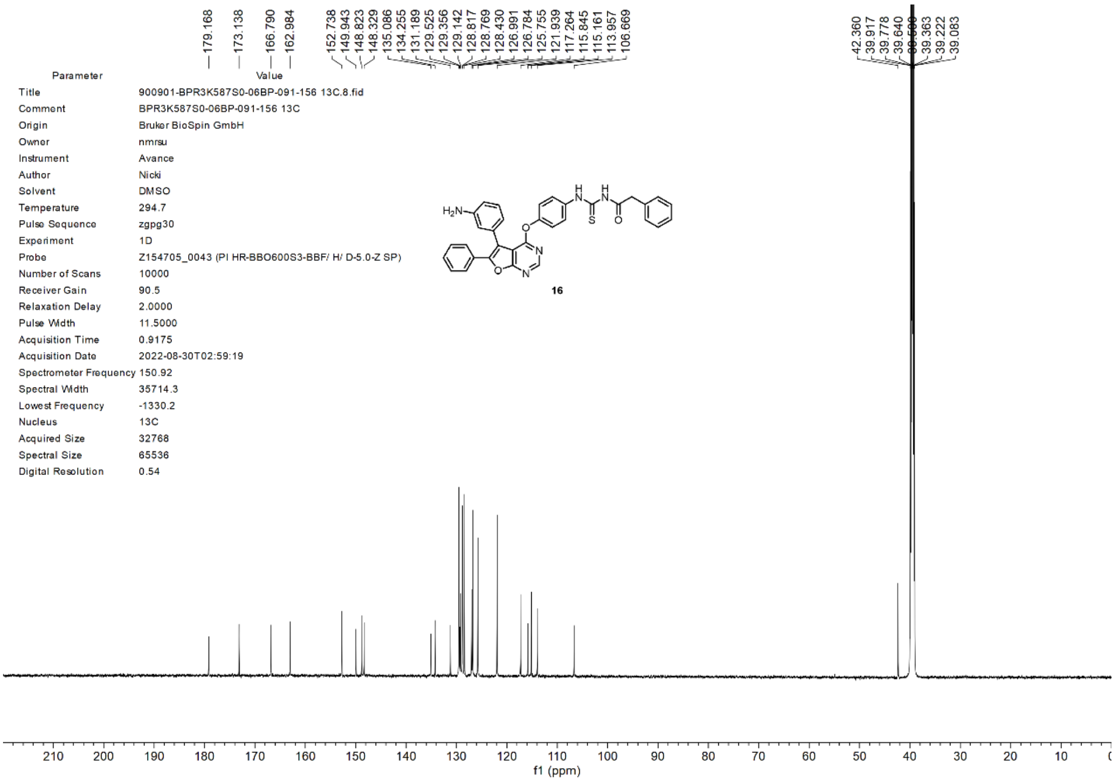
Parameter Value
Title 900901-BPR3K587S0-06BP-091-156 13C.8.fid
Comment BPR3K587S0-06BP-091-156 13C
Origin Bruker BioSpin GmbH
Owner nmrsu
Instrument Avance
Author Nicki
Solvent DMSO
Temperature 294.7
Pulse Sequence zgpg30
Experiment 1D
Probe Z154705_0043 (PI HR-BBO600S3-BBF/ H/ D-5.0-Z SP)
Number of Scans 10000
Receiver Gain 90.5
Relaxation Delay 2.0000
Pulse Width 11.5000
Acquisition Time 0.9175
Acquisition Date 2022-08-30T02:59:19
Spectrometer Frequency 150.92
Spectral Width 35714.3
Lowest Frequency -1330.2
Nucleus 13C
Acquired Size 32768
Spectral Size 65536
Digital Resolution 0.54
16
f1 (ppm)

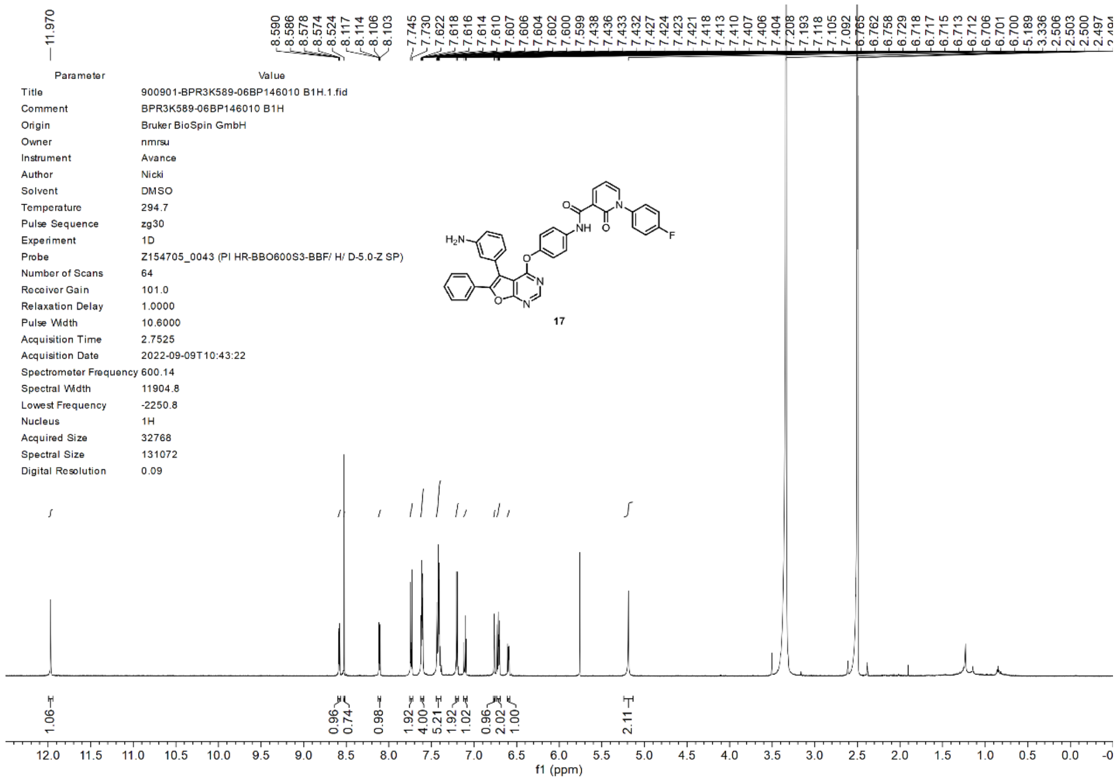
11.970
8.590 8.586 8.578 8.574 8.524 8.117 8.114 8.106 8.103 7.745 7.730 7.622 7.618 7.616 7.614 7.610 7.607 7.606 7.604 7.602 7.600 7.599 7.438 7.436 7.433 7.432 7.427 7.424 7.423 7.421 7.418 7.413 7.410 7.407 7.406 7.404 7.208 7.193 7.118 7.105 7.092 6.765 6.762 6.758 6.729 6.718 6.717 6.715 6.713 6.712 6.706 6.701 6.700 5.189 3.336 2.506 2.503 2.500 2.497
Parameter Value
Title 900901-BPR3K589-06BP146010 B1H.1.fid
Comment BPR3K589-06BP146010 B1H
Origin Bruker BioSpin GmbH
Owner nmrsu
Instrument Avance
Author Nicki
Solvent DMSO
Temperature 294.7
Pulse Sequence zg30
Experiment 1D
Probe Z154705_0043 (PI HR-BBO600S3-BBF/ H/ D-5.0-Z SP)
Number of Scans 64
Receiver Gain 101.0
Relaxation Delay 1.0000
Pulse Width 10.6000
Acquisition Time 2.7525
Acquisition Date 2022-09-09T10:43:22
Spectrometer Frequency 600.14
Spectral Width 11904.8
Lowest Frequency -2250.8
Nucleus 1H
Acquired Size 32768
Spectral Size 131072
Digital Resolution 0.09
H_2N
O NH N F
17
1.06 0.96 0.74 0.98 1.92 4.00 5.21 1.92 1.02 0.96 2.02 1.00 2.11
12.0 11.5 11.0 10.5 10.0 9.5 9.0 8.5 8.0 7.5 7.0 6.5 6.0 5.5 5.0 4.5 4.0 3.5 3.0 2.5 2.0 1.5 1.0 0.5 0.0
f1 (ppm)

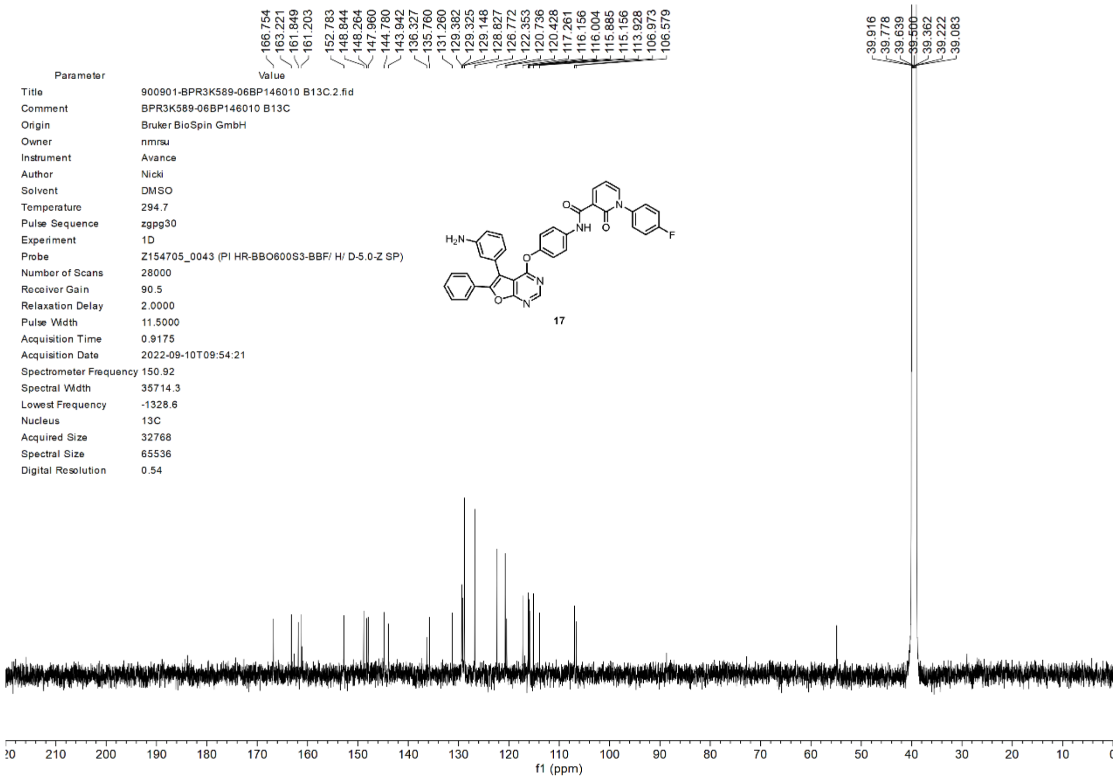
166.754 163.221 161.849 161.203 152.783 148.844 148.264 147.960 144.780 143.942 136.327 135.760 131.260 129.382 129.325 129.148 128.827 126.772 122.353 120.736 120.428 117.261 116.156 116.004 115.885 115.156 113.928 106.973 106.579
39.916 39.778 39.639 39.500 39.362 39.222 39.083
Parameter Value
Title 900901-BPR3K589-06BP146010 B13C.2.fid
Comment BPR3K589-06BP146010 B13C
Origin Bruker BioSpin GmbH
Owner nmrsu
Instrument Avance
Author Nicki
Solvent DMSO
Temperature 294.7
Pulse Sequence zgpg30
Experiment 1D
Probe Z154705_0043 (PI HR-BBO600S3-BBF/ H/ D-5.0-Z SP)
Number of Scans 28000
Receiver Gain 90.5
Relaxation Delay 2.0000
Pulse Width 11.5000
Acquisition Time 0.9175
Acquisition Date 2022-09-10T09:54:21
Spectrometer Frequency 150.92
Spectral Width 35714.3
Lowest Frequency -1328.6
Nucleus 13C
Acquired Size 32768
Spectral Size 65536
Digital Resolution 0.54
H_2N
O NH N F
17
210 200 190 180 170 160 150 140 130 120 110 100 90 80 70 60 50 40 30 20 10
f1 (ppm)

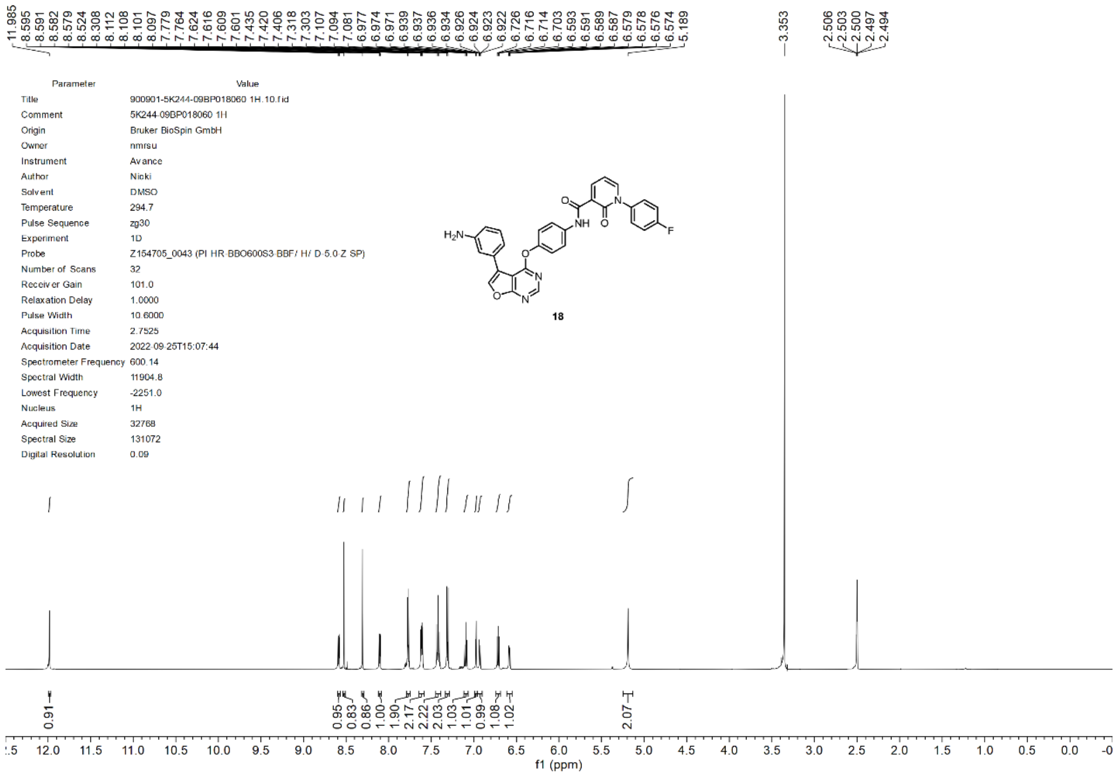
11.985 8.595 8.591 8.582 8.579 8.524 8.308 8.112 8.108 8.101 8.097 7.779 7.764 7.624 7.616 7.609 7.601 7.435 7.420 7.406 7.318 7.303 7.107 7.094 7.081 6.977 6.974 6.971 6.939 6.937 6.936 6.934 6.926 6.924 6.923 6.922 6.726 6.716 6.714 6.703 6.593 6.591 6.589 6.587 6.579 6.578 6.576 6.574 5.189
3.353
2.506 2.503 2.500 2.497 2.494
Parameter Value
Title 900901-5K244-09BP018060 1H.10.fid
Comment 5K244-09BP018060 1H
Origin Bruker BioSpin GmbH
Owner nmrsu
Instrument Avance
Author Nicki
Solvent DMSO
Temperature 294.7
Pulse Sequence zg30
Experiment 1D
Probe Z154705_0043 (PI HR-BBO600S3-BBF/ H/ D-5.0 Z SP)
Number of Scans 32
Receiver Gain 101.0
Relaxation Delay 1.0000
Pulse Width 10.6000
Acquisition Time 2.7525
Acquisition Date 2022-09-25T15:07:44
Spectrometer Frequency 600.14
Spectral Width 11904.8
Lowest Frequency -2251.0
Nucleus 1H
Acquired Size 32768
Spectral Size 131072
Digital Resolution 0.09
H₂N
O
NH
N
F
18
0.91
0.95 0.83 0.86 1.00 1.90 2.17 2.22 2.03 1.03 1.01 0.99 1.08 1.02
2.07
12.0 11.5 11.0 10.5 10.0 9.5 9.0 8.5 8.0 7.5 7.0 6.5 6.0 5.5 5.0 4.5 4.0 3.5 3.0 2.5 2.0 1.5 1.0 0.5 0.0
f1 (ppm)

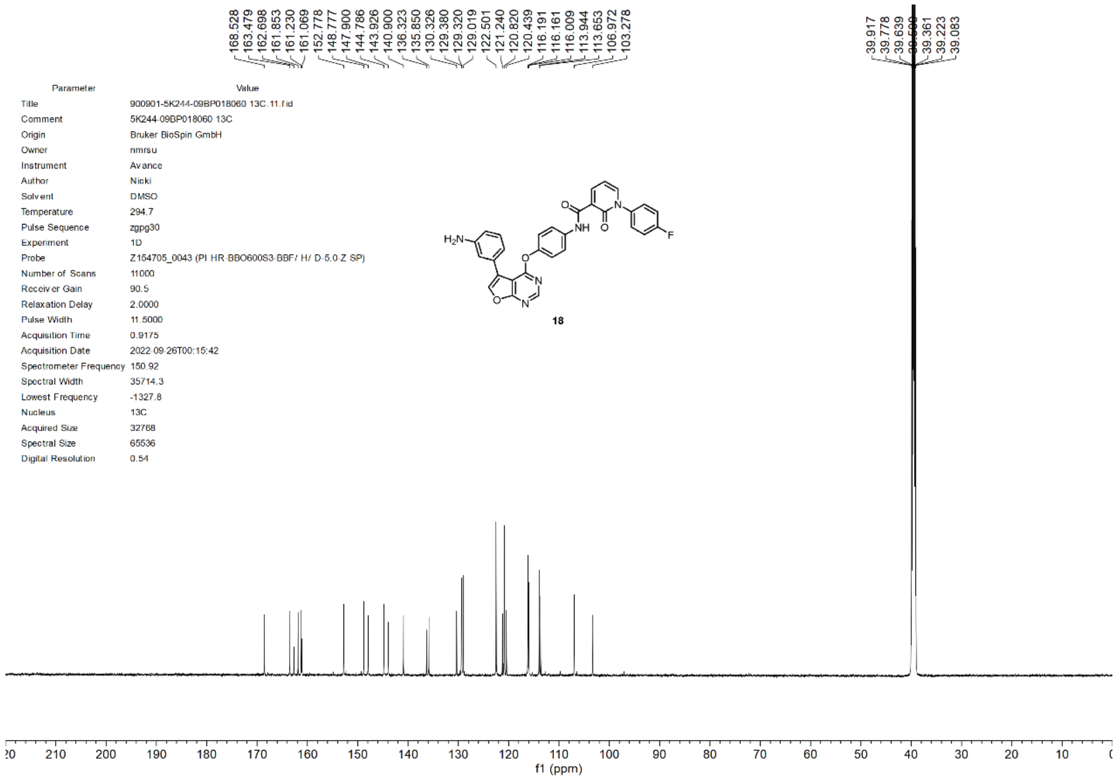
168.528 163.479 162.698 161.853 161.230 161.069 152.778 148.777 147.900 144.786 143.926 140.900 136.323 135.850 130.326 129.380 129.320 129.019 122.501 121.240 120.820 120.439 116.191 116.161 116.009 113.944 113.653 106.972 103.278
39.917 39.778 39.639 39.361 39.223 39.083
Parameter Value
Title 900901-5K244-09BP018060 13C.11.fid
Comment 5K244-09BP018060 13C
Origin Bruker BioSpin GmbH
Owner nmrsu
Instrument Avance
Author Nicki
Solvent DMSO
Temperature 294.7
Pulse Sequence zgpg30
Experiment 1D
Probe Z154705_0043 (PI HR-BBO600S3-BBF/ H/ D-5.0 Z SP)
Number of Scans 11000
Receiver Gain 90.5
Relaxation Delay 2.0000
Pulse Width 11.5000
Acquisition Time 0.9175
Acquisition Date 2022-09-26T00:15:42
Spectrometer Frequency 150.92
Spectral Width 35714.3
Lowest Frequency -1327.8
Nucleus 13C
Acquired Size 32768
Spectral Size 65536
Digital Resolution 0.54
H₂N
O
NH
N
F
18
210 200 190 180 170 160 150 140 130 120 110 100 90 80 70 60 50 40 30 20 10
f1 (ppm)

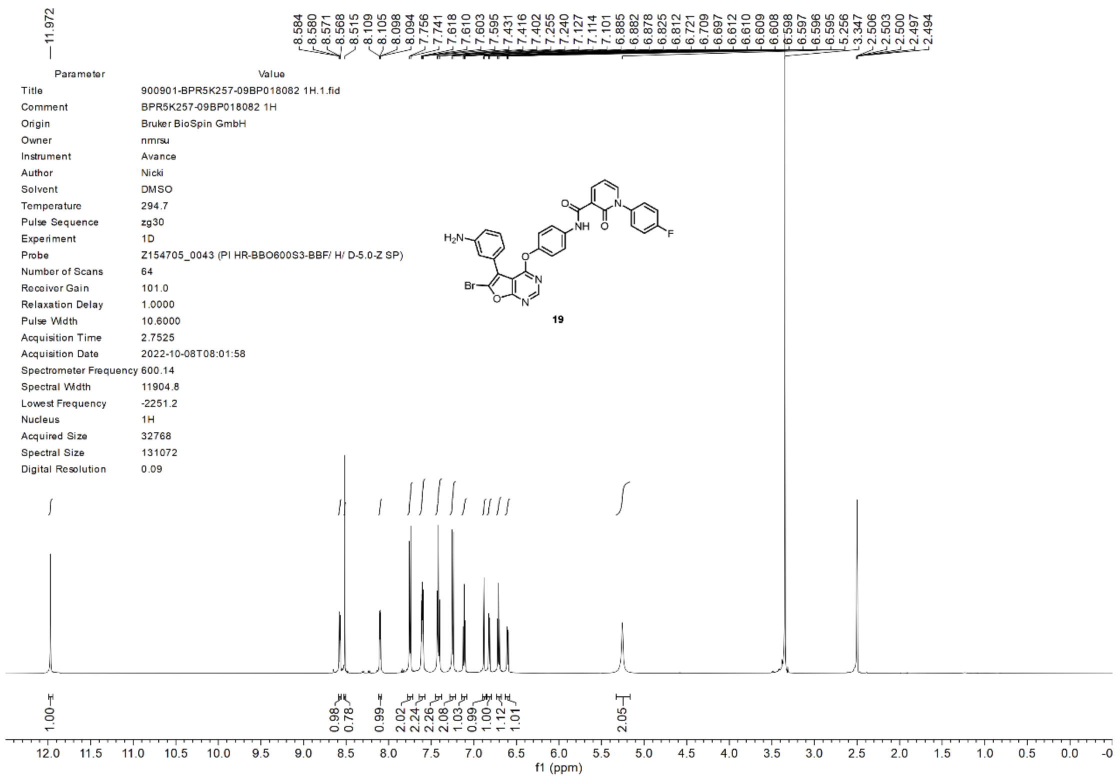
11.972
8.584
8.580
8.571
8.568
8.515
8.109
8.105
8.098
8.094
7.756
7.741
7.618
7.610
7.603
7.595
7.431
7.416
7.402
7.255
7.240
7.127
7.114
7.101
6.885
6.882
6.878
6.825
6.812
6.721
6.709
6.697
6.612
6.610
6.609
6.608
6.598
6.597
6.596
6.595
5.256
3.347
2.506
2.503
2.500
2.497
2.494
Parameter
Value
Title
900901-BPR5K257-09BP018082 1H.1.fid
Comment
BPR5K257-09BP018082 1H
Origin
Bruker BioSpin GmbH
Owner
nmrsu
Instrument
Avance
Author
Nicki
Solvent
DMSO
Temperature
294.7
Pulse Sequence
zg30
Experiment
1D
Probe
Z154705_0043 (PI HR-BBO600S3-BBF/ H/ D-5.0-Z SP)
Number of Scans
64
Receiver Gain
101.0
Relaxation Delay
1.0000
Pulse Width
10.6000
Acquisition Time
2.7525
Acquisition Date
2022-10-08T08:01:58
Spectrometer Frequency
600.14
Spectral Width
11904.8
Lowest Frequency
-2251.2
Nucleus
1H
Acquired Size
32768
Spectral Size
131072
Digital Resolution
0.09
19
1.00
0.98
0.78
0.99
2.02
2.24
2.26
2.08
1.03
0.99
1.00
1.12
1.01
2.05
f1 (ppm)

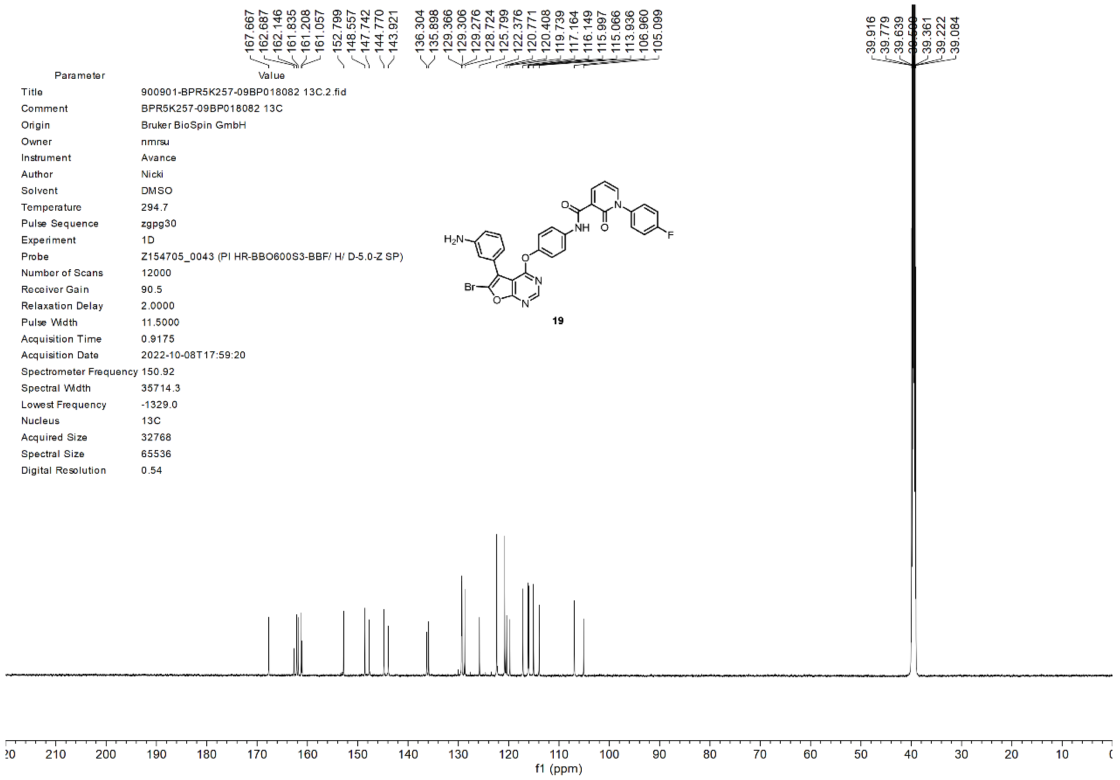
167.667
162.687
162.146
161.835
161.208
161.057
152.799
148.557
147.742
144.770
143.921
136.304
135.898
129.366
129.306
129.276
128.724
125.799
122.376
120.771
120.408
119.739
117.164
116.149
115.997
115.066
113.936
106.960
105.099
39.916
39.779
39.639
39.361
39.222
39.084
Parameter
Value
Title
900901-BPR5K257-09BP018082 13C.2.fid
Comment
BPR5K257-09BP018082 13C
Origin
Bruker BioSpin GmbH
Owner
nmrsu
Instrument
Avance
Author
Nicki
Solvent
DMSO
Temperature
294.7
Pulse Sequence
zgpg30
Experiment
1D
Probe
Z154705_0043 (PI HR-BBO600S3-BBF/ H/ D-5.0-Z SP)
Number of Scans
12000
Receiver Gain
90.5
Relaxation Delay
2.0000
Pulse Width
11.5000
Acquisition Time
0.9175
Acquisition Date
2022-10-08T17:59:20
Spectrometer Frequency
150.92
Spectral Width
35714.3
Lowest Frequency
-1329.0
Nucleus
13C
Acquired Size
32768
Spectral Size
65536
Digital Resolution
0.54
19
f1 (ppm)

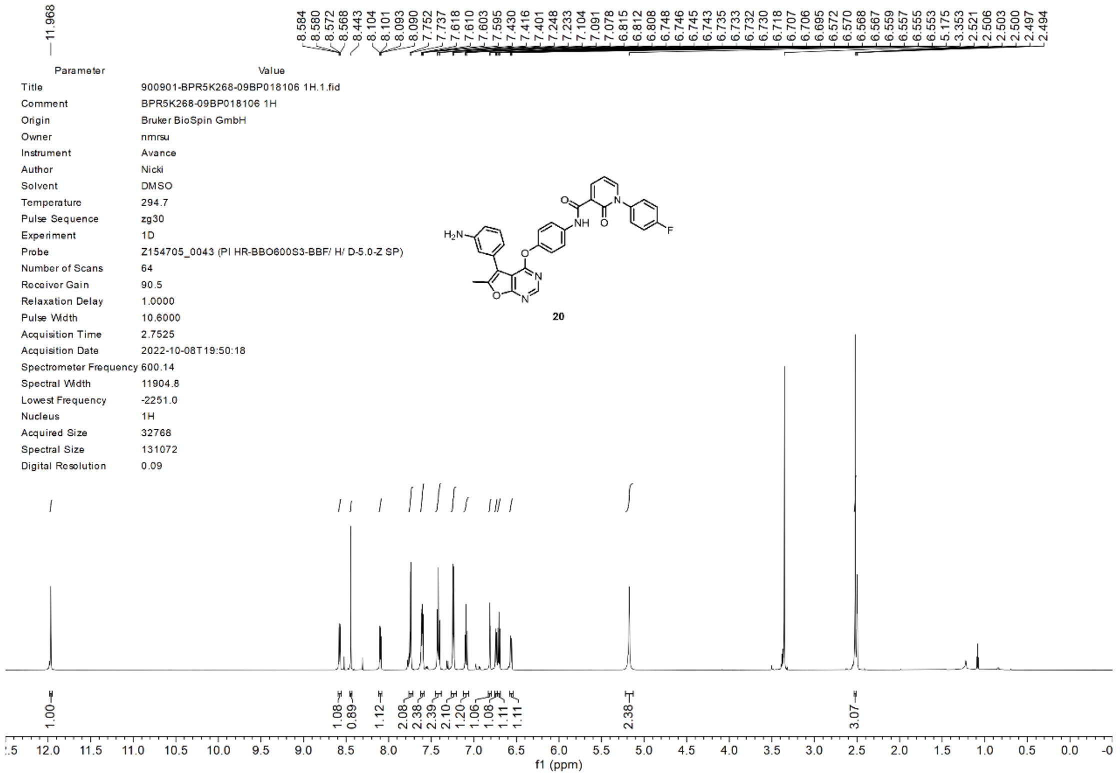

Parameter	Value
Title	900901-BPR5K268-09BP018106 1H.1.fid
Comment	BPR5K268-09BP018106 1H
Origin	Bruker BioSpin GmbH
Owner	nmrsu
Instrument	Avance
Author	Nicki
Solvent	DMSO
Temperature	294.7
Pulse Sequence	zg30
Experiment	1D
Probe	Z154705_0043 (PI HR-BBO600S3-BBF/ H/ D-5.0-Z SP)
Number of Scans	64
Receiver Gain	90.5
Relaxation Delay	1.0000
Pulse Width	10.6000
Acquisition Time	2.7525
Acquisition Date	2022-10-08T19:50:18
Spectrometer Frequency	600.14
Spectral Width	11904.8
Lowest Frequency	-2251.0
Nucleus	1H
Acquired Size	32768
Spectral Size	131072
Digital Resolution	0.09

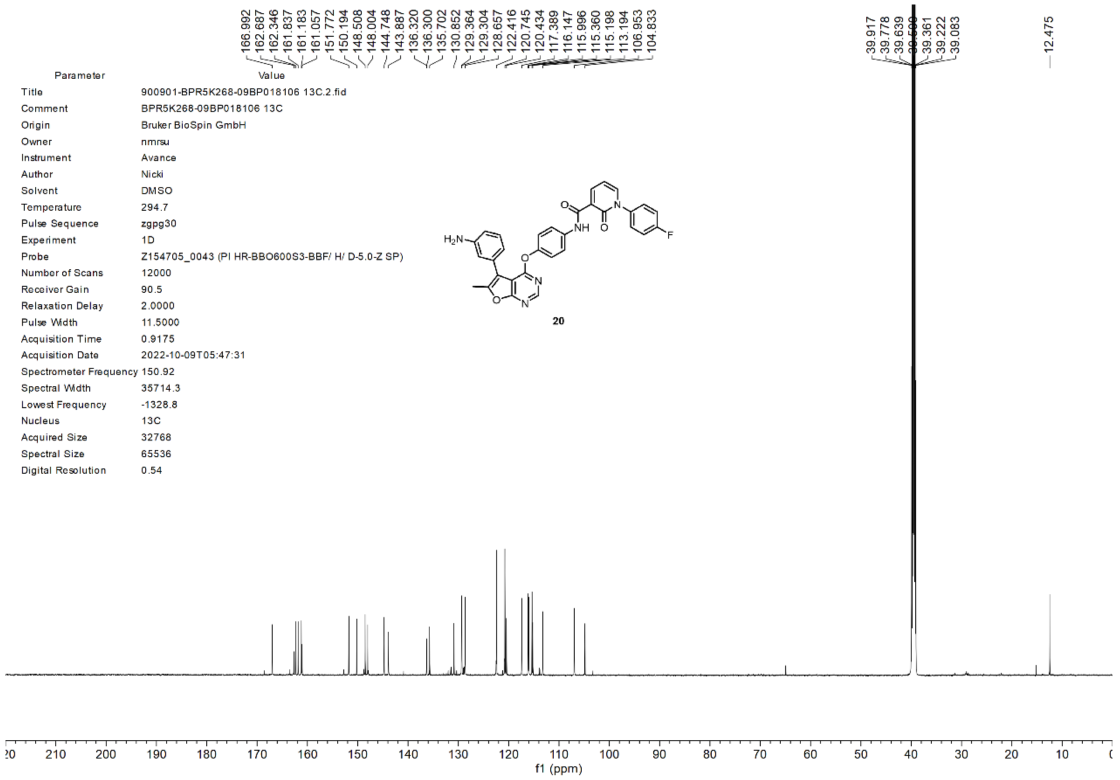

Parameter	Value
Title	900901-BPR5K268-09BP018106 13C.2.fid
Comment	BPR5K268-09BP018106 13C
Origin	Bruker BioSpin GmbH
Owner	nmrsu
Instrument	Avance
Author	Nicki
Solvent	DMSO
Temperature	294.7
Pulse Sequence	zgpg30
Experiment	1D
Probe	Z154705_0043 (PI HR-BBO600S3-BBF/ H/ D-5.0-Z SP)
Number of Scans	12000
Receiver Gain	90.5
Relaxation Delay	2.0000
Pulse Width	11.5000
Acquisition Time	0.9175
Acquisition Date	2022-10-09T05:47:31
Spectrometer Frequency	150.92
Spectral Width	35714.3
Lowest Frequency	-1328.8
Nucleus	13C
Acquired Size	32768
Spectral Size	65536
Digital Resolution	0.54

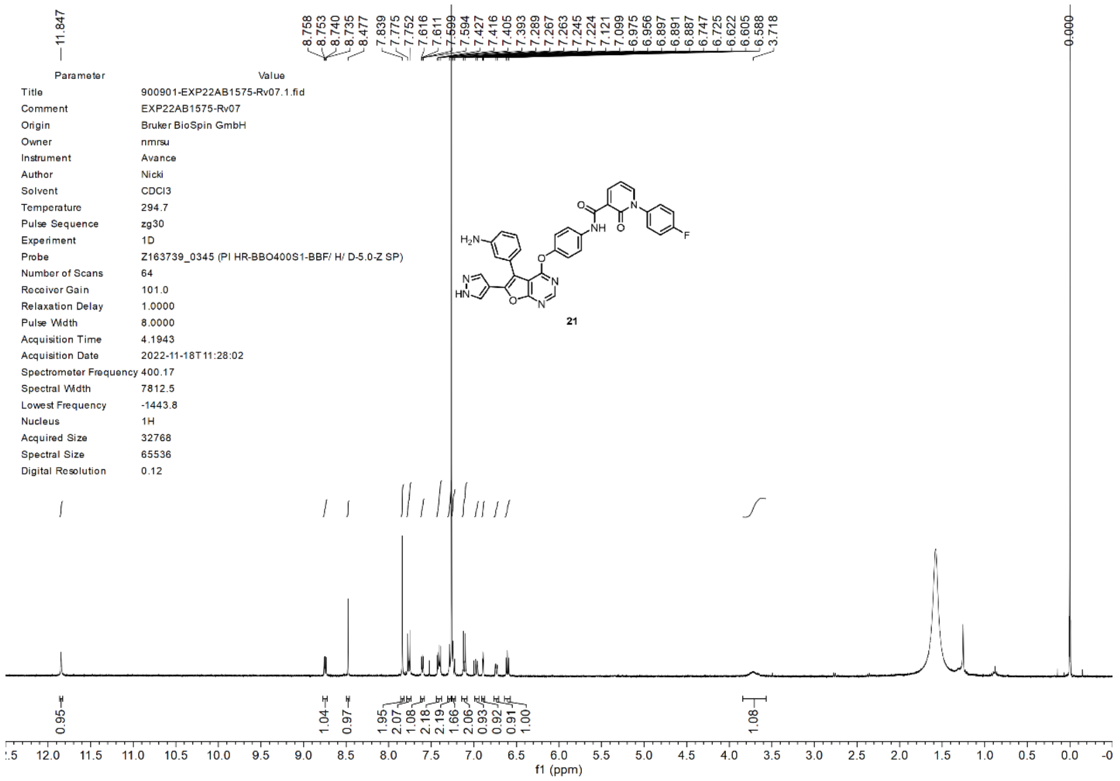
11.847
8.758
8.753
8.740
8.735
8.477
7.839
7.775
7.752
7.616
7.611
7.594
7.427
7.416
7.405
7.393
7.289
7.267
7.263
7.245
7.224
7.121
7.099
6.975
6.956
6.897
6.891
6.887
6.747
6.725
6.622
6.605
6.588
3.718
0.000
Parameter Value
Title 900901-EXP22AB1575-Rv07.1.fid
Comment EXP22AB1575-Rv07
Origin Bruker BioSpin GmbH
Owner nmrsu
Instrument Avance
Author Nicki
Solvent CDCl3
Temperature 294.7
Pulse Sequence zg30
Experiment 1D
Probe Z163739_0345 (PI HR-BBO400S1-BBF/ H/ D-5.0-Z SP)
Number of Scans 64
Receiver Gain 101.0
Relaxation Delay 1.0000
Pulse Width 8.0000
Acquisition Time 4.1943
Acquisition Date 2022-11-18T11:28:02
Spectrometer Frequency 400.17
Spectral Width 7812.5
Lowest Frequency -1443.8
Nucleus 1H
Acquired Size 32768
Spectral Size 65536
Digital Resolution 0.12
21
0.95
1.04
0.97
1.95
2.07
1.08
2.18
2.19
1.66
2.06
0.93
0.92
0.91
1.00
1.08
f1 (ppm)

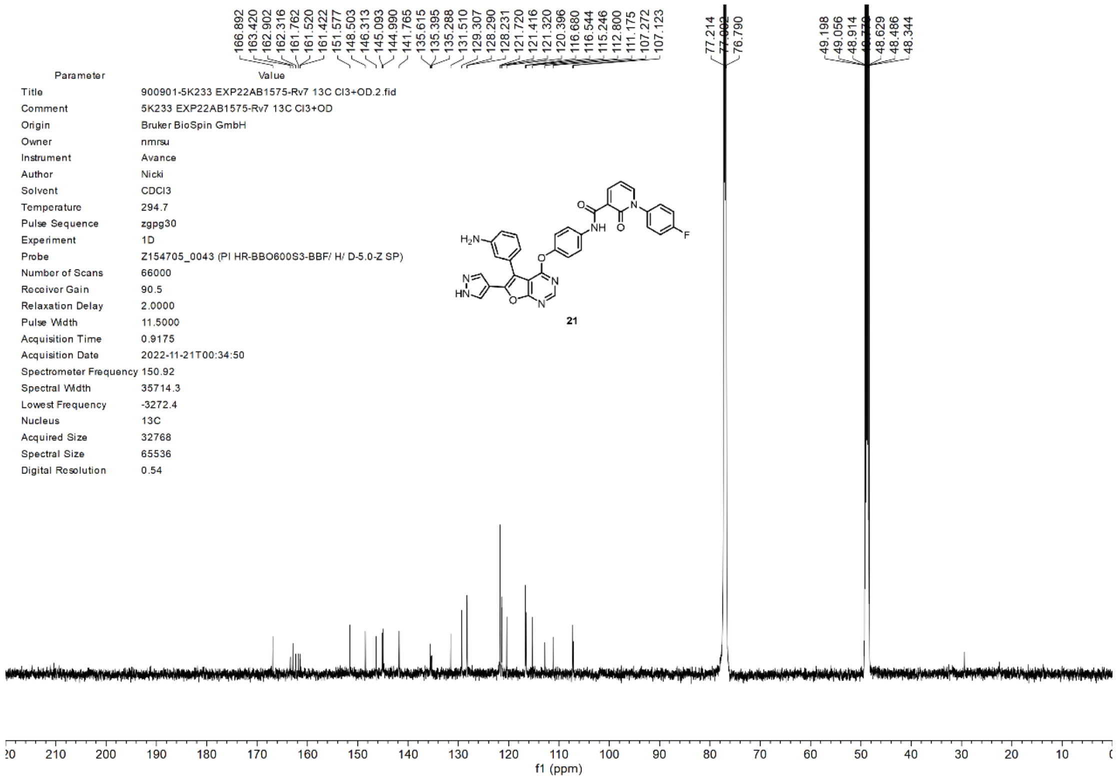
166.892
163.420
162.902
162.316
161.762
161.520
161.422
151.577
148.503
146.313
145.093
144.990
141.765
135.615
135.395
135.288
131.510
129.307
128.290
128.231
121.720
121.416
121.320
120.396
116.680
116.544
115.246
112.800
111.175
107.272
107.123
77.214
76.790
49.198
49.056
48.914
48.629
48.486
48.344
Parameter Value
Title 900901-5K233 EXP22AB1575-Rv7 13C Cl3+OD.2.fid
Comment 5K233 EXP22AB1575-Rv7 13C Cl3+OD
Origin Bruker BioSpin GmbH
Owner nmrsu
Instrument Avance
Author Nicki
Solvent CDCl3
Temperature 294.7
Pulse Sequence zgpg30
Experiment 1D
Probe Z154705_0043 (PI HR-BBO600S3-BBF/ H/ D-5.0-Z SP)
Number of Scans 66000
Receiver Gain 90.5
Relaxation Delay 2.0000
Pulse Width 11.5000
Acquisition Time 0.9175
Acquisition Date 2022-11-21T00:34:50
Spectrometer Frequency 150.92
Spectral Width 35714.3
Lowest Frequency -3272.4
Nucleus 13C
Acquired Size 32768
Spectral Size 65536
Digital Resolution 0.54
21
f1 (ppm)

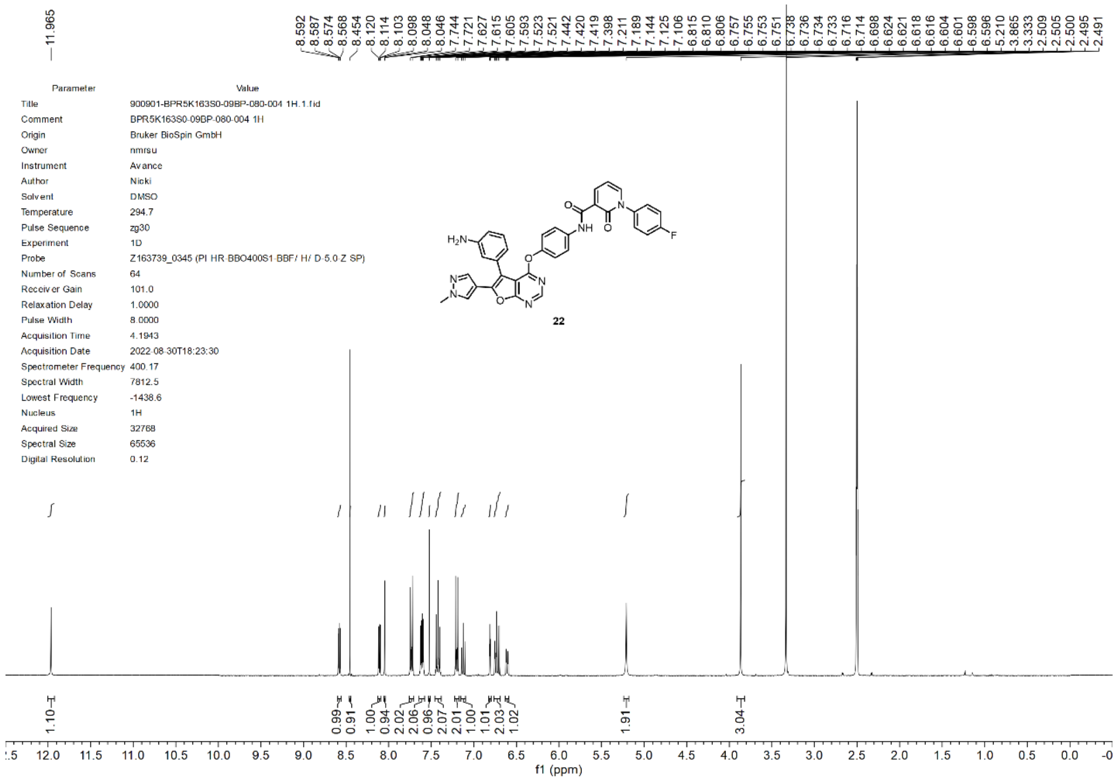
11.965
8.592 8.587 8.574 8.568 8.454 8.120 8.114 8.103 8.098 8.048 8.046 7.744 7.721 7.627 7.615 7.605 7.593 7.523 7.521 7.442 7.420 7.419 7.398 7.211 7.189 7.144 7.125 7.106 6.815 6.810 6.806 6.757 6.753 6.751 6.738 6.736 6.734 6.733 6.716 6.714 6.698 6.624 6.621 6.618 6.616 6.604 6.601 6.598 6.596 5.210 3.865 3.333 2.509 2.505 2.500 2.495 2.491
Parameter Value
Title 900901-BPR5K163S0-09BP-080-004 1H.1.fid
Comment BPR5K163S0-09BP-080-004 1H
Origin Bruker BioSpin GmbH
Owner nmrsu
Instrument Avance
Author Nicki
Solvent DMSO
Temperature 294.7
Pulse Sequence zg30
Experiment 1D
Probe Z163739_0345 (PI HR-BBO400S1-BBF/ H/ D-5.0 Z SP)
Number of Scans 64
Receiver Gain 101.0
Relaxation Delay 1.0000
Pulse Width 8.0000
Acquisition Time 4.1943
Acquisition Date 2022-08-30T18:23:30
Spectrometer Frequency 400.17
Spectral Width 7812.5
Lowest Frequency -1438.6
Nucleus 1H
Acquired Size 32768
Spectral Size 65536
Digital Resolution 0.12
H2N
O
NH
N
F
22
1.10 0.99 0.91 1.00 0.94 2.02 2.06 0.96 2.07 2.01 1.00 1.01 2.03 1.02 1.91 3.04
f1 (ppm)

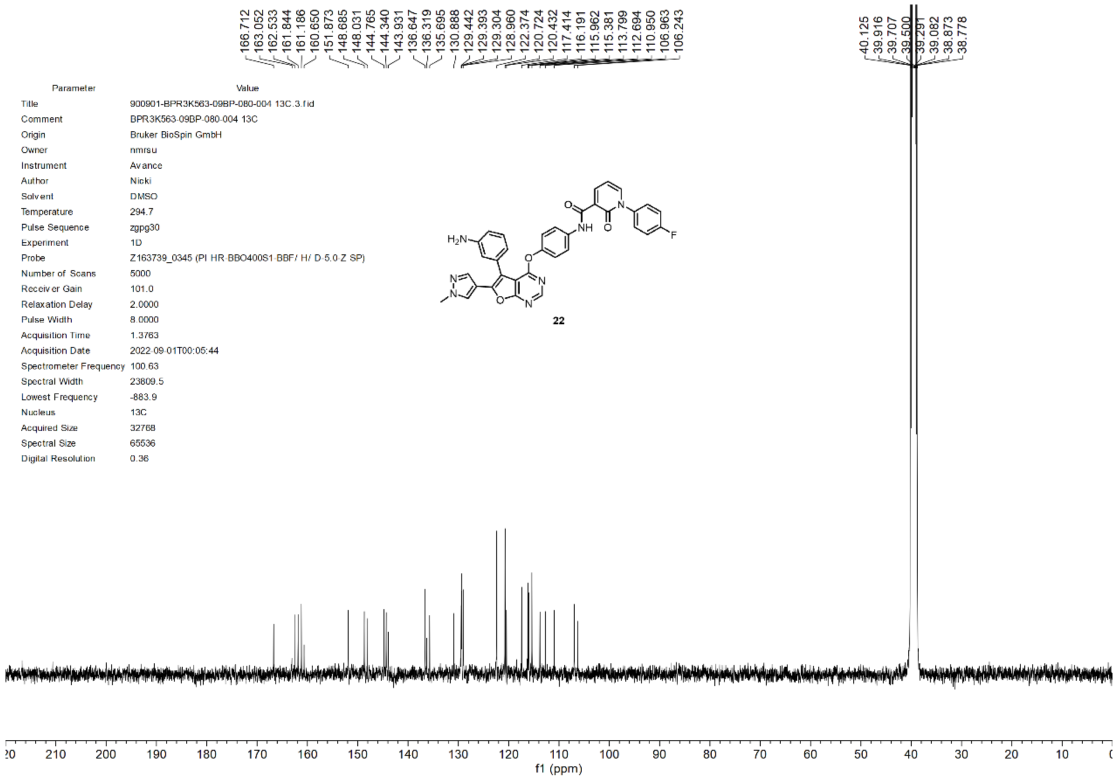
166.712 163.052 162.533 161.844 161.186 160.650 151.873 148.685 148.031 144.765 144.340 143.931 136.647 136.319 135.695 130.888 129.442 129.393 129.304 128.960 122.374 120.724 120.432 117.414 116.191 115.962 115.381 113.799 112.694 110.950 106.963 106.243
40.125 39.916 39.707 39.500 39.291 39.082 38.873 38.778
Parameter Value
Title 900901-BPR3K563-09BP-080-004 13C.3.fid
Comment BPR3K563-09BP-080-004 13C
Origin Bruker BioSpin GmbH
Owner nmrsu
Instrument Avance
Author Nicki
Solvent DMSO
Temperature 294.7
Pulse Sequence zgpg30
Experiment 1D
Probe Z163739_0345 (PI HR-BBO400S1-BBF/ H/ D-5.0 Z SP)
Number of Scans 5000
Receiver Gain 101.0
Relaxation Delay 2.0000
Pulse Width 8.0000
Acquisition Time 1.3763
Acquisition Date 2022-09-01T00:05:44
Spectrometer Frequency 100.63
Spectral Width 23809.5
Lowest Frequency -883.9
Nucleus 13C
Acquired Size 32768
Spectral Size 65536
Digital Resolution 0.36
22
f1 (ppm)

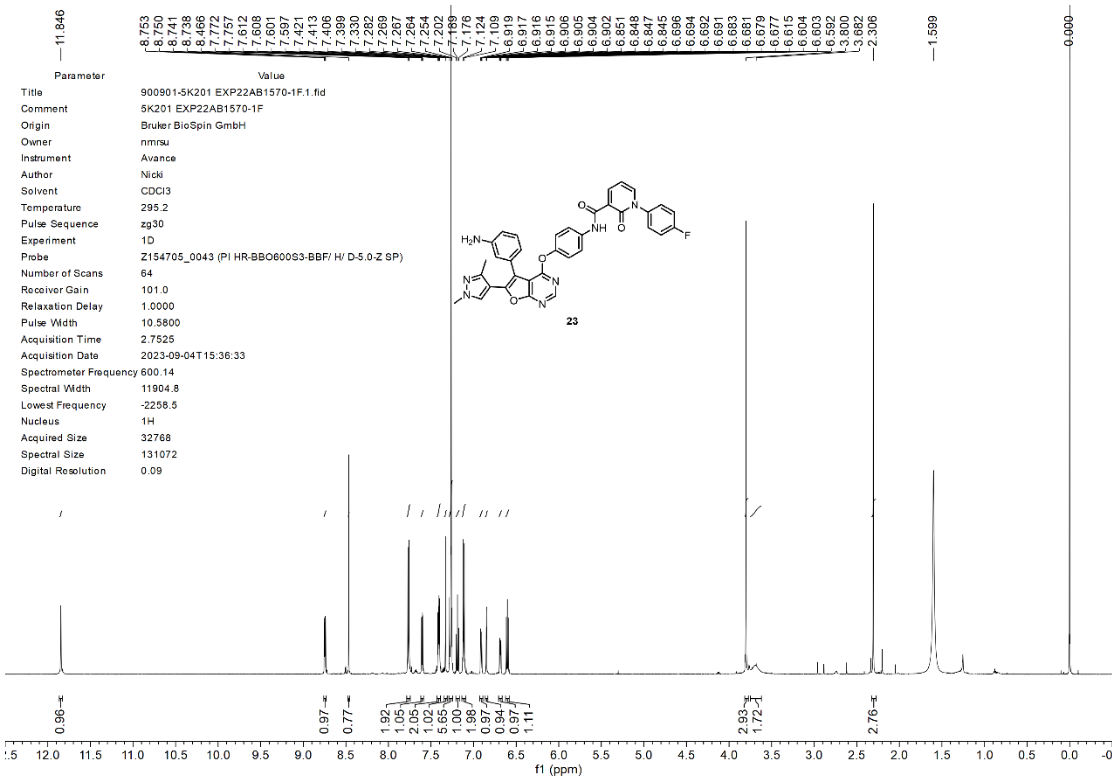

Parameter	Value
Title	900901-5K201 EXP22AB1570-1F.1.fid
Comment	5K201 EXP22AB1570-1F
Origin	Bruker BioSpin GmbH
Owner	nmrsu
Instrument	Avance
Author	Nicki
Solvent	CDCl3
Temperature	295.2
Pulse Sequence	zg30
Experiment	1D
Probe	Z154705_0043 (PI HR-BBO600S3-BBF/ H/ D-5.0-Z SP)
Number of Scans	64
Receiver Gain	101.0
Relaxation Delay	1.0000
Pulse Width	10.5800
Acquisition Time	2.7525
Acquisition Date	2023-09-04T15:36:33
Spectrometer Frequency	600.14
Spectral Width	11904.8
Lowest Frequency	-2258.5
Nucleus	1H
Acquired Size	32768
Spectral Size	131072
Digital Resolution	0.09

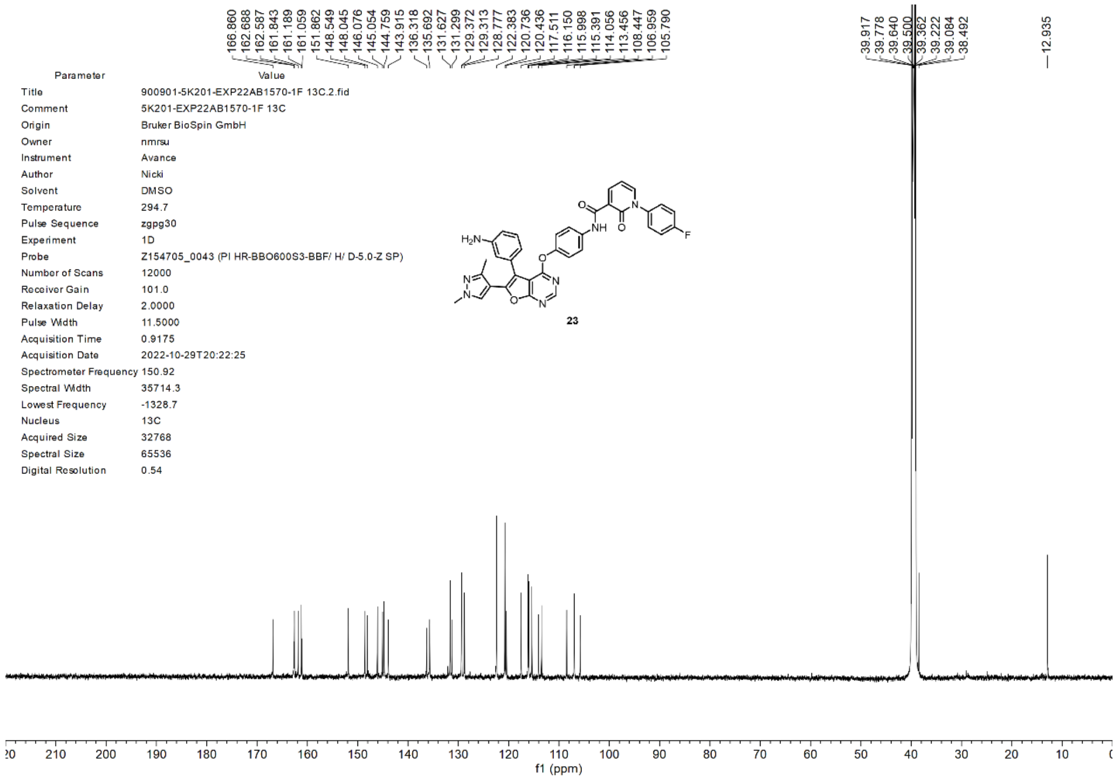

Parameter	Value
Title	900901-5K201-EXP22AB1570-1F 13C.2.fid
Comment	5K201-EXP22AB1570-1F 13C
Origin	Bruker BioSpin GmbH
Owner	nmrsu
Instrument	Avance
Author	Nicki
Solvent	DMSO
Temperature	294.7
Pulse Sequence	zgpg30
Experiment	1D
Probe	Z154705_0043 (PI HR-BBO600S3-BBF/ H/ D-5.0-Z SP)
Number of Scans	12000
Receiver Gain	101.0
Relaxation Delay	2.0000
Pulse Width	11.5000
Acquisition Time	0.9175
Acquisition Date	2022-10-29T20:22:25
Spectrometer Frequency	150.92
Spectral Width	35714.3
Lowest Frequency	-1328.7
Nucleus	13C
Acquired Size	32768
Spectral Size	65536
Digital Resolution	0.54

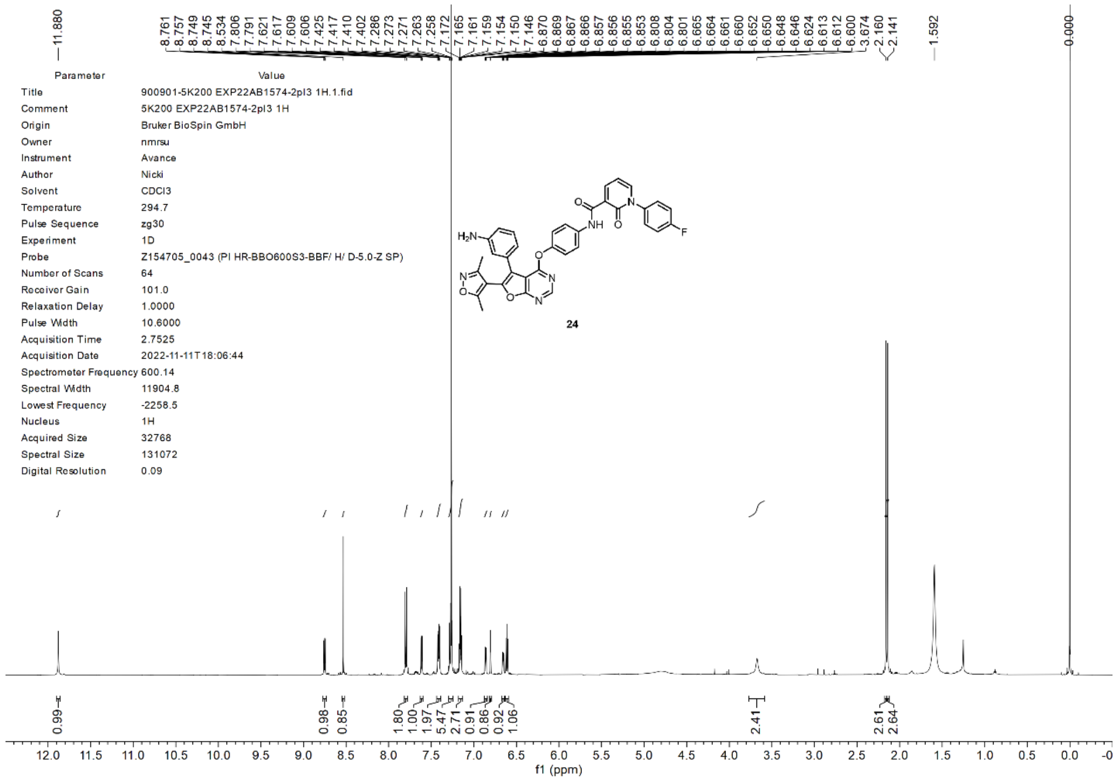
11.880
8.761 8.757 8.749 8.745 8.534 7.806 7.791 7.621 7.617 7.609 7.606 7.425 7.417 7.410 7.402 7.286 7.273 7.271 7.263 7.258 7.172 7.165 7.161 7.159 7.154 7.150 7.146 6.870 6.869 6.867 6.866 6.857 6.856 6.855 6.853 6.808 6.804 6.801 6.665 6.664 6.661 6.660 6.652 6.650 6.648 6.646 6.624 6.613 6.612 6.600 3.674 2.160 2.141
1.592
0.000
Parameter Value
Title 900901-5K200 EXP22AB1574-2pl3 1H.1.fid
Comment 5K200 EXP22AB1574-2pl3 1H
Origin Bruker BioSpin GmbH
Owner nmrsu
Instrument Avance
Author Nicki
Solvent CDCl3
Temperature 294.7
Pulse Sequence zg30
Experiment 1D
Probe Z154705_0043 (PI HR-BBO600S3-BBF/ H/ D-5.0-Z SP)
Number of Scans 64
Receiver Gain 101.0
Relaxation Delay 1.0000
Pulse Width 10.6000
Acquisition Time 2.7525
Acquisition Date 2022-11-11T18:06:44
Spectrometer Frequency 600.14
Spectral Width 11904.8
Lowest Frequency -2258.5
Nucleus 1H
Acquired Size 32768
Spectral Size 131072
Digital Resolution 0.09
H2N
O
NH
N
F
N
O
24
0.99 0.98 0.85 1.80 1.00 1.97 5.47 2.71 0.91 0.86 0.92 1.06 2.41 2.61 2.64
12.0 11.5 11.0 10.5 10.0 9.5 9.0 8.5 8.0 7.5 7.0 6.5 6.0 5.5 5.0 4.5 4.0 3.5 3.0 2.5 2.0 1.5 1.0 0.5 0.0 -0
f1 (ppm)

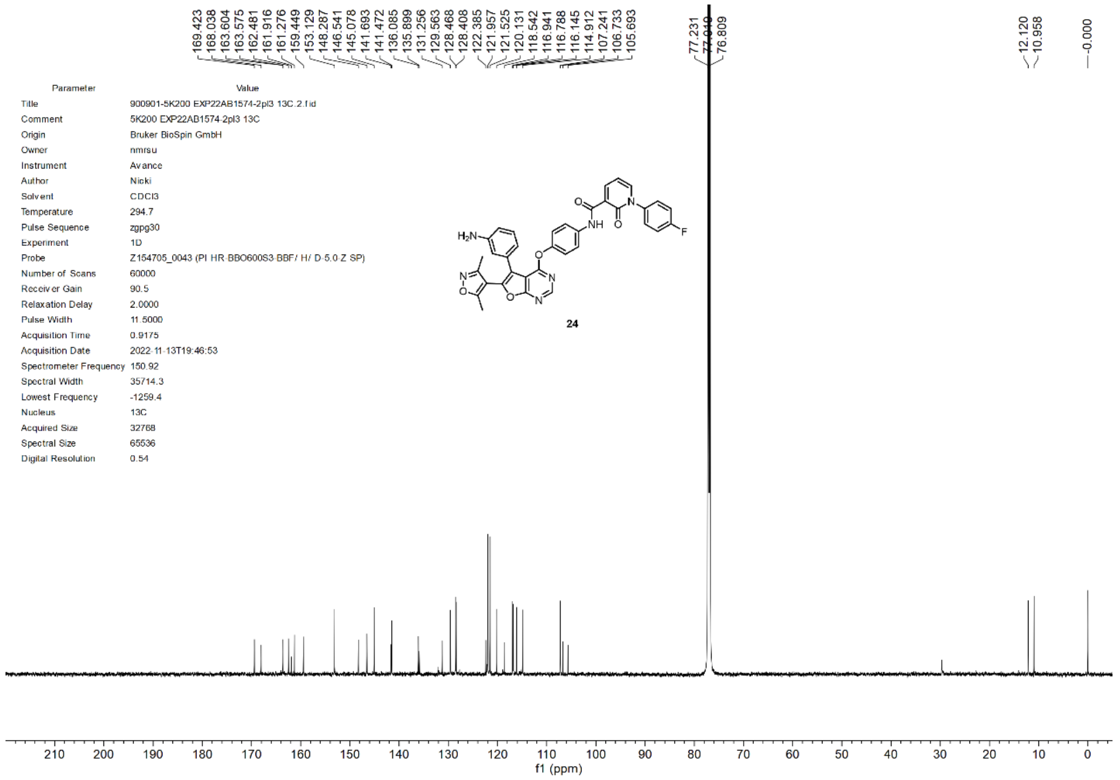
169.423 168.038 163.604 163.575 162.481 161.916 161.276 159.449 153.129 148.287 146.541 145.078 141.693 141.472 136.085 135.899 131.256 129.563 128.468 128.408 122.385 121.957 121.525 120.131 118.542 116.941 116.788 116.145 114.912 107.241 106.733 105.693
77.231 [illegible] 76.809
12.120 10.958
-0.000
Parameter Value
Title 900901-5K200 EXP22AB1574-2pl3 13C.2.fid
Comment 5K200 EXP22AB1574-2pl3 13C
Origin Bruker BioSpin GmbH
Owner nmrsu
Instrument Avance
Author Nicki
Solvent CDCl3
Temperature 294.7
Pulse Sequence zgpg30
Experiment 1D
Probe Z154705_0043 (PI HR-BBO600S3-BBF/ H/ D-5.0-Z SP)
Number of Scans 60000
Receiver Gain 90.5
Relaxation Delay 2.0000
Pulse Width 11.5000
Acquisition Time 0.9175
Acquisition Date 2022-11-13T19:46:53
Spectrometer Frequency 150.92
Spectral Width 35714.3
Lowest Frequency -1259.4
Nucleus 13C
Acquired Size 32768
Spectral Size 65536
Digital Resolution 0.54
H2N
O
NH
N
F
N
O
24
210 200 190 180 170 160 150 140 130 120 110 100 90 80 70 60 50 40 30 20 10 0
f1 (ppm)

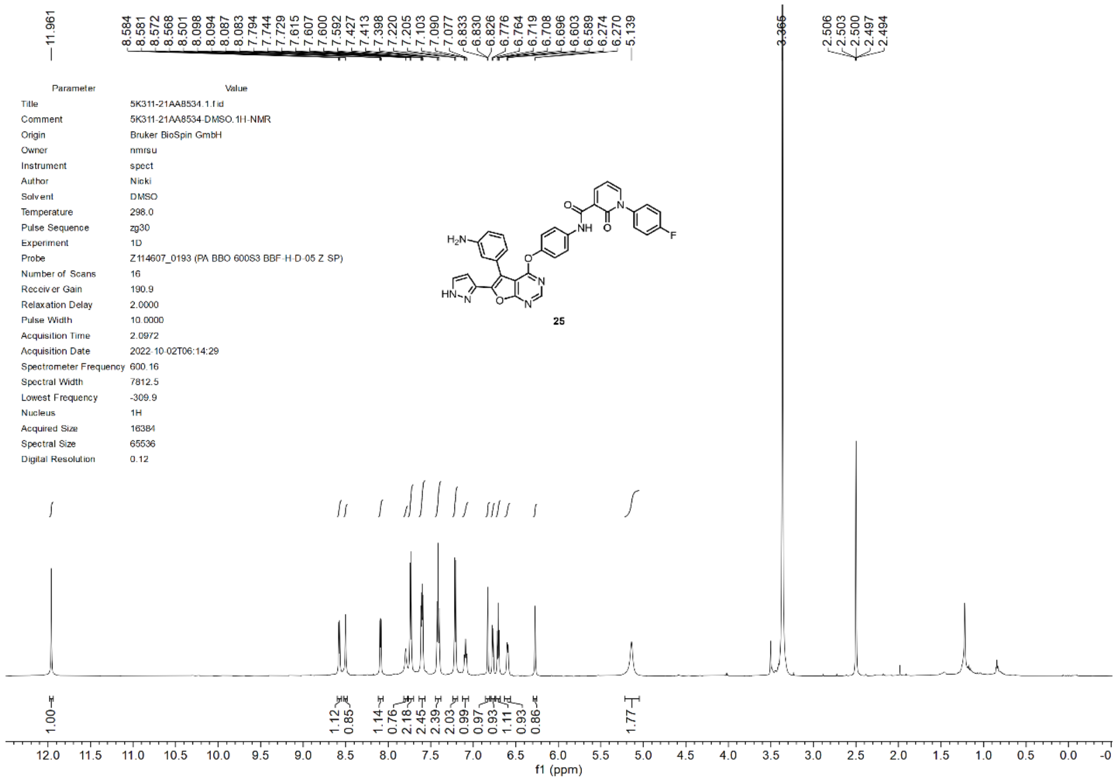
11.961
8.584 8.581 8.572 8.568 8.501 8.098 8.094 8.087 8.083 7.794 7.744 7.729 7.615 7.607 7.600 7.592 7.427 7.413 7.398 7.220 7.205 7.103 7.090 7.077 6.833 6.830 6.826 6.776 6.764 6.719 6.708 6.696 6.603 6.589 6.274 6.270 5.139
3.365
2.506 2.503 2.500 2.497 2.494
Parameter Value
Title 5K311-21AA8534.1.fid
Comment 5K311-21AA8534-DMSO.1H-NMR
Origin Bruker BioSpin GmbH
Owner nmrsu
Instrument spect
Author Nicki
Solvent DMSO
Temperature 298.0
Pulse Sequence zg30
Experiment 1D
Probe Z114607_0193 (PA BBO 600S3 BBF-H-D-05 Z SP)
Number of Scans 16
Receiver Gain 190.9
Relaxation Delay 2.0000
Pulse Width 10.0000
Acquisition Time 2.0972
Acquisition Date 2022-10-02T06:14:29
Spectrometer Frequency 600.16
Spectral Width 7812.5
Lowest Frequency -309.9
Nucleus 1H
Acquired Size 16384
Spectral Size 65536
Digital Resolution 0.12
H_2N
O
NH
N
F
HN
N
25
1.00 1.12 0.85 1.14 0.76 2.18 2.45 2.39 2.03 0.99 0.97 0.93 1.11 0.93 0.86 1.77
f1 (ppm)

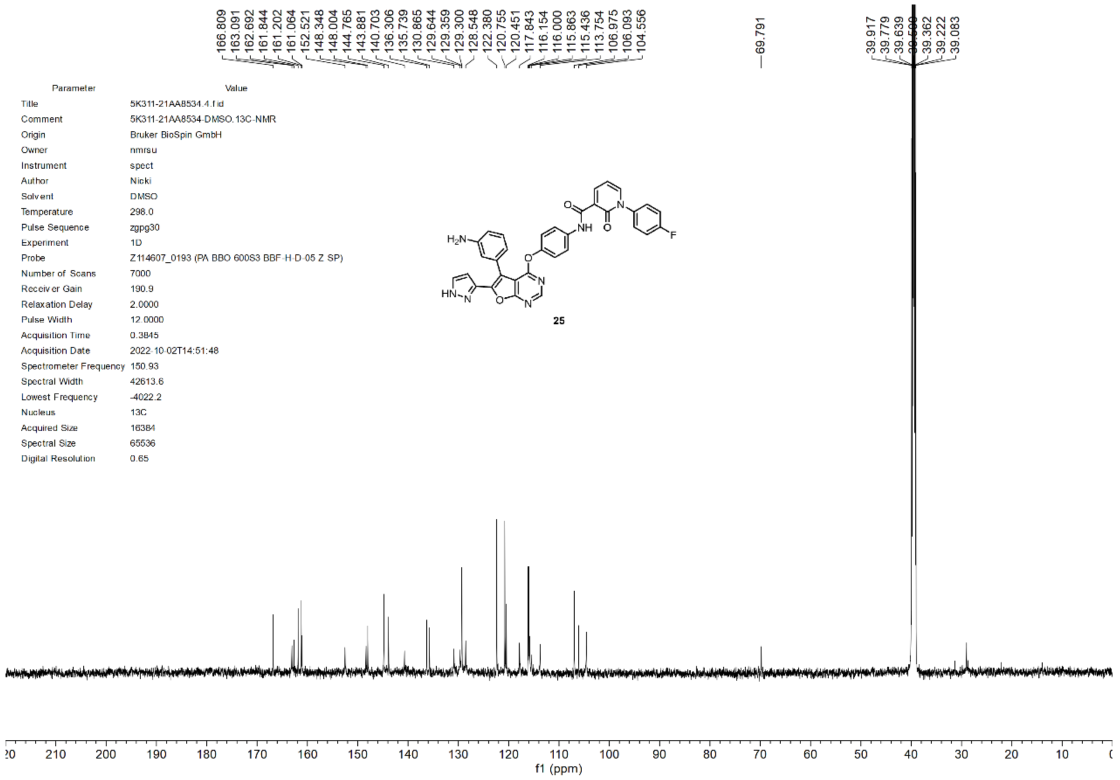
166.809 163.091 162.692 161.844 161.202 161.064 152.521 148.348 148.004 144.765 143.881 140.703 136.306 135.739 130.865 129.644 129.359 129.300 128.548 122.380 120.755 120.451 117.843 116.154 116.000 115.863 115.436 113.754 106.975 106.093 104.556
69.791
39.917 39.779 39.639 39.362 39.222 39.083
Parameter Value
Title 5K311-21AA8534.4.fid
Comment 5K311-21AA8534-DMSO.13C-NMR
Origin Bruker BioSpin GmbH
Owner nmrsu
Instrument spect
Author Nicki
Solvent DMSO
Temperature 298.0
Pulse Sequence zgpg30
Experiment 1D
Probe Z114607_0193 (PA BBO 600S3 BBF-H-D-05 Z SP)
Number of Scans 7000
Receiver Gain 190.9
Relaxation Delay 2.0000
Pulse Width 12.0000
Acquisition Time 0.3845
Acquisition Date 2022-10-02T14:51:48
Spectrometer Frequency 150.93
Spectral Width 42613.6
Lowest Frequency -4022.2
Nucleus 13C
Acquired Size 16384
Spectral Size 65536
Digital Resolution 0.65
H_2N
NH
F
25
f1 (ppm)

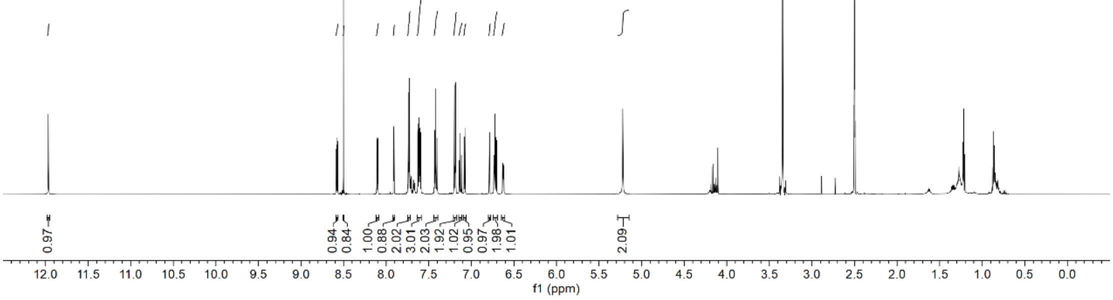

Parameter	Value
Title	900901-BPR5K275-09BP018160 1H.1.fid
Comment	BPR5K275-09BP018160 1H
Origin	Bruker BioSpin GmbH
Owner	nmrsu
Instrument	Avance
Author	Nicki
Solvent	DMSO
Temperature	294.7
Pulse Sequence	zg30
Experiment	1D
Probe	Z154705_0043 (PI HR-BBO600S3-BBF/ H/ D-5.0-Z SP)
Number of Scans	64
Receiver Gain	101.0
Relaxation Delay	1.0000
Pulse Width	10.6000
Acquisition Time	2.7525
Acquisition Date	2022-10-09T07:38:56
Spectrometer Frequency	600.14
Spectral Width	11904.8
Lowest Frequency	-2250.8
Nucleus	1H
Acquired Size	32768
Spectral Size	131072
Digital Resolution	0.09

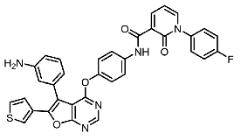

26

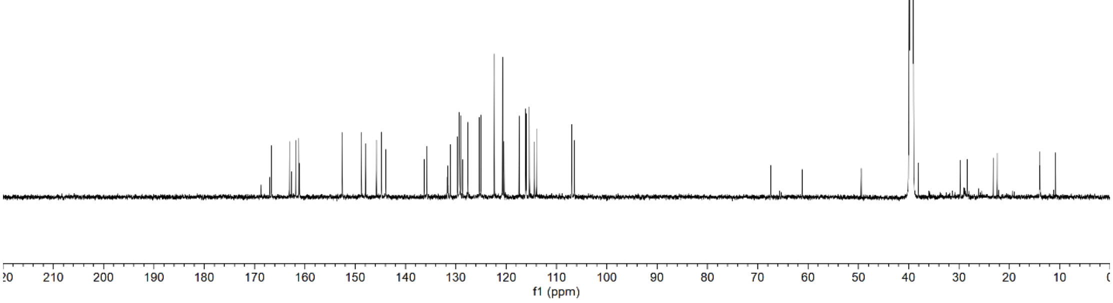

Parameter	Value
Title	900901-BPR5K275-09BP018160 13C.2.fid
Comment	BPR5K275-09BP018160 13C
Origin	Bruker BioSpin GmbH
Owner	nmrsu
Instrument	Avance
Author	Nicki
Solvent	DMSO
Temperature	294.7
Pulse Sequence	zgpg30
Experiment	1D
Probe	Z154705_0043 (PI HR-BBO600S3-BBF/ H/ D-5.0-Z SP)
Number of Scans	10000
Receiver Gain	90.5
Relaxation Delay	2.0000
Pulse Width	11.5000
Acquisition Time	0.9175
Acquisition Date	2022-10-09T15:57:07
Spectrometer Frequency	150.92
Spectral Width	35714.3
Lowest Frequency	-1329.1
Nucleus	13C
Acquired Size	32768
Spectral Size	65536
Digital Resolution	0.54

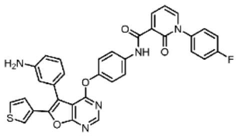

26

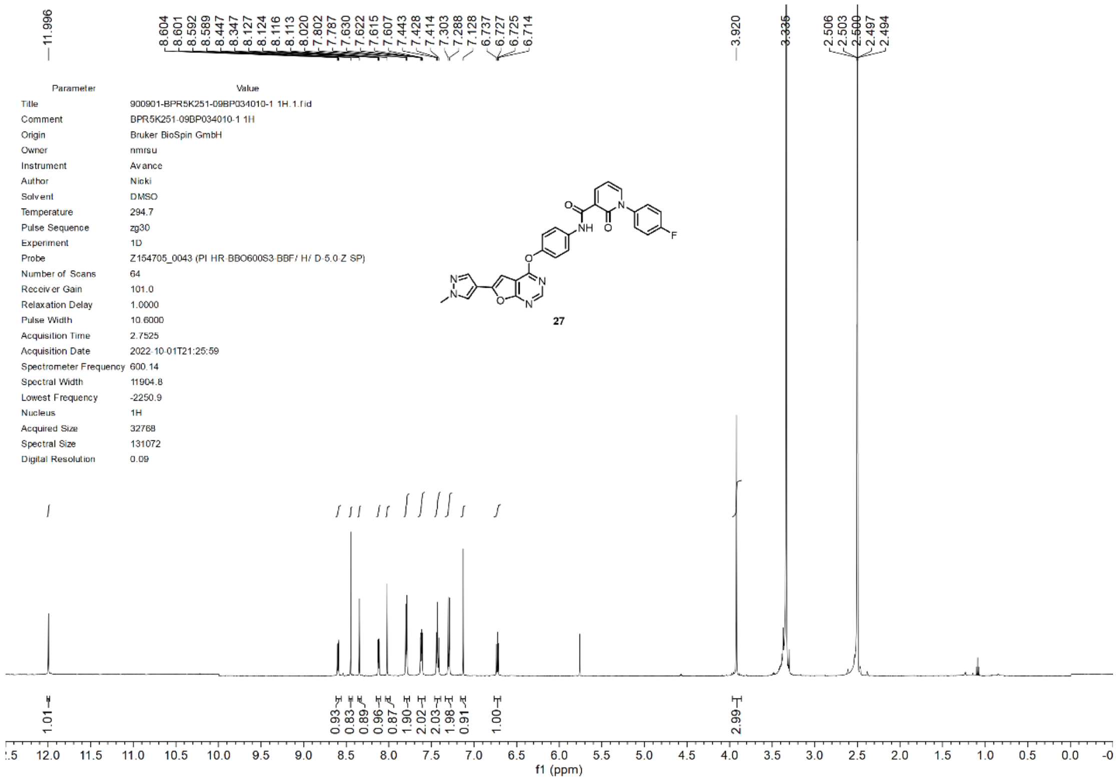

Parameter	Value
Title	900901-BPR5K251-09BP034010-1 1H.1.fid
Comment	BPR5K251-09BP034010-1 1H
Origin	Bruker BioSpin GmbH
Owner	nmrsu
Instrument	Avance
Author	Nicki
Solvent	DMSO
Temperature	294.7
Pulse Sequence	zg30
Experiment	1D
Probe	Z154705_0043 (PI HR-BBO600S3-BBF/ H/ D-5.0 Z SP)
Number of Scans	64
Receiver Gain	101.0
Relaxation Delay	1.0000
Pulse Width	10.6000
Acquisition Time	2.7525
Acquisition Date	2022-10-01T21:25:59
Spectrometer Frequency	600.14
Spectral Width	11904.8
Lowest Frequency	-2250.9
Nucleus	1H
Acquired Size	32768
Spectral Size	131072
Digital Resolution	0.09

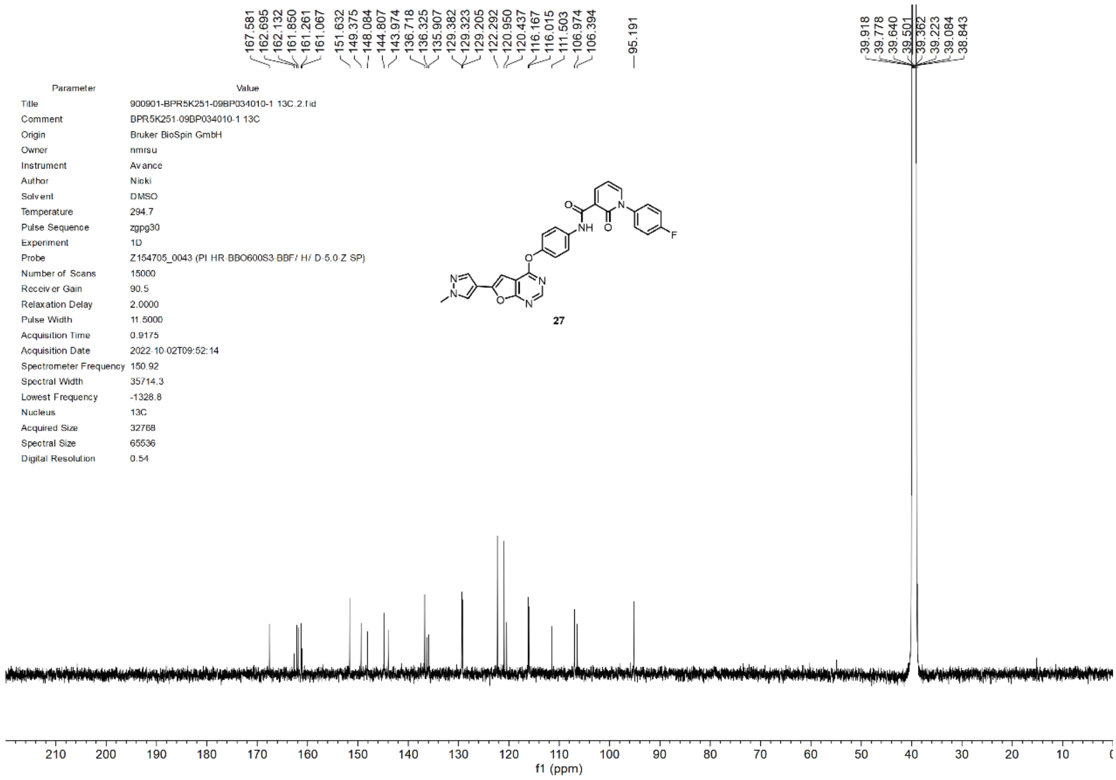

Parameter	Value
Title	900901-BPR5K251-09BP034010-1 13C.2.fid
Comment	BPR5K251-09BP034010-1 13C
Origin	Bruker BioSpin GmbH
Owner	nmrsu
Instrument	Avance
Author	Nicki
Solvent	DMSO
Temperature	294.7
Pulse Sequence	zgpg30
Experiment	1D
Probe	Z154705_0043 (PI HR-BBO600S3-BBF/ H/ D-5.0 Z SP)
Number of Scans	15000
Receiver Gain	90.5
Relaxation Delay	2.0000
Pulse Width	11.5000
Acquisition Time	0.9175
Acquisition Date	2022-10-02T09:52:14
Spectrometer Frequency	150.92
Spectral Width	35714.3
Lowest Frequency	-1328.8
Nucleus	13C
Acquired Size	32768
Spectral Size	65536
Digital Resolution	0.54

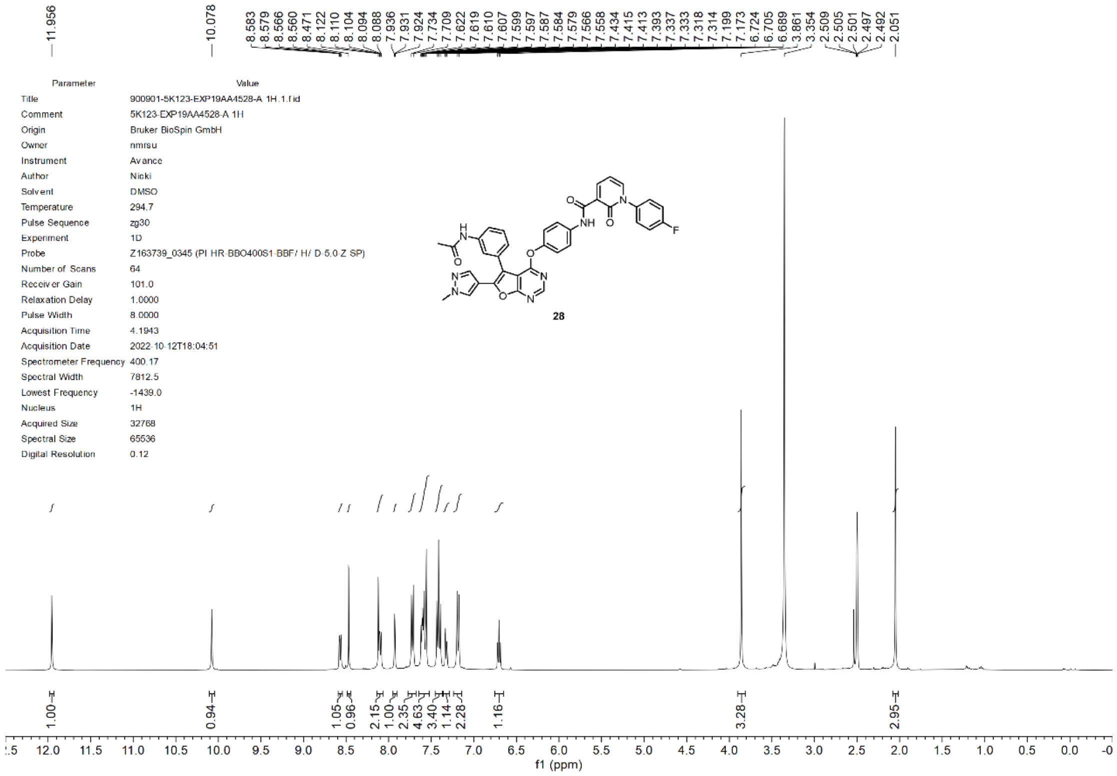
11.956
10.078
8.583
8.579
8.566
8.560
8.471
8.122
8.110
8.104
8.094
8.088
7.936
7.931
7.924
7.734
7.709
7.622
7.619
7.610
7.607
7.599
7.597
7.587
7.584
7.579
7.566
7.558
7.434
7.415
7.413
7.393
7.337
7.333
7.318
7.314
7.199
7.173
6.724
6.705
6.689
3.861
3.354
2.509
2.505
2.501
2.497
2.492
2.051
Parameter Value
Title 900901-5K123-EXP19AA4528-A 1H.1.fid
Comment 5K123-EXP19AA4528-A 1H
Origin Bruker BioSpin GmbH
Owner nmrsu
Instrument Avance
Author Nicki
Solvent DMSO
Temperature 294.7
Pulse Sequence zg30
Experiment 1D
Probe Z163739_0345 (PI HR-BBO400S1-BBF/ H/ D-5.0 Z SP)
Number of Scans 64
Receiver Gain 101.0
Relaxation Delay 1.0000
Pulse Width 8.0000
Acquisition Time 4.1943
Acquisition Date 2022-10-12T18:04:51
Spectrometer Frequency 400.17
Spectral Width 7812.5
Lowest Frequency -1439.0
Nucleus 1H
Acquired Size 32768
Spectral Size 65536
Digital Resolution 0.12
28
1.00
0.94
1.05
0.96
2.15
1.00
2.35
4.63
3.40
1.14
2.28
1.16
3.28
2.95
f1 (ppm)

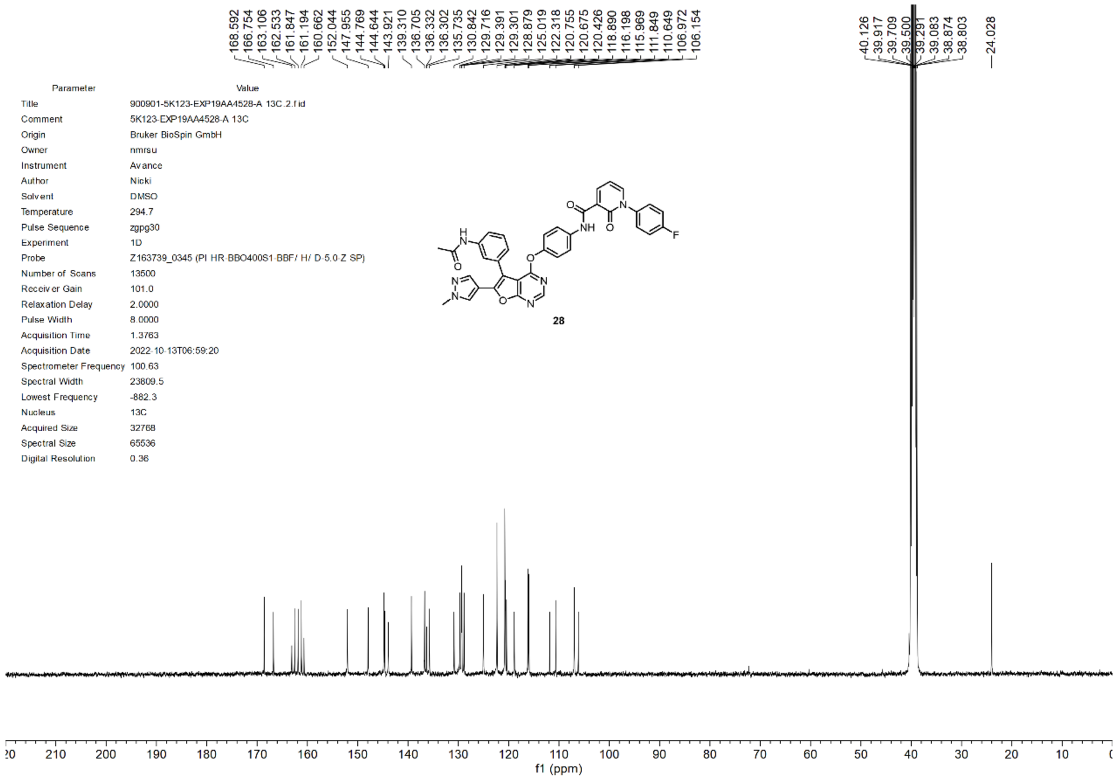
168.592
166.754
163.106
162.533
161.847
161.194
160.662
152.044
147.955
144.769
144.644
143.921
139.310
136.705
136.332
136.302
135.735
130.842
129.716
129.391
129.301
128.879
125.019
122.318
120.755
120.675
120.426
118.890
116.198
115.969
111.849
110.649
106.972
106.154
40.126
39.917
39.709
39.500
39.291
39.083
38.874
38.803
24.028
Parameter Value
Title 900901-5K123-EXP19AA4528-A 13C.2.fid
Comment 5K123-EXP19AA4528-A 13C
Origin Bruker BioSpin GmbH
Owner nmrsu
Instrument Avance
Author Nicki
Solvent DMSO
Temperature 294.7
Pulse Sequence zgpg30
Experiment 1D
Probe Z163739_0345 (PI HR-BBO400S1-BBF/ H/ D-5.0 Z SP)
Number of Scans 13500
Receiver Gain 101.0
Relaxation Delay 2.0000
Pulse Width 8.0000
Acquisition Time 1.3763
Acquisition Date 2022-10-13T06:59:20
Spectrometer Frequency 100.63
Spectral Width 23809.5
Lowest Frequency -882.3
Nucleus 13C
Acquired Size 32768
Spectral Size 65536
Digital Resolution 0.36
28
f1 (ppm)

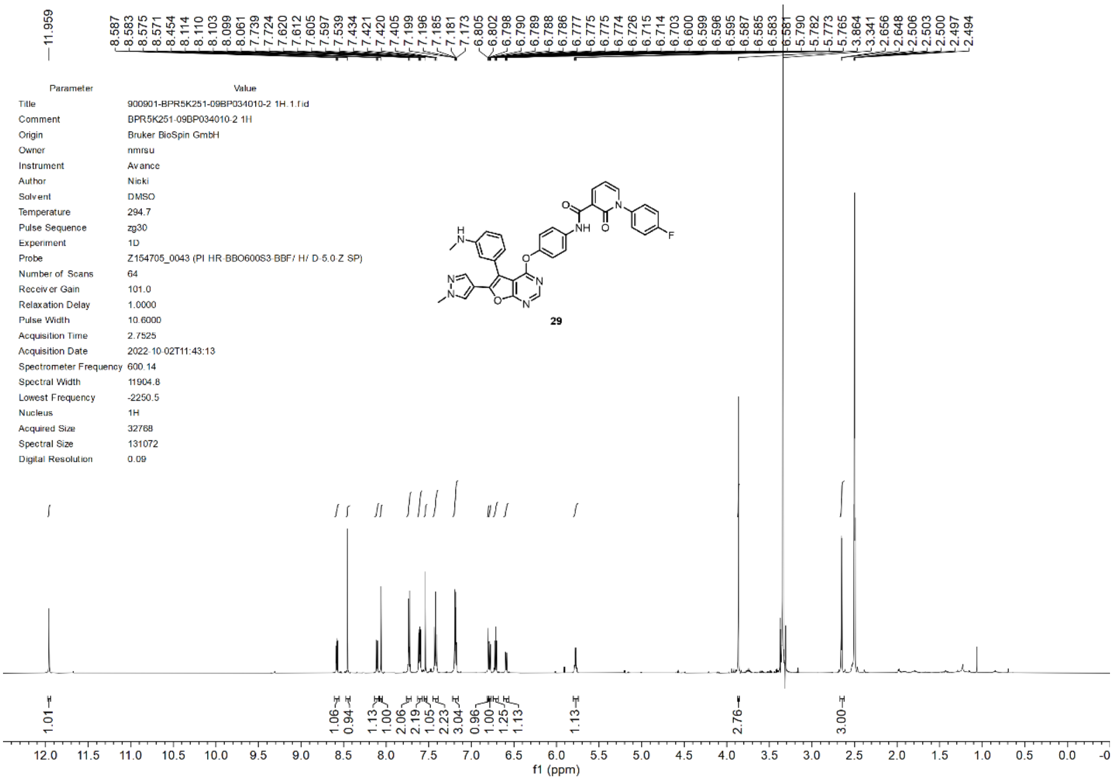

Parameter	Value
Title	900901-BPR5K251-09BP034010-2 1H.1.fid
Comment	BPR5K251-09BP034010-2 1H
Origin	Bruker BioSpin GmbH
Owner	nmrsu
Instrument	Avance
Author	Nicki
Solvent	DMSO
Temperature	294.7
Pulse Sequence	zg30
Experiment	1D
Probe	Z154705_0043 (PI HR-BBO600S3-BBF/ H/ D-5.0 Z SP)
Number of Scans	64
Receiver Gain	101.0
Relaxation Delay	1.0000
Pulse Width	10.6000
Acquisition Time	2.7525
Acquisition Date	2022-10-02T11:43:13
Spectrometer Frequency	600.14
Spectral Width	11904.8
Lowest Frequency	-2250.5
Nucleus	1H
Acquired Size	32768
Spectral Size	131072
Digital Resolution	0.09

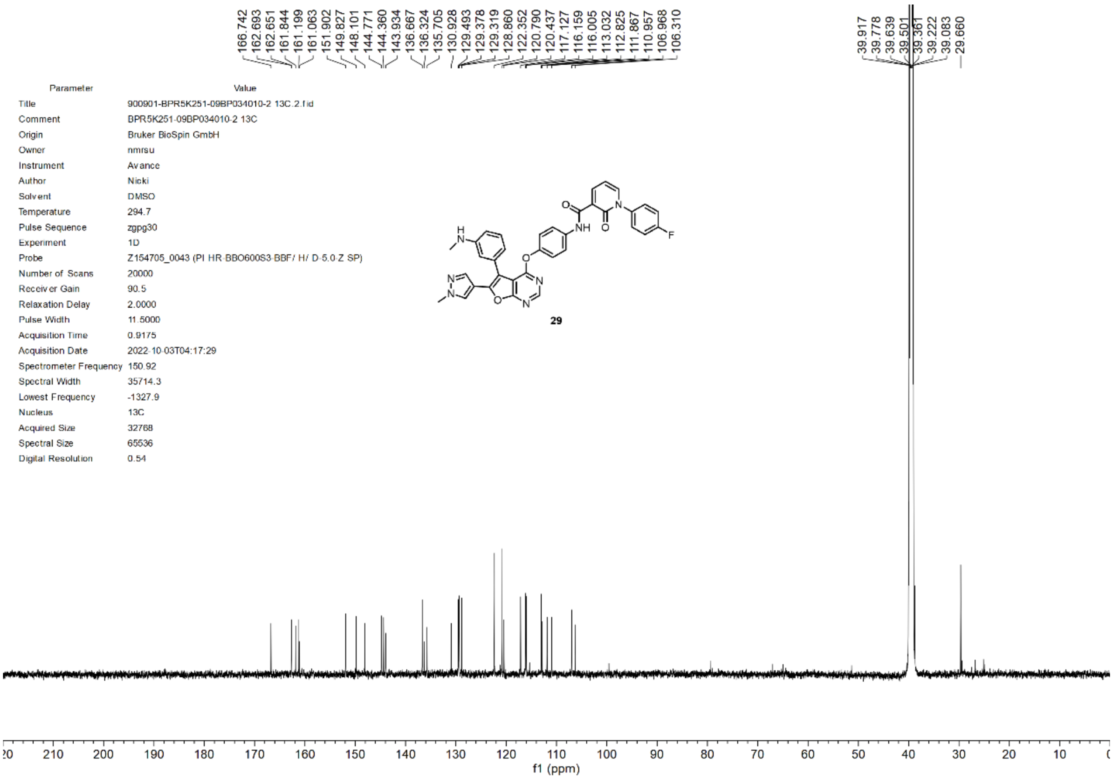

Parameter	Value
Title	900901-BPR5K251-09BP034010-2 13C.2.fid
Comment	BPR5K251-09BP034010-2 13C
Origin	Bruker BioSpin GmbH
Owner	nmrsu
Instrument	Avance
Author	Nicki
Solvent	DMSO
Temperature	294.7
Pulse Sequence	zgpg30
Experiment	1D
Probe	Z154705_0043 (PI HR-BBO600S3-BBF/ H/ D-5.0 Z SP)
Number of Scans	20000
Receiver Gain	90.5
Relaxation Delay	2.0000
Pulse Width	11.5000
Acquisition Time	0.9175
Acquisition Date	2022-10-03T04:17:29
Spectrometer Frequency	150.92
Spectral Width	35714.3
Lowest Frequency	-1327.9
Nucleus	13C
Acquired Size	32768
Spectral Size	65536
Digital Resolution	0.54

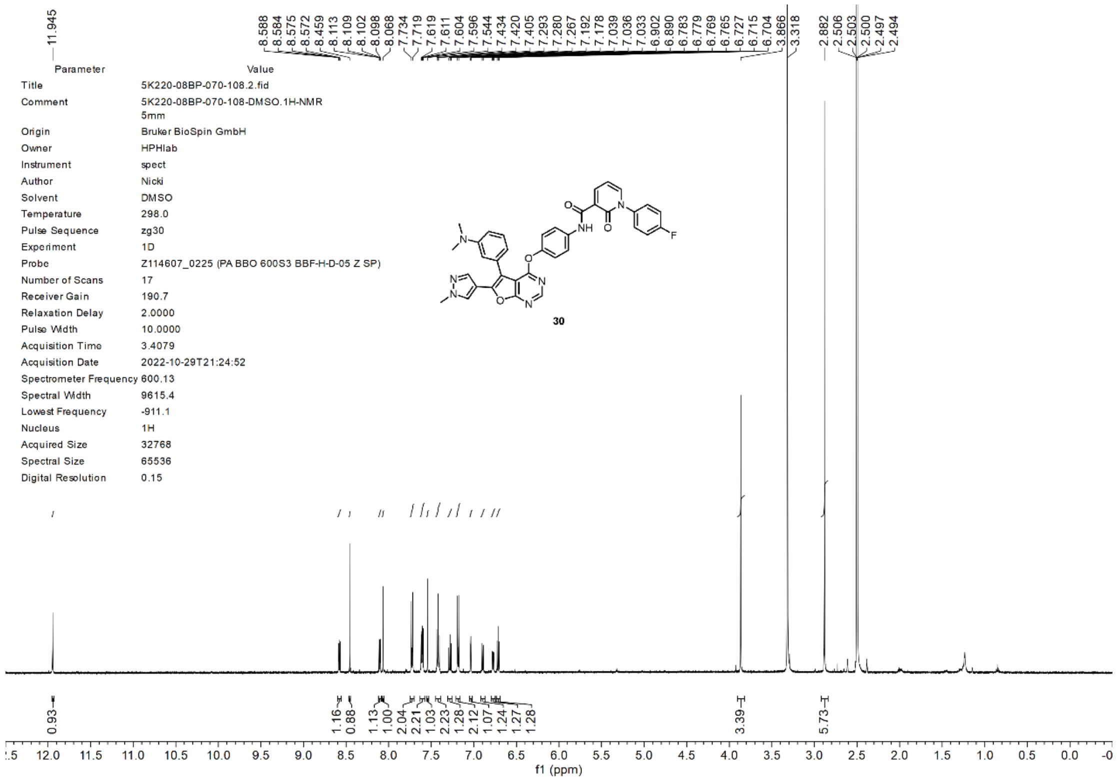

11.945
8.588 8.584 8.575 8.572 8.459 8.113 8.109 8.102 8.098 8.068 7.734 7.719 7.619 7.611 7.604 7.596 7.544 7.434 7.420 7.405 7.293 7.280 7.267 7.192 7.178 7.039 7.036 7.033 6.902 6.890 6.783 6.779 6.769 6.765 6.727 6.715 6.704 3.866 3.318
2.882 2.506 2.503 2.500 2.497 2.494
Parameter Value
Title 5K220-08BP-070-108.2.fid
Comment 5K220-08BP-070-108-DMSO.1H-NMR 5mm
Origin Bruker BioSpin GmbH
Owner HPHlab
Instrument spect
Author Nicki
Solvent DMSO
Temperature 298.0
Pulse Sequence zg30
Experiment 1D
Probe Z114607_0225 (PA BBO 600S3 BBF-H-D-05 Z SP)
Number of Scans 17
Receiver Gain 190.7
Relaxation Delay 2.0000
Pulse Width 10.0000
Acquisition Time 3.4079
Acquisition Date 2022-10-29T21:24:52
Spectrometer Frequency 600.13
Spectral Width 9615.4
Lowest Frequency -911.1
Nucleus 1H
Acquired Size 32768
Spectral Size 65536
Digital Resolution 0.15
30
0.93 1.16 0.88 1.13 1.00 2.04 2.21 1.03 2.23 1.28 2.12 1.07 1.24 1.27 1.28 3.39 5.73
f1 (ppm)

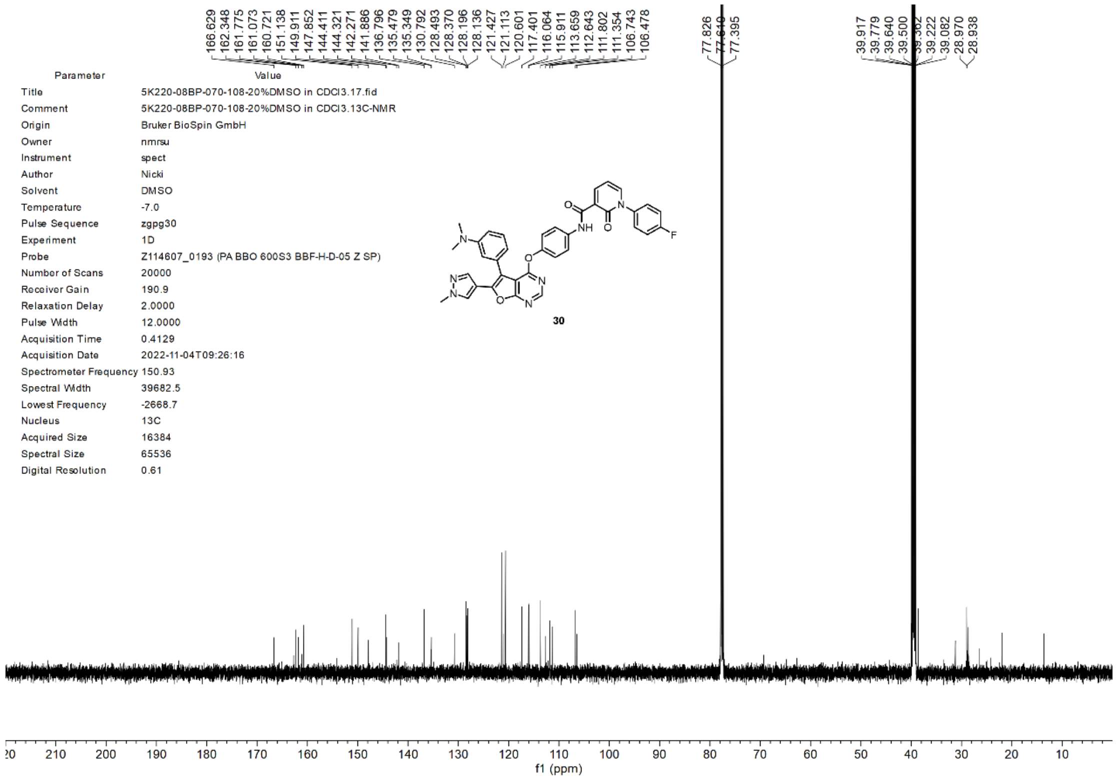

166.629 162.348 161.775 161.073 160.721 151.138 149.911 147.852 144.411 144.321 142.271 141.886 136.796 135.479 135.349 130.792 128.493 128.370 128.196 128.136 121.427 121.113 120.601 117.401 116.064 115.911 113.659 112.643 111.802 111.354 106.743 106.478
77.826 77.610 77.395
39.917 39.779 39.640 39.500 39.362 39.222 39.082 28.970 28.938
Parameter Value
Title 5K220-08BP-070-108-20%DMSO in CDCl3.17.fid
Comment 5K220-08BP-070-108-20%DMSO in CDCl3.13C-NMR
Origin Bruker BioSpin GmbH
Owner nmrsu
Instrument spect
Author Nicki
Solvent DMSO
Temperature -7.0
Pulse Sequence zgpg30
Experiment 1D
Probe Z114607_0193 (PA BBO 600S3 BBF-H-D-05 Z SP)
Number of Scans 20000
Receiver Gain 190.9
Relaxation Delay 2.0000
Pulse Width 12.0000
Acquisition Time 0.4129
Acquisition Date 2022-11-04T09:26:16
Spectrometer Frequency 150.93
Spectral Width 39682.5
Lowest Frequency -2668.7
Nucleus 13C
Acquired Size 16384
Spectral Size 65536
Digital Resolution 0.61
30
f1 (ppm)

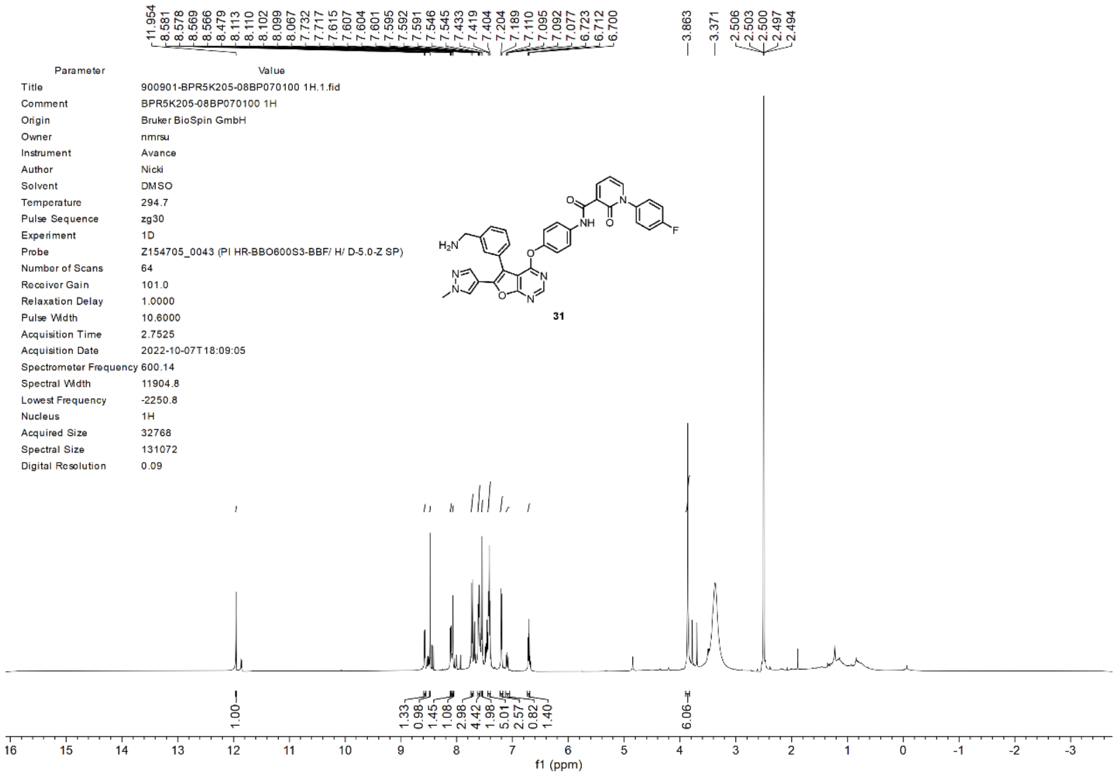

Parameter	Value
Title	900901-BPR5K205-08BP070100 1H.1.fid
Comment	BPR5K205-08BP070100 1H
Origin	Bruker BioSpin GmbH
Owner	nmrsu
Instrument	Avance
Author	Nicki
Solvent	DMSO
Temperature	294.7
Pulse Sequence	zg30
Experiment	1D
Probe	Z154705_0043 (PI HR-BBO600S3-BBF/ H/ D-5.0-Z SP)
Number of Scans	64
Receiver Gain	101.0
Relaxation Delay	1.0000
Pulse Width	10.6000
Acquisition Time	2.7525
Acquisition Date	2022-10-07T18:09:05
Spectrometer Frequency	600.14
Spectral Width	11904.8
Lowest Frequency	-2250.8
Nucleus	1H
Acquired Size	32768
Spectral Size	131072
Digital Resolution	0.09

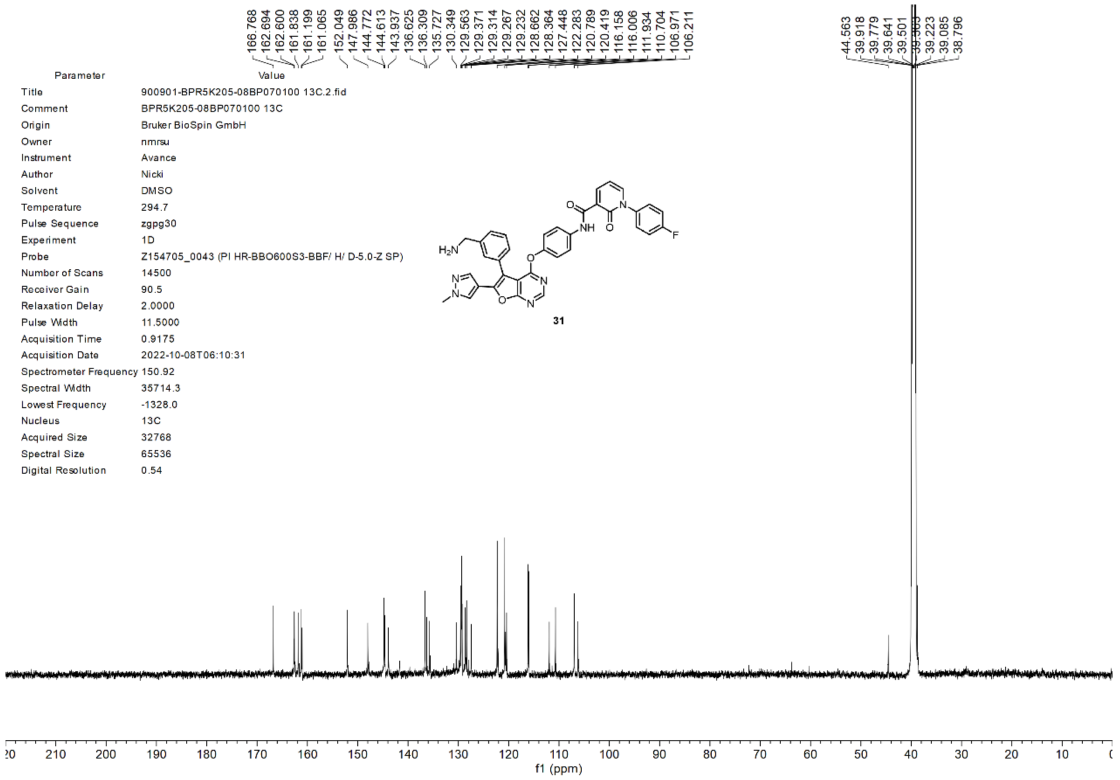

Parameter	Value
Title	900901-BPR5K205-08BP070100 13C.2.fid
Comment	BPR5K205-08BP070100 13C
Origin	Bruker BioSpin GmbH
Owner	nmrsu
Instrument	Avance
Author	Nicki
Solvent	DMSO
Temperature	294.7
Pulse Sequence	zgpg30
Experiment	1D
Probe	Z154705_0043 (PI HR-BBO600S3-BBF/ H/ D-5.0-Z SP)
Number of Scans	14500
Receiver Gain	90.5
Relaxation Delay	2.0000
Pulse Width	11.5000
Acquisition Time	0.9175
Acquisition Date	2022-10-08T06:10:31
Spectrometer Frequency	150.92
Spectral Width	35714.3
Lowest Frequency	-1328.0
Nucleus	13C
Acquired Size	32768
Spectral Size	65536
Digital Resolution	0.54

Parameter	Value
Title	900901-BPR5K249-08BP070178 1H.1.fid
Comment	BPR5K249-08BP070178 1H
Origin	Bruker BioSpin GmbH
Owner	nmrsu
Instrument	Avance
Author	Nicki
Solvent	DMSO
Temperature	294.7
Pulse Sequence	zg30
Experiment	1D
Probe	Z154705_0043 (PI HR-BBO600S3-BBF/ H/ D-5.0 Z SP)
Number of Scans	64
Receiver Gain	101.0
Relaxation Delay	1.0000
Pulse Width	10.6000
Acquisition Time	2.7525
Acquisition Date	2022-10-01T07:08:16
Spectrometer Frequency	600.14
Spectral Width	11904.8
Lowest Frequency	-2250.9
Nucleus	1H
Acquired Size	32768
Spectral Size	131072
Digital Resolution	0.09

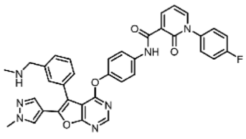

32

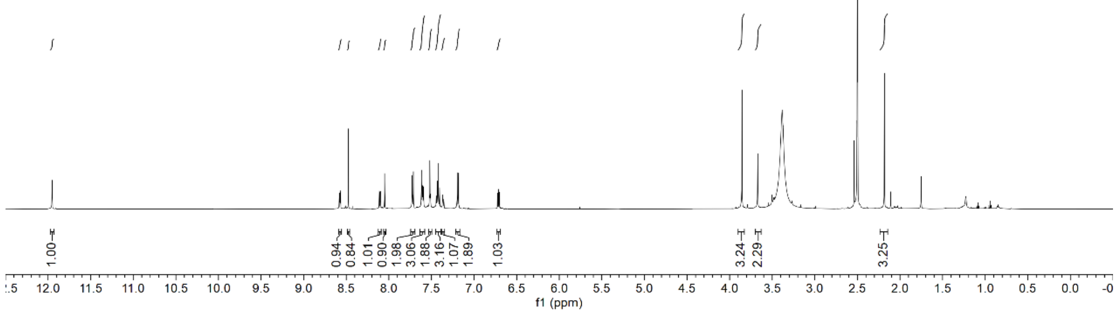

Parameter	Value
Title	900901-BPR5K249-08BP070178 13C.2.fid
Comment	BPR5K249-08BP070178 13C
Origin	Bruker BioSpin GmbH
Owner	nmrsu
Instrument	Avance
Author	Nicki
Solvent	DMSO
Temperature	294.7
Pulse Sequence	zgpg30
Experiment	1D
Probe	Z154705_0043 (PI HR-BBO600S3-BBF/ H/ D-5.0 Z SP)
Number of Scans	15000
Receiver Gain	90.5
Relaxation Delay	2.0000
Pulse Width	11.5000
Acquisition Time	0.9175
Acquisition Date	2022-10-01T19:34:31
Spectrometer Frequency	150.92
Spectral Width	35714.3
Lowest Frequency	-1327.6
Nucleus	13C
Acquired Size	32768
Spectral Size	65536
Digital Resolution	0.54

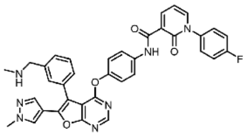

32

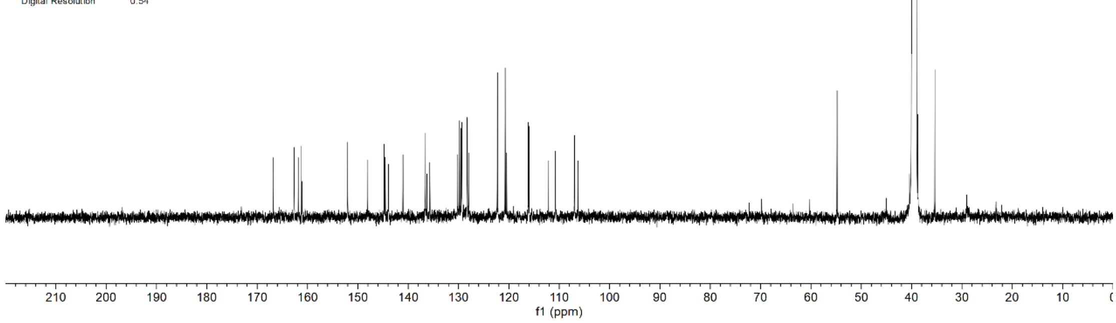

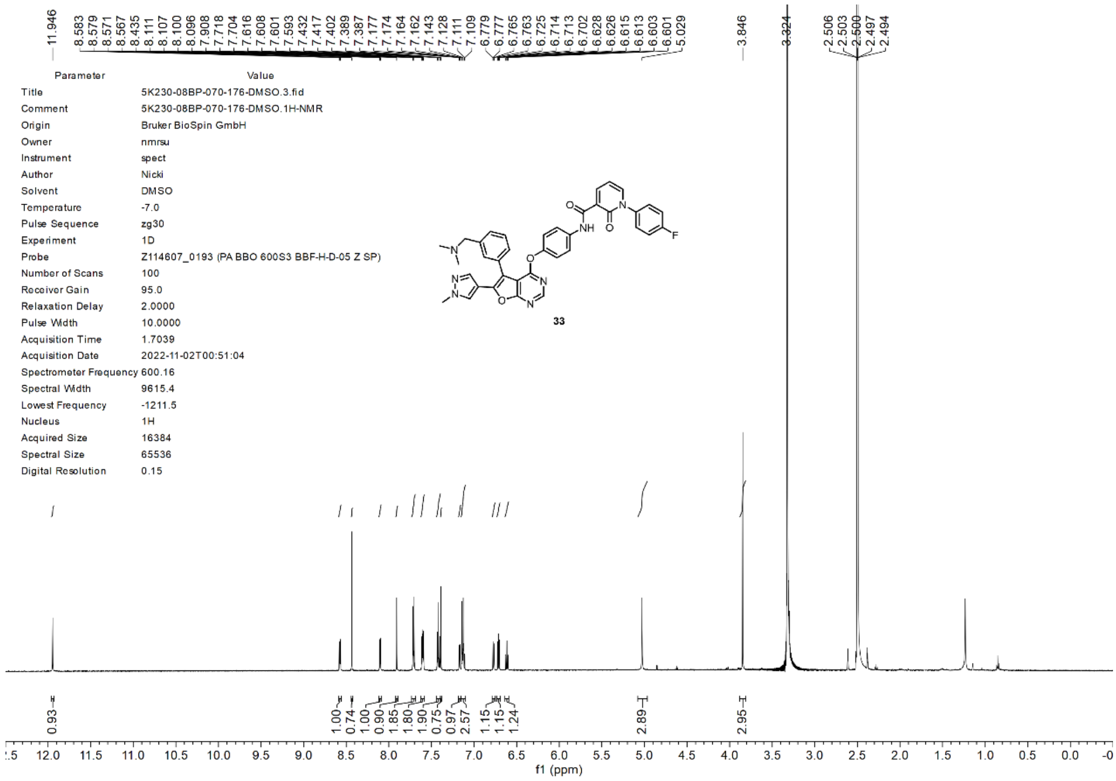
11.946
8.583 8.579 8.571 8.567 8.435 8.111 8.107 8.100 8.096 7.908 7.718 7.704 7.616 7.608 7.601 7.593 7.432 7.417 7.402 7.389 7.387 7.177 7.174 7.164 7.162 7.143 7.128 7.111 7.109 6.779 6.777 6.765 6.763 6.725 6.714 6.713 6.702 6.628 6.626 6.615 6.613 6.603 6.601 5.029
3.846
3.324
2.506 2.503 2.500 2.497 2.494
Parameter Value
Title 5K230-08BP-070-176-DMSO.3.fid
Comment 5K230-08BP-070-176-DMSO.1H-NMR
Origin Bruker BioSpin GmbH
Owner nmrsu
Instrument spect
Author Nicki
Solvent DMSO
Temperature -7.0
Pulse Sequence zg30
Experiment 1D
Probe Z114607_0193 (PA BBO 600S3 BBF-H-D-05 Z SP)
Number of Scans 100
Receiver Gain 95.0
Relaxation Delay 2.0000
Pulse Width 10.0000
Acquisition Time 1.7039
Acquisition Date 2022-11-02T00:51:04
Spectrometer Frequency 600.16
Spectral Width 9615.4
Lowest Frequency -1211.5
Nucleus 1H
Acquired Size 16384
Spectral Size 65536
Digital Resolution 0.15
33
0.93 1.00 0.74 1.00 0.90 1.85 1.80 1.90 0.75 0.97 2.57 1.15 1.15 1.24 2.89 2.95
12.0 11.5 11.0 10.5 10.0 9.5 9.0 8.5 8.0 7.5 7.0 6.5 6.0 5.5 5.0 4.5 4.0 3.5 3.0 2.5 2.0 1.5 1.0 0.5 0.0
f1 (ppm)

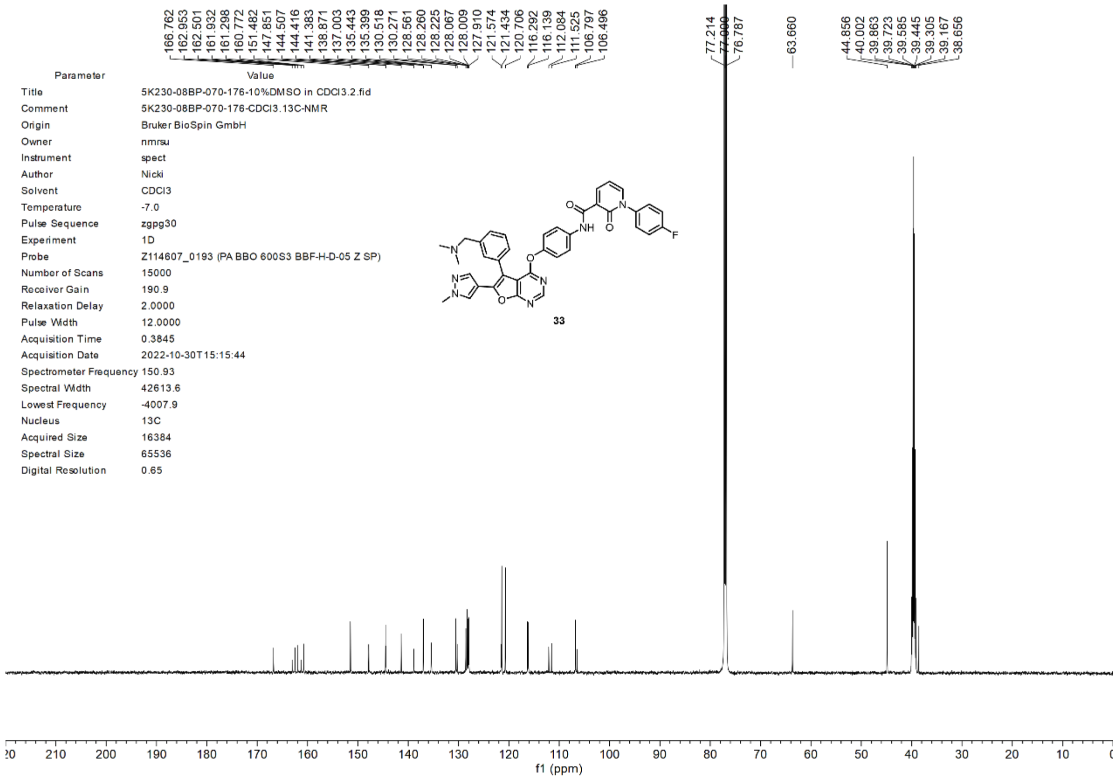
166.762 162.953 162.501 161.932 161.298 160.772 151.482 147.851 144.507 144.416 141.383 138.871 137.003 135.443 135.399 130.518 130.271 128.561 128.260 128.225 128.067 128.009 127.910 121.574 121.434 120.706 116.292 116.139 112.084 111.525 106.797 106.496
77.214 77.000 76.787
63.660
44.856 40.002 39.863 39.723 39.585 39.445 39.305 39.167 38.656
Parameter Value
Title 5K230-08BP-070-176-10%DMSO in CDCl3.2.fid
Comment 5K230-08BP-070-176-CDCl3.13C-NMR
Origin Bruker BioSpin GmbH
Owner nmrsu
Instrument spect
Author Nicki
Solvent CDCl3
Temperature -7.0
Pulse Sequence zgpg30
Experiment 1D
Probe Z114607_0193 (PA BBO 600S3 BBF-H-D-05 Z SP)
Number of Scans 15000
Receiver Gain 190.9
Relaxation Delay 2.0000
Pulse Width 12.0000
Acquisition Time 0.3845
Acquisition Date 2022-10-30T15:15:44
Spectrometer Frequency 150.93
Spectral Width 42613.6
Lowest Frequency -4007.9
Nucleus 13C
Acquired Size 16384
Spectral Size 65536
Digital Resolution 0.65
33
210 200 190 180 170 160 150 140 130 120 110 100 90 80 70 60 50 40 30 20 10
f1 (ppm)

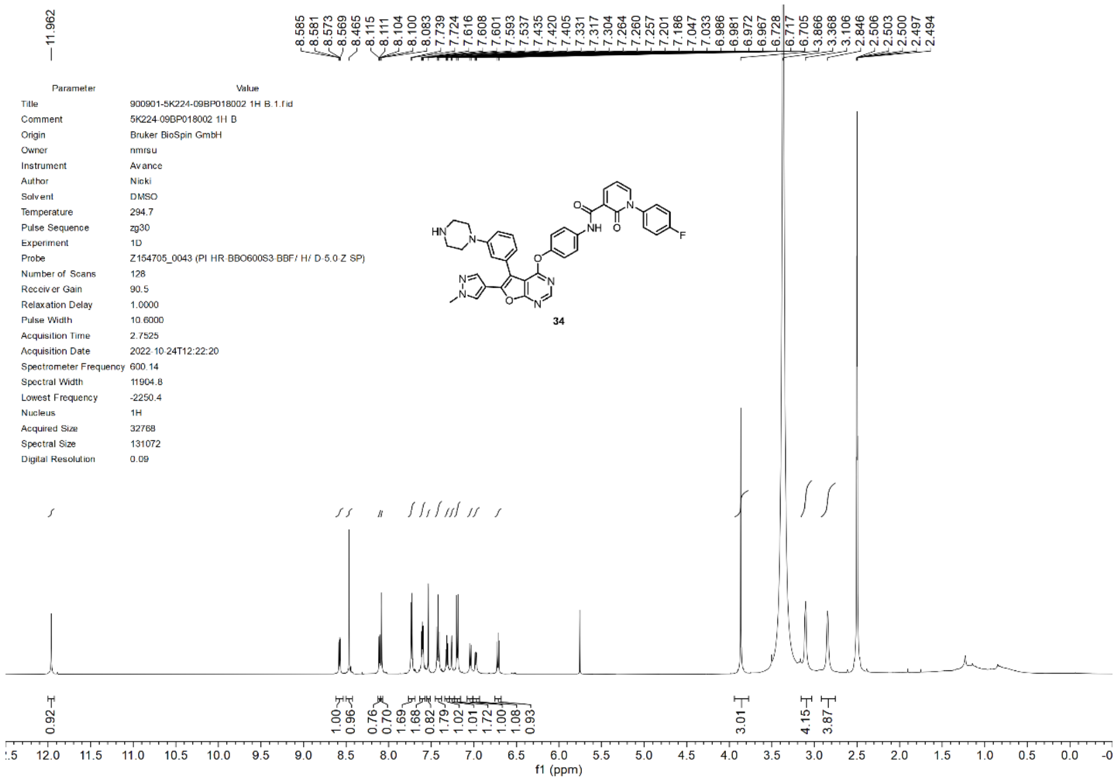
11.962
8.585
8.581
8.573
8.569
8.465
8.115
8.111
8.104
8.100
8.083
7.739
7.724
7.616
7.608
7.601
7.593
7.537
7.435
7.420
7.405
7.331
7.317
7.304
7.264
7.260
7.257
7.201
7.186
7.047
7.033
6.986
6.981
6.972
6.967
6.728
6.717
6.705
3.866
3.368
3.106
2.846
2.506
2.503
2.500
2.497
2.494
Parameter Value
Title 900901-5K224-09BP018002 1H B.1.fid
Comment 5K224-09BP018002 1H B
Origin Bruker BioSpin GmbH
Owner nmrsu
Instrument Avance
Author Nicki
Solvent DMSO
Temperature 294.7
Pulse Sequence zg30
Experiment 1D
Probe Z154705_0043 (PI HR-BBO600S3-BBF/ H/ D-5.0 Z SP)
Number of Scans 128
Receiver Gain 90.5
Relaxation Delay 1.0000
Pulse Width 10.6000
Acquisition Time 2.7525
Acquisition Date 2022-10-24T12:22:20
Spectrometer Frequency 600.14
Spectral Width 11904.8
Lowest Frequency -2250.4
Nucleus 1H
Acquired Size 32768
Spectral Size 131072
Digital Resolution 0.09
HN
N
O
NH
F
34
0.92
1.00
0.96
0.76
0.70
1.69
1.68
0.82
1.79
1.02
1.01
1.72
1.00
1.08
0.93
3.01
4.15
3.87
f1 (ppm)

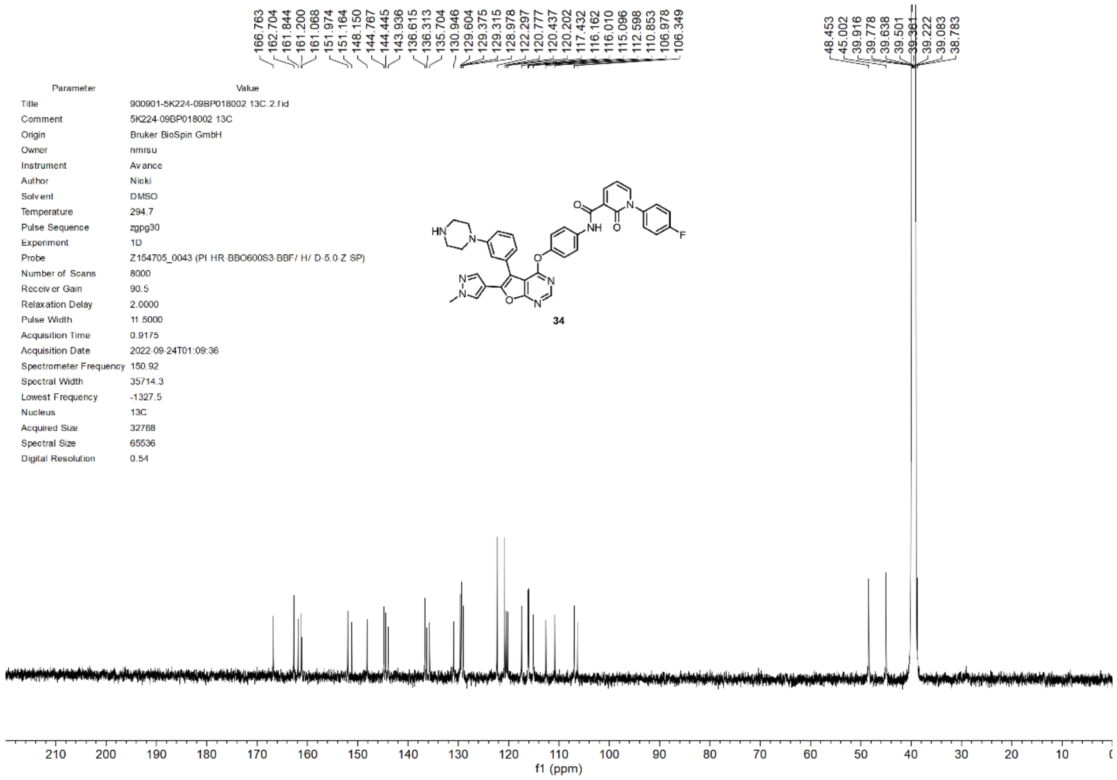
166.763
162.704
161.844
161.200
161.068
151.974
151.164
148.150
144.767
144.445
143.936
136.615
136.313
135.704
130.946
129.604
129.375
129.315
128.978
122.297
120.777
120.437
120.202
117.432
116.162
116.010
115.096
112.598
110.853
106.978
106.349
48.453
45.002
39.916
39.778
39.638
39.501
39.361
39.222
39.083
38.783
Parameter Value
Title 900901-5K224-09BP018002 13C.2.fid
Comment 5K224-09BP018002 13C
Origin Bruker BioSpin GmbH
Owner nmrsu
Instrument Avance
Author Nicki
Solvent DMSO
Temperature 294.7
Pulse Sequence zgpg30
Experiment 1D
Probe Z154705_0043 (PI HR-BBO600S3-BBF/ H/ D-5.0 Z SP)
Number of Scans 8000
Receiver Gain 90.5
Relaxation Delay 2.0000
Pulse Width 11.5000
Acquisition Time 0.9175
Acquisition Date 2022-09-24T01:09:36
Spectrometer Frequency 150.92
Spectral Width 35714.3
Lowest Frequency -1327.5
Nucleus 13C
Acquired Size 32768
Spectral Size 65536
Digital Resolution 0.54
HN
N
O
NH
F
34
f1 (ppm)

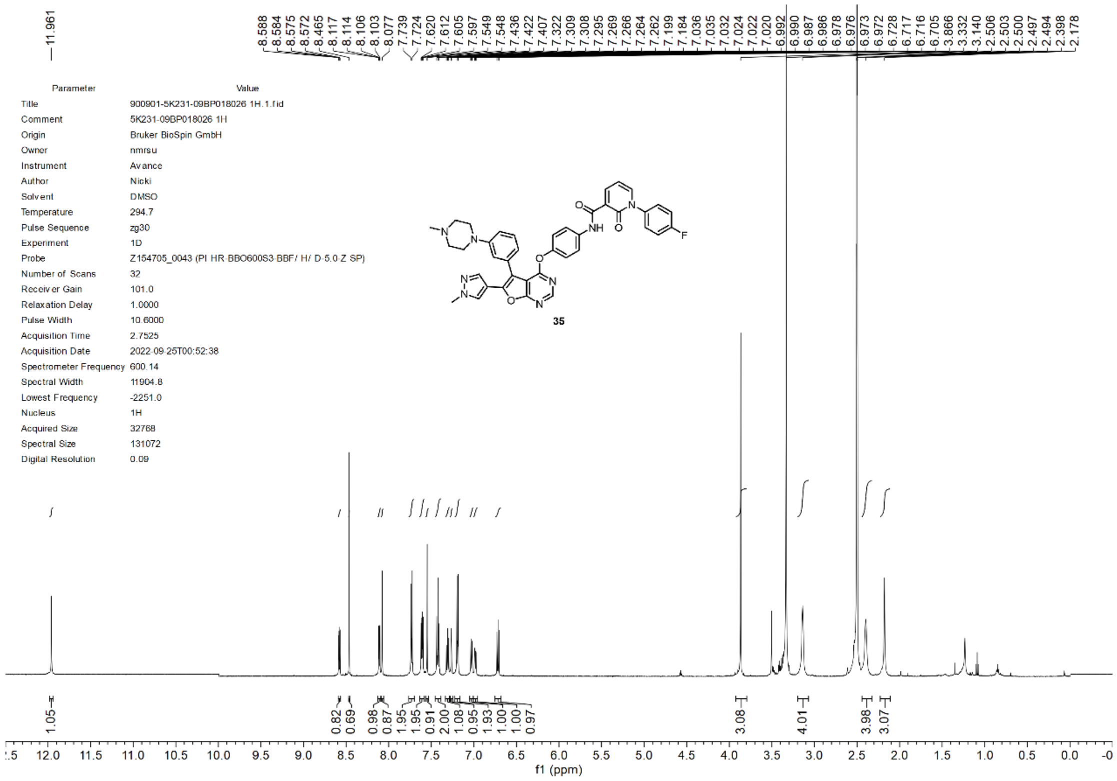
11.961
8.588
8.584
8.575
8.572
8.465
8.117
8.114
8.106
8.103
8.077
7.739
7.724
7.620
7.612
7.605
7.597
7.549
7.548
7.436
7.422
7.407
7.322
7.309
7.308
7.295
7.269
7.266
7.264
7.262
7.199
7.184
7.036
7.035
7.032
7.024
7.022
7.020
6.992
6.990
6.987
6.986
6.978
6.976
6.973
6.972
6.728
6.717
6.716
6.705
3.866
3.332
3.140
2.506
2.503
2.500
2.497
2.494
2.398
2.178
Parameter | Value
Title | 900901-5K231-09BP018026 1H.1.fid
Comment | 5K231-09BP018026 1H
Origin | Bruker BioSpin GmbH
Owner | nmrsu
Instrument | Avance
Author | Nicki
Solvent | DMSO
Temperature | 294.7
Pulse Sequence | zg30
Experiment | 1D
Probe | Z154705_0043 (PI HR BBO600S3-BBF/ H/ D-5.0 Z SP)
Number of Scans | 32
Receiver Gain | 101.0
Relaxation Delay | 1.0000
Pulse Width | 10.6000
Acquisition Time | 2.7525
Acquisition Date | 2022-09-25T00:52:38
Spectrometer Frequency | 600.14
Spectral Width | 11904.8
Lowest Frequency | -2251.0
Nucleus | 1H
Acquired Size | 32768
Spectral Size | 131072
Digital Resolution | 0.09
O
NH
O
N
F
N
N
O
N
N
N
N
O
35
1.05
0.82
0.69
0.98
0.87
1.95
1.95
2.00
0.91
1.08
0.95
1.93
1.00
1.00
0.97
3.08
4.01
3.98
3.07
f1 (ppm)

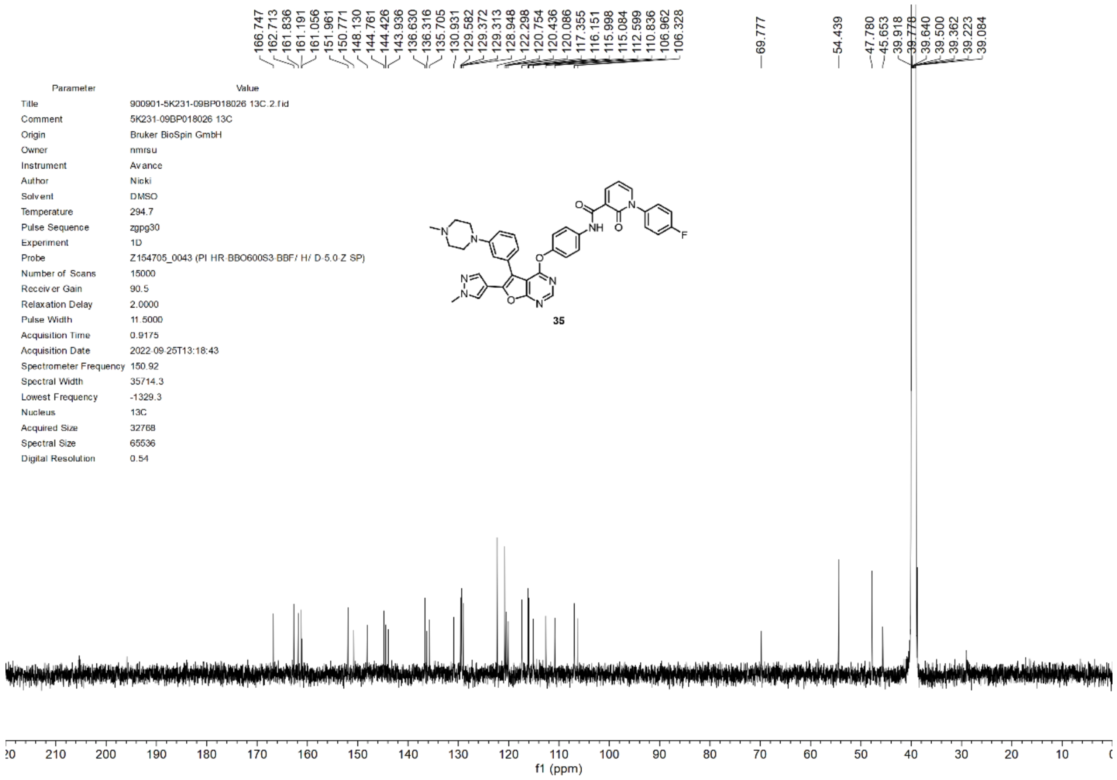
166.747
162.713
161.836
161.191
161.056
151.961
150.771
148.130
144.761
144.426
143.936
136.630
136.316
135.705
130.931
129.582
129.372
129.313
128.948
122.298
120.754
120.436
120.086
117.355
116.151
115.998
115.084
112.599
110.836
106.962
106.328
69.777
54.439
47.780
45.653
39.918
39.778
39.640
39.500
39.362
39.223
39.084
Parameter | Value
Title | 900901-5K231-09BP018026 13C.2.fid
Comment | 5K231-09BP018026 13C
Origin | Bruker BioSpin GmbH
Owner | nmrsu
Instrument | Avance
Author | Nicki
Solvent | DMSO
Temperature | 294.7
Pulse Sequence | zgpg30
Experiment | 1D
Probe | Z154705_0043 (PI HR BBO600S3-BBF/ H/ D-5.0 Z SP)
Number of Scans | 15000
Receiver Gain | 90.5
Relaxation Delay | 2.0000
Pulse Width | 11.5000
Acquisition Time | 0.9175
Acquisition Date | 2022-09-25T13:18:43
Spectrometer Frequency | 150.92
Spectral Width | 35714.3
Lowest Frequency | -1329.3
Nucleus | 13C
Acquired Size | 32768
Spectral Size | 65536
Digital Resolution | 0.54
35
f1 (ppm)

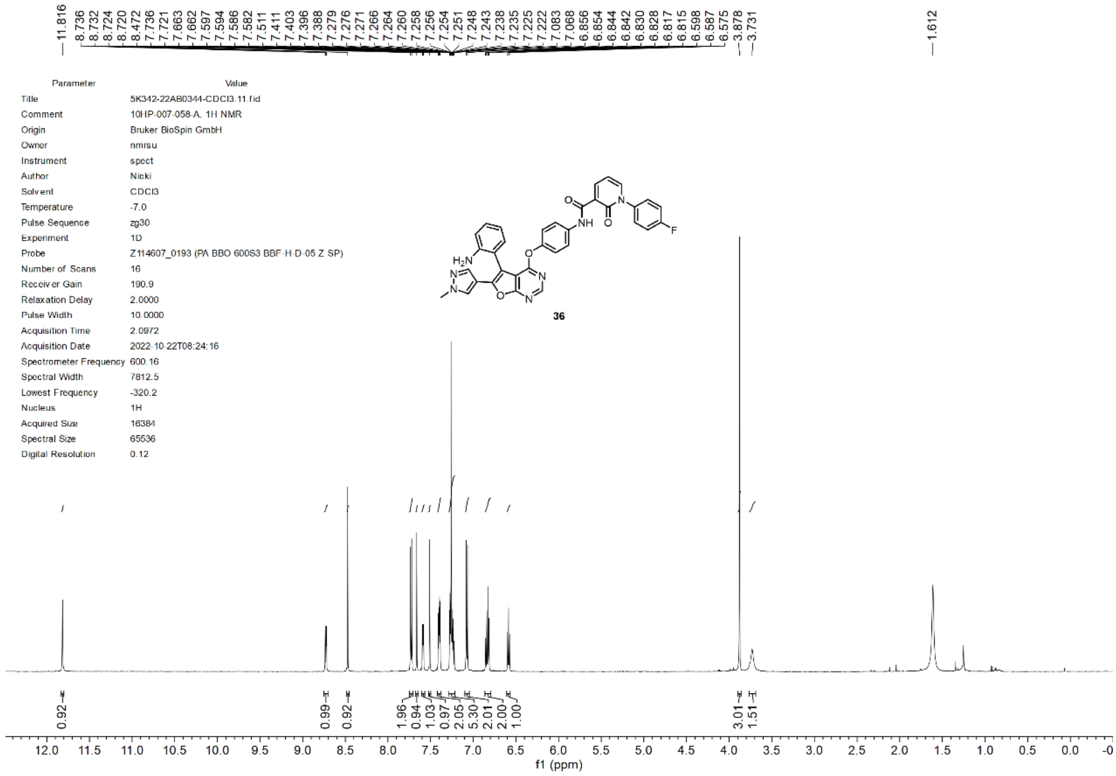
11.816
8.736
8.732
8.724
8.720
8.472
7.736
7.721
7.663
7.662
7.597
7.594
7.586
7.582
7.511
7.411
7.403
7.396
7.388
7.279
7.276
7.271
7.266
7.264
7.260
7.258
7.256
7.254
7.251
7.248
7.243
7.238
7.235
7.225
7.222
7.083
7.068
6.856
6.854
6.844
6.842
6.830
6.828
6.817
6.815
6.598
6.587
6.575
3.878
3.731
1.612
Parameter Value
Title 5K342-22AB0344-CDCl3.11.fid
Comment 10HP-007-058-A. 1H NMR
Origin Bruker BioSpin GmbH
Owner nmrsu
Instrument spect
Author Nicki
Solvent CDCl3
Temperature -7.0
Pulse Sequence zg30
Experiment 1D
Probe Z114607_0193 (PA BBO 600S3 BBF-H-D-05 Z SP)
Number of Scans 16
Receiver Gain 190.9
Relaxation Delay 2.0000
Pulse Width 10.0000
Acquisition Time 2.0972
Acquisition Date 2022-10-22T08:24:16
Spectrometer Frequency 600.16
Spectral Width 7812.5
Lowest Frequency -320.2
Nucleus 1H
Acquired Size 16384
Spectral Size 65536
Digital Resolution 0.12
36
0.92
0.99
0.92
1.96
0.94
1.03
0.97
2.05
5.30
2.01
2.00
1.00
3.01
1.51
f1 (ppm)

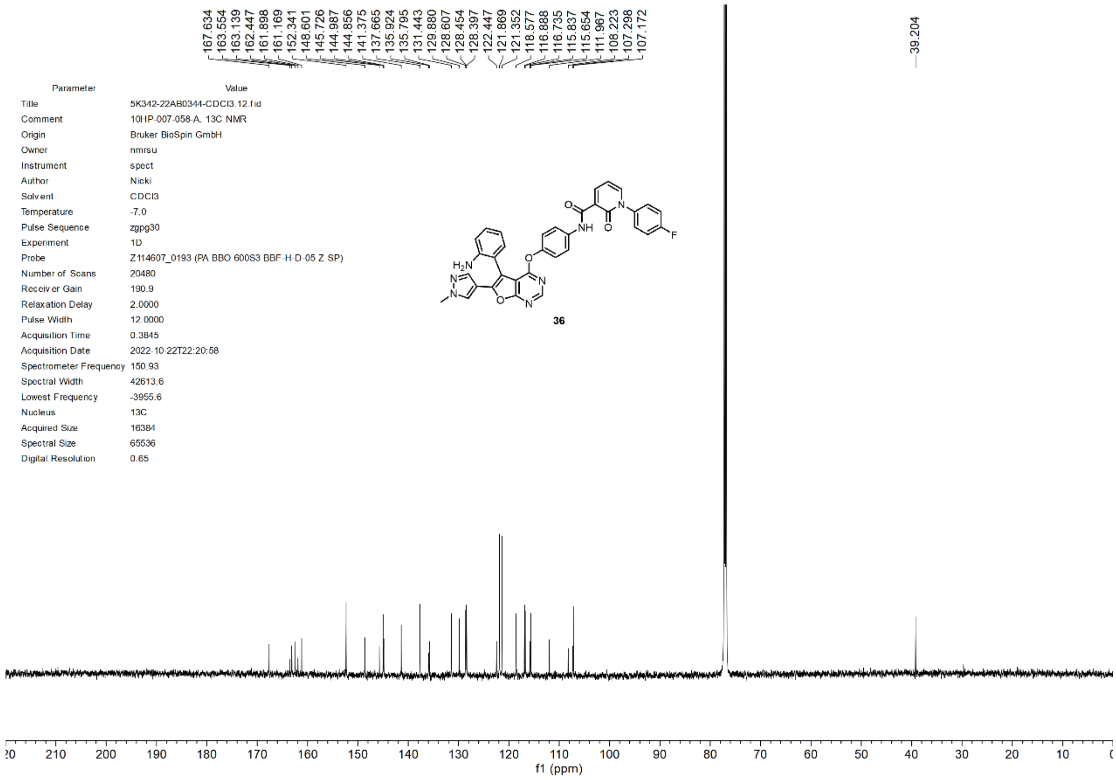
167.634
163.554
163.139
162.447
161.898
161.169
152.341
148.601
145.726
144.987
144.856
141.375
137.665
135.924
135.795
131.443
129.880
128.607
128.454
128.397
122.447
121.869
121.352
118.577
116.888
116.735
115.837
115.654
111.967
108.223
107.298
107.172
39.204
Parameter Value
Title 5K342-22AB0344-CDCl3.12.fid
Comment 10HP-007-058-A. 13C NMR
Origin Bruker BioSpin GmbH
Owner nmrsu
Instrument spect
Author Nicki
Solvent CDCl3
Temperature -7.0
Pulse Sequence zgpg30
Experiment 1D
Probe Z114607_0193 (PA BBO 600S3 BBF-H-D-05 Z SP)
Number of Scans 20480
Receiver Gain 190.9
Relaxation Delay 2.0000
Pulse Width 12.0000
Acquisition Time 0.3845
Acquisition Date 2022-10-22T22:20:58
Spectrometer Frequency 150.93
Spectral Width 42613.6
Lowest Frequency -3955.6
Nucleus 13C
Acquired Size 16384
Spectral Size 65536
Digital Resolution 0.65
36
f1 (ppm)

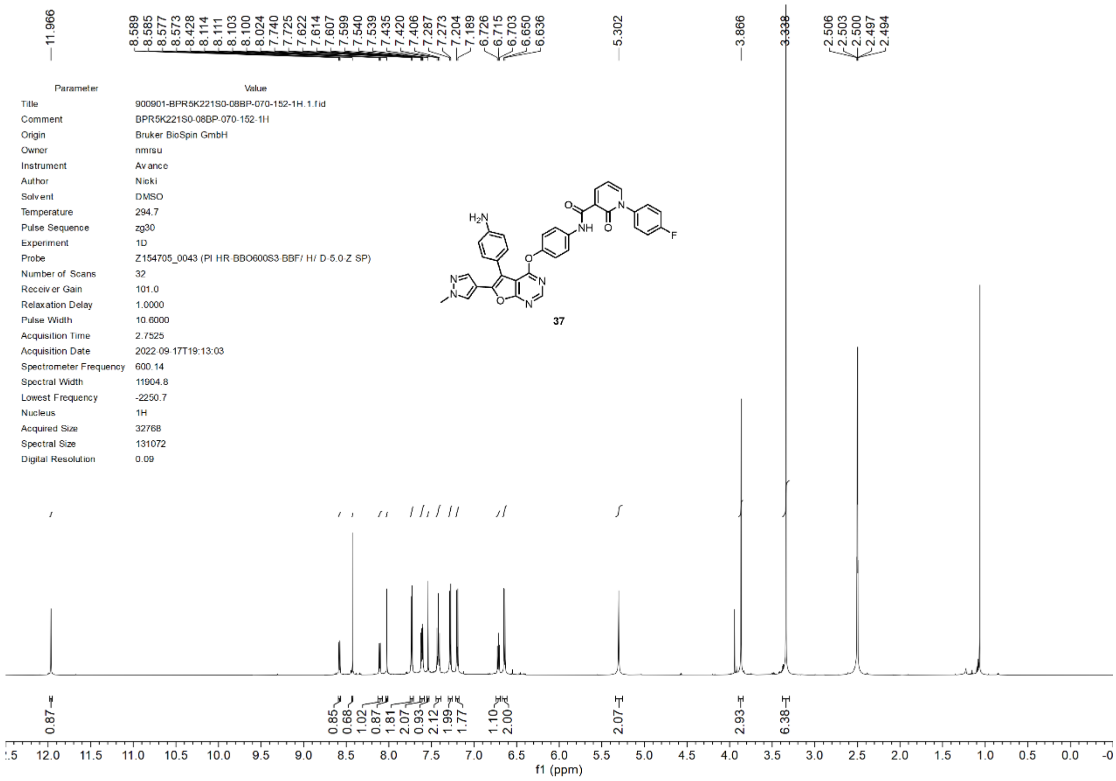
11.966
8.589 8.585 8.577 8.573 8.428 8.114 8.111 8.103 8.100 8.024 7.740 7.725 7.622 7.614 7.607 7.599 7.540 7.539 7.435 7.420 7.406 7.287 7.273 7.204 7.189 6.726 6.715 6.703 6.650 6.636
5.302
3.866
3.338
2.506 2.503 2.500 2.497 2.494
Parameter Value
Title 900901-BPR5K221S0-08BP-070-152-1H.1.fid
Comment BPR5K221S0-08BP-070-152-1H
Origin Bruker BioSpin GmbH
Owner nmrsu
Instrument Avance
Author Nicki
Solvent DMSO
Temperature 294.7
Pulse Sequence zg30
Experiment 1D
Probe Z154705_0043 (PI HR-BBO600S3-BBF/ H/ D-5.0 Z SP)
Number of Scans 32
Receiver Gain 101.0
Relaxation Delay 1.0000
Pulse Width 10.6000
Acquisition Time 2.7525
Acquisition Date 2022-09-17T19:13:03
Spectrometer Frequency 600.14
Spectral Width 11904.8
Lowest Frequency -2250.7
Nucleus 1H
Acquired Size 32768
Spectral Size 131072
Digital Resolution 0.09
H_2N
37
0.87 0.85 0.68 1.02 0.87 1.81 2.07 0.93 2.12 1.99 1.77 1.10 2.00 2.07 2.93 6.38
f1 (ppm)

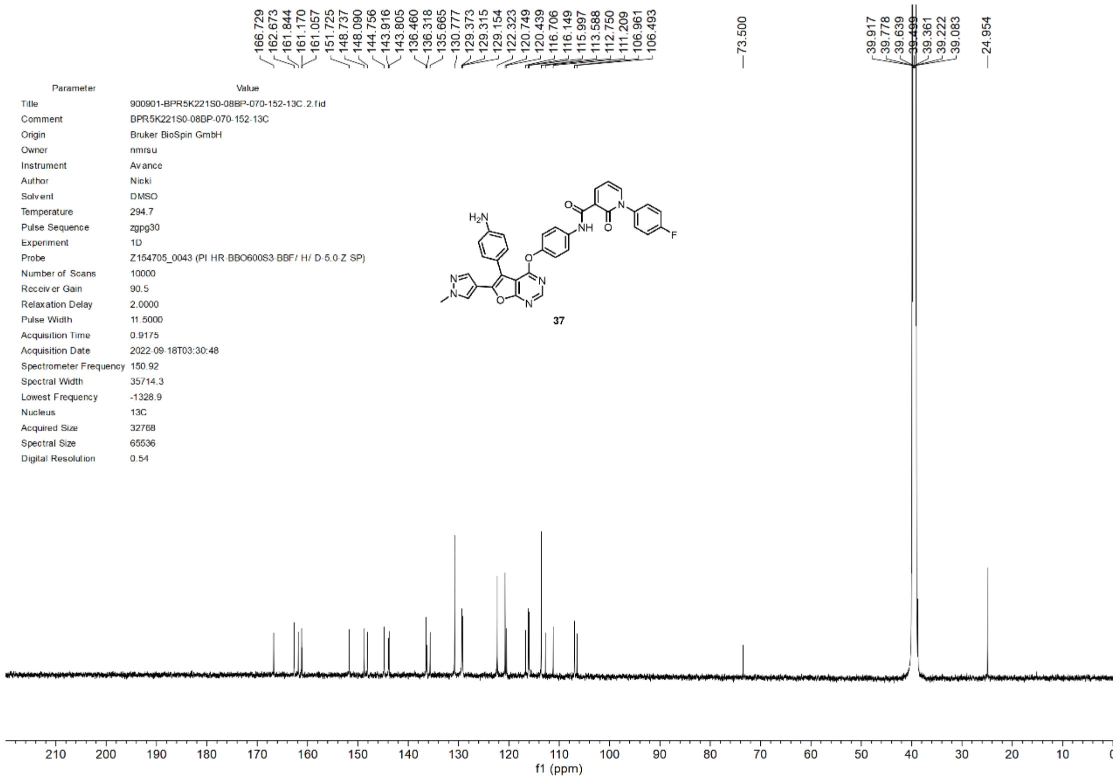
166.729 162.673 161.844 161.170 161.057 151.725 148.737 148.090 144.756 143.916 143.805 136.460 136.318 135.665 130.777 129.373 129.315 129.154 122.323 120.749 120.439 116.706 116.149 115.997 113.588 112.750 111.209 106.961 106.493
73.500
39.917 39.778 39.639 39.499 39.361 39.222 39.083
24.954
Parameter Value
Title 900901-BPR5K221S0-08BP-070-152-13C.2.fid
Comment BPR5K221S0-08BP-070-152-13C
Origin Bruker BioSpin GmbH
Owner nmrsu
Instrument Avance
Author Nicki
Solvent DMSO
Temperature 294.7
Pulse Sequence zgpg30
Experiment 1D
Probe Z154705_0043 (PI HR-BBO600S3-BBF/ H/ D-5.0 Z SP)
Number of Scans 10000
Receiver Gain 90.5
Relaxation Delay 2.0000
Pulse Width 11.5000
Acquisition Time 0.9175
Acquisition Date 2022-09-18T03:30:48
Spectrometer Frequency 150.92
Spectral Width 35714.3
Lowest Frequency -1328.9
Nucleus 13C
Acquired Size 32768
Spectral Size 65536
Digital Resolution 0.54
H_2N
37
f1 (ppm)

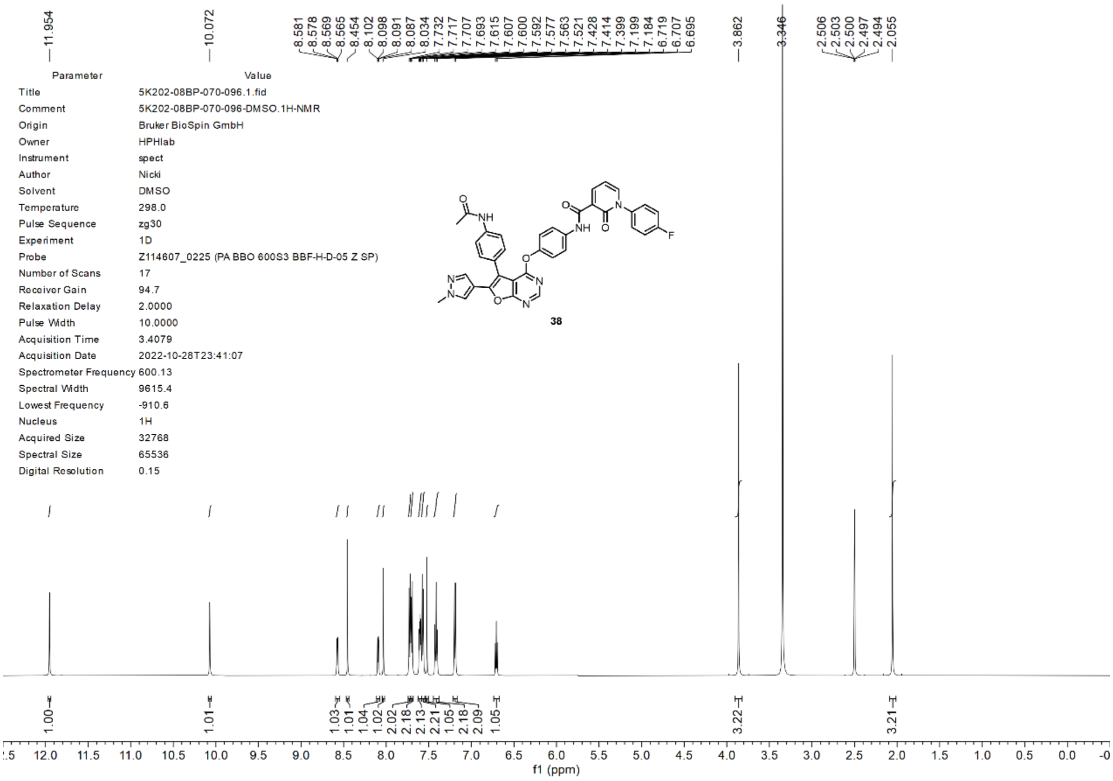

Parameter	Value
Title	5K202-08BP-070-096.1.fid
Comment	5K202-08BP-070-096-DMSO.1H-NMR
Origin	Bruker BioSpin GmbH
Owner	HPHlab
Instrument	spect
Author	Nicki
Solvent	DMSO
Temperature	298.0
Pulse Sequence	zg30
Experiment	1D
Probe	Z114607_0225 (PA BBO 600S3 BBF-H-D-05 Z SP)
Number of Scans	17
Receiver Gain	94.7
Relaxation Delay	2.0000
Pulse Width	10.0000
Acquisition Time	3.4079
Acquisition Date	2022-10-28T23:41:07
Spectrometer Frequency	600.13
Spectral Width	9615.4
Lowest Frequency	-910.6
Nucleus	1H
Acquired Size	32768
Spectral Size	65536
Digital Resolution	0.15

38

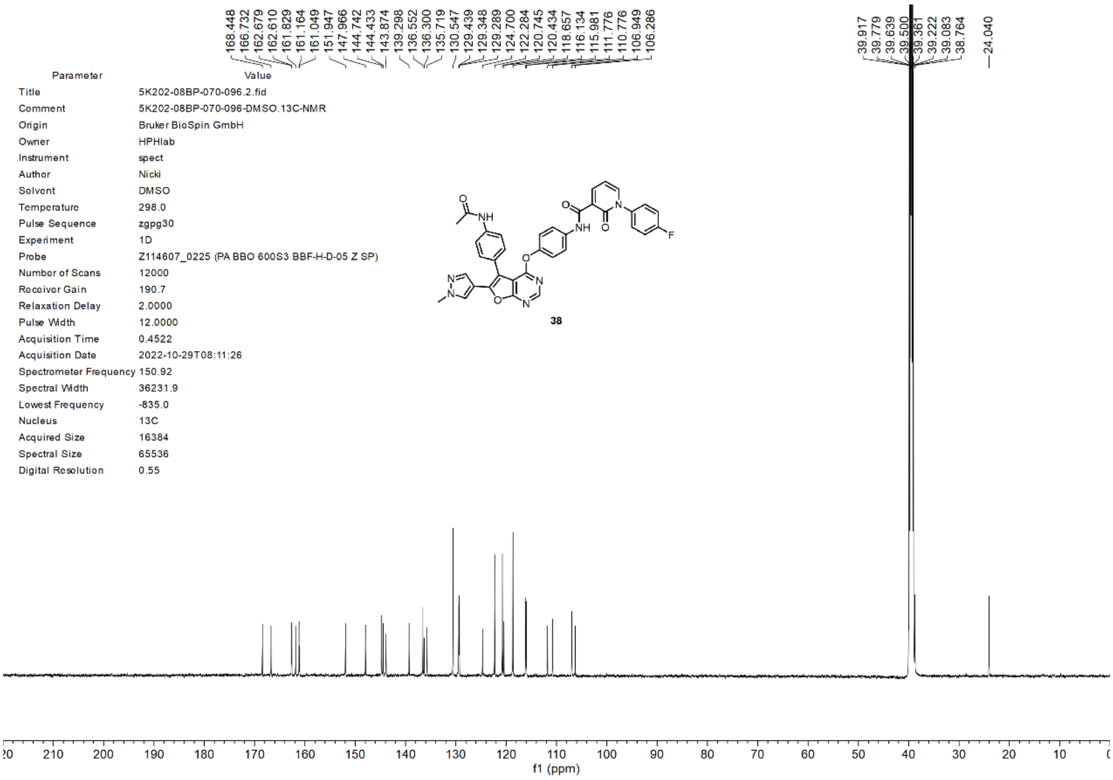

Parameter	Value
Title	5K202-08BP-070-096.2.fid
Comment	5K202-08BP-070-096-DMSO.13C-NMR
Origin	Bruker BioSpin GmbH
Owner	HPHlab
Instrument	spect
Author	Nicki
Solvent	DMSO
Temperature	298.0
Pulse Sequence	zgpg30
Experiment	1D
Probe	Z114607_0225 (PA BBO 600S3 BBF-H-D-05 Z SP)
Number of Scans	12000
Receiver Gain	190.7
Relaxation Delay	2.0000
Pulse Width	12.0000
Acquisition Time	0.4522
Acquisition Date	2022-10-29T08:11:26
Spectrometer Frequency	150.92
Spectral Width	36231.9
Lowest Frequency	-835.0
Nucleus	13C
Acquired Size	16384
Spectral Size	65536
Digital Resolution	0.55

38

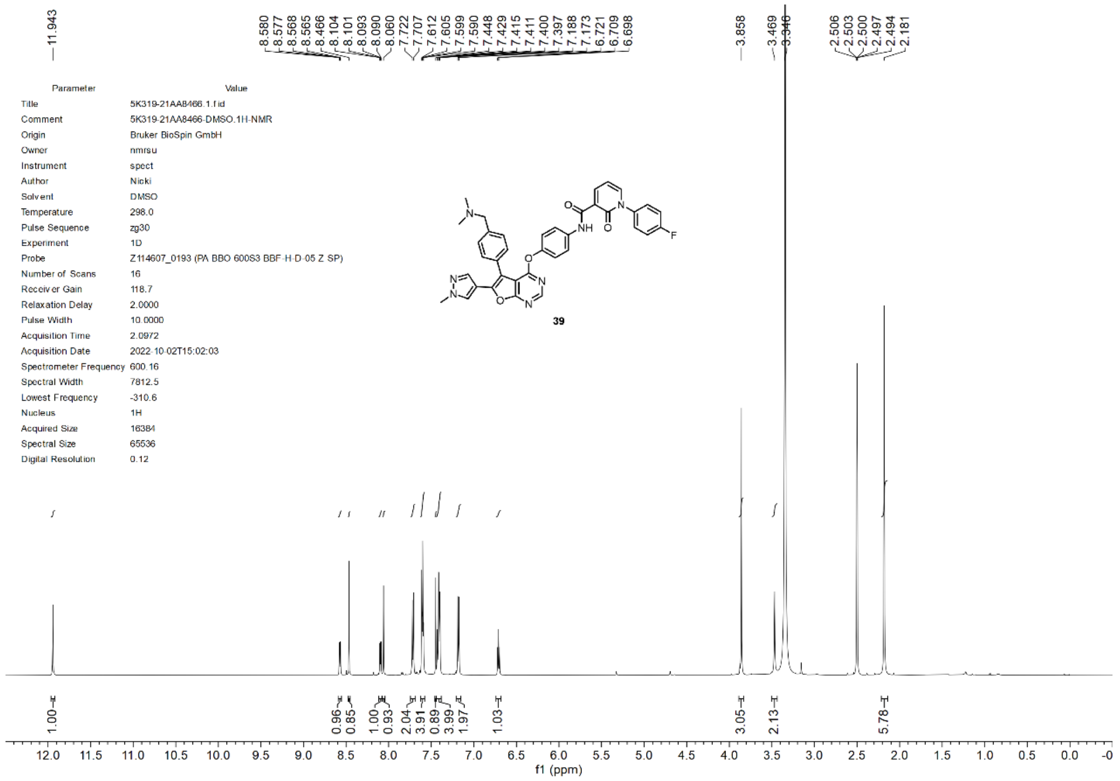

11.943
8.580 8.577 8.568 8.565 8.466 8.104 8.101 8.093 8.090 8.060 7.722 7.707 7.612 7.605 7.599 7.590 7.448 7.429 7.415 7.411 7.400 7.397 7.188 7.173 6.721 6.709 6.698
3.858
3.469
3.346
2.506 2.503 2.500 2.497 2.494 2.181
Parameter Value
Title 5K319-21AA8466.1.fid
Comment 5K319-21AA8466-DMSO.1H-NMR
Origin Bruker BioSpin GmbH
Owner nmrsu
Instrument spect
Author Nicki
Solvent DMSO
Temperature 298.0
Pulse Sequence zg30
Experiment 1D
Probe Z114607_0193 (PA BBO 600S3 BBF-H-D-05 Z SP)
Number of Scans 16
Receiver Gain 118.7
Relaxation Delay 2.0000
Pulse Width 10.0000
Acquisition Time 2.0972
Acquisition Date 2022-10-02T15:02:03
Spectrometer Frequency 600.16
Spectral Width 7812.5
Lowest Frequency -310.6
Nucleus 1H
Acquired Size 16384
Spectral Size 65536
Digital Resolution 0.12
39
1.00 0.96 0.85 1.00 0.93 2.04 3.91 0.89 3.99 1.97 1.03 3.05 2.13 5.78
f1 (ppm)

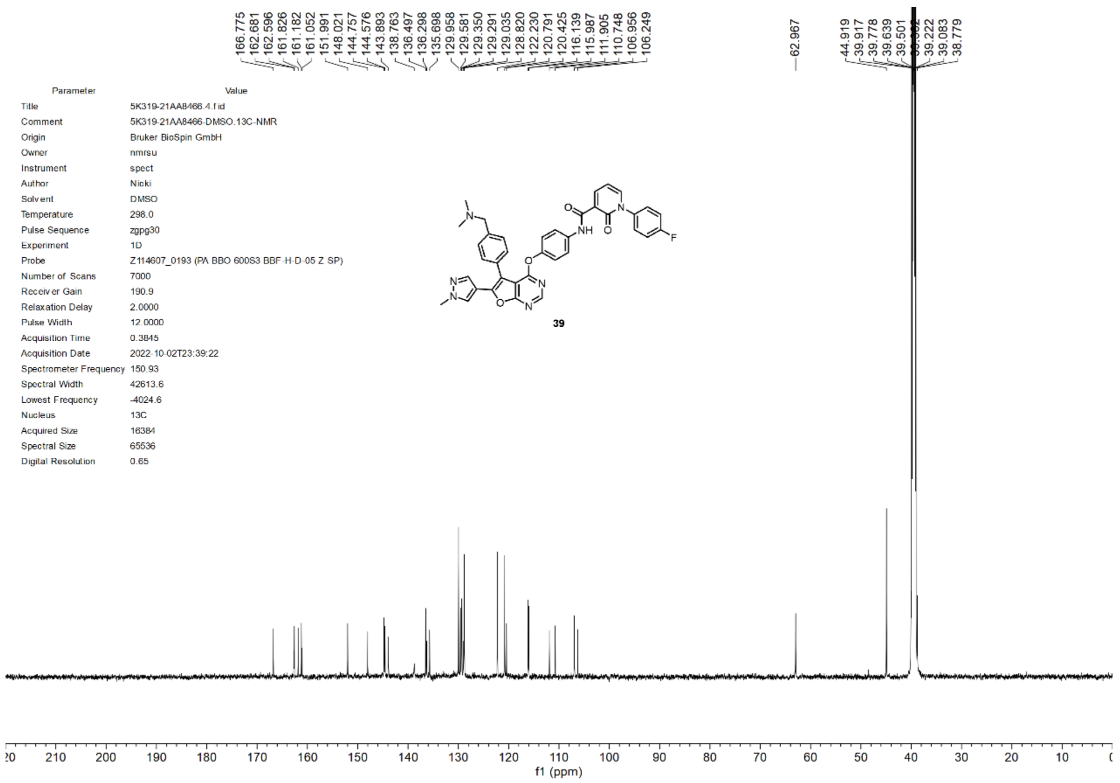

166.775 162.681 162.596 161.826 161.182 161.052 151.991 148.021 144.757 144.576 143.893 138.763 136.497 136.298 135.698 129.958 129.581 129.350 129.291 129.035 128.820 122.230 120.791 120.425 116.139 115.987 111.905 110.748 106.956 106.249
62.967
44.919 39.917 39.778 39.639 39.501 39.222 39.083 38.779
Parameter Value
Title 5K319-21AA8466.4.fid
Comment 5K319-21AA8466-DMSO.13C-NMR
Origin Bruker BioSpin GmbH
Owner nmrsu
Instrument spect
Author Nicki
Solvent DMSO
Temperature 298.0
Pulse Sequence zgpg30
Experiment 1D
Probe Z114607_0193 (PA BBO 600S3 BBF-H-D-05 Z SP)
Number of Scans 7000
Receiver Gain 190.9
Relaxation Delay 2.0000
Pulse Width 12.0000
Acquisition Time 0.3845
Acquisition Date 2022-10-02T23:39:22
Spectrometer Frequency 150.93
Spectral Width 42613.6
Lowest Frequency -4024.6
Nucleus 13C
Acquired Size 16384
Spectral Size 65536
Digital Resolution 0.65
39
f1 (ppm)

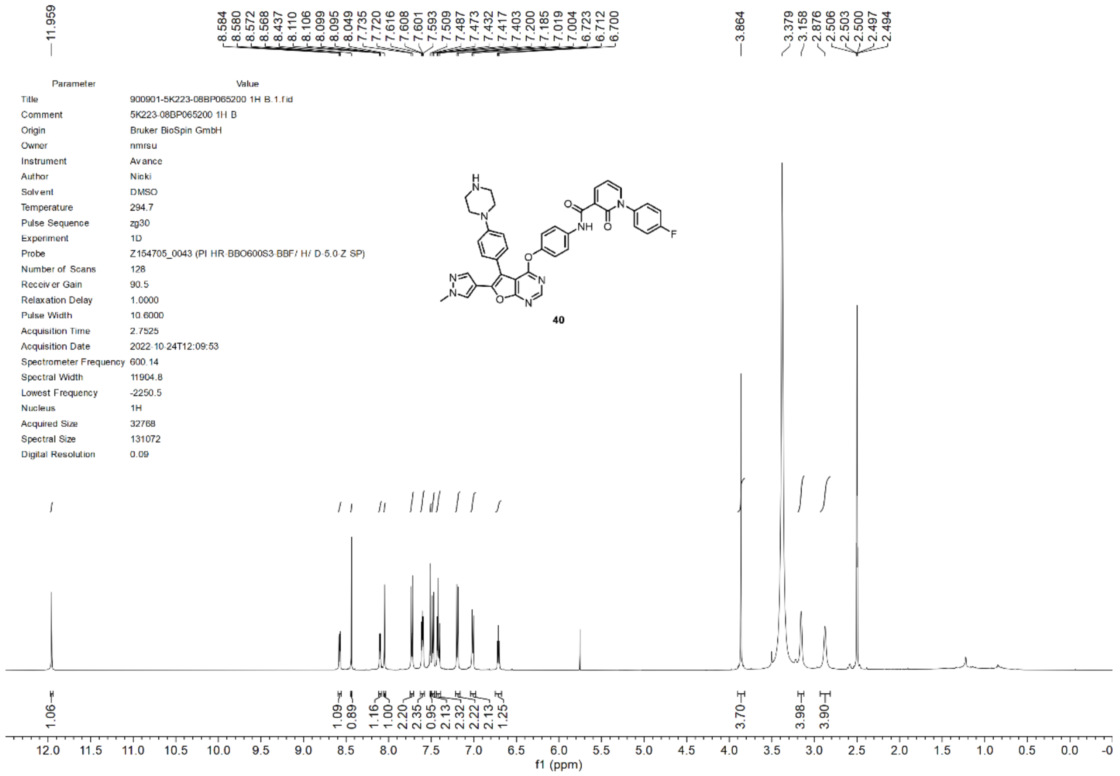
11.959
8.584
8.580
8.572
8.568
8.437
8.110
8.106
8.099
8.095
8.049
7.735
7.720
7.616
7.608
7.601
7.593
7.509
7.487
7.473
7.432
7.417
7.403
7.200
7.185
7.019
7.004
6.723
6.712
6.700
3.864
3.379
3.158
2.876
2.506
2.503
2.500
2.497
2.494
Parameter Value
Title 900901-5K223-08BP065200 1H B.1.fid
Comment 5K223-08BP065200 1H B
Origin Bruker BioSpin GmbH
Owner nmrsu
Instrument Avance
Author Nicki
Solvent DMSO
Temperature 294.7
Pulse Sequence zg30
Experiment 1D
Probe Z154705_0043 (PI HR-BBO600S3-BBF/ H/ D-5.0-Z SP)
Number of Scans 128
Receiver Gain 90.5
Relaxation Delay 1.0000
Pulse Width 10.6000
Acquisition Time 2.7525
Acquisition Date 2022-10-24T12:09:53
Spectrometer Frequency 600.14
Spectral Width 11904.8
Lowest Frequency -2250.5
Nucleus 1H
Acquired Size 32768
Spectral Size 131072
Digital Resolution 0.09
H
N
N
O
NH
O
N
O
F
N
N
O
N
N
40
1.06
1.09
0.89
1.16
1.00
2.20
2.35
0.95
2.13
2.32
2.22
2.13
1.25
3.70
3.98
3.90
12.0 11.5 11.0 10.5 10.0 9.5 9.0 8.5 8.0 7.5 7.0 6.5 6.0 5.5 5.0 4.5 4.0 3.5 3.0 2.5 2.0 1.5 1.0 0.5 0.0 -0
f1 (ppm)

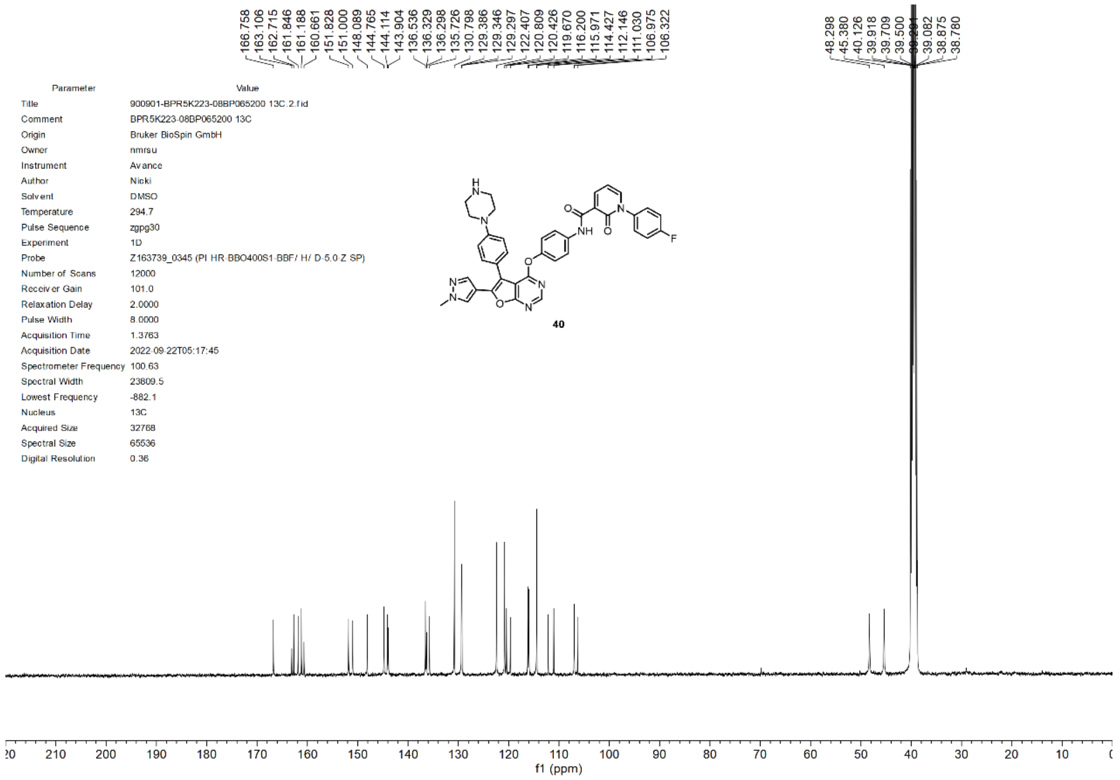
166.758
163.106
162.715
161.846
161.188
160.661
151.828
151.000
148.089
144.765
144.114
143.904
136.536
136.329
136.298
135.726
130.798
129.386
129.346
129.297
122.407
120.809
120.426
119.670
116.200
115.971
114.427
112.146
111.030
106.975
106.322
48.298
45.380
40.126
39.918
39.709
39.500
39.082
38.875
38.780
Parameter Value
Title 900901-BPR5K223-08BP065200 13C.2.fid
Comment BPR5K223-08BP065200 13C
Origin Bruker BioSpin GmbH
Owner nmrsu
Instrument Avance
Author Nicki
Solvent DMSO
Temperature 294.7
Pulse Sequence zgpg30
Experiment 1D
Probe Z163739_0345 (PI HR-BBO400S1-BBF/ H/ D-5.0-Z SP)
Number of Scans 12000
Receiver Gain 101.0
Relaxation Delay 2.0000
Pulse Width 8.0000
Acquisition Time 1.3763
Acquisition Date 2022-09-22T05:17:45
Spectrometer Frequency 100.63
Spectral Width 23809.5
Lowest Frequency -882.1
Nucleus 13C
Acquired Size 32768
Spectral Size 65536
Digital Resolution 0.36
H
N
N
O
NH
O
N
O
F
N
N
O
N
N
40
210 200 190 180 170 160 150 140 130 120 110 100 90 80 70 60 50 40 30 20 10
f1 (ppm)

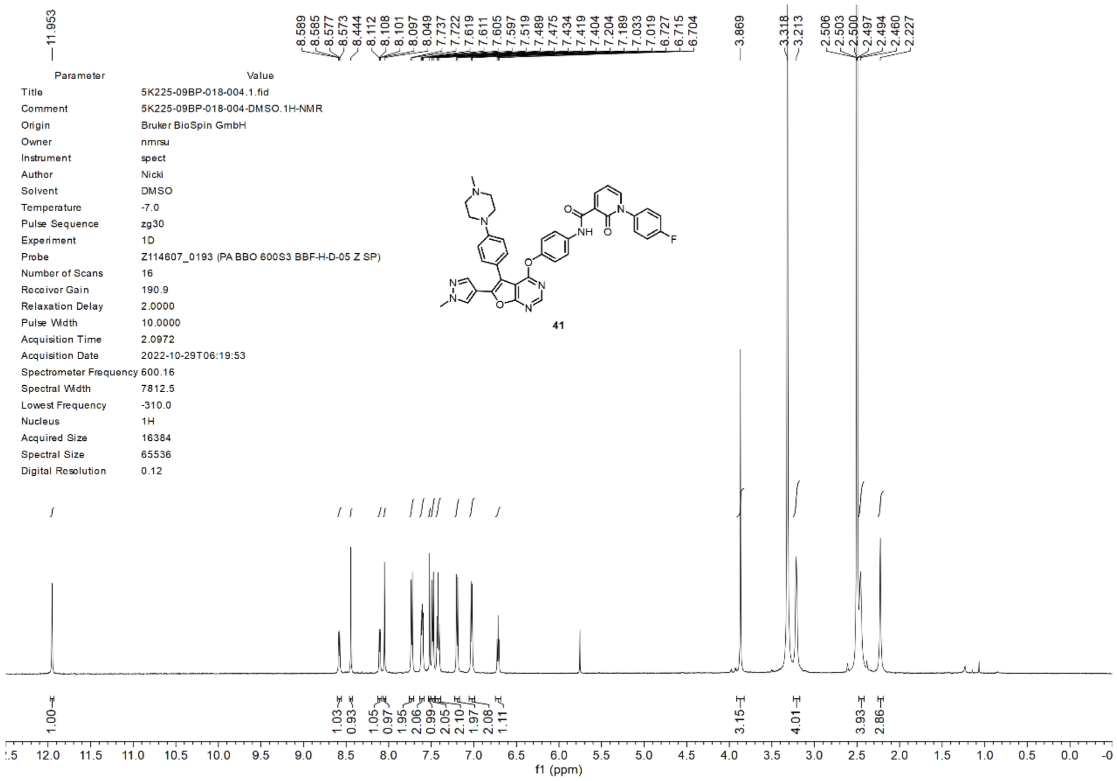
11.953
8.589 8.585 8.577 8.573 8.444 8.112 8.108 8.101 8.097 8.049 7.737 7.722 7.619 7.611 7.605 7.597 7.519 7.489 7.475 7.434 7.419 7.404 7.204 7.189 7.033 7.019 6.727 6.715 6.704
3.869 3.318 3.213 2.506 2.503 2.500 2.497 2.494 2.460 2.227
Parameter Value
Title 5K225-09BP-018-004.1.fid
Comment 5K225-09BP-018-004-DMSO.1H-NMR
Origin Bruker BioSpin GmbH
Owner nmrsu
Instrument spect
Author Nicki
Solvent DMSO
Temperature -7.0
Pulse Sequence zg30
Experiment 1D
Probe Z114607_0193 (PA BBO 600S3 BBF-H-D-05 Z SP)
Number of Scans 16
Receiver Gain 190.9
Relaxation Delay 2.0000
Pulse Width 10.0000
Acquisition Time 2.0972
Acquisition Date 2022-10-29T06:19:53
Spectrometer Frequency 600.16
Spectral Width 7812.5
Lowest Frequency -310.0
Nucleus 1H
Acquired Size 16384
Spectral Size 65536
Digital Resolution 0.12
41
1.00 1.03 0.93 1.05 0.97 1.95 2.06 0.99 2.05 2.10 1.97 2.08 1.11 3.15 4.01 3.93 2.86
f1 (ppm)

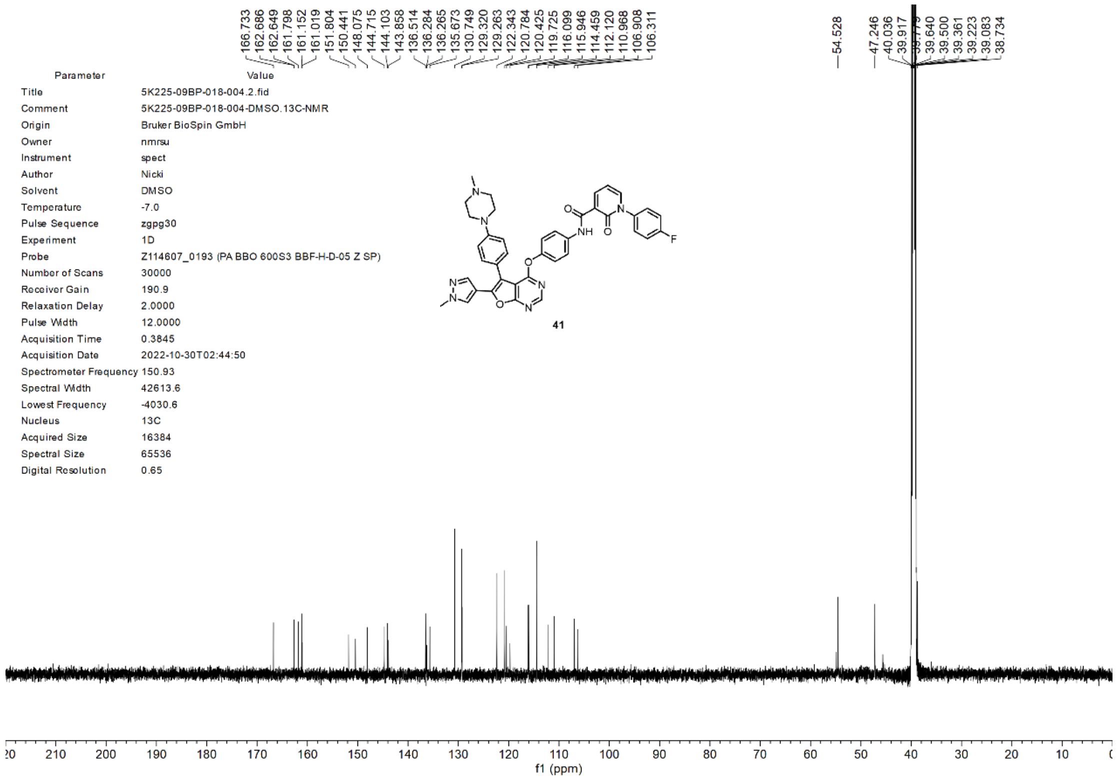
166.733 162.686 162.649 161.798 161.152 161.019 151.804 150.441 148.075 144.715 144.103 143.858 136.514 136.284 136.265 135.673 130.749 129.320 129.263 122.343 120.784 120.425 119.725 116.099 115.946 114.459 112.120 110.968 106.908 106.311
54.528 47.246 40.036 39.917 39.779 39.640 39.500 39.361 39.223 39.083 38.734
Parameter Value
Title 5K225-09BP-018-004.2.fid
Comment 5K225-09BP-018-004-DMSO.13C-NMR
Origin Bruker BioSpin GmbH
Owner nmrsu
Instrument spect
Author Nicki
Solvent DMSO
Temperature -7.0
Pulse Sequence zgpg30
Experiment 1D
Probe Z114607_0193 (PA BBO 600S3 BBF-H-D-05 Z SP)
Number of Scans 30000
Receiver Gain 190.9
Relaxation Delay 2.0000
Pulse Width 12.0000
Acquisition Time 0.3845
Acquisition Date 2022-10-30T02:44:50
Spectrometer Frequency 150.93
Spectral Width 42613.6
Lowest Frequency -4030.6
Nucleus 13C
Acquired Size 16384
Spectral Size 65536
Digital Resolution 0.65
41
f1 (ppm)

5. Kinase profiling data for 33

Table S1. Kinase profiling for **33** against a 658-kinase panel (containing 370 non-mutant kinases) at a concentration of 1 µM using the [33]PanQinase™ technology.

Kinase	% Enzyme Activity (relative to DMSO controls)
ABL1	89.75
ABL2/ARG	72.63
ACK1	70.54
AKT1	99.34
AKT2	92.50
AKT3	88.79
ALK	93.77
ALK1/ACVRL1	101.81
ALK2/ACVR1	101.08
ALK3/BMPR1A	84.96
ALK4/ACVR1B	111.83
ALK5/TGFBR1	97.63
ALK6/BMPR1B	101.84
ARAF	93.13
ARK5/NUAK1	104.85
ASK1/MAP3K5	100.91
AURORA A	92.26
AURORA B	109.65
AURORA C	94.88
AXL	2.37
BLK	36.25
BMPR2	106.84
BMX/ETK	58.73
BRAF	113.84
BRK	72.47
BRSK1	96.93
BRSK2	104.16
BTK	85.75
c-Kit	1.05
c-MER	-0.20
c-MET	15.42
c-Src	95.32
CAMK1a	108.84
CAMK1b	96.96
CAMK1d	93.44
CAMK1g	99.83

Kinase	% Enzyme Activity (relative to DMSO controls)
CAMK2a	89.32
CAMK2b	99.62
CAMK2d	97.32
CAMK2g	88.45
CAMK4	89.02
CAMKK1	94.15
CAMKK2	98.90
CDC7/DBF4	99.27
CDK1/cyclin A	101.23
CDK1/cyclin B	106.58
CDK1/cyclin E	99.73
CDK14/cyclin Y (PFTK1)	91.61
CDK16/cyclin Y (PCTAIRE)	123.94
CDK17/cyclin Y (PCTK2)	109.89
CDK18/cyclin Y (PCTK3)	96.29
CDK2/cyclin A	99.85
CDK2/cyclin A1	100.34
CDK2/CYCLIN E	105.32
CDK2/cyclin E2	93.28
CDK2/cyclin O	88.49
CDK3/cyclin E	114.29
CDK3/cyclin E2	100.54
CDK4/cyclin D1	101.93
CDK4/cyclin D2	92.19
CDK4/cyclin D3	102.98
CDK5/P25	98.89
CDK5/p35	104.37
CDK6/cyclin D1	104.82
CDK6/cyclin D2	120.65
CDK6/cyclin D3	45.07
CDK7/cyclin H	104.12
CDK9/CYCLIN K	102.96
CDK9/cyclin T1	92.02
CDK9/cyclin T2	94.31

Kinase	% Enzyme Activity (relative to DMSO controls)
CHK1	100.32
CHK2	86.40
CK1a1	93.71
CK1a1L	99.45
CK1d	112.07
CK1epsilon	96.69
CK1g1	100.63
CK1g2	106.85
CK1g3	91.87
CK2a	98.15
CK2a2	100.52
CLK1	96.60
CLK2	97.46
CLK3	99.99
CLK4	110.28
COT1/MAP3K8	106.30
CSK	96.79
CTK/MATK	94.26
DAPK1	108.83
DAPK2	102.45
DCAMKL1	98.34
DCAMKL2	103.30
DDR1	9.39
DDR2	4.45
DMPK	91.31
DMPK2	95.99
DRAK1/STK17A	97.82
DYRK1/DYRK1A	100.25
DYRK1B	107.85
DYRK2	100.47
DYRK3	100.33
DYRK4	103.26
EGFR	101.26
EPHA1	102.07
EPHA2	71.01
EPHA3	111.03
EPHA4	94.70
EPHA5	83.78
EPHA6	20.89

Kinase	% Enzyme Activity (relative to DMSO controls)
EPHA7	27.91
EPHA8	107.08
EPHB1	85.48
EPHB2	85.96
EPHB3	101.88
EPHB4	94.87
ERBB2/HER2	99.06
ERBB4/HER4	117.18
ERK1	117.18
ERK2/MAPK1	96.44
ERK5/MAPK7	101.53
ERK7/MAPK15	53.14
ERN1/IRE1	94.67
ERN2/IRE2	97.09
FAK/PTK2	94.72
FER	16.49
FES/FPS	62.50
FGFR1	90.56
FGFR2	79.53
FGFR3	94.04
FGFR4	94.33
FGR	26.15
FLT1/VEGFR1	83.00
FLT3	-0.03
FLT4/VEGFR3	54.16
FMS	16.13
FRK/PTK5	91.78
FYN	96.59
GCK/MAP4K2	102.39
GLK/MAP4K3	36.89
GRK1	99.81
GRK2	98.23
GRK3	105.83
GRK4	98.19
GRK5	96.74
GRK6	100.18
GRK7	99.70
GSK3a	103.89
GSK3b	97.94

Kinase	% Enzyme Activity (relative to DMSO controls)
Haspin	107.16
HCK	43.42
HGK/MAP4K4	100.95
HIPK1	101.37
HIPK2	96.97
HIPK3	95.85
HIPK4	11.68
HPK1/MAP4K1	69.22
IGF1R	60.53
IKKa/CHUK	103.02
IKKb/IKBKB	102.20
IKKe/IKBKE	97.52
IR	41.28
IRAK1	103.00
IRAK4	101.35
IRR/INSRR	1.79
ITK	102.37
JAK1	107.73
JAK2	96.39
JAK3	104.12
JNK1	100.32
JNK2	102.94
JNK3	104.96
KDR/VEGFR2	23.76
KHS/MAP4K5	62.48
KSR1	98.48
KSR2	98.07
LATS1	97.82
LATS2	94.93
LCK	33.21
LCK2/ICK	104.49
LIMK1	100.78
LIMK2	95.68
LKB1	100.14
LOK/STK10	28.06
LRRK2	90.32
LYN	34.66
LYN B	68.00
MAK	98.18

Kinase	% Enzyme Activity (relative to DMSO controls)
MAPKAPK2	100.20
MAPKAPK3	106.51
MAPKAPK5/PRAK	93.89
MARK1	101.13
MARK2/PAR-1Ba	94.88
MARK3	95.32
MARK4	103.88
MAST3	99.22
MASTL	101.29
MEK1	77.33
MEK2	67.45
MEK3	103.68
MEK5	94.13
MEKK1	97.39
MEKK2	85.26
MEKK3	112.96
MEKK6	88.96
MELK	90.72
MINK/MINK1	97.41
MKK4	117.78
MKK6	101.38
MKK7	102.64
MLCK/MYLK	100.29
MLCK2/MYLK2	101.23
MLK1/MAP3K9	54.61
MLK2/MAP3K10	58.40
MLK3/MAP3K11	30.45
MLK4	126.42
MNK1	87.01
MNK2	59.66
MRCKa/CDC42BPA	102.16
MRCKb/CDC42BPB	95.78
MSK1/RPS6KA5	104.86
MSK2/RPS6KA4	101.88
MSSK1/STK23	100.74
MST1/STK4	102.80
MST2/STK3	99.62
MST3/STK24	93.24
MST4	100.16

Kinase	% Enzyme Activity (relative to DMSO controls)
MUSK	11.08
MYLK3	94.94
MYLK4	100.51
MYO3A	104.58
MYO3b	96.97
NEK1	102.22
NEK11	98.13
NEK2	100.26
NEK3	103.07
NEK4	96.91
NEK5	101.31
NEK6	105.54
NEK7	105.38
NEK9	103.52
NIM1	99.32
NLK	83.40
OSR1/OXSR1	103.59
P38a/MAPK14	98.13
P38b/MAPK11	99.19
P38d/MAPK13	106.03
P38g	99.29
p70S6K/RPS6KB1	94.10
p70S6Kb/RPS6KB2	92.98
PAK1	94.32
PAK2	98.14
PAK3	105.11
PAK4	100.47
PAK5	97.74
PAK6	100.01
PASK	113.72
PBK/TOPK	116.04
PDGFRa	59.39
PDGFRb	80.90
PDK1/PDPK1	100.70
PHKg1	97.35
PHKg2	101.83
PIM1	95.18
PIM2	96.60
PIM3	109.03

Kinase	% Enzyme Activity (relative to DMSO controls)
PKA	102.92
PKAcb	100.89
PKAcg	98.98
PKCa	107.27
PKCb1	99.79
PKCb2	104.78
PKCd	100.12
PKCepsilon	99.25
PKCeta	93.61
PKCg	99.59
PKCIOTA	93.29
PKCmu/PRKD1	104.76
PKCnu/PRKD3	111.22
PKCtheta	97.79
PKCzeta	95.32
PKD2/PRKD2	96.95
PKG1a	99.73
PKG1b	104.84
PKG2/PRKG2	96.63
PKN1/PRK1	104.62
PKN2/PRK2	101.68
PKN3/PRK3	94.13
PLK1	94.02
PLK2	113.69
PLK3	108.81
PLK4/SAK	69.36
PRKX	97.95
PYK2	89.44
RAF1	99.04
RET	35.47
RIPK2	108.30
RIPK4	96.73
RIPK5	95.54
ROCK1	97.59
ROCK2	94.22
RON/MST1R	15.78
ROS/ROS1	2.88
RSK1	87.38
RSK2	100.93

Kinase	% Enzyme Activity (relative to DMSO controls)
RSK3	103.93
RSK4	104.48
SBK1	99.63
SGK1	104.94
SGK2	96.27
SGK3/SGKL	95.43
SIK1	74.98
SIK2	98.13
SIK3	105.16
SLK/STK2	90.50
SNARK/NUAK2	98.13
SNRK	95.97
SRMS	121.56
SRPK1	92.94
SRPK2	93.86
SSTK/TSSK6	98.10
STK16	106.55
STK21/CIT	100.65
STK22D/TSSK1	103.29
STK25/YSK1	101.84
STK32B/YANK2	104.01
STK32C/YANK3	97.81
STK33	81.18
STK38/NDR1	98.08
STK38L/NDR2	101.19
STK39/STLK3	106.07
SYK	99.88
TAK1	101.18
TAOK1	102.83
TAOK2/TAO1	93.32
TAOK3/JIK	103.79
TBK1	101.28
TEC	102.53
TESK1	97.81
TESK2	94.05
TGFBR2	95.08
TIE2/TEK	56.72
TLK1	100.15
TLK2	98.49

Kinase	% Enzyme Activity (relative to DMSO controls)
TNIK	100.05
TNK1	93.24
TRKA	3.35
TRKB	6.07
TRKC	2.26
TSSK2	101.39
TSSK3/STK22C	101.01
TTBK1	104.44
TTBK2	109.45
TXK	98.75
TYK1/LTK	101.03
TYK2	95.66
TYRO3/SKY	2.23
ULK1	103.92
ULK2	92.14
ULK3	91.52
VRK1	94.20
VRK2	93.26
WEE1	102.17
WNK1	124.25
WNK2	104.27
WNK3	82.00
YES/YES1	39.96
YSK4/MAP3K19	103.98
ZAK/MLTK	103.07
ZAP70	100.77
ZIPK/DAPK3	87.28
ABL1 (E255K)	103.79
ABL1 (E255V)	76.90
ABL1 (F317I)	97.18
ABL1 (F317L)	94.84
ABL1 (G250E)	97.69
ABL1 (H396P)	122.90
ABL1 (M351T)	111.13
ABL1 (Q252H)	95.73
ABL1 (T315I)	100.06
ABL1 (V299L)	71.21
ABL1 (Y253F)	106.22
ABL1 (Y253H)	92.53

Kinase	% Enzyme Activity (relative to DMSO controls)
AKT1 (E17K)	110.61
AKT2 (E17K)	98.69
AKT3 (E17K)	113.49
AKT3 (G171R)	94.30
ALK (C1156Y)	77.75
ALK (F1174L)	110.15
ALK (F1174L)-EML4	110.92
ALK (F1174L)-NPM1	103.92
ALK (F1174S)	82.88
ALK (G1202R)	85.60
ALK (G1269A)	22.57
ALK (G1269S)	67.68
ALK (L1152R)	67.28
ALK (L1196M)	110.67
ALK (R1275Q)	68.45
ALK (S1206R)	122.75
ALK (T1151-L1152insT)	106.02
ALK (T1151M)	84.49
ALK-KIF5B (Kex24Aex20)	90.81
ALK-KLC1 (Kex8Aex20)	73.19
ALK-NPM1	102.90
ALK-TFG	80.78
ALK-TFG (Tex4Aex20)	89.80
ALK-TPM1	89.61
ALK-TPM3	105.19
ALK2 (Q207D)	108.70
ALK2 (R206H)	107.35
Aurora B (G160L)	85.76
AXL (R499C)	3.19
BRAF (d485-489/P490Y)	94.50
BRAF (G464V)	102.03
BRAF (G469A)	98.92
BRAF (K601E)	106.06
BRAF (L597V)	111.85
BRAF (R506_K507insVLR)	105.92
BRAF (T599_V600insT)	114.52
BRAF (V599E)	107.17
BRAF (V600A)	110.54
BRAF (V600D)	105.89
BRAF (V600K)	107.26
BRAF-FAM131B (Fex2Bex9)	105.36
BRAF-KIAA1549 (Kex15Bex9)	109.08
BRAF-KIAA1549 (Kex16Bex9)	120.96
BRAF-SRGAP3 (Sex12Bex9)	107.87
BTK (C481S)	106.52
BTK (E41K)	87.65
BTK (P190K)	85.56
BTK (T474I)	64.00
BTK (Y485F)	89.82
c-Kit (A829P)	15.88
c-Kit (d557-558)	22.36
c-Kit (D816E)	20.57
c-Kit (D816F)	82.30
c-Kit (D816H)	92.87
c-Kit (D816I)	110.72
c-Kit (D816V)	95.90
c-Kit (D816Y)	106.54
c-Kit (D820E)	17.85
c-Kit (D820Y)	11.92
c-Kit (T670I)	12.00
c-Kit (V559A)	5.71
c-Kit (V559D)	10.92
c-Kit (V559D/T670I)	4.00
c-Kit (V559D/V654A)	91.10
c-Kit (V560G)	17.05
c-Kit (V560G/D816V)	96.03
c-Kit (V560G/N822K)	24.97
c-Kit (V654A)	65.55
c-Kit (Y823D)	6.78
c-MER (A708S)	1.38
c-MET (D1228A)	18.41
c-MET (D1228G)	11.11
c-MET (D1228H)	37.76

Kinase	% Enzyme Activity (relative to DMSO controls)
c-MET (D1228N)	28.59
c-MET (D1228V)	15.96
c-MET (D1228Y)	26.31
c-MET (F1200I)	56.24
c-MET (G1163R)	63.70
c-MET (H1094L)	22.25
c-MET (H1094Y)	21.35
c-MET (K1244R)	15.56
c-MET (L1195F)	49.91
c-MET (L1195V)	55.01
c-MET (M1250I)	12.57
c-MET (M1250T)	24.66
c-MET (P991S)	9.26
c-MET (T1173I)	9.08
c-MET (T992I)	5.56
c-MET (V1092I)	10.54
c-MET (Y1230A)	24.54
c-MET (Y1230C)	9.94
c-MET (Y1230D)	11.99
c-MET (Y1230H)	21.71
c-MET (Y1230S)	15.35
c-MET (Y1235D)	5.70
c-MET-KIF5B (Kex24Mex14)	8.72
c-MET-TFG (Tex5Mex15)	11.25
c-SRC (T341M)	87.24
c-Src (Y530F)	93.63
CHK2 (I157T)	90.74
CK1epsilon (R178C)	92.75
DDR2 (N456S)	1.78
DDR2 (T654M)	3.12
EGFR (A763_Y764insFHEA)	112.03
EGFR (A763_Y764insFQEA)	98.58
EGFR (A767_S768insTLA)	93.83
EGFR (C775S/T790M/L858R)	129.30
EGFR (C797A)	90.80

Kinase	% Enzyme Activity (relative to DMSO controls)
EGFR (C797S)	98.86
EGFR (C797S/L858R)	112.83
EGFR (d746)	115.50
EGFR (d746-750)	90.85
EGFR (d746-750/C775S/T790M/L858R)	112.31
EGFR (d746-750/C797A)	92.11
EGFR (d746-750/C797S)	94.68
EGFR (d746-750/T790M)	101.37
EGFR (d746-750/T790M/C797S)	105.23
EGFR (d746-750/T790M/C797S/L858R)	117.29
EGFR (d747-749)	87.06
EGFR (d747-749/A750P)	100.94
EGFR (d747-752/P753S)	99.45
EGFR (d752-759)	101.92
EGFR (D761Y)	94.17
EGFR (D770GY)	99.68
EGFR (D770_N771insNPG)	98.63
EGFR (D770_N771insNPG/T790M)	98.49
EGFR (G719C)	103.60
EGFR (G719D)	90.62
EGFR (G719S)	101.06
EGFR (K716A)	78.69
EGFR (K716A/C797S/L858R)	97.98
EGFR (K716A/T790M/C797S/L858R)	101.80
EGFR (K716Q/L718Q)	96.97
EGFR (K728A)	74.61
EGFR (K728A/T790M/C797S/L858R)	115.90
EGFR (L718Q)	86.41

Kinase	% Enzyme Activity (relative to DMSO controls)
EGFR (L747S)	103.97
EGFR (L792F)	95.15
EGFR (L792F/L858R)	86.42
EGFR (L792H)	104.41
EGFR (L792H/C797S/L858R)	106.15
EGFR (L858R)	95.89
EGFR (L858R/T790M)	93.41
EGFR (L861Q)	104.19
EGFR (N771_P772insH)	95.74
EGFR (R999A)	96.27
EGFR (T790M)	100.54
EGFR (T790M/C797S)	97.27
EGFR (T790M/C797S/L858R)	95.45
EGFR (T790M/L792F/C797S/L858R)	96.33
EGFR (T790M/L792F/L858R)	102.05
EGFR (T790M/L792H/C797S/L858R)	87.07
EGFR (T790M/L792H/L858R)	86.99
EGFR (V769_D770insGE)	84.06
ERBB2 (A775_G776insYVMA)	96.67
ERBB2 (D769H)	107.14
ERBB2 (D769Y)	87.64
ERBB2 (P1170A)	96.59
ERBB2 (P780_Y781insGSP)	109.61
ERBB2 (R896C)	102.32
ERBB2 (V777L)	125.15
ERBB2 (V777_G778insCG)	98.41
ERN1/IRE1 (R727A)	83.58
ERN1/IRE1 (R728A)	95.37

Kinase	% Enzyme Activity (relative to DMSO controls)
FGFR1 (V561M)	99.62
FGFR1OP-FGFR1	121.49
FGFR2 (C491A)	109.69
FGFR2 (C491F)	88.27
FGFR2 (C491S)	80.97
FGFR2 (E565G)	77.58
FGFR2 (K526E)	65.82
FGFR2 (K641R)	89.69
FGFR2 (K659N)	77.46
FGFR2 (N549H)	82.63
FGFR2 (R612T)	85.51
FGFR2 (V564F)	86.75
FGFR3 (G697C)	104.09
FGFR3 (K650E)	124.00
FGFR3 (K650M)	108.53
FGFR3 (K650Q)	99.11
FGFR3 (V555M)	110.16
FGFR4 (N535K)	116.40
FGFR4 (V550E)	115.60
FGFR4 (V550L)	111.74
FGFR4 (V550M)	95.22
FLT3 (D835Y)	17.97
FLT3 (F594_R595insR)	9.63
FLT3 (F594_R595insREY)	9.19
FLT3 (ITD)	3.29
FLT3 (ITD)-NPOS	8.47
FLT3 (ITD)-W51	7.40
FLT3 (R595_E596insEY	4.55
FLT3 (Y591-V592insVDFREYEYD)	5.82
FYN (Y531F)	74.29
JAK2 (V617F)	120.73
KSR1 (A635F)	107.18
KSR1 (L639F)	94.20
KSR2 (R676S)	99.05
LRRK2 (G2019S)	91.45
LRRK2 (I2020T)	80.31
LRRK2 (R1441C)	92.86

Kinase	% Enzyme Activity (relative to DMSO controls)
MEK1 (P124L)	79.41
MELK (T460M)	102.21
P38a (T106M)	105.12
PDGFRa (D842V)	67.73
PDGFRa (T674I)	4.31
PDGFRa (V561D)	20.90
PDGFRa-FIP1L1	43.15
PDGFRb-TPM3	91.78
PKA (L206R)	96.16
PKA-DNAJB1	95.74
PKD2 (G870E)	107.25
PKMzeta	95.98
PKN1-TECR (Tex1Pex10)	119.30
RAF1 (R391W)	81.19
RET (A883F)	27.40
RET (E762Q)	54.78
RET (G691S)	48.63
RET (G810C)	89.49
RET (G810R)	85.29
RET (G810S)	11.90
RET (L790F)	7.31
RET (M918T)	94.23
RET (R749T)	34.25
RET (R813Q)	35.49
RET (R912P)	85.72
RET (S891A)	17.07
RET (S904A)	19.26
RET (S904F)	34.87
RET (V778I)	53.38
RET (V804E)	108.15
RET (V804L)	80.06
RET (V804L)-KIF5B	82.01
RET (V804M)	100.03
RET (V804M)-KIF5B	86.91
RET (Y791F)	19.58
RET (Y806H)	85.89
RET-BCR	38.03
RET-CCDC6 (PTC1)	27.40

Kinase	% Enzyme Activity (relative to DMSO controls)
RET-KIF5B (Kex15Rex14)	32.27
RET-NCOA4 (PTC3)	35.53
RET-PRKAR1A (PTC2)	33.17
ROS1 (G2032R)	51.11
ROS1 (G2101A)	1.02
ROS1 (G2101C)	0.35
ROS1-GOPC	2.46
ROS1-TPM3	33.39
RSK2 (I416V)	101.84
RSK2 (L608F)	95.00
TIE2 (A1124V)	19.58
TIE2 (P883A)	12.68
TIE2 (R849W)	59.01
TIE2 (Y1108F)	48.53
TIE2 (Y897C)	40.91
TIE2 (Y897S)	27.40
TRKA (A608D)	3.93
TRKA (F589L)	12.02
TRKA (G595R)	104.51
TRKA (G595R/A608D)	72.70
TRKA (G595R/G667A)	24.94
TRKA (G595R/G667C)	29.11
TRKA (G595R/G667S)	63.20
TRKA (G595R/L657M)	74.78
TRKA (G667C)	0.19
TRKA (G667S)	2.15
TRKA (L657M)	6.09
TRKA-TFG (TRK-T3)	1.41
TRKA-TPM3	1.57
TRKA-TPR	2.41
TRKC (G623E)	70.79
TRKC (G623R)	79.54
TRKC (G623R/L686M)	93.11
TRKC (G696A)	0.80
TRKC (L686M)	32.73
YES1 (T348I)	66.86
ZAP70 (Y319F)	100.24

Table S2. *S*-score results for **33**.

Compound	Selectivity Score Type	Number of Hits	Number of Non-Mutant Kinases	Screening Concentration (nM)	Selectivity Score
**33**	*S*(35)	26	370	1000	0.070
**33**	*S*(10)	12	370	1000	0.032
**33**	*S*(1)	2	370	1000	0.005

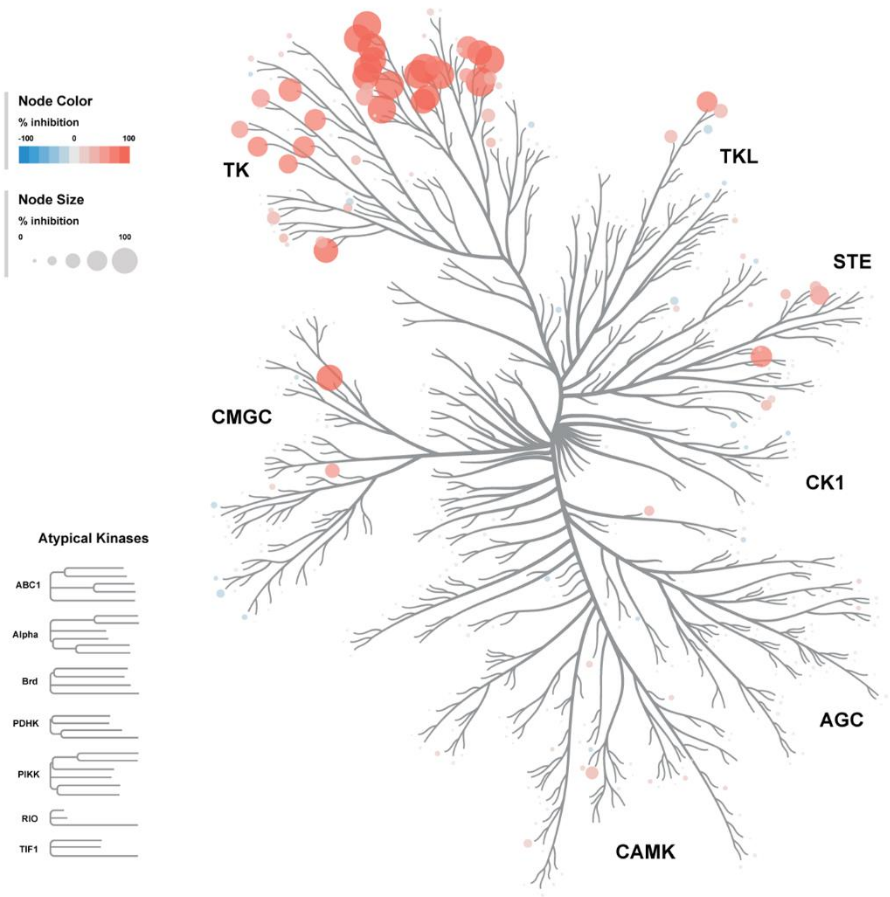

Figure S12. Kinase profiling of 33 using HotSpot™ kinase screening at a concentration of 1 μM.

Table S3. pIC_{50} determination of **33**.[a]

Kinase	pIC_{50}	Kinase	pIC_{50}
MER	10.26	KIT	10.21
AXL	11.29	IRR	8.05
TYRO3	10.87	ROS1	10.92
DDR1	8.36	TRKA	9.03
DDR2	10.59	TRKB	8.57
FLT3	9.37	TRKC	9.70

6. HPLC trace of 22 and 33

Advanced Individual Report

SAMPLE INFORMATION

Sample Name:	EXP-21-AA8079-50	Acquired By:	uplc
Sample Type:	Unknown	Sample Set Name	Purity_Test_STOP
Vial:	2:A,8	Acq. Method Set:	5cm Std
Injection #:	1	Processing Methoc	Def_Processing_Method
Injection Volume:	5.00 ul	Channel Name:	PDA Ch1 254nm@4.8nm
Run Time:	6.5 Minutes	Proc. Chnl. Descr.:	PDA Ch1 254nm@4.8nm
Date Acquired:	2021/4/20 AM 10:33:58 CST		
Date Processed:	2021/4/20 PM 01:12:42 CST		

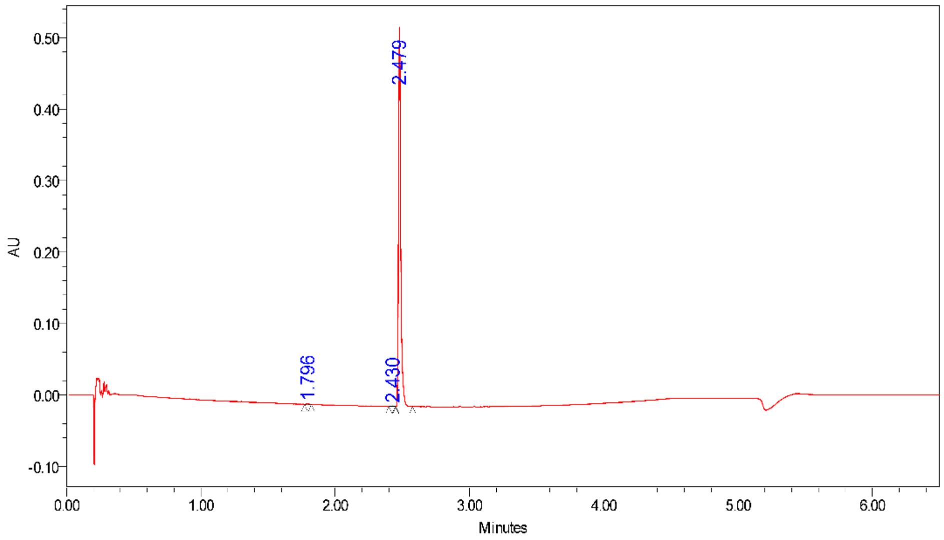

	RT	Height	Area	% Area
1	1.796	726	1061	0.17
2	2.430	666	961	0.16
3	2.479	529997	613002	99.67

Reported by User: uplc (uplc)
Report Method: Advanced Individual Rep
Report Method I[6669
Page: 1 of 4

Project Name: Robert
Date Printed:
2021/4/20
下午 01:14:30 Asia/Taipei

Figure S13. UPLC trace of **22**.

D-2000: Samples Series: 0127 Report Name: modified System: Sys 1

Analyzed Date and Time: 2020/03/30 07:00 下午
Reported Date and Time: 2020/03/31 08:55 上午
Data Path: C:\Win32app\D2000HSM\samples\DATA\0127\
Processing Method: Purity_37min

Sample Name: 08BP-070-176-50 Vial Number: 162
Injection from this vial: 1 of 1 Volume: 20.0 ul
Sample Description:

Chrom Type: Fixed WL Chromatogram, 254 nm

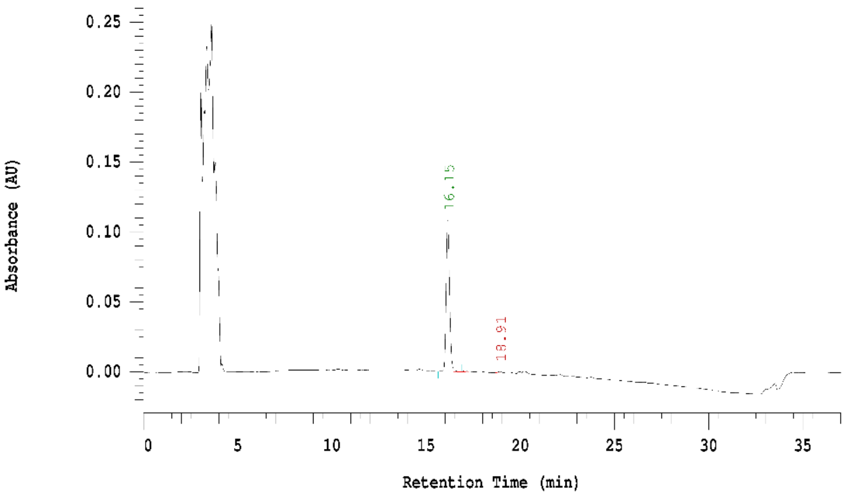

Processing Method: Purity_37min
Column Type: Column Method Developer: Bob
Method Description:

Peak Quantitation: AREA
Calculation Method: AREA%

No.	RT	Area	Height	Conc 1
1	16.15	673775	54038	98.636
2	18.91	9315	558	1.364
		683090	54596	100.000

Peak rejection level: 0

Page Indicator: 1 / 3

Figure S14. HPLC trace of **33**.
